# Synthesis and Antiviral Activities of Neoechinulin B and Its Derivatives

**DOI:** 10.1021/acs.jnatprod.1c01120

**Published:** 2021-12-30

**Authors:** Kota Nishiuchi, Hirofumi Ohashi, Kazane Nishioka, Masako Yamasaki, Masateru Furuta, Takumi Mashiko, Shusuke Tomoshige, Kenji Ohgane, Shinji Kamisuki, Koichi Watashi, Kouji Kuramochi

**Affiliations:** † Department of Applied Biological Science, Tokyo University of Science, 2641 Yamazaki, Noda, Chiba 278-8510, Japan; ‡ Department of Virology II, National Institute of Infectious Diseases, 1-23-1 Toyama, Shinjuku-ku, Tokyo 162-8640, Japan; § School of Veterinary Medicine and Center for Human and Animal Symbiosis Science, Azabu University, 1-17-71 Fuchinobe, Chuo-ku, Sagamihara, Kanagawa 252-5201, Japan; ⊥ Research Center for Drug and Vaccine Development, National Institute of Infectious Diseases, 1-23-1 Toyama, Shinjuku-ku, Tokyo 162-8640, Japan

## Abstract

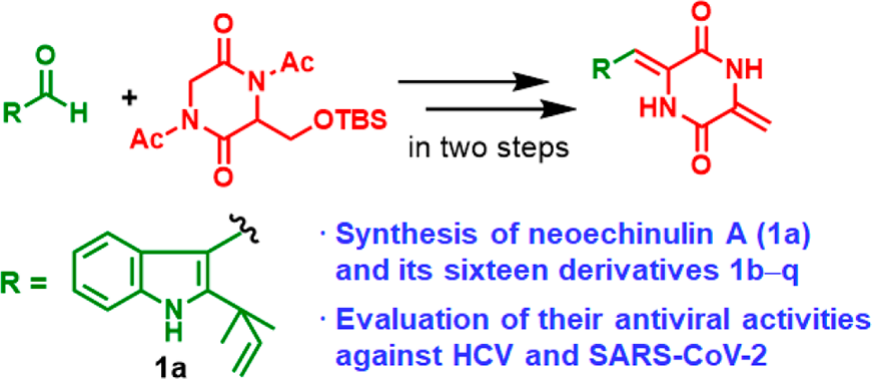

We have previously reported that neoechinulin B (**1a**), a prenylated indole diketopiperazine alkaloid, shows antiviral activities against hepatitis C virus (HCV) via the inactivation of the liver X receptors (LXRs) and the resultant disruption of double-membrane vesicles. In this study, a two-step synthesis of the diketopiperazine scaffold of **1a** was achieved by the base-induced coupling of 1,4-diacetyl-3-{[*(tert*-butyldimethylsilyl)oxy]methyl}piperazine-2,5-dione with aldehydes, followed by the treatment of the resultant coupling products with tetra-*n*-butylammonium fluoride. Compound **1a** and its 16 derivatives **1b**–**q** were prepared using this method. Furthermore, variecolorin H, a related alkaloid, was obtained by the acid treatment of **1a** in MeOH. The antiviral evaluation of **1a** and its derivatives revealed that **1a**, **1c**, **1d**, **1h**, **1j**, **1l**, and **1o** exhibited both anti-HCV and anti-severe acute respiratory syndrome coronavirus 2 (SARS-CoV-2) activities. The results of this study indicate that the exomethylene moiety on the diketopiperazine ring is important for the antiviral activities. The antiviral compounds can inhibit the production of HCV and SARS-CoV-2 by inactivating LXRs.

The diversity and complexity of natural products afford remarkable efficacy and specificity to target viral infections. Therefore, natural products serve as excellent sources for discovering antiviral agents.^1^ We have previously reported that neoechinulin B (**1a**), isolated from *Eurotium rubrum* Hiji025,^2^ exhibited antiviral effects against hepatitis C virus (HCV).^3^ Mechanistic studies revealed that this compound disrupted the formation of double-membrane vesicles (DMVs), which are the sites of viral RNA replication, by inhibiting the liver X receptor (LXR)-regulated gene induction required for DMV formation. Consistent with the unique mechanism of action targeting LXRs, which are those of the host-encoded proteins that HCV hijacks during its replication, compound **1a** augmented the antiviral activity of the approved anti-HCV agents that target viral proteins via combination treatment. Notably, **1a** reduced the RNA replication of poliovirus in DMVs.^3^ This compound was also reported to inhibit the entry of influenza A virus subtype H1N1 by targeting viral hemagglutinin.^4^ These reports suggest that **1a** and its derivatives are potential broad-spectrum antiviral drugs.
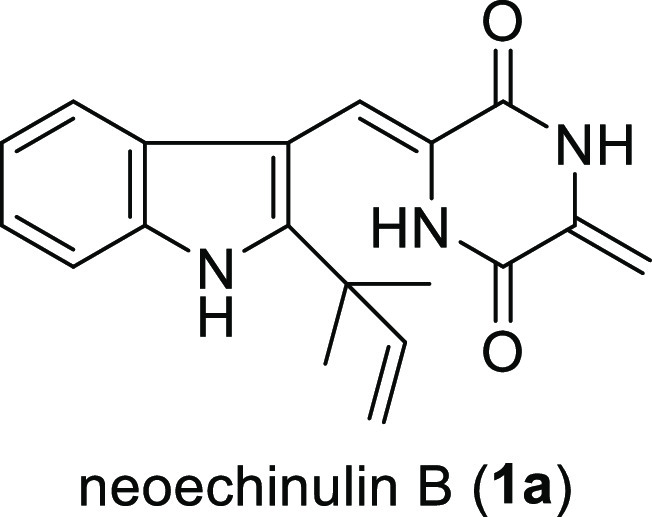


Despite its potential as a lead compound for a new class of antiviral agents, only one report by Inoue, Kishi, and co-workers in 1977 has described the chemical synthesis of **1a** (Scheme 1).^5^ A key step in its synthesis involved the coupling between aldehyde **2a** and diketopiperazine **3** in dry piperidine at 110 °C, which afforded **1a** in 45% yield. This reaction condition was also employed by Kuttruff, Zipse, and Trauner to prepare **1a** as a key building block in the synthesis of variecolortide B.^6^ However, there have been no attempts to systematically synthesize neoechinulin B derivatives using this protocol. Thus, in this study, we attempted to develop an alternative synthetic route to **1a** and its derivatives under mild conditions. Building upon our previous findings, extensive structure–activity relationship studies were also conducted to identify simplified derivatives with greater potency against HCV. In addition, the LXR antagonistic activity of the synthesized compounds was evaluated to investigate the mechanisms of the anti-HCV activity.

**Scheme 1:**
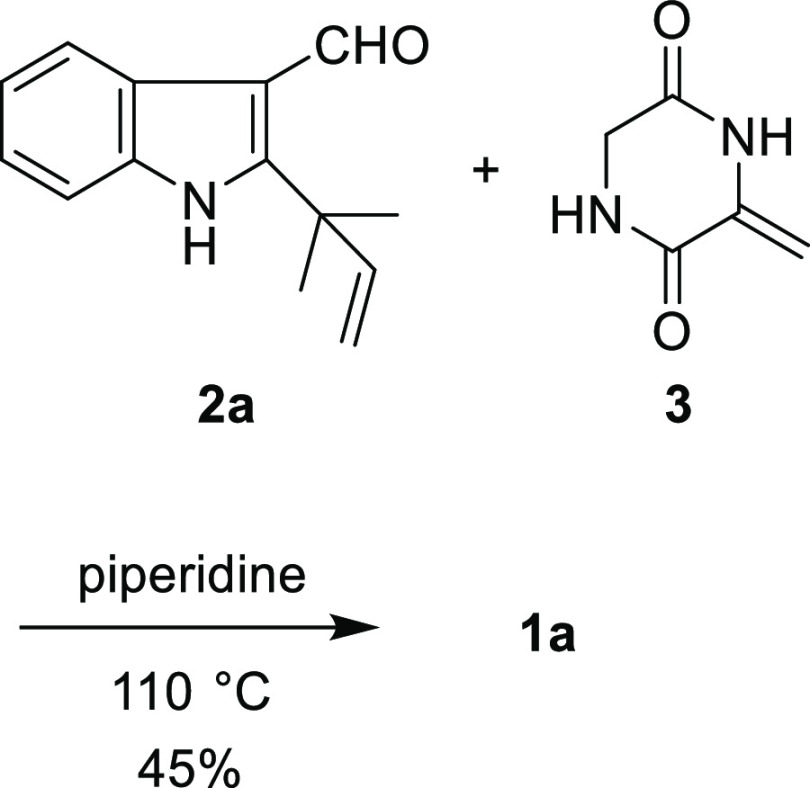
Synthesis of Neoechinulin B (**1a**) Reported by Inoue, Kishi, and Co-workers

Herein, the synthesis of **1a** and a series of simplified derivatives was achieved. Antiviral activities of these compounds against HCV and severe acute respiratory syndrome coronavirus 2 (SARS-CoV-2) are described. In addition to HCV and poliovirus, SARS-CoV-2 replicates genome RNA in DMVs in the infected cells.^7−9^ Therefore, the antiviral activity of **1a** and its derivatives against SARS-CoV-2 was evaluated in this study. As expected, **1a** exhibited both anti-HCV and anti-SARS-CoV-2 activities. Furthermore, the simplification of **1a** afforded a series of derivatives that exhibited more potent antiviral activities against both anti-HCV and anti-SARS-CoV-2.

## Results and Discussion

### Synthetic Approach

**Scheme 2:**
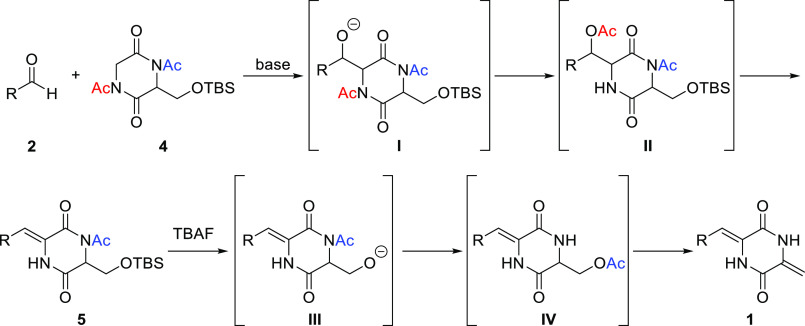
Synthetic Approach toward 2,5-Diketopiperazine Core **1**

It was envisioned that 2,5-diketopiperazine core **1** could be synthesized using aldehyde **2** and 1,4-diacetyl-3-{[(*tert*-butyldimethylsilyl)oxy]methyl}piperazine-2,5-dione (**4**)^10^ in two steps (Scheme 2). The base-induced coupling of **2** with **4** produces intermediate **I**. The migration of the acetyl group, followed by the elimination of the acetoxy group in the resultant intermediate **II**, affords **5**.^11^ Similarly, the removal of the *tert*-butyldimethylsilyl (TBS) group in **5** and migration of the acetyl group in the resultant intermediate **III**, followed by the elimination of the acetoxy group in intermediate **IV**, affords **1**.

### Synthesis of Deprenylneoechinulin B (**1b**) and 3-Arylmethylene-6-methylenepiperazine-2,5-diones

**Scheme 3:**
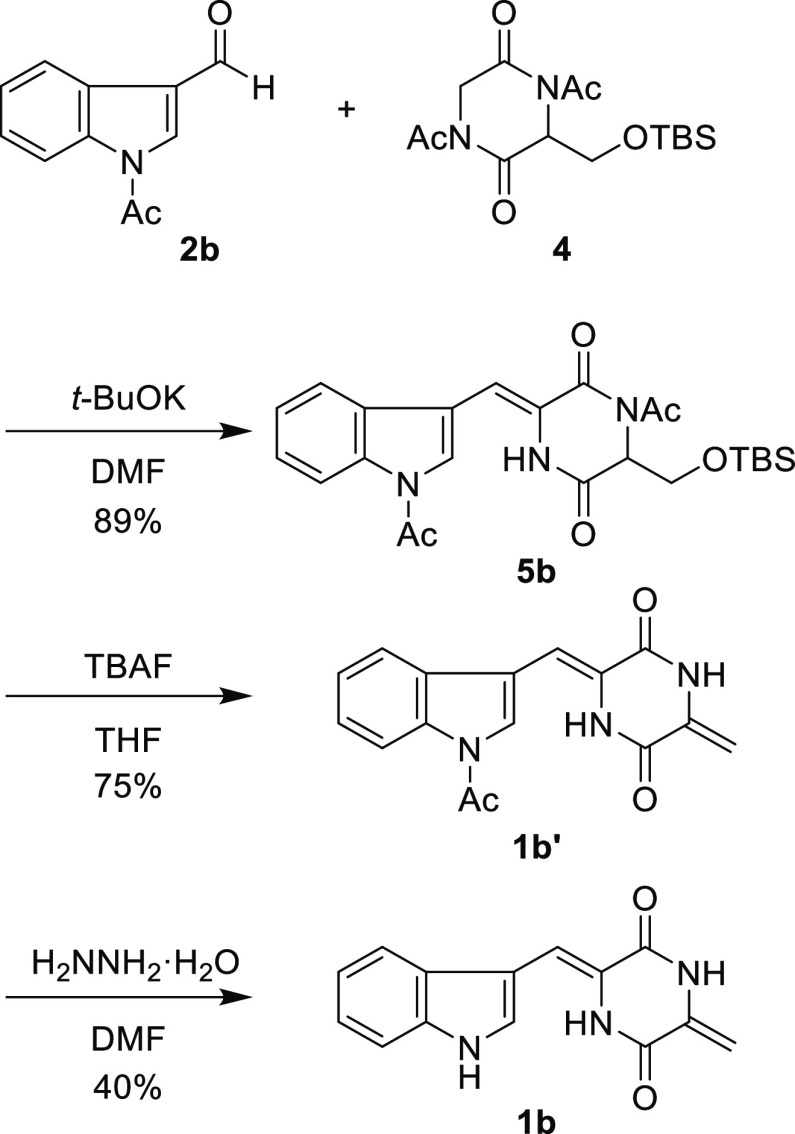
Synthesis of Deprenylneoechinulin B (**1b**)

**Scheme 4:**
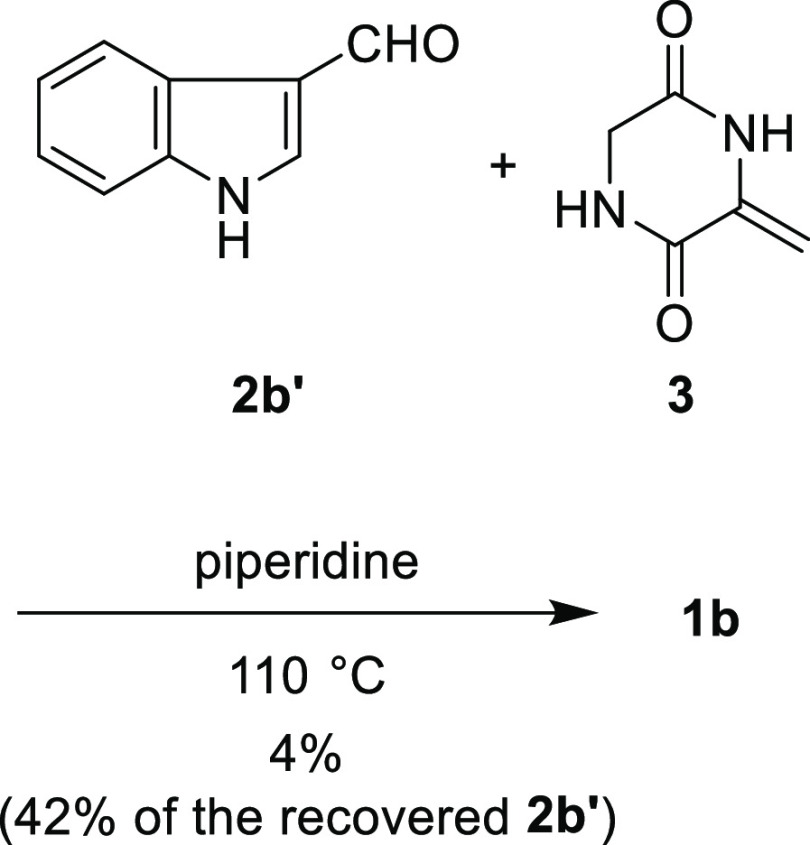
Synthesis of **1b** According to the Protocol Reported by Inoue, Kishi, and Co-workers Reaction was performed with **2b′** (0.20 mmol) and **3** (0.40 mmol) in piperidine (2.0 mL) according to the reported procedure.^5^

As a model compound, **1b** was synthesized using the procedure shown in Scheme 2. Treatment of *N*-acetyl-3-indolecarboxaldehyde (**2b**) and **4** with *t*-BuOK in *N*,*N*-dimethylformamide (DMF) afforded **5b** in 89% yield (Scheme 3). The subsequent treatment of **5b** with tetra-*n*-butylammonium fluoride (TBAF) afforded the desired compound **1b′** in 75% yield. The deacetylation of **1b′** by hydrazine monohydrate in DMF generated **1b** in 40% yield. To demonstrate the utility of this method, **1b** was also synthesized according to the protocol reported by Inoue, Kishi, and co-workers (Scheme 4). Coupling between **2b′** and **3** in piperidine at 110 °C afforded the desired compound **1b** in only 4% yield. One of the starting materials, **2b′**, was also recovered in 42% yield, and the rest of the compounds underwent side reactions to form unidentified byproducts. These results indicate that the alternative method described herein can be used for the synthesis of 2,5-diketopiperazine core **1**.

**Scheme 5:**
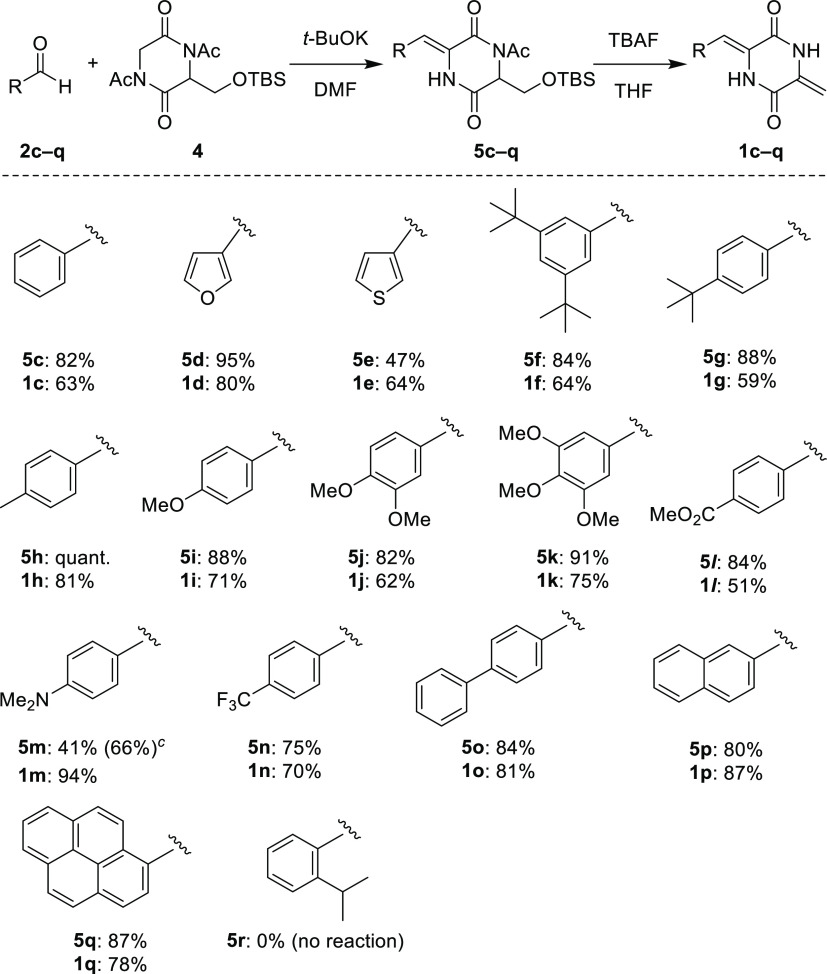
Coupling of Aldehydes **2c**–**r** with Diketopiperazine **4** and Transformation of Intermediates **5c**–**q** into 3-Arylmethylene-6-methylenepiperazines **1c**–**q**^,^ Reaction was performed with **2** (0.20 mmol), **4** (0.40 mmol), and *t*-BuOK (0.58 mmol) in DMF (2.0 mL) at rt. Isolated yields are presented, unless otherwise stated. The yield based on recovered **2m** is included in parentheses.

Next, the scope and limitations of this method were examined (Scheme 5). The present method was found to be applicable to the synthesis of a series of 3-arylmethylene-6-methylenepiperazines bearing benzene (**1c** and **1f**–**o**), furan (**1d**), thiophene (**1e**), naphthalene (**1p**), and pyrene (**1q**) groups. However, the coupling between 2-isopropylbenzaldehyde (**1r**) and **4** did not occur owing to the steric hindrance of the *ortho*-substituted isopropyl group.

**Scheme 6:**
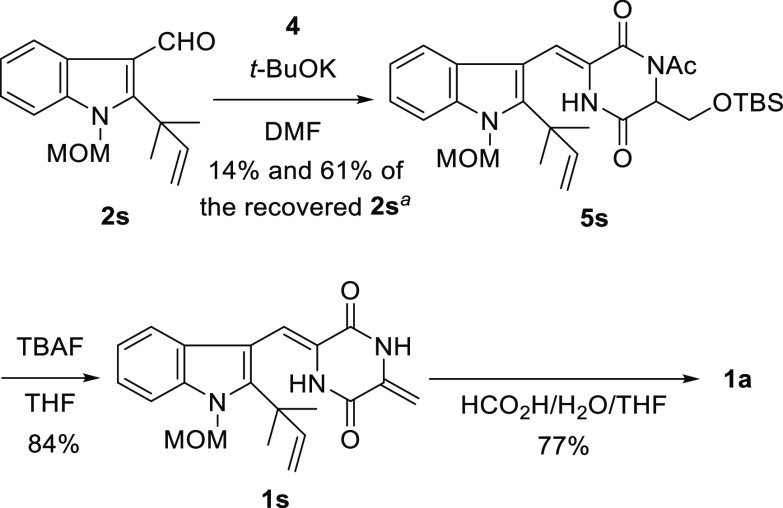
Synthesis of **1a** Reaction was carried out totally four times.

### Synthesis of **1a** and Variecolorin H

**Scheme 7:**
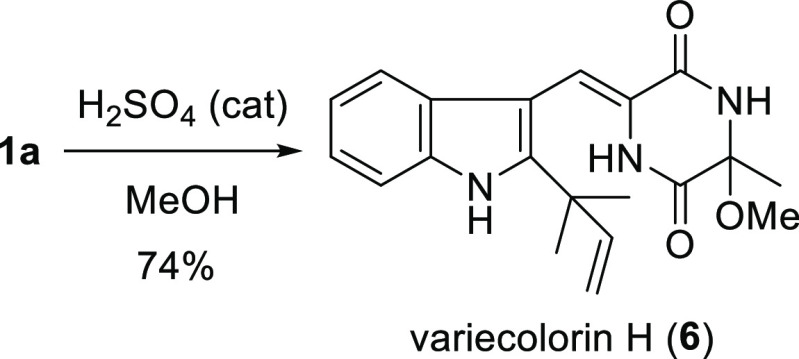
Synthesis of Variecolorin H (**6**)

This methodology was next applied to the synthesis of **1a** (Scheme 6). Compound **2s**,^2,12^ which possesses a methoxymethyl (MOM) group, was used as the substrate. Unfortunately, coupling between **2s** and **4** afforded only a small amount of the desired product **5s**, and considerable amounts of **2s** and **4** were recovered. The reaction was repeated three times to obtain **5s** in a total yield of 14%, while **2s** was recovered in 61% yield. The reaction was also screened using different bases and solvents and at different temperatures, but the yield of **2s** did not improve. These results indicated that this coupling reaction was sensitive to steric hindrance. The treatment of **5s** with TBAF generated **1s** in 84% yield. Finally, the removal of the MOM group from **1s** under weak acidic conditions^12^ provided **1a** in 77% yield. The spectroscopic data of **1a** synthesized in this study were identical to those of the natural product **1a**.^2^ During the purification of **1a** by silica gel chromatography using CHCl_3_ and MeOH as eluents, **1a** was frequently transformed into variecolorin H (**6**),^13^ a related alkaloid isolated from a halotolerant strain of *Aspergillus variecolor*. The treatment of **1a** with a catalytic amount of H_2_SO_4_ in MeOH afforded **6** in 74% yield (Scheme 7). The specific rotation of **6** was reported to be [α]^25^_D_ 0 (*c* 0.3, MeOH),^13^ suggesting that it is a racemate. These results suggested that **6** could be an artifact generated during isolation.

### Inhibitory Effect on HCV Production

**Figure 1:**
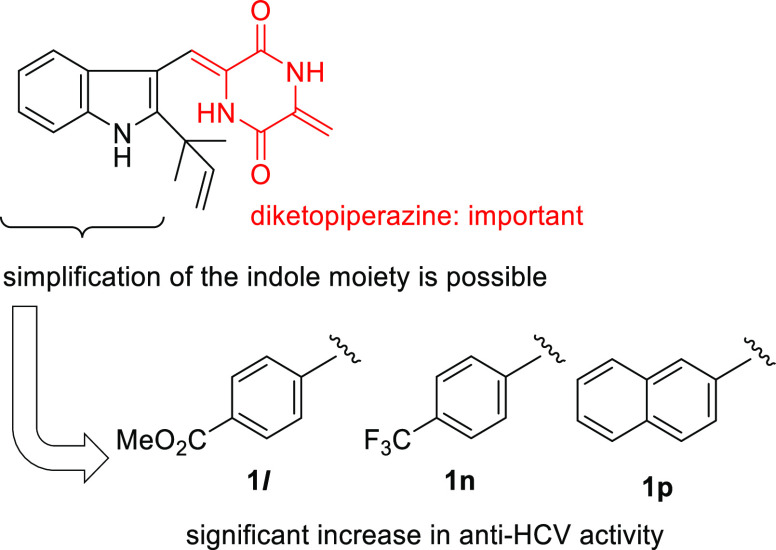
Structure–activity relationships of **1a** and its derivatives as inhibitors of HCV production.

The cytotoxicity and anti-HCV activity of **1a**–**q**, **6**, neoechinulin A (**7**),^14^ and preechinulin (**8**)^15^ were evaluated. Neoechinulin A (**7**) has a single bond between C-12 and C-20, whereas preechinulin (**8**) has two single bonds between C-8 and C-9 and between C-12 and C-20. The 50% and 90% virus inhibitory concentrations (IC_50_ and IC_90_) and 50% cytotoxic concentration (CC_50_) of **1a**–**q** and **6**–**8** are summarized in Table 1. The CC_50_ values of **1a**–**q** and **6**–**8** were greater than 20 μM, indicating negligible cytotoxic activity against the host Huh7.5.1 cells at concentrations below 20 μM (Figure S1 in the Supporting Information). The anti-HCV activity of **1b**–**q** and **6**–**8** were evaluated using **1a** as a positive control (Figure S2 in the Supporting Information).^3^ Interestingly, compounds **1a**–**q** showed antiviral activity irrespective of the structure of the aromatic moiety (Table 1). In contrast, compounds **6**–**8** did not show antiviral activity. These results clearly indicate that the exomethylene moiety in **1a** is important for the anti-HCV activity. It is possible that compounds **1a**–**q** act as electrophilic Michael acceptors with cellular nucleophiles such as proteins and DNA bases. However, the lack of cytotoxicity of **1a**–**q** can rule out the possibility that they nonspecifically bind to these biomolecules via Michael addition. Only compounds **1l**, **1n**, and **1p** reduced the relative virus production by more than 90%, with IC_90_ values of 6.5, 9.7, and 1.9 μM, respectively (Figure 1). The other compounds did not suppress the virus production by up to 90% at concentrations below 20 μM. Compounds **1l** and **1n** contain electron-withdrawing methoxycarbonyl and trifluoromethyl groups, respectively, on the benzene ring, suggesting that the electron density on the benzene ring could influence the anti-HCV activity.
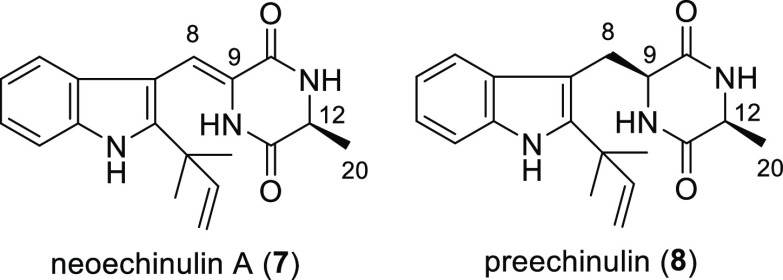


**Table 1:** Anti-HCV Activity (IC_50_ and IC_90_, μM) and Cytotoxicity (CC_50,_ μM) Data for Compounds **1a**–**q**^a^

compound	IC_50_ (μM)^b^	IC_90_ (μM)^b^	CC_50_ (μM)^c^
**1a**	4.7 ± 1.4	>20	>20
**1b**	2.2 ± 0.64	>20	>20
**1c**	0.0059 ± 0.0042	>20	>20
**1d**	1.4 ± 0.34	>20	>20
**1e**	1.4 ± 0.48	>20	>20
**1f**	0.015 ± 0.019	>20	>20
**1g**	1.1 ± 0.34	>20	>20
**1h**	2.7 ± 1.4	>20	>20
**1i**	1.3 ± 0.42	>20	>20
**1j**	2.0 ± 0.068	>20	>20
**1k**	1.1 ± 0.60	>20	>20
**1***l*	1.2 ± 0.16	6.5 ± 1.84	>20
**1m**	1.2 ± 0.54	>20	>20
**1n**	1.6 ± 0.32	9.7 ± 1.06	>20
**1o**	4.9 ± 0.66	>20	>20
**1p**	0.26 ± 0.11	1.9 ± 0.65	>20
**1q**	3.9 ± 0.45	>20	>20
**6**	>20	>20	>20
**7**	>20	>20	>20
**8**	>20	>20	>20

a All experiments were performed in triplicate and means ± standard deviations (SD) are reported.

b IC_50_ and IC_90_ values were determined according to the procedure described previously.^3^

c CC_50_ values were determined by the MTT assay.^16^

### Effect on LXR-Mediated Transcriptional Activity

**Figure 2:**
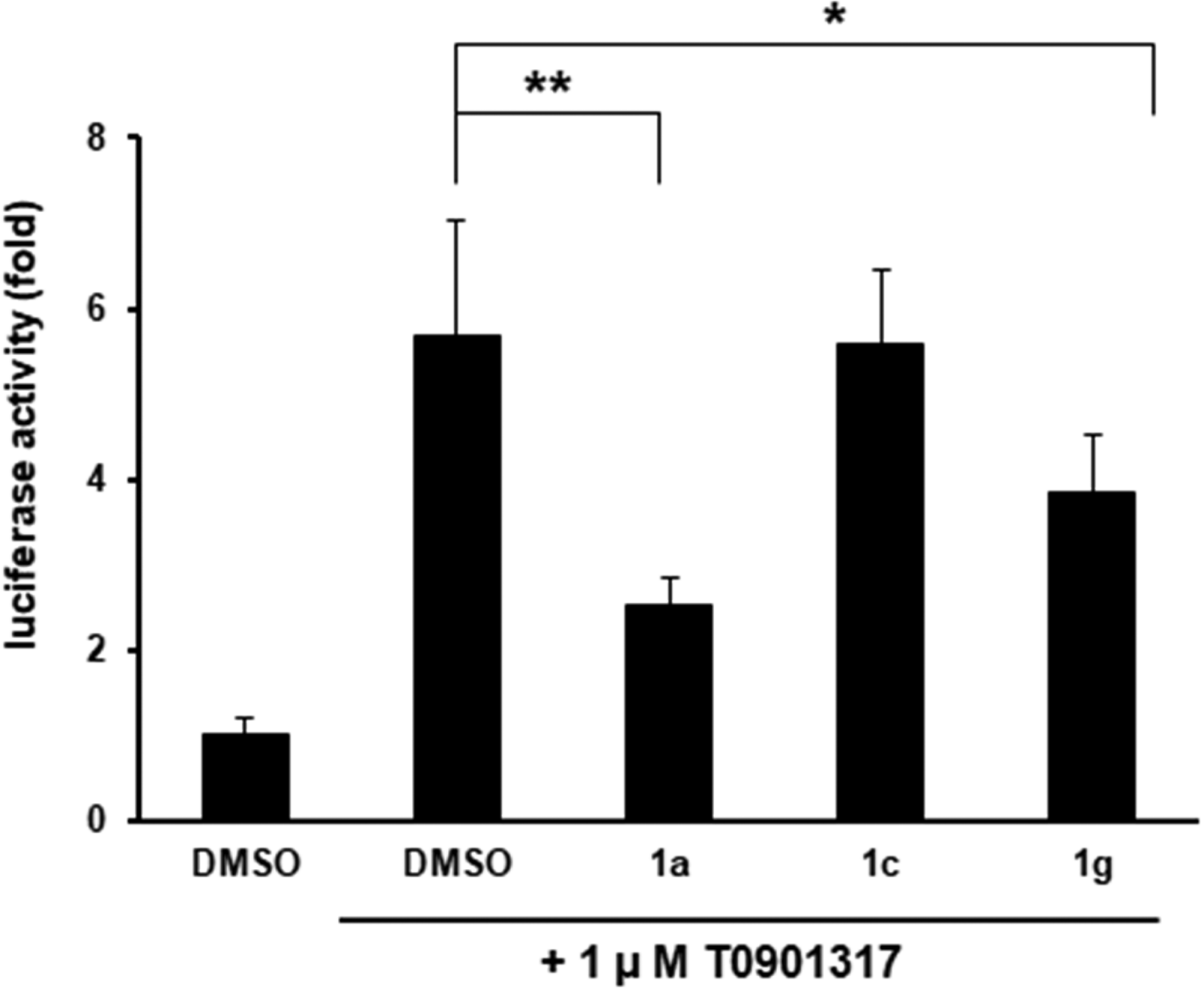
Effects of **1a**, **1c**, and **1g** on LXR-mediated transcriptional activity. Huh-7 cells, which were transfected with a reporter plasmid carrying the binding element of LXR upstream of the firefly luciferase, were treated with or without **1a** (10 μM), **1c** (30 μM), and **1g** (5 μM) in the presence of T0901317. Relative luciferase activities are shown. The values represent means ± SD. The statistical significance was assessed by Tukey’s honestly significant difference (HSD) test, and the asterisks ** and * indicate *p* < 0.01 and *p* < 0.05, respectively.

The effect of **1c** and **1g** on LXR-mediated transcriptional activity was examined using **1a** as a positive control. We performed the reporter gene assay using an LXR element (LXRE)-driven luciferase plasmid to investigate whether they inhibit the transactivation of LXRs.^3^ T0901317,^17^ which is an agonist of LXRs, increased the LXRE-driven luciferase activity (Figure 2). Compounds **1a** and **1g** reduced the T0901317-induced reporter activity mediated by LXRs. Thus, **1a** and **1g** showed anti-HCV activity due to the inhibition of LXR-regulated gene induction, which is required for DMV formation. However, to our surprise, compound **1c** did not show LXR antagonistic activity even at 30 μM, although it showed anti-HCV activity. This suggests that **1c** exhibited antiviral activity via different mechanisms. We have previously reported that **1a** interacts with recombinant LXRα and LXRβ, based on the results of surface plasmon resonance (SPR) analysis.^3^ The association of **1a** with LXRs and dissociation of **1a** from LXRs were observed in the SPR curves, suggesting that neoechinulin B and its derivatives interact noncovalently with LXRs. Although this interaction does not completely exclude the possibility of the reaction of the exomethylene moiety in **1a** with the thiol or amine moiety in LXRs, the lack of the LXR antagonistic activity of **1c** suggests that the diketopiperazine moiety in **1c** does not bind to LXRs and the overall structure of **1a** is important for the specific binding with LXRs. This result also rules out the possibility that the exomethylene moiety on the diketopiperazine ring in **1a** nonspecifically binds to LXRs via Michael addition.

### Inhibitory Effect on SARS-CoV-2 Production

**Figure 3:**
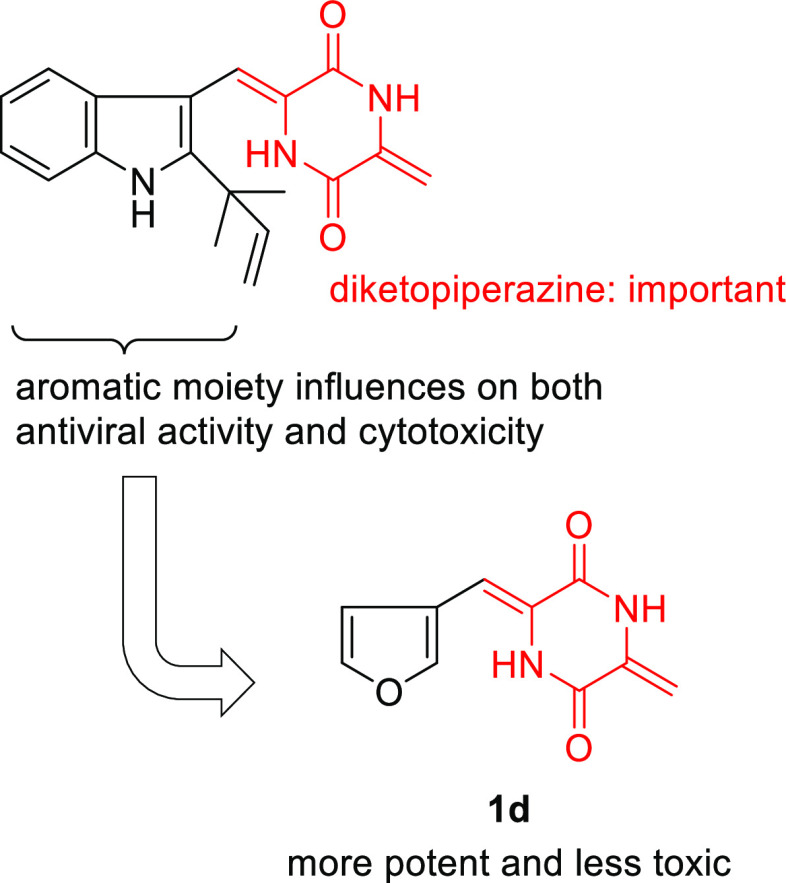
Structure–activity relationships of **1a** and its derivatives as inhibitors of SARS-CoV-2 production.

The cytotoxicity and anti-SARS-CoV-2 activity of **1a**–**q** and **6**–**8** were evaluated by employing a cell-based SARS-CoV-2 infection system using VeroE6 cells expressing the transmembrane serine protease TMPRSS2 (VeroE6/TMPRSS2 cells; Figures S3 and S4 in the Supporting Information).^18,19^ The IC_50_, IC_90_, and CC_50_ values of the compounds are summarized in Table 2. Compound **1a** reduced the production of SARS-CoV-2 RNA with IC_50_ and IC_90_ values of 32.9 and 45.6 μM, respectively, without exhibiting any remarkable cytotoxicity at these concentrations. Compounds **1b**, **1f**, **1g**, **1k**, **1p**, **1q**, and **6**–**8** did not show anti-SARS-CoV-2 activity. The lack of antiviral activity of **6**–**8** indicates that the exomethylene moiety in **1a** is important for the anti-SARS-CoV-2 activity. Because compounds **1e**, **1i**, **1m**, and **1n** showed toxicity toward the host cells, the inhibition of virus production by these compounds could not be evaluated accurately. In contrast, compounds **1c**, **1d**, **1h**, **1j**, **1l**, and **1o** exhibited anti-SARS-CoV-2 activity. The antiviral activity of **1c**, **1d**, **1h**, **1j**, and **1l** was more potent than that of **1a**. Particularly, **1d** was more potent and less toxic than **1a** (Figure 3). It can thus be concluded that the combination of the diketopiperazine core and the aromatic moiety is important for the anti-SARS-CoV-2 activity. No distinct relationships between the structural features and anti-SARS-CoV-2 activity of **1a**, **1c**, **1d**, **1h**, **1j**, **1l**, and **1o** was identified, suggesting that these compounds acted through different or multiple mechanisms.

**Table 2:** Anti-SARS-CoV-2 Activity (IC_50_ and IC_90_, μM) and Cytotoxicity (CC_50_, μM) Data for Compounds **1a**–**q**^a^

compound	IC_50_ (μM)^b^	IC_90_ (μM)^b^	CC_50_ (μM)^c^
**1a**	32.9 ± 13.2	45.6 ± 7.4	>70
**1b**	>40	>40	>100
**1c**	13.6 ± 3.2	20.3 ± 3.2	40.1 ± 10.5
**1d**	9.3 ± 6.2	21.2 ± 9.0	>80
**1e**	–d	–d	42.2 ± 7.9
**1f**	–e	>40	>100
**1g**	–e	>40	>70
**1h**	6.0 ± 2.4	16.3 ± 6.2	63.1 ± 17.9
**1i**	–d	–d	47.2 ± 0.6
**1j**	10.3 ± 3.7	20.1 ± 8.5	62.8 ± 17.4
**1k**	–e	>40	78.8 ± 4.5
**1l**	8.4 ± 2.1	14.1 ± 1.8	>50
**1m**	–d	–d	28.3 ± 6.2
**1n**	9.1 ± 0.7	–d	30.1 ± 7.3
**1o**	20.8 ± 8.0	>40	>100
**1p**	>40	>40	40.7 ± 20.1
**1q**	>40	>40	>100
**6**	>40	>40	>100
**7**	>40	>40	>100
**8**	>40	>40	>100

a Values shown are the means ± SD of triplicate measurements.

b IC_50_ and IC_90_ values were determined according to a previously reported procedure.^17^

c CC_50_ values were determined by the quantification of the survival cell numbers after fixation with 4% paraformaldehyde and staining with 0.02% DAPI.^20^

d IC_50_ and IC_90_ values were not determined because they were much higher than CC_50_.

e The IC_50_ value could not be determined because of poor dose–response curve fitting.

## Conclusion

The diketopiperazine scaffold of **1a** was successfully constructed in this study. Seventeen 3-arylmethylene-6-methylenepiperazine-2,5-diones (**1a**–**q**) were synthesized by the coupling of aldehydes **2** and 1,4-diacetyl-3-{[(*tert*-butyldimethylsilyl)oxy]methyl}piperazine-2,5-dione (**4**), followed by the TBAF treatment of the coupling products **5**. Although **1a** was successfully synthesized, the yield of the coupling reaction between **2a** and **4** needed improvements. Compound **6** was formed during the purification of **1a** by silica gel chromatography using CHCl_3_ and MeOH as eluents, as well as upon the acid treatment of **1a** in MeOH, suggesting that **6** could be an artifact formed during extraction or purification.

The antiviral activities of **1a**–**q** and **6**–**8** against HCV and SARS-CoV-2 were evaluated. Compound **1a** showed antiviral activities against HCV and SARS-CoV-2. In contrast, the structurally related compounds **6**, **7**, and **8** did not show any antiviral activities. These results indicate that the exomethylene moiety in **1a** is important for the antiviral activities against both HCV and SARS-CoV-2. Neoechinulin B derivatives **1b**–**q**, which contain the common diketopiperazine scaffold, showed anti-HCV activity. Among the neoechinulin B derivatives tested in this study, compounds **1l**, **1n**, and **1p** showed more potent anti-HCV activity than **1a** without exhibiting any serious cytotoxicity. Furthermore, **1c**, **1d**, **1h**, **1j**, **1l**, and **1o** exhibited anti-SARS-CoV-2 activity. Particularly, **1c**, **1d**, **1h**, **1j**, and **1l** exhibited more potent anti-SARS-CoV-2 activity than **1a**. The aromatic moieties in **1a**–**q** significantly influence the anti-SARS-CoV-2 activity and cytotoxicity against host cells. However, no clear relationships between the structural features and anti-SARS-CoV-2 activity of **1a**, **1c**, **1d**, **1h**, **1j**, **1l**, and **1o** could be identified, suggesting that these compounds impair the replication of SARS-CoV-2 by different or multiple mechanisms. Although further attempts to improve the antiviral potency and selectivity against HCV and SARS-CoV-2 are necessary, the results of this study clearly show that natural product **1a** is one of the promising lead compounds for the development of broad-spectrum antiviral drugs. The antiviral compounds discussed herein can inhibit the production of HCV and SARS-CoV-2 by targeting LXRs. However, compound **1c**, which showed anti-HCV activity, did not show LXR antagonistic activity, suggesting that this compound showed anti-HCV activity via different mechanisms. Further structural optimization to improve the antiviral efficiency and additional biological studies to elucidate the mechanisms of action are underway and will be reported in due course.

## Experimental Section

### General Experimental Procedures

All reactions sensitive to air or moisture were carried out under an argon atmosphere under anhydrous conditions, unless otherwise noted. Solvents and reagents were used without further purification unless otherwise noted. Analytical TLC was performed using silica gel 60 F_254_ plates (0.25 mm, normal phase, Merck). Normal phase flash column chromatography was performed using silica gel 60 (particle size 40–63 μm; 230–400 mesh ASTM; SilicaFlash F60, SiliCycle Inc.). Melting point (mp) data were determined using a Shimadzu MM-2 instrument and were uncorrected. IR spectra were recorded on a Horiba FT-720 spectrometer, using KBr pellets (solid). ^1^H and proton-decoupled ^13^C (^13^C{^1^H}) NMR spectra were recorded on a Bruker Avance 400 spectrometer (400 and 100 MHz, respectively), using chloroform-*d* (CDCl_3_) and dimethyl sulfoxide-*d*_6_ (DMSO-*d*_6_) as solvents. Chemical shift values are expressed in δ (ppm) relatively to the residual solvent resonance (CDCl_3_, δ 7.26 for ^1^H NMR and δ 77.0 for ^13^C NMR; DMSO-*d*_6_, δ 2.49 for ^1^H NMR and δ 39.7 for ^13^C NMR). Data are reported as follows: chemical shift, multiplicity (s = singlet, d = doublet, t = triplet, q = quartet, br = broad, dd = double doublet, m = multiplet), coupling constants (*J*; Hz), and integration. Mass spectra were obtained by a Sciex X500R quadrupole time-of-flight (QTOF) high-resolution mass spectrometer using electrospray ionization (ESI). Neoechinulin A (**7**) and preechinulin (**8**), which were prepared in our previous study,^21^ were used in this study. Huh7.5.1 cells were obtained from Dr. Francis Chisari at the Scripps Research Institute.^22^ VeroE6/TMPRSS2 cells was obtained from Dr. Makoto Takeda at National Institute of Infectious Diseases.^16^ HCV JFH-1 strain was obtained from Dr. Takaji Wakita at National Institute of Infectious Diseases. SARS-CoV-2 Wk-521 strain was obtained from Dr. Shutoku Matsuyama at National Institute of Infectious Diseases. LXRE-driven luciferase plasmid was obtained from Dr. Maiko Okada at Tokyo University of Technology.

### General Procedure of Coupling of Aldehydes **2** with Diketopiperazine **4** (General Procedure A; Scheme 5)

A solution of **2** (0.20 mmol, 1.0 equiv) and **4**(8) (0.40 mmol, 2.0 equiv) in DMF (2.0 mL) was stirred at 0 °C for 15 min. *t*-BuOK (0.58 mmol, 2.9 equiv) was added to the mixture, and the resultant mixture was stirred at rt until no further TLC changes were observed. The reaction was quenched by the addition of a saturated aqueous NH_4_Cl solution. The mixture was diluted with EtOAc. After the layers were separated, the organic layer was washed with H_2_O. The aqueous layer was extracted with EtOAc three times. The combined organic layer was washed with brine, dried over Na_2_SO_4_, and concentrated to give a residue. The residue was purified by silica gel column chromatography. For the specific procedures and spectroscopic data for the prepared compounds, see the Supporting Information.

### General Procedure of Transformation of the Intermediates **5** into Methylenepiperazine-2,5-diones **1** (General Procedure B; Scheme 5)

A 1.0 M solution of TBAF (2.0 equiv) in THF was added to a solution of **5** (1.0 equiv) in THF. The mixture was stirred at rt until no further TLC changes were observed. The reaction was quenched by the addition of a saturated aqueous NH_4_Cl solution. The mixture was diluted with EtOAc. After the layers were separated, the organic layer was washed with H_2_O. The aqueous layer was extracted with EtOAc. The combined organic layer was washed with brine, dried over Na_2_SO_4_, and concentrated to give a residue. The residue was purified by trituration with MeOH. For the specific procedures and spectroscopic data for the prepared compounds, see the Supporting Information.

### (*Z*)-3-[(1*H*-Indol-3-yl)methylene]-6-methylenepiperazine-2,5-dione (**1b**)

Hydrazine monohydrate (14.0 μL, 0.44 mmol) was added to a solution of **1b′** (43.3 mg, 0.15 mmol) in DMF (1.5 mL) at 0 °C. The mixture was stirred at rt for 2 h. The mixture was concentrated to a crude residue. The residue was purified by trituration with MeOH to give **1b** (14.9 mg, 40%) as a yellow solid. Mp = 285–286 °C; IR (KBr) ν_max_ = 3330, 3257, 3188, 3051, 1682, 1637, 1601, 1529 cm^–1^; ^1^H NMR (400 MHz, DMSO-*d*_6_) δ 11.74 (s, 1H), 10.82 (s, 1H), 9.66 (s, 1H), 8.07 (s, 1H), 7.64 (d, *J* = 7.7 Hz, 1H), 7.43 (d, *J* = 7.7 Hz, 1H), 7.18 (t, *J* = 7.7 Hz, 1H), 7.11 (t, *J* = 7.7 Hz, 1H), 7.07 (s, 1H), 5.25 (s, 1H), 4.89 (s, 1H); ^13^C{^1^H} NMR (100 MHz, DMSO-*d*_6_) δ 157.8, 156.9, 135.9, 135.1, 127.3, 127.1, 122.4, 122.2, 120.3, 118.2, 112.1, 108.6, 107.9, 99.5; HRMS (ESI/QTOF) *m*/*z* [M + Na]^+^ calcd for C_14_H_11_N_3_NaO_2_ 276.0744; found 276.0749.

### Synthesis of **1b** by Coupling between **2b′** and **3** (Scheme 4)

A solution of **2b′** (29.0 mg, 0.20 mmol) and **3** (50.4 mg, 0.40 mmol) in piperidine (2.0 mL) was stirred at 110 °C for 8 h. The mixture was concentrated. The residue was purified by silica gel column chromatography (CHCl_3_/acetone, 15:1) and trituration with MeOH to give **2b′** (2.2 mg, 4%) with recovered **2b′** (12.1 mg, 40%).

### Neoechinulin B (**1a**)

A solution of **1s** (6.5 mg, 0.18 mmol) in H_2_O/THF/HCO_2_H, 1:1:2 (1.0 mL), was stirred at rt for 3.5 h. The reaction was quenched by the addition of a saturated aqueous NaHCO_3_ solution. The mixture was diluted with EtOAc. After the layers were separated, the organic layer was washed with H_2_O. The aqueous layer was extracted with EtOAc. The combined organic layer was washed with brine, dried over Na_2_SO_4_, and concentrated to give a residue. The residue was purified by silica gel column chromatography (hexane/EtOAc, 2:1) to give **1a** (4.4 mg, 77%) as a yellow solid. The spectroscopic data of synthetic **1a** were identical with those reported for natural **1a**.^2^

### Variecolorin H (**6**)

A catalytic amount (3 drops) of concentrated H_2_SO_4_ was added to a solution of **1a** (3.2 mg, 0.01 mmol) in MeOH (1.5 mL). The mixture was stirred at rt for 3 h. The reaction was quenched by the addition of a saturated aqueous NaHCO_3_ solution. The mixture was diluted with EtOAc. After the layers were separated, the organic layer was washed with H_2_O. The aqueous layer was extracted with EtOAc. The combined organic layer was washed with brine, dried over Na_2_SO_4_, and concentrated to give a residue. The residue was purified by silica gel column chromatography (hexane/EtOAc, 3:1) to give **6** (2.6 mg, 74%) as a yellow solid. Mp = 234–235 °C; (KBr) ν_max_ = 3336, 3087, 3066, 2968, 2931, 1684, 1628 cm^–1^; ^1^H NMR (400 MHz, CDCl_3_) δ 8.36 (s, 1H), 7.59 (s, 1H), 7.37 (d, *J* = 7.6 Hz, 1H), 7.30 (s, 1H), 7.24–7.15 (m, 3H), 6.43 (s, 1H), 6.07 (dd, *J* = 17.6, 11.4 Hz, 1H), 5.23 (d, *J* = 11.4 Hz, 1H), 5.19 (d, *J* = 17.6 Hz, 1H), 3.36 (s, 3H), 1.72 (s, 3H), 1.53 (s, 6H); ^1^H NMR (400 MHz, DMSO-*d*_6_) δ 11.09 (s, 1H), 9.24 (s, 1H), 9.08 (s, 1H), 7.40 (d, *J* = 7.7 Hz, 1H), 7.16 (d, *J* = 7.7 Hz, 1H), 7.07 (t, *J* = 7.7 Hz, 1H), 7.01–6.98 (m, 2H), 6.06 (dd, *J* = 17.2, 10.6 Hz, 1H), 5.04 (d, *J* = 10.4 Hz, 1H), 5.02 (d, *J* = 17.2 Hz, 1H), 3.21 (s, 3H), 1.47 (s, 3H), 1.46 (s, 3H), 1.44 (s, 1H); ^13^C{^1^H} NMR (100 MHz, CDCl_3_) δ 163.0, 160.0, 144.2, 144.1, 134.3, 125.9, 124.0, 122.5, 121.3, 118.7, 113.6, 113.5, 111.4, 102.8, 85.2, 51.4, 39.2, 27.4, 27.3, 25.9; ^13^C{^1^H} NMR (100 MHz, DMSO-*d*_6_) δ 163.0, 160.0, 144.2, 144.1, 134.3, 125.9, 124.0, 122.5, 121.3, 118.7, 113.6, 113.5, 111.4, 102.8, 85.0, 51.4, 39.2, 27.4, 27.3, 25.9; HRMS (ESI/QTOF) *m*/*z* [M + Na]^+^ calcd for C_20_H_23_N_3_NaO_3_ 376.1632; found 376.1634.

### Cell Culture

Huh7.5.1 and Huh-7 cells were cultured in Dulbecco’s modified Eagle’s medium (DMEM) supplemented with 10% fetal bovine serum (FBS), 10 units/mL penicillin, 10 μg/mL streptomycin, 0.1 mM nonessential amino acids, 1 mM sodium pyruvate, and 10 mM HEPES (pH 7.4) at 37 °C in 5% CO_2_. VeroE6/TMPRSS2 cells were cultured in DMEM supplemented with 10% FBS, 100 units/mL penicillin, 100 μg/mL streptomycin, 10 mM HEPES (pH 7.4), and 1 mg/mL G418 at 37 °C in 5% CO_2_. During the infection assay, 10% FBS was replaced by 2% FBS and G418 removed. MRC-5 cells were cultured in minimum essential medium Eagle-alpha modification (α-MEM) without ribonucleosides and deoxyribonucleosides supplemented with 10% FBS at 37 °C in 5% CO_2_.

### Anti-HCV and MTT Assays

The anti-HCV assay was performed as described previously.^3^ In brief, Huh7.5.1 cells were treated with HCV JFH-1 at a multiplicity of infection (MOI) of 0.15 for 4 h. The cells were then washed and cultured with growth medium in the presence of various concentrations of each compound for 72 h. The infectivity of HCV in the medium was quantified by a focus-forming assay with Huh7.5.1 cells. Cell viability at 72 h post-treatment was simultaneously measured by the MTT assay.^16^ Cell growth inhibition was evaluated as the ratio of the absorbance of the sample to that of the control.

### Luciferase Reporter Assay

Huh-7 cells were transfected with a reporter plasmid carrying the binding element for LXR upstream of the firefly luciferase.^3^ The cells were then either left untreated or treated with compounds in the presence or absence of the agonist for 48 h. The cells were lysed, and the luciferase activity was measured.^3^

### Anti-SARS-CoV-2 Assay

SARS-CoV-2 was handled in a biosafety level 3 (BSL3). SARS-CoV-2 Wk-521 strain, a clinical isolate from a patient with coronavirus disease 2019 (COVID-19), was used for the anti-SARS-CoV-2 assay.^18^ Virus infectious titers were measured by inoculating cells with a 10-fold serial dilution of virus and cytopathology measured to calculate 50% tissue culture infective dose (TCID_50_)/mL. For the infection assay, VeroE6/TMPRSS2 cells were inoculated with virus at an MOI of 0.003 for 1 h, and unbound virus was removed by washing. Cells were cultured with growth medium in the presence of various concentrations of each compound for 24 h, and extracellular viral RNA was quantified.^19^

### Quantification of Viral RNA

Viral RNA was extracted with a MagMax viral/pathogen II nucleic acid isolation kit (Thermo Fisher Scientific) and quantified by real-time reverse transcription polymerase chain reaction (RT-PCR) analysis with a one-step RT-qPCR kit (THUNDERBIRD Probe one-step qRT-PCR kit, TOYOBO) using 5′-ACAGGTACGTTAATAGTTAATAGCGT-3′ for forward primer and 5′-ATATTGCAGCAGTACGCACACA-3′ for reverse primer, and a 5′-FAM-ACACTAGCCATCCTTACTGCGCTTCG-TAMRA-3′ probe, as described. Detection limit of SARS-CoV-2 RNA in this study was the 38th cycle as the C_t_ cycle.^19^

### Cell Viability of VeroE6/TMPRSS2 Cells

The number of cells was quantified based on the measurement of the DNA content.^20^ Cell growth inhibition was evaluated as the ratio of the absorbance of the sample to that of the control. VeroE6/TMPRSS2 cells were incubated in the presence of various concentrations of each compound for 24 h. The cells were washed with PBS and fixed in 4% paraformaldehyde. The resultant cells were stained with 0.02% 4′,6-diamidino-2-phenylindole (DAPI) and washed with PBS four times. The number of surviving cells was quantified with a high-content imaging analyzer, ImageXpress Micro Confocal (Molecular Device, San Jose, CA, USA).

## Supporting Information Available

Details for synthetic procedures, spectroscopic data, and biological activities for the prepared compounds; copies of ^1^H and ^13^C{^1^H} NMR spectra of the prepared compounds (PDF)
