# Supplementary material for: Synthesis and Antiviral Activities of Neoechinulin B and Its Derivatives
Source: J Nat Prod. 2021 Dec 30;85(1):284–91. doi: 10.1021/acs.jnatprod.1c01120 (PMC8751641; doi:10.1021/acs.jnatprod.1c01120)

Supporting Information

Synthesis and Antiviral Activities of Neoechinulin B and Its Derivatives

Kota Nishiuchi,[†] Hirofumi Ohashi,[†,‡,⊥] Kazane Nishioka,[†,‡] Masako Yamasaki,[†,‡] Masateru Furuta,[†] Takumi Mashiko,[†] Shusuke Tomoshige,[†,^] Kenji Ohgane,[†,∥] Shinji Kamisuki,[§] Koichi Watashi,[†,‡,⊥] Kouji Kuramochi[†,*]

[†]Department of Applied Biological Science, Tokyo University of Science, 2641 Yamazaki, Noda, Chiba, 278-8510, Japan.

[‡]Department of Virology II, National Institute of Infectious Diseases, 1-23-1 Toyama, Shinjuku-ku,Tokyo 162-8640, Japan.

[§]School of Veterinary Medicine and Center for Human and Animal Symbiosis Science, Azabu University, 1-17-71 Fuchinobe, Chuo-ku, Sagamihara, Kanagawa, 252-5201, Japan.

[⊥]Research Center for Drug and Vaccine Development, National Institute of Infectious Diseases, 1-23-1 Toyama, Shinjuku-ku, Tokyo, 162-8640, Japan

[^]Present Address: Graduate School of Life Sciences, Tohoku University, 2-1-1 Katahira, Aoba-ku, Sendai 980-8577, Japan.

[∥]Present Address: Department of Chemistry, Ochanomizu University, 2-1-1 Otsuka, Bunkyo-ku, Tokyo 112-8610, Japan.

Index

Details for the synthetic procedure and spectroscopic data

Procedure of coupling of aldehydes 2 with diketopiperazine 4. S4

Procedure of transformation of the intermediates 5 into methylenepiperazine-2,5-diones 1. S8

Details for the biological activities

Figure S1. Cytotoxicity of **1a**–**q** against Huh7.5.1 cells. S13

Figure S2. Anti-HCV activity of **1a**–**q** and **6**–**7**. S14

Figure S3. Cytotoxicity of **1a**–**q** and **6**–**7** against VeroE6/TMPRSS2 cells. S16

Figure S4. Anti-SARS-CoV-2 activity of **1a**,**1c**, **1d**, **1h**, **1j**, **1*l***, **1n**, and **1o**. S18

^{1}H and ^{13}C NMR spectroscopic data

Figure S5. ^{1}H NMR spectrum (400 MHz, $CDCl_3$) of **5b**. S19

Figure S6. $^{13}C\{^{1}H\}$ NMR spectrum (100 MHz, $CDCl_3$) of **5b**. S20

Figure S7. ^{1}H NMR spectrum (400 MHz, $CDCl_3$) of **5c**. S21
Figure S8. $^{13}C\{^{1}H\}$ NMR spectrum (100 MHz, $CDCl_3$) of **5c**. S22
Figure S9. ^{1}H NMR spectrum (400 MHz, $CDCl_3$) of **5d**. S23
Figure S10. $^{13}C\{^{1}H\}$ NMR spectrum (100 MHz, $CDCl_3$) of **5d**. S24
Figure S11. ^{1}H NMR spectrum (400 MHz, $CDCl_3$) of **5e**. S25
Figure S12. $^{13}C\{^{1}H\}$ NMR spectrum (100 MHz, $CDCl_3$) of **5e**. S26
Figure S13. ^{1}H NMR spectrum (400 MHz, $CDCl_3$) of **5f**. S27
Figure S14. $^{13}C\{^{1}H\}$ NMR spectrum (100 MHz, $CDCl_3$) of **5f**. S28
Figure S15. ^{1}H NMR spectrum (400 MHz, $CDCl_3$) of **5g**. S29
Figure S16. $^{13}C\{^{1}H\}$ NMR spectrum (100 MHz, $CDCl_3$) of **5g**. S30
Figure S17. ^{1}H NMR spectrum (400 MHz, $CDCl_3$) of **5h**. S31
Figure S18. $^{13}C\{^{1}H\}$ NMR spectrum (100 MHz, $CDCl_3$) of **5h**. S32
Figure S19. ^{1}H NMR spectrum (400 MHz, $CDCl_3$) of **5i**. S33
Figure S20. $^{13}C\{^{1}H\}$ NMR spectrum (100 MHz, $CDCl_3$) of **5i**. S34
Figure S21. ^{1}H NMR spectrum (400 MHz, $CDCl_3$) of **5j**. S35
Figure S22. $^{13}C\{^{1}H\}$ NMR spectrum (100 MHz, $CDCl_3$) of **5j**. S36
Figure S23. ^{1}H NMR spectrum (400 MHz, $CDCl_3$) of **5k**. S37
Figure S24. $^{13}C\{^{1}H\}$ NMR spectrum (100 MHz, $CDCl_3$) of **5k**. S38
Figure S25. ^{1}H NMR spectrum (400 MHz, $CDCl_3$) of **5*l***. S39
Figure S26. $^{13}C\{^{1}H\}$ NMR spectrum (100 MHz, $CDCl_3$) of **5*l***. S40
Figure S27. ^{1}H NMR spectrum (400 MHz, $CDCl_3$) of **5m**. S41
Figure S28. $^{13}C\{^{1}H\}$ NMR spectrum (100 MHz, $CDCl_3$) of **5m**. S42
Figure S29. ^{1}H NMR spectrum (400 MHz, $CDCl_3$) of **5n**. S43
Figure S30. $^{13}C\{^{1}H\}$ NMR spectrum (100 MHz, $CDCl_3$) of **5n**. S44
Figure S31. ^{1}H NMR spectrum (400 MHz, $CDCl_3$) of **5o**. S45
Figure S32. $^{13}C\{^{1}H\}$ NMR spectrum (100 MHz, $CDCl_3$) of **5o**. S46
Figure S33. ^{1}H NMR spectrum (400 MHz, $CDCl_3$) of **5p**. S47
Figure S34. $^{13}C\{^{1}H\}$ NMR spectrum (100 MHz, $CDCl_3$) of **5p**. S48
Figure S35. ^{1}H NMR spectrum (400 MHz, $CDCl_3$) of **5q**. S49
Figure S36. $^{13}C\{^{1}H\}$ NMR spectrum (100 MHz, $CDCl_3$) of **5q**. S50
Figure S37. ^{1}H NMR spectrum (400 MHz, $CDCl_3$) of **5s**. S51
Figure S38. $^{13}C\{^{1}H\}$ NMR spectrum (100 MHz, $CDCl_3$) of **5s**. S52
Figure S39. ^{1}H NMR spectrum (400 MHz, $CDCl_3$) of **1a**. S53
Figure S40. $^{13}C\{^{1}H\}$ NMR spectrum (100 MHz, $CDCl_3$) of **1a**. S54
Figure S41. ^{1}H NMR spectrum (400 MHz, DMSO-d_6) of **1b**. S55
Figure S42. $^{13}C\{^{1}H\}$ NMR spectrum (100 MHz, DMSO-d_6) of **1b**. S56
Figure S43. ^{1}H NMR spectrum (400 MHz, DMSO-d_6) of **1b′**. S57

Figure S44. $^{13}C\{^1H\}$ NMR spectrum (100 MHz, DMSO-d_6) of **1b′**. S58
Figure S45. ^1H NMR spectrum (400 MHz, DMSO-d_6) of **1c**. S59
Figure S46. $^{13}C\{^1H\}$ NMR spectrum (100 MHz, DMSO-d_6) of **1c**. S60
Figure S47. ^1H NMR spectrum (400 MHz, DMSO-d_6) of **1d**. S61
Figure S48. $^{13}C\{^1H\}$ NMR spectrum (100 MHz, DMSO-d_6) of **1d**. S62
Figure S49. ^1H NMR spectrum (400 MHz, DMSO-d_6) of **1e**. S63
Figure S50. $^{13}C\{^1H\}$ NMR spectrum (100 MHz, DMSO-d_6) of **1e**. S64
Figure S51. ^1H NMR spectrum (400 MHz, DMSO-d_6) of **1f**. S65
Figure S52. $^{13}C\{^1H\}$ NMR spectrum (100 MHz, DMSO-d_6) of **1f**. S66
Figure S53. ^1H NMR spectrum (400 MHz, DMSO-d_6) of **1g**. S67
Figure S54. $^{13}C\{^1H\}$ NMR spectrum (100 MHz, DMSO-d_6) of **1g**. S68
Figure S55. ^1H NMR spectrum (400 MHz, DMSO-d_6) of **1h**. S69
Figure S56. $^{13}C\{^1H\}$ NMR spectrum (100 MHz, DMSO-d_6) of **1h**. S70
Figure S57. ^1H NMR spectrum (400 MHz, DMSO-d_6) of **1i**. S71
Figure S58. $^{13}C\{^1H\}$ NMR spectrum (100 MHz, DMSO-d_6) of **1i**. S72
Figure S59. ^1H NMR spectrum (400 MHz, DMSO-d_6) of **1j**. S73
Figure S60. $^{13}C\{^1H\}$ NMR spectrum (100 MHz, DMSO-d_6) of **1j**. S74
Figure S61. ^1H NMR spectrum (400 MHz, DMSO-d_6) of **1k**. S75
Figure S62. $^{13}C\{^1H\}$ NMR spectrum (100 MHz, DMSO-d_6) of **1k**. S76
Figure S63. ^1H NMR spectrum (400 MHz, DMSO-d_6) of **1*l***. S77
Figure S64. $^{13}C\{^1H\}$ NMR spectrum (100 MHz, DMSO-d_6) of **1*l***. S78
Figure S65. ^1H NMR spectrum (400 MHz, DMSO-d_6) of **1m**. S79
Figure S66. $^{13}C\{^1H\}$ NMR spectrum (100 MHz, DMSO-d_6) of **1m**. S80
Figure S67. ^1H NMR spectrum (400 MHz, DMSO-d_6) of **1n**. S81
Figure S68. $^{13}C\{^1H\}$ NMR spectrum (100 MHz, DMSO-d_6) of **1n**. S82
Figure S69. ^1H NMR spectrum (400 MHz, DMSO-d_6) of **1o**. S83
Figure S70. $^{13}C\{^1H\}$ NMR spectrum (100 MHz, DMSO-d_6) of **1o**. S84
Figure S71. ^1H NMR spectrum (400 MHz, DMSO-d_6) of **1p**. S85
Figure S72. $^{13}C\{^1H\}$ NMR spectrum (100 MHz, DMSO-d_6) of **1p**. S86
Figure S73. ^1H NMR spectrum (400 MHz, DMSO-d_6) of **1q**. S87
Figure S74. $^{13}C\{^1H\}$ NMR spectrum (100 MHz, DMSO-d_6) of **1q**. S88
Figure S75. ^1H NMR spectrum (400 MHz, $CDCl_3$) of **1s**. S89
Figure S76. $^{13}C\{^1H\}$ NMR spectrum (100 MHz, $CDCl_3$) of **1s**. S90
Figure S75. ^1H NMR spectrum (400 MHz, $CDCl_3$) of **6**. S91
Figure S78. $^{13}C\{^1H\}$ NMR spectrum (100 MHz, $CDCl_3$) of **6**. S92

Details for the synthetic procedure and spectroscopic data

Procedure of coupling of aldehydes 2 with diketopiperazine 4 (Scheme 5).

(Z)-1-Acetyl-3-{(1-acetyl-1H-indol-3-yl)methylene}-6-{[(tert-butyldimethylsilyl)oxy]methyl}piperazine-2,5-dione (5b). Following the general procedure A, the reaction was performed using **2b** (37.4 mg, 0.20 mmol), **4** (137 mg, 0.40 mmol) and *t*-BuOK (65.3 mg, 0.58 mmol) in DMF (2.0 mL) for 15 h. The residue was purified by silica gel column chromatography (hexane/EtOAc = 2/1) to give **5b** (83.5 mg, 89%) as a yellow solid. Mp = 173–174 °C; IR (KBr) ν_{max} = 3282, 3132, 3080, 3020, 2952, 2931, 2893, 2856, 1711, 1678, 1622, 1552 cm^{-1}; ^{1}H NMR (400 MHz, $CDCl_3$) δ 8.45 (d, *J* = 7.7 Hz, 1H), 8.03 (s, 1H), 7.65–7.63 (m, 2H), 7.46 (t, *J* = 7.7 Hz, 1H), 7.37 (t, *J* = 7.7 Hz, 1H), 7.21 (s, 1H), 5.11 (s, 1H), 4.14 (d, *J* = 10.3 Hz, 1H), 3.94 (dd, *J* = 10.3, 2.6 Hz, 1H), 2.70 (s, 3H), 2.64 (s, 3H), 0.79 (s, 9H), −0.02 (s, 6H); $^{13}C\{^{1}H\}$ NMR (100 MHz, $CDCl_3$) δ 172.4, 168.3, 165.6, 160.7, 135.6, 128.9, 127.5, 126.5, 124.4, 123.9, 119.5, 116.8, 115.1, 108.8, 64.5, 59.3, 26.7, 25.6 (3C), 24.1, 18.2, −5.78, −5.89; HRMS (ESI/QTOF) *m*/*z*: $[M+H]^+$ Calcd for $C_{24}H_{32}N_3O_5Si$ 470.2106; Found 470.2104.

(Z)-1-Acetyl-3-benzylidene-6-{[(tert-butyldimethylsilyl)oxy]methyl}piperazine-2,5-dione (5c). Following the general procedure A, the reaction was performed using **2c** (20.4 mL, 0.20 mmol), **4** (137 mg, 0.40 mmol) and *t*-BuOK (65.3 mg, 0.58 mmol) in DMF (2.0 mL) for 6 h. The residue was purified by silica gel column chromatography (hexane/EtOAc = 3/1) to give **5c** (63.9 mg, 82%) as a white solid. Mp = 104–105 °C; IR (KBr) ν_{max} = 3242, 2956, 2929, 2885, 2856, 1682, 1633 cm^{-1}; ^{1}H NMR (400 MHz, $CDCl_3$) δ 7.95 (s, 1H), 7.47–7.43 (m, 2H), 7.38–7.34 (m, 3H), 7.09 (s, 1H), 5.09 (s, 1H), 4.12 (dd, *J* = 10.4, 1.4 Hz, 1H), 3.92 (dd, *J* = 10.4, 2.8 Hz, 1H), 2.61 (s, 3H), 0.80 (s, 9H), −0.02 (s, 6H); $^{13}C\{^{1}H\}$ NMR (100 MHz, $CDCl_3$) δ 172.4, 165.4, 161.1, 132.8, 129.5 (2C), 129.0, 128.4 (2C), 126.9, 117.8, 64.5, 59.3, 26.6, 25.6 (3C), 18.1, −5.85 (2C); HRMS (ESI/QTOF) *m*/*z*: $[M+H]^+$ Calcd for $C_{20}H_{29}N_2O_4Si$ 389.1891; Found 389.1877.

(Z)-1-Acetyl-6-{[(tert-butyldimethylsilyl)oxy]methyl}-3-(furan-3-ylmethylene)piperazine-2,5-dione (5d). Following the general procedure A, the reaction was performed using **2d** (17.5 µL, 0.20 mmol), **4** (137 mg, 0.40 mmol) and *t*-BuOK (65.3 mg, 0.58 mmol) in DMF (2.0 mL) for 14 h. The residue was purified by silica gel column chromatography (hexane/EtOAc = 3/1) to give **5d** (72.2 mg, 95%) as a white solid. Mp = 158–159 °C; IR (KBr) ν_{max} = 3400, 3255, 3143, 3120, 2956, 2931, 2885, 2858, 1707, 1687, 1635 cm^{-1}; ^{1}H NMR (400 MHz, $CDCl_3$) δ 7.81 (s, 1H), 7.68 (s, 1H), 7.53 (m, 1H), 6.90 (s, 1H), 6.56 (s, 1H), 5.09 (s, 1H), 4.10 (dd, *J* = 10.4, 1.6 Hz, 1H), 3.90 (dd, *J* = 10.4, 2.8 Hz, 1H), 2.59 (s, 3H), 0.76 (s, 9H), −0.05 (s, 6H); $^{13}C\{^{1}H\}$ NMR (100 MHz, $CDCl_3$) δ 172.4, 165.4, 160.9, 144.7, 142.9, 126.3, 118.6, 109.2, 109.1, 64.4, 59.3, 26.6, 25.5 (3C), 18.1, −5.89, −5.90; HRMS (ESI/QTOF) *m*/*z*: $[M+H]^+$ Calcd for $C_{18}H_{27}N_2O_5Si$ 379.1684; Found 379.1677.

(Z)-1-Acetyl-6-{[(tert-butyldimethylsilyl)oxy]methyl}-3-(thiophen-3-ylmethylene)piperazine-2,5-dione (5e). Following the general procedure A, the reaction was performed using **2e** (18.2 µL, 0.20 mmol), **4** (137 mg, 0.40 mmol) and *t*-BuOK (65.3 mg, 0.58 mmol) in DMF (2.0 mL) for 14 h. The residue was purified by silica gel column chromatography (hexane/EtOAc = 3/1) to give **5e** (36.7 mg, 47%) as a white solid. Mp = 84–85 °C; IR (KBr) ν_{max} = 3176, 3095, 3030, 2951, 2929, 2858, 2341, 1689, 1631 cm^{-1}; ^{1}H NMR (400 MHz, $CDCl_3$) δ 7.92 (s, 1H), 7.47–7.45 (m, 1H), 7.42 (m, 1H), 7.17 (dd, *J* = 5.0, 1.1 Hz, 1H), 7.05 (s, 1H), 5.09 (s, 1H), 4.11 (dd, *J* = 10.4, 1.6 Hz, 1H), 3.91 (dd, *J* = 10.4, 2.8 Hz, 1H), 2.60 (s, 3H), 0.77 (s, 9H), −0.04 (s, 6H); $^{13}C\{^{1}H\}$ NMR (100 MHz, $CDCl_3$)

δ 172.4, 165.4, 161.1 133.7, 127.6, 127.2, 126.1, 125.7, 112.7, 64.4, 59.3, 26.6, 25.6 (3C), 18.1, −5.87, −5.88; HRMS (ESI/QTOF) *m*/*z*: $[M+H]^+$ Calcd for $C_{18}H_{27}N_2O_4SSi$ 395.1455; Found 395.1443.

(Z)-1-Acetyl-6-{[(tert-butyldimethylsilyl)oxy]methyl}-3-(3,5-di-tert-butylbenzylidene)piperazine-2,5-dione (5f). Following the general procedure A, the reaction was performed using **2f** (43.7 mg, 0.20 mmol), **4** (137 mg, 0.40 mmol) and *t*-BuOK (65.3 mg, 0.58 mmol) in DMF (2.0 mL) for 13 h. The residue was purified by silica gel column chromatography (hexane/EtOAc = 4/1) to give **5f** (86.6 mg, 84%) as a white solid. Mp = 57–58 °C; IR (KBr) ν_{max} = 3388, 3070, 2958, 2931, 2860, 1707, 1637, 1593, 1471 cm^{-1}; ^1H NMR (400 MHz, $CDCl_3$) δ 7.88 (s, 1H), 7.43 (s, 1H), 7.16 (s, 2H), 7.12 (s, 1H), 5.09 (s, 1H), 4.13 (d, *J* = 10.4 Hz, 1H), 3.92 (dd, *J* = 10.4, 2.6 Hz, 1H), 2.61 (s, 3H), 1.33 (s, 18H), 0.82 (s, 9H), 0.01 (s, 3H), 0.00 (s, 3H); $^{13}C\{^1H\}$ NMR (100 MHz, $CDCl_3$) δ 172.4, 165.3, 161.1, 152.4, 129.8, 128.2 (2C), 126.4 (2C), 118.1, 64.4, 59.3, 34.8 (2C), 31.1 (6C), 26.6, 25.6 (3C), 18.1, −5.84 (2C). One carbon signal is overlapped; HRMS (ESI/QTOF) *m*/*z*: $[M+H]^+$ Calcd for $C_{28}H_{45}N_2O_4Si$ 501.3143; Found 501.3135.

(Z)-1-Acetyl-3-[4-(tert-butyl)benzylidene]-6-{[(tert-butyldimethylsilyl)oxy]methyl}piperazine-2,5-dione (5g). Following the general procedure A, the reaction was performed using **2g** (33.5 μL, 0.20 mmol), **4** (137 mg, 0.40 mmol) and *t*-BuOK (65.3 mg, 0.58 mmol) in DMF (2.0 mL) for 13 h. The residue was purified by silica gel column chromatography (hexane/EtOAc = 4/1) to give **5g** (78.4 mg, 88%) as a white solid. Mp = 163–164 °C; IR (KBr) ν_{max} = 3203, 3087, 2958, 2931, 2879, 2858, 1707, 1691, 1639, 1512, 1460 cm^{-1}; ^1H NMR (400 MHz, $CDCl_3$) δ 7.94 (s, 1H), 7.46 (d, *J* = 8.3 Hz, 2H), 7.29 (d, *J* = 8.3 Hz, 2H), 7.07 (s, 1H), 5.08 (s, 1H), 4.12 (dd, *J* = 10.4, 1.3 Hz, 1H), 3.92 (dd, *J* = 10.4, 2.8 Hz, 1H), 2.61 (s, 3H), 1.34 (s, 9H), 0.80 (s, 9H), −0.02 (s, 6H).; $^{13}C\{^1H\}$ NMR (100 MHz, $CDCl_3$) δ 172.4, 165.3, 161.1, 152.4, 129.9, 128.2 (2C), 126.4 (2C), 118.0, 64.4, 59.3, 34.8, 31.1 (3C), 26.6, 25.6 (3C), 18.1, −5.84 (2C). One carbon signal is overlapped; HRMS (ESI/QTOF) *m*/*z*: $[M+H]^+$ Calcd for $C_{24}H_{37}N_2O_4Si$ 445.2517; Found 445.2511.

(Z)-1-Acetyl-6-(((tert-butyldimethylsilyl)oxy)methyl)-3-(4-methylbenzylidene)piperazine-2,5-dione (5h). Following the general procedure A, the reaction was performed using **2h** (23.6 μL, 0.20 mmol), **4** (137 mg, 0.40 mmol) and *t*-BuOK (65.3 mg, 0.58 mmol) in DMF (2.0 mL) for 13 h. The residue was purified by silica gel column chromatography (hexane/EtOAc = 3/1) to give **5h** (82.1 mg, quant.) as a white solid. Mp = 131–133 °C; IR (KBr) ν_{max} = 3194, 2943, 2927, 2858, 1703, 1682, 1630 cm^{-1}; ^1H NMR (400 MHz, $CDCl_3$) δ 7.92 (s, 1H), 7.27–7.26 (m, 4H), 7.06 (s, 1H), 5.09 (s, 1H), 4.12 (dd, *J* = 10.5, 1.4 Hz, 1H), 3.92 (dd, *J* = 10.5, 2.7 Hz, 1H), 2.61 (s, 3H), 2.39 (s, 3H), 0.80 (s, 9H), −0.02 (s, 6H); $^{13}C\{^1H\}$ NMR (100 MHz, $CDCl_3$) δ 172.4, 165.4, 161.2, 139.3, 130.2 (2C), 129.9, 128.4 (2C), 126.3, 118.1, 64.5, 59.3, 26.6, 25.6 (3C), 21.4, 18.1, −5.85 (2C); HRMS (ESI/QTOF) *m*/*z*: $[M+H]^+$ Calcd for $C_{21}H_{31}N_2O_4Si$ 403.2048; Found 403.2045.

(Z)-1-Acetyl-6-{[(tert-butyldimethylsilyl)oxy]methyl}-3-(4-methoxybenzylidene)piperazine-2,5-dione (5i). Following the general procedure A, the reaction was performed using **2i** (24.3 μL, 0.20 mmol), **4** (137 mg, 0.40 mmol) and *t*-BuOK (65.3 mg, 0.58 mmol) in DMF (2.0 mL) for 13 h. The residue was purified by silica gel column chromatography (hexane/EtOAc = 3/1) to give **5i** (73.8 mg, 88%) as a white solid. Mp = 140–141 °C; IR (KBr) ν_{max} = 3180, 3107, 3072, 3033, 3012, 2954, 2929, 2856, 1682, 1631, 1606, 1514, 1464 cm^{-1}; ^1H NMR (400 MHz, $CDCl_3$) δ 7.93 (s, 1H), 7.32 (d, *J* = 8.6 Hz, 2H), 7.03 (s, 1H), 6.96 (d, *J* = 8.6 Hz, 2H), 5.08 (s, 1H), 4.11 (dd, *J* =

10.4, 1.6 Hz, 1H), 3.91 (dd, J = 10.4, 2.8 Hz, 1H), 3.84 (s, 3H), 2.60 (s, 3H), 0.78 (s, 9H), −0.03 (s, 6H); $^{13}C\{^1H\}$ NMR (100 MHz, $CDCl_3$) δ 172.4, 165.4, 161.3, 160.0, 130.0 (2C), 125.5, 125.1, 118.1, 114.9 (2C), 64.4, 59.3, 55.4, 26.6, 25.6 (3C), 18.1, −5.87 (2C); HRMS (ESI/QTOF) m/z: $[M+H]^+$ Calcd for $C_{21}H_{31}N_2O_5Si$ 419.1997; Found 419.1990.

(Z)-1-Acetyl-6-{[(tert-butyldimethylsilyl)oxy]methyl}-3-(3,4-dimethoxybenzylidene)piperazine-2,5-dione (5j). Following the general procedure A, the reaction was performed using **2j** (33.2 mg, 0.20 mmol), **4** (137 mg, 0.40 mmol) and t-BuOK (65.3 mg, 0.58 mmol) in DMF (2.0 mL) for 15.5 h. The residue was purified by silica gel column chromatography (hexane/EtOAc = 3/1) to give **5j** (73.7 mg, 82%) as a white solid. Mp = 175–176 °C; IR (KBr) ν_{max} = 3217, 2949, 2929, 2875, 1707, 1682, 1631, 1604, 1510 cm^{-1}; ^1H NMR (400 MHz, $CDCl_3$) δ 7.94 (s, 1H), 7.02 (s, 1H), 6.97 (d, J = 8.3 Hz, 1H), 6.92 (d, J = 8.3 Hz, 1H), 6.82 (s, 1H), 5.09 (s, 1H), 4.12 (d, J = 10.4 Hz, 1H), 3.92 (s, 3H), 3.92–3.90 (m, 1H), 3.88 (s, 3H), 2.60 (s, 3H), 0.80 (s, 9H), 0.00 (s, 6H); $^{13}C\{^1H\}$ NMR (100 MHz, $CDCl_3$) δ 172.4, 165.3, 161.2, 149.7, 149.6, 125.8, 125.5, 121.0, 118.1, 111.70, 111.67, 64.5, 59.3, 56.0, 55.9, 26.6, 25.6 (3C), 18.1, −5.83, −5.85; HRMS (ESI/QTOF) m/z: $[M+H]^+$ Calcd for $C_{22}H_{33}N_2O_6Si$ 449.2102; Found 449.2108.

(Z)-1-Acetyl-6-{[(tert-butyldimethylsilyl)oxy]methyl}-3-(3,4,5-trimethoxybenzylidene)piperazine-2,5-dione (5k). Following the general procedure A, the reaction was performed using **2k** (39.2 mg, 0.20 mmol), **4** (137 mg, 0.40 mmol) and t-BuOK (65.3 mg, 0.58 mmol) in DMF (2.0 mL) for 16 h. The residue was purified by silica gel column chromatography (hexane/EtOAc = 3/1) to give **5k** (87.1 mg, 91%) as a white solid. Mp = 47–48 °C; IR (KBr) ν_{max} = 3244, 2960, 2931, 2879, 1705, 1641, 1581, 1510, 1460 cm^{-1}; ^1H NMR (400 MHz, $CDCl_3$) δ 8.10 (s, 1H), 6.99 (s, 1H), 6.51 (s, 2H), 5.06 (s, 1H), 4.10 (d, J = 9.3 Hz, 1H), 3.90 (d, J = 9.3 Hz, 1H), 3.85 (s, 3H), 3.83 (s, 6H), 2.59 (s, 3H), 0.80 (s, 9H), −0.02 (s, 3H), −0.03 (s, 3H); $^{13}C\{^1H\}$ NMR (100 MHz, $CDCl_3$) δ 172.3, 165.2, 161.0, 153.9 (2C), 138.5, 128.2, 126.7, 118.0, 105.4 (2C), 64.5, 60.9, 59.3, 56.2 (2C), 26.6, 25.6 (3C), 18.1, −5.82, −5.87; HRMS (ESI/QTOF) m/z: $[M+H]^+$ Calcd for $C_{23}H_{35}N_2O_7Si$ 479.2208; Found 479.2210.

Methyl (Z)-4-[(4-acetyl-5-{[(tert-butyldimethylsilyl)oxy]methyl}-3,6-dioxopiperazin-2-ylidene)methyl]benzoate (5l). Following the general procedure A, the reaction was performed using **2*l*** (32.8 mg, 0.20 mmol), **4** (137 mg, 0.40 mmol) and t-BuOK (65.3 mg, 0.58 mmol) in DMF (2.0 mL) for 16 h. The residue was purified by silica gel column chromatography (hexane/EtOAc = 3/1) to give **5*l*** (75.4 mg, 84%) as a white solid. Mp = 46–48 °C; IR (KBr) ν_{max} = 3440, 2952, 2931, 2883, 2856, 1707, 1685, 1637, 1608, 1508, 1473 cm^{-1}; ^1H NMR (400 MHz, $CDCl_3$) δ 8.09 (d, J = 8.2 Hz, 2H), 8.05 (s, 1H), 7.42 (d, J = 8.2 Hz, 2H), 7.07 (s, 1H), 5.07 (s, 1H), 4.10 (d, J = 10.4 Hz, 1H), 3.93 (s, 3H), 3.93–3.89 (m, 1H), 2.60 (s, 3H), 0.78 (s, 9H), −0.03 (s, 6H); $^{13}C\{^1H\}$ NMR (100 MHz, $CDCl_3$) δ 172.3, 166.2, 165.5, 160.8, 137.3, 130.5 (2C), 130.2, 128.4 (2C), 128.0, 116.2, 64.4, 59.2, 52.3, 26.6, 25.6 (3C), 18.1, −5.88 (2C); HRMS (ESI/QTOF) m/z: $[M+H]^+$ Calcd for $C_{22}H_{31}N_2O_6Si$ 447.1946; Found 447.1949.

(Z)-1-Acetyl-6-{[(tert-butyldimethylsilyl)oxy]methyl}-3-[4-(dimethylamino)benzylidene]piperazine-2,5-dione (5m). Following the general procedure A, the reaction was performed using **2m** (29.8 mg, 0.20 mmol), **4** (137 mg, 0.40 mmol) and t-BuOK (65.3 mg, 0.58 mmol) in DMF (2.0 mL) for 16 h. The residue was purified by silica gel column chromatography (hexane/EtOAc = 4/1) to give **5m** (35.1 mg, 41%) as a yellow solid. Mp = 183–184 °C;

IR (KBr) ν_{max} = 3184, 3072, 3016, 2952, 2935, 2883, 2856, 2808, 1701, 1676, 1604, 1525 cm^{-1}; ^{1}H NMR (400 MHz, $CDCl_3$) δ 7.90 (s, 1H), 7.29 (d, *J*= 8.7 Hz, 2H), 7.02 (s, 1H), 6.72 (d, *J*= 8.7 Hz, 2H), 5.09 (s, 1H), 4.13 (d, *J* = 10.4 Hz, 1H), 3.92 (dd, *J* = 10.4, 2.6 Hz, 1H), 3.02 (s, 6H), 2.60 (s, 3H), 0.78 (s, 9H), −0.03 (s, 6H); $^{13}C\{^1H\}$ NMR (100 MHz, $CDCl_3$) δ 172.6, 165.3, 161.6, 150.5, 130.1 (2C), 123.4, 120.1, 119.6, 112.3 (2C), 64.4, 59.4, 40.1 (2C), 26.6, 25.6 (3C), 18.1, −5.84 (2C); HRMS (ESI/QTOF) *m*/*z*: $[M+H]^+$ Calcd for $C_{22}H_{34}N_3O_4Si$ 432.2313; Found 432.2328.

(Z)-1-Acetyl-6-(((tert-butyldimethylsilyl)oxy)methyl)-3-(4-(trifluoromethyl)benzylidene)piperazine-2,5-dione (5n). Following the general procedure A, the reaction was performed using **2n** (26.8 μL, 0.20 mmol), **4** (137 mg, 0.40 mmol) and *t*-BuOK (65.3 mg, 0.58 mmol) in DMF (2.0 mL) for 15 h. The residue was purified by silica gel column chromatography (hexane/EtOAc = 4/1) to give **5n** (68.1 mg, 75%) as a white solid. Mp = 163–164 °C; IR (KBr) ν_{max} = 3080, 3020. 2956, 2929, 2897, 1712, 1685, 1633, 1466 cm^{-1}; ^{1}H NMR (400 MHz, $CDCl_3$) δ 8.03 (s, 1H), 7.70 (d, *J* = 8.2 Hz, 2H), 7.47 (d, *J* = 8.2 Hz, 2H), 7.07 (s, 1H), 5.08 (s, 1H), 4.09 (dd, *J* = 10.4, 1.4 Hz, 1H), 3.91 (dd, *J* = 10.4, 2.8 Hz, 1H), 2.61 (s, 3H), 0.79 (s, 9H), −0.02 (s, 6H); $^{13}C\{^1H\}$ NMR (100 MHz, $CDCl_3$) δ 172.3, 165.5, 160.7, 136.5, 130.7 (q, *J* = 32.7 Hz), 128.7 (2C), 128.3, 126.4 (q, *J* = 3.7 Hz, 2C), 123.7 (q, *J* = 270.7 Hz), 115.6, 64.5, 59.2, 26.6, 25.6 (3C), 18.1, −5.85, −5.87; HRMS (ESI/QTOF) *m*/*z*: $[M+H]^+$ Calcd for $C_{21}H_{28}F_3N_2O_4Si$ 457.1765; Found 457.1785.

(Z)-3-[(1,1'-Biphenyl)-4-ylmethylene]-1-acetyl-6-{[(tert-butyldimethylsilyl)oxy]methyl}piperazine-2,5-dione (5o). Following the general procedure A, the reaction was performed using **2o** (36.4 mg, 0.20 mmol), **4** (137 mg, 0.40 mmol) and *t*-BuOK (65.3 mg, 0.58 mmol) in DMF (2.0 mL) for 17 h. The residue was purified by silica gel column chromatography (hexane/EtOAc = 5/1) to give **5o** (77.9 mg, 84%) as a white solid. Mp = 144–146 °C; IR (KBr) ν_{max} = 3184, 3033, 2958, 2929, 2883, 2856, 1695, 1637, 1606, 1489 cm^{-1}; ^{1}H NMR (400 MHz, $CDCl_3$) δ 8.01 (s, 1H), 7.68 (d, *J* = 8.2 Hz, 2H), 7.61 (d, *J* = 7.4 Hz, 2H), 7.49–7.39 (m, 5H), 7.12 (s, 1H), 5.11 (s, 1H), 4.14 (dd, *J* = 10.4, 1.2 Hz, 1H), 3.93 (dd, *J* = 10.4, 2.8 Hz, 1H), 2.62 (s, 3H), 0.81 (s, 9H), −0.01 (s, 6H); $^{13}C\{^1H\}$ NMR (100 MHz, $CDCl_3$) δ 172.4, 165.4, 161.1, 141.8, 139.8, 131.7, 128.94 (2C), 128.92 (2C), 128.1 (2C), 127.9, 127.0 (2C), 126.8, 117.5, 64.5, 59.3, 26.7, 25.6 (3C), 18.1, −5.84 (2C); HRMS (ESI/QTOF) *m*/*z*: $[M+H]^+$ Calcd for $C_{26}H_{33}N_2O_4Si$ 465.2204; Found 465.2200.

(Z)-1-Acetyl-6-{[(tert-butyldimethylsilyl)oxy]methyl}-3-(naphthalen-2-ylmethylene)piperazine-2,5-dione (5p). Following the general procedure A, the reaction was performed using **2p** (31.2 mg, 0.20 mmol), **4** (137 mg, 0.40 mmol) and *t*-BuOK (65.3 mg, 0.58 mmol) in DMF (2.0 mL) for 18 h. The residue was purified by silica gel column chromatography (hexane/EtOAc = 3/1) to give **5p** (70.0 mg, 80%) as a white solid. Mp = 150–152 °C; IR(KBr) ν_{max} = 3197, 3057, 2952, 2925, 2854, 1711, 1701, 1678, 1639, 1624, 1506, 1469 cm^{-1}; ^{1}H NMR (400 MHz, $CDCl_3$) δ 8.14 (s, 1H), 7.91 (d, *J* = 8.5 Hz, 1H), 7.86–7.84 (m, 3H), 7.56– 7.53 (m, 2H), 7.44 (d, *J* = 8.5 Hz, 1H), 7.23 (s, 1H), 5.11 (s, 1H), 4.14 (d, *J* = 10.4 Hz, 1H), 3.94 (dd, *J* = 10.4, 2.6 Hz, 1H), 2.63 (s, 3H), 0.81 (s, 9H), 0.00 (s, 6H); $^{13}C\{^1H\}$ NMR (100 MHz, $CDCl_3$) δ 172.4, 165.5, 161.1, 133.3, 133.0, 130.2, 129.4, 128.2, 128.0, 127.8, 127.2, 127.0, 126.9, 125.6, 118.0, 64.5, 59.3, 26.7, 25.7 (3C), 18.1, −5.83 (2C); HRMS (ESI/QTOF) *m*/*z*: $[M+H]^+$ Calcd for; $C_{24}H_{31}N_2O_4Si$ 439.2048; Found 439.2038.

(Z)-1-Acetyl-6-{[(tert-butyldimethylsilyl)oxy]methyl}-3-(pyren-2-ylmethylene)piperazine-2,5-dione (5q). Following the general procedure A, the reaction was performed using **2q** (46.1 mg, 0.20 mmol), **4** (137 mg, 0.40 mmol) and *t*-BuOK (65.3 mg, 0.58 mmol) in DMF (2.0 mL) for 15 h. The residue was purified by silica gel column chromatography (hexane/EtOAc = 3/1) to give **5q** (88.8 mg, 87%) as a yellow solid. Mp = 83–85 °C; IR (KBr) ν_{max} = 3194, 3037, 2949, 2927, 2881, 2854, 1703, 1633 cm^{-1}; ^{1}H NMR (400 MHz, $CDCl_3$) δ 8.26–8.21 (m, 3H), 8.18–8.14 (m, 3H), 8.10–8.05 (m, 2H), 7.98 (d, *J* = 7.9 Hz, 1H), 7.92 (s, 1H), 7.87 (s, 1H), 5.12 (s, 1H), 4.18 (d, *J* = 10.4 Hz, 1H), 4.00 (dd, *J* = 10.4, 2.6 Hz, 1H), 2.70 (s, 3H), 0.87 (s, 9H), 0.06 (d, *J* = 7.9 Hz, 6H); ^{13}C{^{1}H} NMR (100 MHz, $CDCl_3$) δ 172.4, 165.3, 161.0, 131.9, 131.2, 130.8, 129.3, 128.8, 128.6, 127.9, 127.2, 126.5, 126.4, 126.1, 126.0, 125.9, 125.5, 125.2, 124.4, 123.6, 116.6, 64.6, 59.4, 26.7, 25.8 (3C), 18.3, −5.68, −5.80; HRMS (ESI/QTOF) *m/z*: $[M+H]^+$ Calcd for $C_{30}H_{33}N_2O_4Si$ 513.2204; Found 513.2192.

(*Z*)-1-Acetyl-6-{[(tert-butyldimethylsilyl)oxy]methyl}-3-[(1-(methoxymethyl)-2-(2-methylbut-3-en-2-yl)-1*H*-indol-3-yl]methylenepiperazine-2,5-dione (5s). Following the general procedure A, the reaction was performed using **2s**[2,12] (51.4 mg, 0.20 mmol), **4** (137 mg, 0.40 mmol) and *t*-BuOK (65.3 mg, 0.58 mmol) in DMF (2.0 mL) for 16 h. The residue was purified by silica gel column chromatography (hexane/EtOAc = 4/1) to separate **5s** and the starting materials. The reaction was repeated additionally three times to give **5s** (15.2 mg, 14%) as a yellow amorphous solid and recovered **2s** (31.3 mg, 61%). **5s**: IR (NaCl) ν_{max} = 3367, 3178, 3018, 2954, 2929, 2883, 2858, 1693, 1633 cm^{-1}; ^{1}H NMR (400 MHz, $CDCl_3$) δ 7.42–7.36 (m, 4H), 7.26–7.23 (m, 1H), 7.14 (t, *J* = 7.2 Hz, 1H), 6.19 (dd, *J* = 17.4, 10.6 Hz, 1H), 5.51 (s, 2H), 5.14–5.02 (m, 2H), 5.07 (s, 1H), 4.23 (d, *J* = 10.6 Hz, 1H), 4.02 (dd, *J* = 10.6, 2.8 Hz, 1H), 3.40 (s, 3H), 2.64 (s, 3H), 1.63 (s, 3H), 1.60 (s, 3H), 0.88 (s, 9H), 0.05 (s, 3H), 0.04 (s, 3H); ^{13}C{^{1}H} NMR (100 MHz, $CDCl_3$) δ 172.4, 164.9, 161.0, 146.8, 143.9, 137.9, 125.8, 124.8, 123.2, 121.5, 120.0, 115.4 ,112.2, 110.3, 105.6, 75.2, 64.7, 59.3, 55.7, 40.7, 30.1, 29.6, 26.8, 25.8 (3C), 18.6, −5.43, −5.95; HRMS (ESI/QTOF) *m/z*: $[M+H]^+$ Calcd for $C_{29}H_{42}N_3O_5Si$ 540.2888; Found 540.2899.

Procedure of transformation of the intermediates 5 into methylenepiperazine-2,5-diones 1.

(Z)-3-((1-Acetyl-1H-indol-3-yl)methylene)-6-methylenepiperazine-2,5-dione (1b'). Following the general procedure B, the reaction of **5b** (107 mg, 0.23 mmol) with a 1.0 M TBAF solution in THF (460 μL, 0.46 mmol) in THF (2.0 mL) for 4 h gave the crude product. The residue was purified by trituration with MeOH to give **1b′** (50.8 mg, 75%) as a yellow solid. Mp = 256–257 °C; IR (KBr) ν_{max} = 3178, 3006, 2889, 2821, 1707, 1680, 1643, 1562 cm^{-1}; ^{1}H NMR (400 MHz, DMSO-*d*$_6$) δ 11.02 (s, 1H), 10.25 (s, 1H), 8.36 (d, *J* = 7.4 Hz, 1H), 8.31 (s, 1H), 7.67 (d, *J* = 7.4 Hz, 1H), 7.39 (t, *J* = 7.4 Hz, 1H), 7.34 (t, *J* = 7.4 Hz, 1H), 6.91 (s, 1H), 5.32 (s, 1H), 4.96 (s, 1H), 2.74 (s, 3H); ^{13}C{^{1}H} NMR (100 MHz, DMSO-*d*$_6$) δ 170.1, 157.2, 157.1, 134.78, 134.76, 129.9, 127.1, 126.8, 125.6, 124.1, 118.9, 116.2, 113.0, 104.7, 100.6, 24.2; HRMS (ESI/QTOF) *m/z*: $[M+Na]^+$ Calcd for $C_{16}H_{13}N_3NaO_3$ 318.0849; Found 318.0858.

***(Z)-3-Benzylidene-6-methylenepiperazine-2,5-dione (1c*).** Following the general procedure B, the reaction of **5c** (76.0 mg, 0.20 mmol) with a 1.0 M TBAF solution in THF (390 μL, 0.39 mmol) in THF (2.0 mL) for 4 h gave the crude product. The residue was purified by trituration with MeOH to give **1c** (26.6 mg, 63%) as a white solid. Mp = 242–243 °C; IR (KBr) ν_{max} = 3276, 3180, 3080, 3060, 3016, 2914, 1687, 1641, 1617, 1603 cm^{-1}; ^{1}H NMR

(400 MHz, DMSO-d_6) δ 10.90 (br, 1H), 10.05 (br, 1H), 7.49 (d, J = 7.5 Hz, 2H), 7.40 (t, J = 7.5 Hz, 2H), 7.31 (t, J = 7.5 Hz, 1H), 6.74 (s, 1H), 5.28 (s, 1H), 4.93 (s, 1H); $^{13}C\{^1H\}$ NMR (100 MHz, DMSO-d_6) δ 157.4, 157.0, 134.7, 133.2, 129.5 (2C), 128.9 (2C), 128.3, 126.7, 115.0, 100.6; HRMS (ESI/QTOF) *m/z*: $[M+Na]^+$ Calcd for $C_{12}H_{10}N_2NaO_2$ 237.0635; Found 237.0638.

(Z)-3-(Furan-3-ylmethylene)-6-methylenepiperazine-2,5-dione (1d). Following the general procedure B, the reaction of **5d** (72.2 mg, 0.19 mmol) with a 1.0 M TBAF solution in THF (380 µL, 0.38 mmol) in THF (2.0 mL) for 2 h gave the crude product. The residue was purified by trituration with MeOH to give **1d** (31.3 mg, 80%) as a white solid. Mp = 242–243 °C; IR (KBr) $ν_{max}$ = 3291, 3163, 3143, 3022, 2925, 1693, 1643, 1622 cm^{-1}; ^1H NMR (400 MHz, DMSO-d_6) δ 10.88 (s, 1H), 9.74 (s, 1H), 8.20 (s, 1H), 7.73 (s, 1H), 6.89 (s, 1H), 6.62 (s, 1H), 5.27 (s, 1H), 4.91 (s, 1H); $^{13}C\{^1H\}$ NMR (100 MHz, DMSO-d_6) δ 157.4, 157.1, 144.3, 144.2, 134.8, 125.7, 118.7, 110.9, 106.5, 100.4; HRMS (ESI/QTOF) *m/z*: $[M+Na]^+$ Calcd for $C_{10}H_8N_2NaO_3$ 237.0427; Found 227.0432.

(Z)-3-Methylene-6-(thiophen-3-ylmethylene)piperazine-2,5-dione (1e). Following the general procedure B, the reaction of **5e** (41.5 mg, 0.11 mmol) with a 1.0 M TBAF solution in THF (210 µL, 0.21 mmol) in THF (1.5 mL) for 2 h gave the crude product. The residue was purified by trituration with MeOH to give **1e** (14.8 mg, 64%) as a white solid. Mp = 242–243 °C; IR (KBr) $ν_{max}$ = 3266, 3178, 3124, 3055, 3016, 2921, 1687, 1641 cm^{-1}; ^1H NMR (400 MHz, DMSO-d_6) δ 10.92 (s, 1H), 9.91 (s, 1H), 7.94 (d, J = 2.0 Hz, 1H), 7.62–7.60 (m, 1H), 7.37 (d, J = 5.1 Hz, 1H), 6.79 (s, 1H), 5.28 (s, 1H), 4.92 (s, 1H); $^{13}C\{^1H\}$ NMR (100 MHz, DMSO-d_6) δ 157.6, 157.1, 134.8, 134.0, 129.0, 127.0, 126.7, 125.5, 109.9, 100.5; HRMS (ESI/QTOF) *m/z*: $[M+Na]^+$ Calcd for $C_{10}H_8N_2NaO_2S$ 243.0199; Found 243.0190.

(Z)-3-(3,5-Di-tert-butylbenzylidene)-6-methylenepiperazine-2,5-dione (1f) Following the general procedure B, the reaction of **5f** (84.0 mg, 0.16 mmol) with a 1.0 M TBAF solution in THF (330 µL, 0.33 mmol) in THF (1.5 mL) for 2 h gave the crude product. The residue was purified by trituration with MeOH to give **1f** (34.1 mg, 64%) as a white solid. Mp = 204–205 °C; IR (KBr) $ν_{max}$ = 3176, 3022, 2964, 2904, 1684, 1637 cm^{-1}; ^1H NMR (400 MHz, DMSO-d_6) δ 10.90 (s, 1H), 10.00 (s, 1H), 7.36 (s, 1H), 7.29 (s, 2H), 6.76 (s, 1H), 5.27 (s, 1H), 4.93 (s, 1H), 1.29 (s, 18H); $^{13}C\{^1H\}$ NMR (100 MHz, DMSO-d_6) δ 157.5, 156.9, 150.7 (2C), 134.8, 132.5, 126.2, 123.5 (2C), 122.6, 116.3, 100.4, 34.7 (2C), 31.3 (6C); HRMS (ESI/QTOF) *m/z*: $[M+Na]^+$ Calcd for $C_{20}H_{26}N_2NaO_2$ 349.1887; Found 349.1875.

(Z)-3-(4-(tert-Butyl)benzylidene)-6-methylenepiperazine-2,5-dione (1g). Following the general procedure B, the reaction of **5g** (79.5 mg, 0.18 mmol) with a 1.0 M TBAF solution in THF (360 µL, 0.36 mmol) in THF (1.5 mL) for 3 h gave the crude product. The residue was purified by trituration with MeOH to give **1g** (28.6 mg, 59%) as a white solid. Mp = 212–213 °C; IR (KBr) $ν_{max}$ = 3188, 3020, 2962, 2914, 1691, 1645, 1620 cm^{-1}; ^1H NMR (400 MHz, DMSO-d_6) δ 10.91 (s, 1H), 10.05 (s, 1H), 7.43 (s, 4H), 6.71 (s, 1H), 5.26 (s, 1H), 4.91 (s, 1H), 1.28 (s, 9H); $^{13}C\{^1H\}$ NMR (100 MHz, DMSO-d_6) δ 157.6, 157.0, 151.0, 134.9, 130.6, 129.3 (2C), 126.4, 125.7 (2C), 115.0, 100.4, 34.7, 31.2 (3C); HRMS (ESI/QTOF) *m/z*: $[M+Na]^+$ Calcd for $C_{16}H_{18}N_2NaO_2$ 293.1261; Found 293.1253.

(Z)-3-(4-Methylbenzylidene)-6-methylenepiperazine-2,5-dione (1h). Following the general procedure B, the reaction of **5h** (63.7 mg, 0.16 mmol) with a 1.0 M TBAF solution in THF (320 µL, 0.32 mmol) in THF (1.5 mL)

for 3 h gave the crude product. The residue was purified by trituration with MeOH to give **1h** (29.3 mg, 81%) as a white solid. Mp = 237–239 °C; IR (KBr) ν_{max} = 3168, 3060, 3026, 2918, 2889, 1682, 1635, 1604 cm^{-1}; ^{1}H NMR (400 MHz, DMSO-*d$_6$*) δ 10.91 (s,1H), 10.02 (s,1H), 7.39 (d, *J* = 8.0 Hz, 2H), 7.21 (d, *J* = 8.0 Hz, 2H), 6.70 (s, 1H), 5.27 (s, 1H), 4.92 (s, 1H), 2.31 (s, 3H); ^{13}C{^{1}H} NMR (100 MHz, DMSO-*d$_6$*) δ 157.5, 157.0, 138.0, 134.8, 130.4, 129.49 (2C), 129.47 (2C), 126.1, 115.1, 100.3, 21.1; HRMS (ESI/QTOF) *m*/*z*: [M+Na]$^+$ Calcd for $C_{13}H_{12}N_2NaO_2$ 251.0791; Found 251.0782.

(Z)-3-(4-Methoxybenzylidene)-6-methylenepiperazine-2,5-dione (1i). Following the general procedure B, the reaction of **5i** (52.2 mg, 0.13 mmol) with a 1.0 M TBAF solution in THF (250 µL, 0.25 mmol) in THF (1.5 mL) for 2 h gave the crude product. The residue was purified by trituration with MeOH to give **1i** (20.8 mg, 71%) as a yellow solid. Mp = 229–231 °C; IR (KBr) ν_{max} = 3176, 3012, 2925, 2833, 2349, 1684, 1641, 1603, 1574, 1514 cm^{-1}; ^{1}H NMR (400 MHz, DMSO-*d$_6$*) δ 10.90 (s, 1H), 10.08 (s, 1H), 7.47 (d, *J* = 8.6 Hz, 2H), 6.97 (d, *J* = 8.6 Hz, 2H), 6.70 (s, 1H), 5.25 (s, 1H), 4.90 (s, 1H), 3.78 (s, 3H); ^{13}C{^{1}H} NMR (100 MHz, DMSO-*d$_6$*) δ 159.4, 157.7, 157.0, 134.9, 131.2 (2C), 125.6, 125.1, 115.3, 114.4 (2C), 100.2, 55.5; HRMS (ESI/QTOF) *m*/*z*: [M+Na]$^+$ Calcd for $C_{13}H_{12}N_2NaO_3$ 267.0740; Found 267.0750.

(Z)-3-(3,4-Dimethoxybenzylidene)-6-methylenepiperazine-2,5-dione (1j). Following the general procedure B, the reaction of **5j** (63.0 mg, 0.14 mmol) with a 1.0 M TBAF solution in THF (280 µL, 0.28 mmol) in THF (1.5 mL) for 3.5 h gave the crude product. The residue was purified by trituration with MeOH to give **1j** (21.8 mg, 62%) as a yellow solid. Mp = 237–238 °C; IR (KBr) ν_{max} = 3442, 3184, 3041, 1691, 1684, 1641, 1603, 1581, 1516 cm^{-1}; ^{1}H NMR (400 MHz, DMSO-*d$_6$*) δ 10.88 (s, 1H), 7.09–7.07 (m, 2H), 6.98 (d, *J* = 9.1 Hz, 1H), 6.71 (s, 1H), 5.26 (s, 1H), 4.91 (s, 1H), 3.78 (s, 3H), 3.77 (s, 3H). One NH proton was not observed; ^{13}C{^{1}H} NMR (100 MHz, DMSO-*d$_6$*) δ 157.6, 157.0, 149.2, 148.7, 134.8, 125.8, 125.1, 122.7, 115.7, 113.1, 112.0, 100.2, 55.7, 55.6; HRMS (ESI/QTOF) *m*/*z*: [M+Na]$^+$ Calcd for $C_{14}H_{14}N_2NaO_4$ 297.0846; Found 297.0839.

(Z)-3-Methylene-6-(3,4,5-trimethoxybenzylidene)piperazine-2,5-dione (1k). Following the general procedure B, the reaction of **5k** (83.9 mg, 0.18 mmol) with a 1.0 M TBAF solution in THF (350 µL, 0.35 mmol) in THF (1.5 mL) for 5 h gave the crude product. The residue was purified by trituration with MeOH to give **1k** (39.7 mg, 75%) as a white solid. Mp = 219–221 °C; IR (KBr) ν_{max} = 3172, 3016, 2829, 1682, 1643, 1579, 1508 cm^{-1}; ^{1}H NMR (400 MHz, DMSO-*d$_6$*) δ 10.91 (s, 1H), 10.22 (s, 1H), 6.77 (s, 2H), 6.70 (s, 1H), 5.27 (s, 1H), 4.92 (s, 1H), 3.79 (s, 6H), 3.67 (s, 3H); ^{13}C{^{1}H} NMR (100 MHz, DMSO-*d$_6$*) δ 157.4, 157.0, 153.0 (2C), 137.8, 134.8, 128.6, 126.4, 115.4, 107.1 (2C), 100.3, 60.2, 56.1 (2C); HRMS (ESI/QTOF) *m*/*z*: [M+Na]$^+$ Calcd for $C_{15}H_{16}N_2NaO_5$ 327.0951; Found 327.0941.

Methyl (Z)-4-((5-methylene-3,6-dioxopiperazin-2-ylidene)methyl)benzoate (1l). Following the general procedure B, the reaction of **5*l*** (64.9 mg, 0.15 mmol) with a 1.0 M TBAF solution in THF (290 µL, 0.29 mmol) in THF (1.5 mL) for 3.5 h gave the crude product. The residue was purified by trituration with MeOH to give **1*l*** (20.2 mg, 51%) as a white solid. Mp = 248–249 °C; IR (KBr) ν_{max} = 3273, 3170, 3022, 2927, 1734, 1689, 1643, 1612, 1475 cm^{-1}; ^{1}H NMR (400 MHz, DMSO-*d$_6$*) δ 11.03 (s, 1H), 10.41 (s, 1H), 7.94 (d, *J* = 8.4 Hz, 2H), 7.61 (d, *J* = 8.4 Hz, 2H), 6.75 (s, 1H), 5.30 (s, 1H), 4.95 (s, 1H), 3.85 (s, 3H); ^{13}C{^{1}H} NMR (100 MHz, DMSO-*d$_6$*) δ

166.1, 157.2, 157.1, 138.3, 134.6, 129.7 (2C), 129.5 (2C), 128.7, 128.5, 113.3, 100.9, 52.4; HRMS (ESI/QTOF) *m*/*z*: $[M+Na]^+$ Calcd for $C_{14}H_{12}N_2NaO_4$ 295.0689; Found 295.0691.

(Z)-3-(4-(Dimethylamino)benzylidene)-6-methylenepiperazine-2,5-dione (1m). Following the general procedure B, the reaction of **5m** (47.4 mg, 0.11 mmol) with a 1.0 M TBAF solution in THF (220 µL, 0.22 mmol) in THF (1.5 mL) for 2 h gave the crude product. The residue was purified by trituration with MeOH to give **1m** (26.6 mg, 94%) as a yellow solid. Mp = 225–227 °C; IR (KBr) ν_{max} = 3257, 3153, 3030, 3006, 2902, 1682, 1643, 1599, 1527, 1475 cm^{-1}; ^1H NMR (400 MHz, DMSO-d_6) δ 10.77 (s, 1H), 9.84 (s, 1H), 7.39 (d, J = 8.8 Hz, 2H), 6.72 (d, J = 8.8 Hz, 2H), 6.67 (s, 1H), 5.22 (s, 1H), 4.87 (s, 1H), 2.95 (s, 6H); $^{13}C\{^1H\}$ NMR (100 MHz, DMSO-d_6) δ 157.9, 156.9, 150.3, 135.0, 131.0 (2C), 122.9, 120.5, 116.7, 112.1 (2C), 99.5, 39.9 (2C); HRMS (ESI/QTOF) *m*/*z*: $[M+H]^+$ Calcd for $C_{14}H_{16}N_3O_2$ 258.1237; Found 258.1234.

(Z)-3-Methylene-6-(4-(trifluoromethyl)benzylidene)piperazine-2,5-dione (1n). Following the general procedure B, the reaction of **5n** (57.8 mg, 0.13 mmol) with a 1.0 M TBAF solution in THF (260 µL, 0.26 mmol) in THF (1.5 mL) for 3.5 h gave the crude product. The residue was purified by trituration with MeOH to give **1n** (25.0 mg, 70%) as a yellow solid. Mp = 244–245 °C; IR (KBr) ν_{max} = 3180, 3037, 3020, 2902, 2821, 1685, 1641, 1630, 1479 cm^{-1}; ^1H NMR (400 MHz, DMSO-d_6) δ 10.99 (s, 1H), 10.46 (br, 1H), 7.73–7.67 (m, 4H), 6.75 (s, 1H), 5.29 (s, 1H), 4.94 (s, 1H); $^{13}C\{^1H\}$ NMR (100 MHz, DMSO-d_6) δ 157.2, 157.1, 137.7, 134.7, 130.2 (2C), 128.7 128.0 (q, J = 31.5 Hz), 125.6 (q, J = 3.7 Hz, 2C), 124.5 (q, J = 270.3 Hz), 113.0, 101.0; HRMS (ESI/QTOF) *m*/*z*: $[M+Na]^+$ Calcd for $C_{13}H_9F_3N_2NaO_2$ 305.0508; Found 305.0506.

(Z)-3-([1,1'-Biphenyl]-4-ylmethylene)-6-methylenepiperazine-2,5-dione (1o). Following the general procedure B, the reaction of **5o** (67.8 mg, 0.15 mmol) with a 1.0 M TBAF solution in THF (290 µL, 0.29 mmol) in THF (1.5 mL) for 2 h gave the crude product. The residue was purified by trituration with MeOH to give **1o** (34.3 mg, 81%) as a yellow solid. Mp = 229–230 °C; IR (KBr) ν_{max} = 3174, 3072, 3047, 3026, 2902, 1687, 1641, 1601, 1552, 1486 cm^{-1}; ^1H NMR (400 MHz, DMSO-d_6) δ 10.98 (s, 1H), 10.25 (s, 1H), 7.73–7.70 (m, 4H), 7.60 (d, J = 8.0 Hz, 2H), 7.48 (t, J = 7.5 Hz, 2H), 7.38 (t, J = 7.5 Hz, 1H), 6.78 (s, 1H), 5.29 (s, 1H), 4.94 (s, 1H); $^{13}C\{^1H\}$ NMR (100 MHz, DMSO-d_6) δ 157.4, 157.1, 139.8, 139.7, 134.8, 132.5, 130.2 (2C), 129.3 (2C), 128.0, 127.0 (2C), 126.9, 126.8 (2C), 114.6, 100.6; HRMS (ESI/QTOF) *m*/*z*: $[M+Na]^+$ Calcd for $C_{18}H_{14}N_2NaO_2$ 313.0948; Found 313.0941.

(Z)-3-Methylene-6-(naphthalen-2-ylmethylene)piperazine-2,5-dione (1p). Following the general procedure B, the reaction of **5p** (67.8 mg, 0.16 mmol) with a 1.0 M TBAF solution in THF (310 µL, 0.31 mmol) in THF (1.5 mL) for 2 h gave the crude product. The residue was purified by trituration with MeOH to give **1p** (35.5 mg, 87%) as a yellow solid. Mp = 216–217 °C; IR (KBr) ν_{max} = 3289, 3176, 3049, 2910, 1687, 1643, 1610, 1595, 1463 cm^{-1}; ^1H NMR (400 MHz, DMSO-d_6) δ 10.99 (s, 1H), 10.28 (s, 1H), 8.08 (s, 1H), 7.95–7.91 (m, 3H), 7.58–7.51 (m, 3H), 6.89 (s, 1H), 5.30 (s, 1H), 4.95 (s, 1H); $^{13}C\{^1H\}$ NMR (100 MHz, DMSO-d_6) δ 157.4, 157.2, 134.8, 133.1, 132.7, 130.8, 128.8, 128.5, 128.2, 127.7, 127.3, 127.1, 126.8, 126.6, 115.0, 100.6; HRMS (ESI/QTOF) *m*/*z*: $[M+Na]^+$ Calcd for $C_{16}H_{12}N_2NaO_2$ 287.0791; Found 287.0786.

(Z)-3-Methylene-6-(pyren-2-ylmethylene)piperazine-2,5-dione (1q). Following the general procedure B, the reaction of **5q** (88.8 mg, 0.17 mmol) with a 1.0 M TBAF solution in THF (350 µL, 0.35 mmol) in THF (1.5 mL)

for 2 h gave the crude product. The residue was purified by trituration with MeOH to give **1q** (45.6 mg, 78%) as a yellow solid. Mp = 265–266 °C; IR (KBr) ν_{max} = 3163, 3033, 2897, 1682, 1633, 1606 cm^{-1}; ^{1}H NMR (400 MHz, DMSO-d_6) δ 10.95 (br, 1H), 10.23 (br, 1H), 8.34–8.30 (m, 3H), 8.26 (d, J = 9.2 Hz, 1H), 8.21 (s, 2H), 8.16 (d, J = 9.2 Hz, 1H), 8.12–8.07 (m, 2H), 7.47 (s, 1H), 5.30 (s, 1H), 4.98 (s, 1H); ^{13}C{^{1}H} NMR (100 MHz, DMSO-d_6) δ 157.2, 157.0, 134.9, 131.0, 130.9, 130.6, 129.0, 128.8, 128.2, 128.1, 127.8, 127.6, 127.3, 126.6, 125.8, 125.7, 125.2, 124.4, 124.2, 124.1, 113.0, 100.6; HRMS (ESI/QTOF) *m*/*z*: [M+Na]$^+$ Calcd for $C_{22}H_{14}N_2NaO_2$ 361.0948; Found 361.0939.

(*Z*)-3-{[(1-(Methoxymethyl)-2-(2-methylbut-3-en-2-yl)-1*H*-indol-3-yl]methylene}-6-methylenepiperazine-2,5-dione (1s). Following the general procedure B, the reaction of **5s** (28.2 mg, 52.2 μmol) with a 1.0 M TBAF solution in THF (104 μL, 104 mmol) in THF (1.0 mL) for 2 h gave the crude product. The residue was purified by silica gel column chromatography (hexane/EtOAc = 3/1) to give **1s** (16.0 mg, 84%) as a brown solid. Mp = 200–201 °C; IR (KBr) ν_{max} = 3176, 2991, 2970, 2814, 1684, 1643 cm^{-1}; ^{1}H NMR (400 MHz, $CDCl_3$) δ 9.10 (s, 1H), 7.53 (s, 1H), 7.43 (d, J = 7.7 Hz, 1H), 7.28–7.24 (m, 2H), 7.26 (s, 1H), 7.17 (t, J = 7.7 Hz, 1H), 6.19 (dd, J = 17.5, 10.5 Hz, 1H), 5.61 (s, 1H), 5.51 (s, 2H), 5.12 (d, J = 10.5 Hz, 1H), 5.05 (d, J = 17.5 Hz, 1H), 5.00 (s, 1H), 3.40 (s, 3H), 1.60 (s, 6H); ^{13}C{^{1}H} NMR (100 MHz, $CDCl_3$) δ 157.7, 155.9, 146.7, 143.5, 138.0, 133.3, 125.5, 125.0, 123.3, 121.5, 119.0, 114.5 ,112.2, 110.4, 105.0, 102.7, 75.2, 55.7, 40.6, 29.8 (2C); HRMS (ESI/QTOF) *m*/*z*: [M+Na]$^+$ Calcd for $C_{21}H_{23}N_3NaO_3$ 388.1632; Found 388.1645.

Details for the biological activities

Figure S1. Cytotoxicity of **1a–q** against Huh7.5.1 cells.[a]

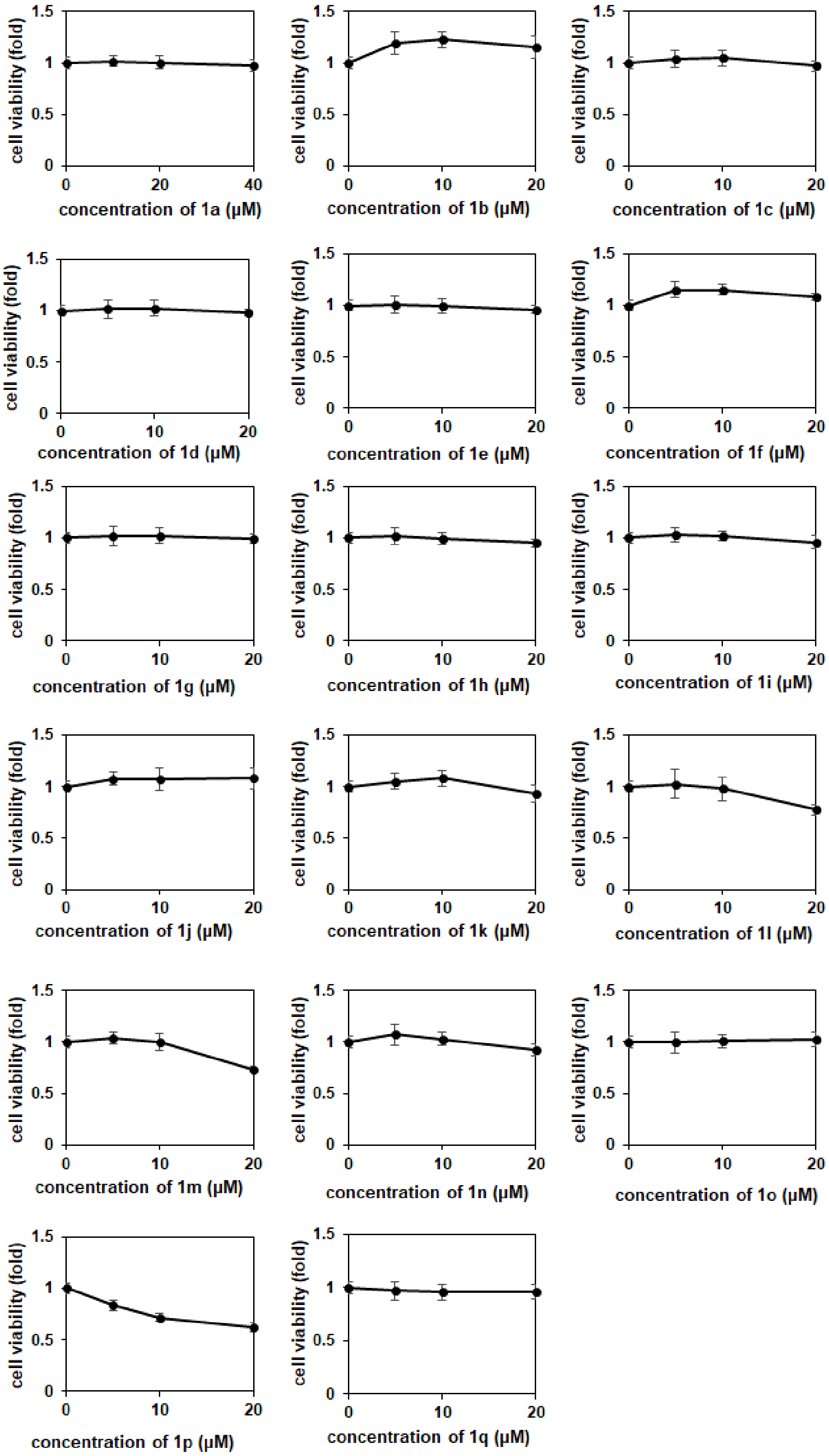

[a]Cell viability was examined by the MTT assay. The experiments were performed in triplicate. The values shown are the means ± SD.

Figure S2. Anti-HCV activity of **1a–q** and **6–8**.[a]

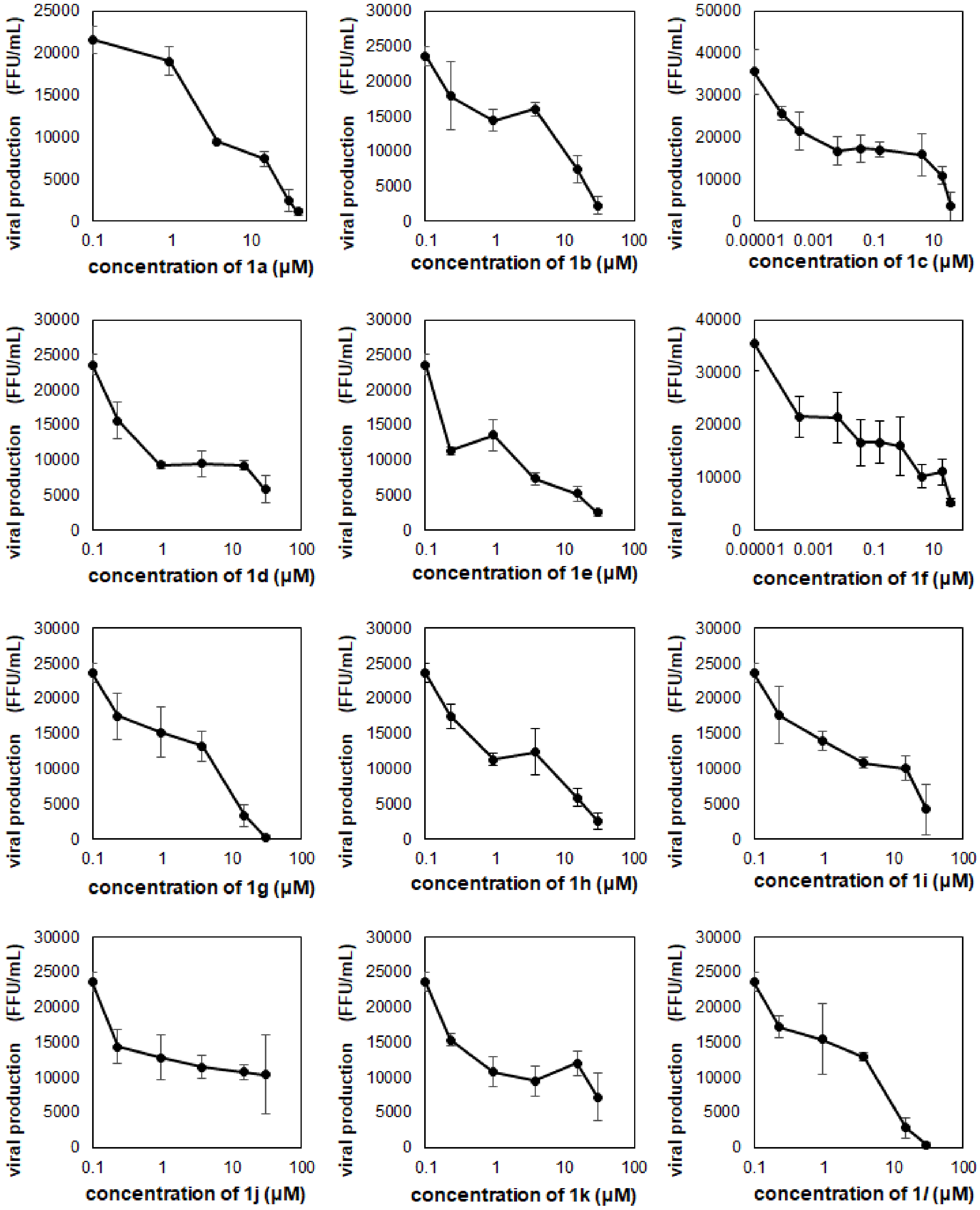

(Figure continued on next page)

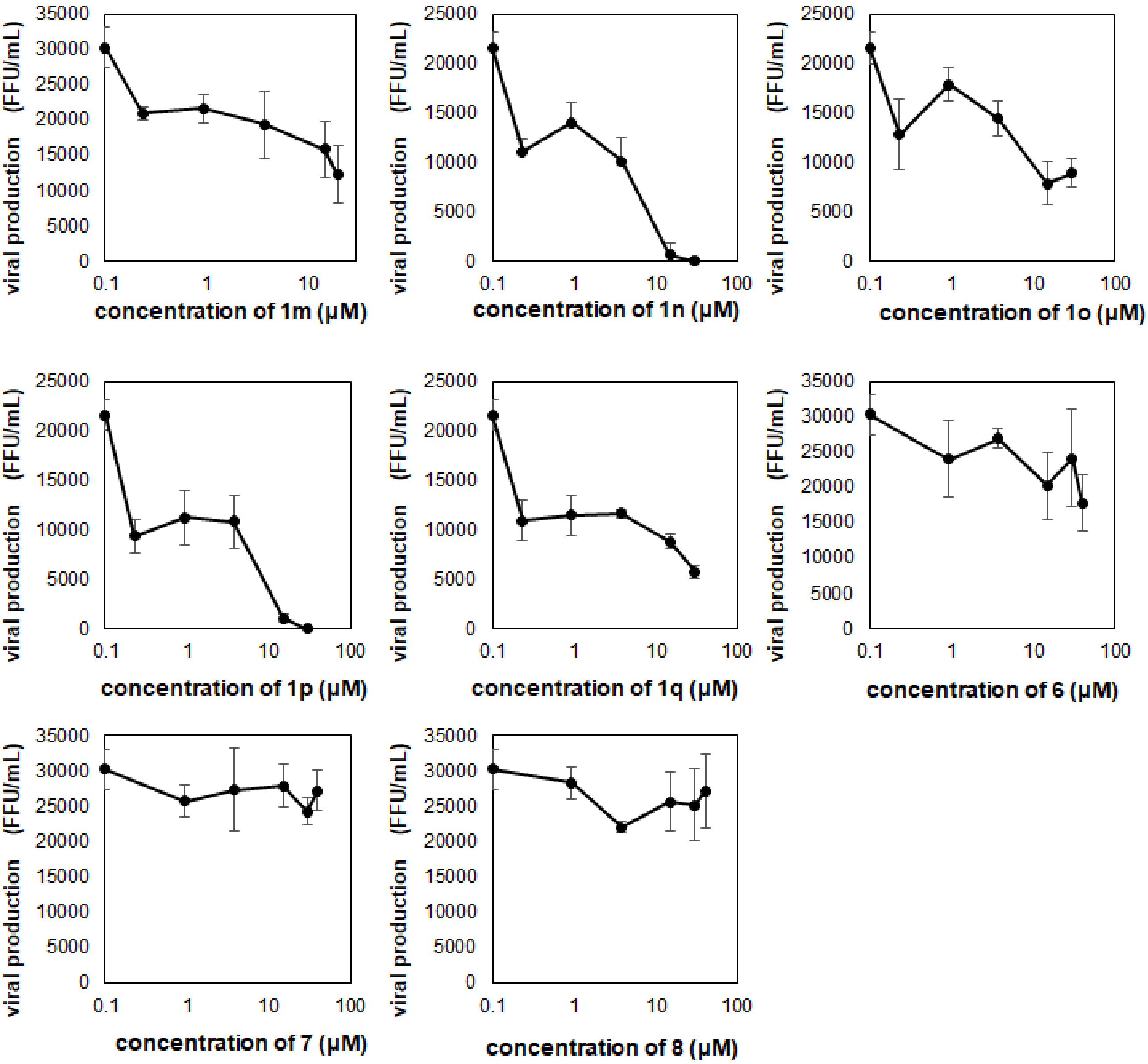

[a]Viral production upon treatment of compounds at various concentrations was determined. The experiments were performed in triplicate. The values shown are the means ± SD.

Figure S3. Cytotoxicity of **1a–q** and **6–8** against VeroE6/TMPRSS2 cells.[a]

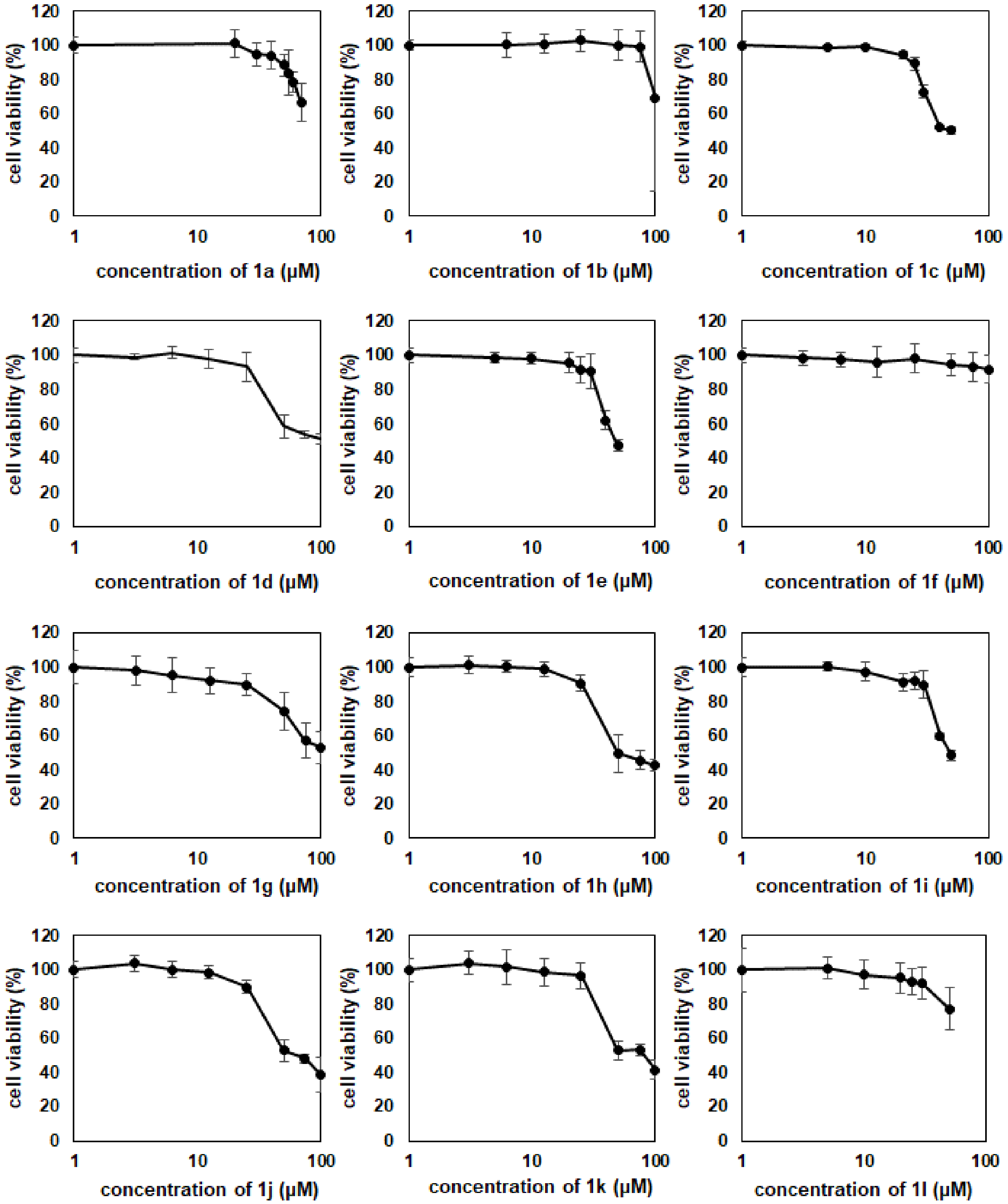

(Figure continued on next page)

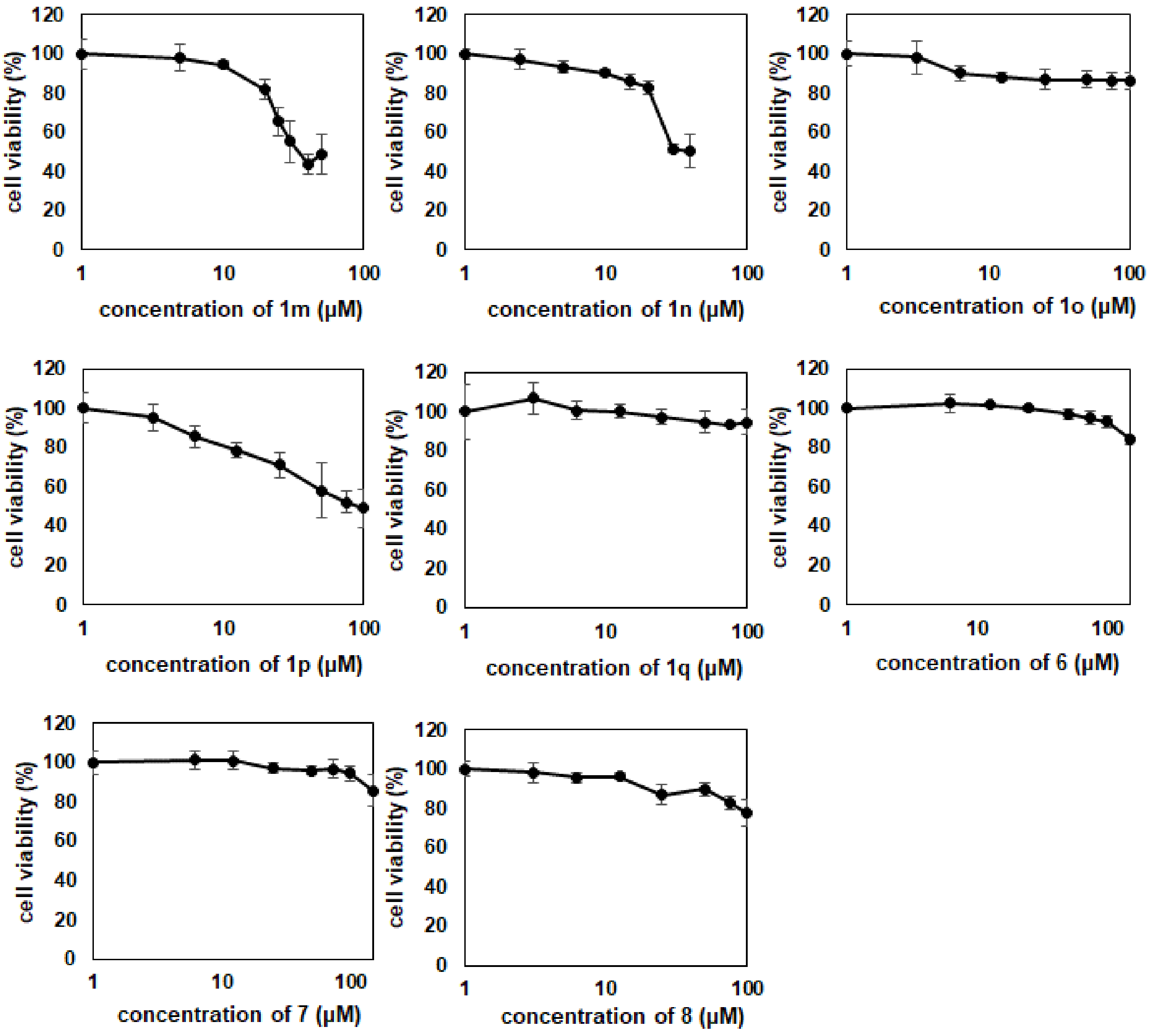

[a]Cell viability was examined by the quantification of survival cell numbers after fixation with 4% paraformaldehyde and staining with 0.02% DAPI. The values shown are the means ± SD of triplicate measurements.

Figure S4. Anti-SARS-CoV-2 activity of **1a**,**1c**, **1d**, **1h**, **1j**, **1*l***, **1n**, and **1o**.[a]

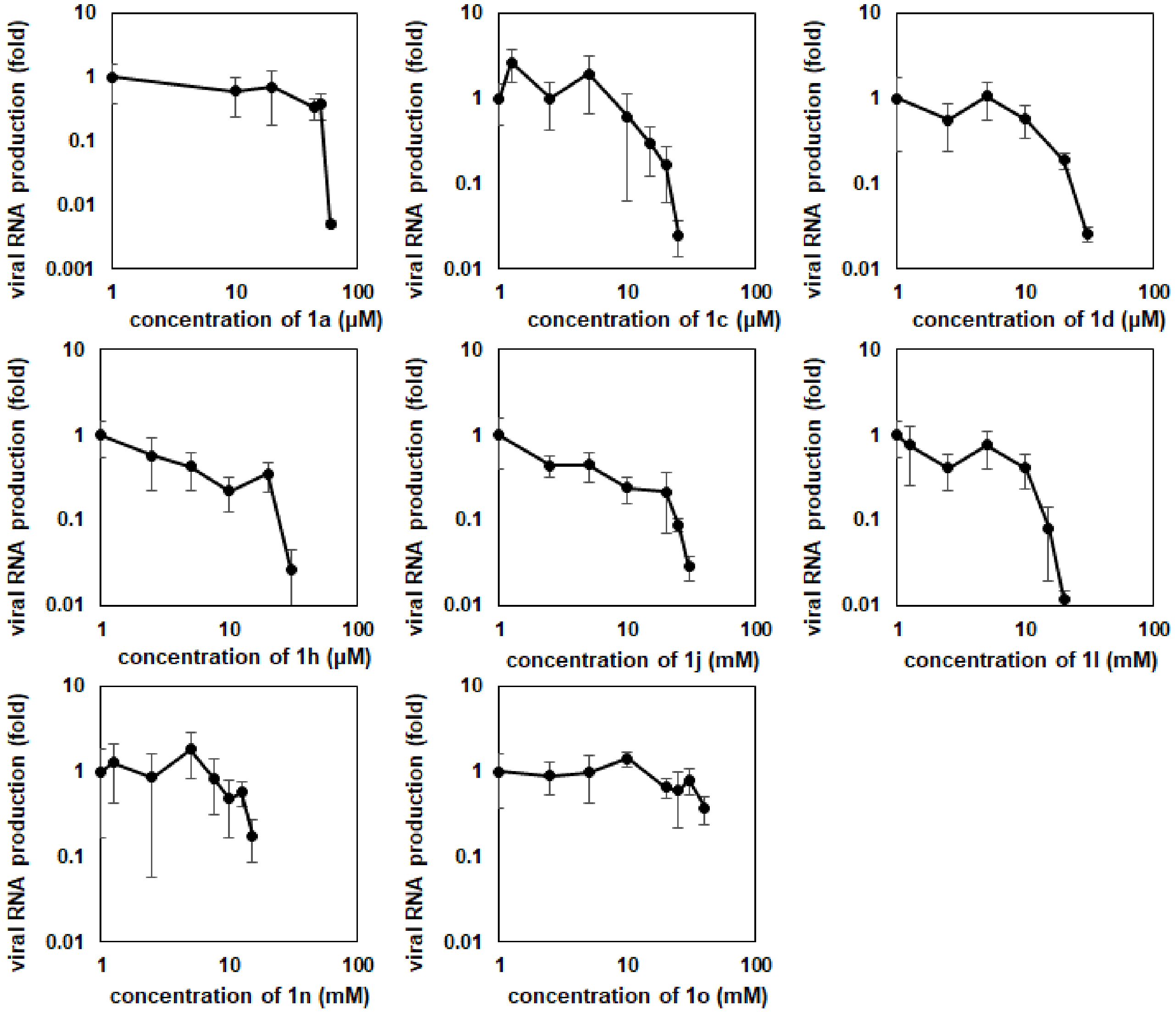

[a]Extracellular SARS-CoV-2 RNA was quantified upon treatment with these compounds at varying concentrations. The relative viral RNA upon treatment of compounds compared to the DMSO control was determined. The values shown are the means ± SD of triplicate measurements.

^{1}H and ^{13}C NMR spectroscopic data

Figure S5. ^{1}H NMR spectrum (400 MHz, $CDCl_3$) of **5b**.

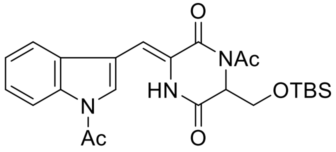

5b

Chemical Formula: $C_{24}H_{31}N_3O_5Si$

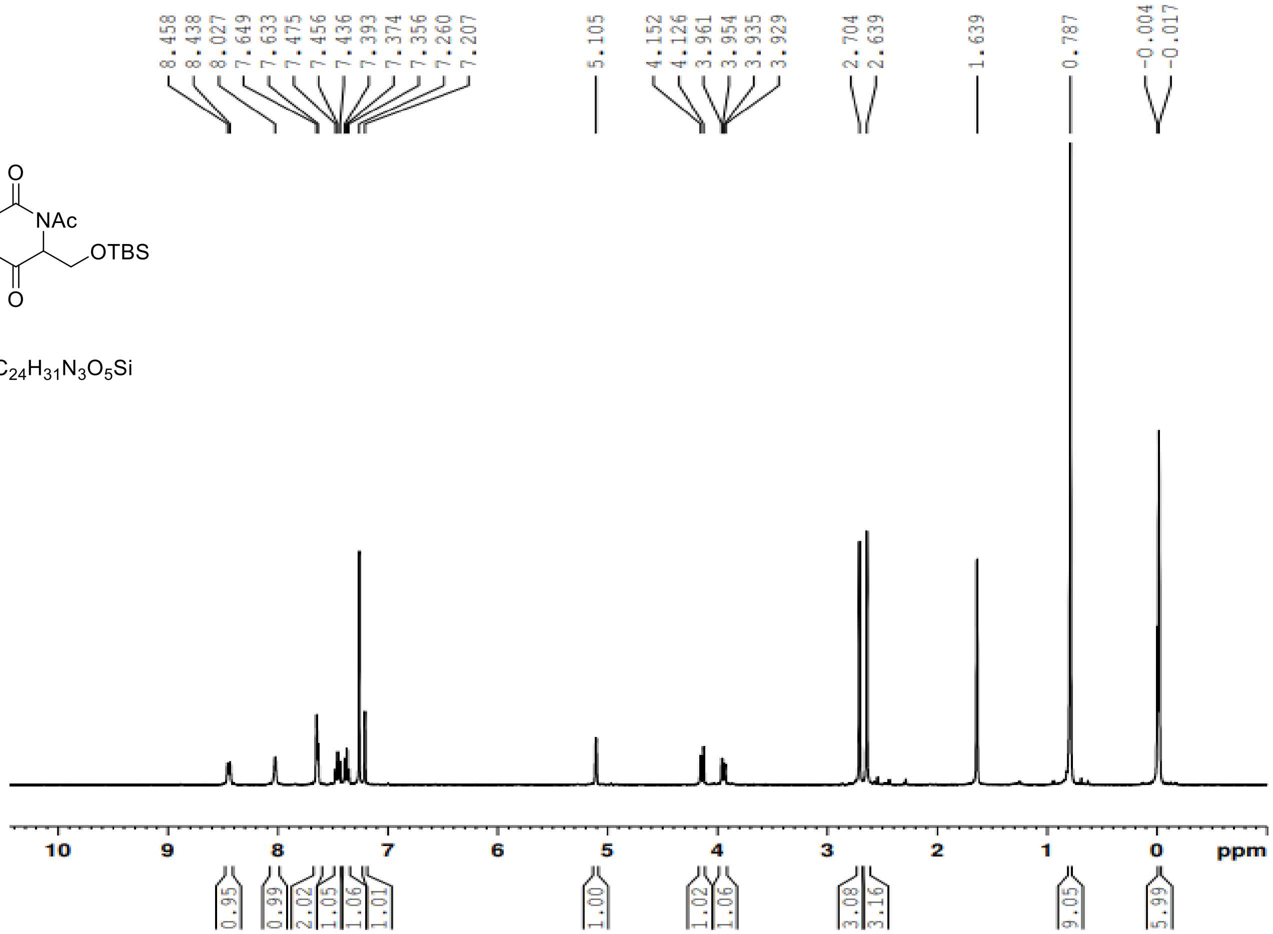

Figure S6. $^{13}C\{^{1}H\}$ NMR spectrum (100 MHz, $CDCl_3$) of **5b**.

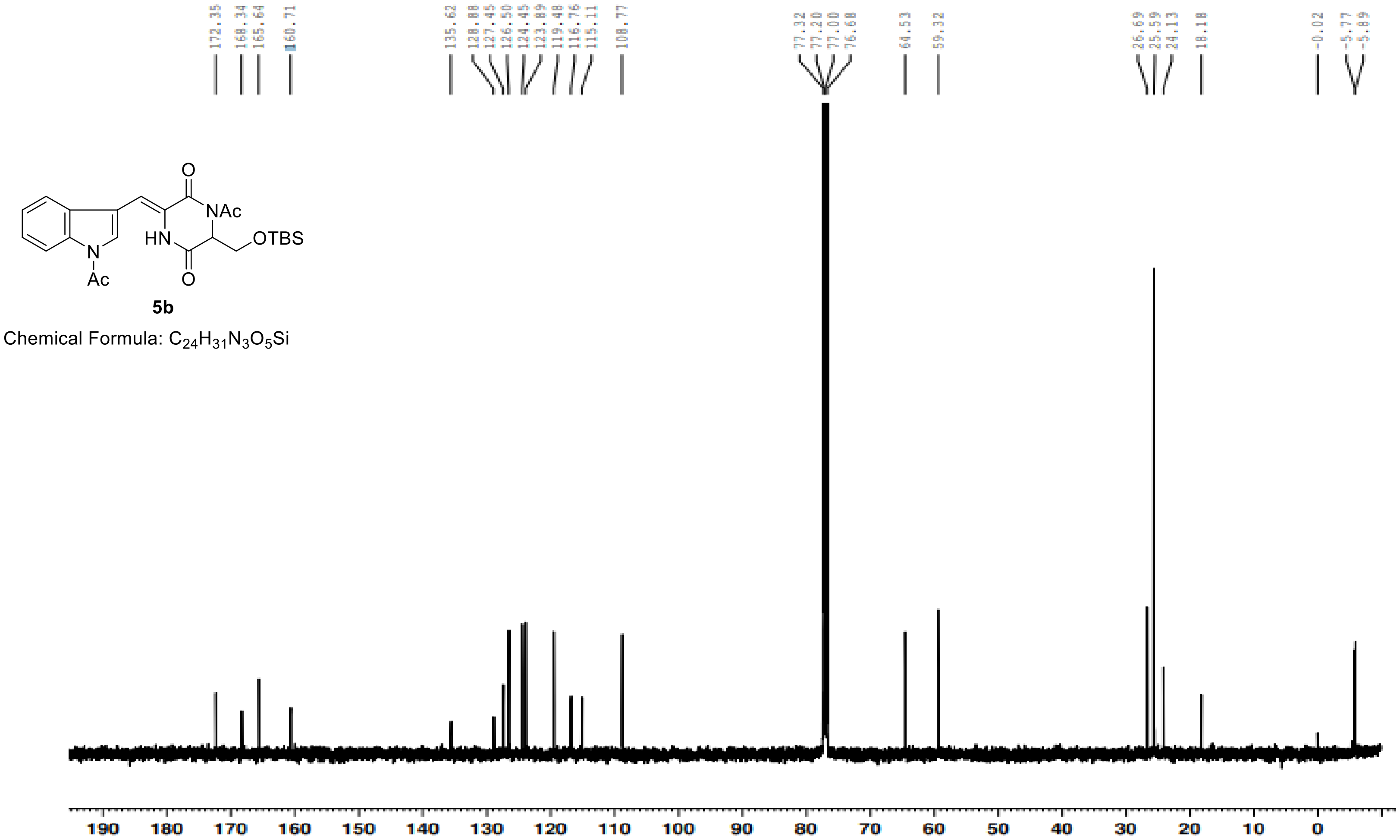

Figure S7. ^{1}H NMR spectrum (400 MHz, $CDCl_3$) of **5c**.

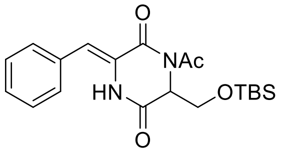

5c

Chemical Formula: $C_{20}H_{28}N_2O_4Si$

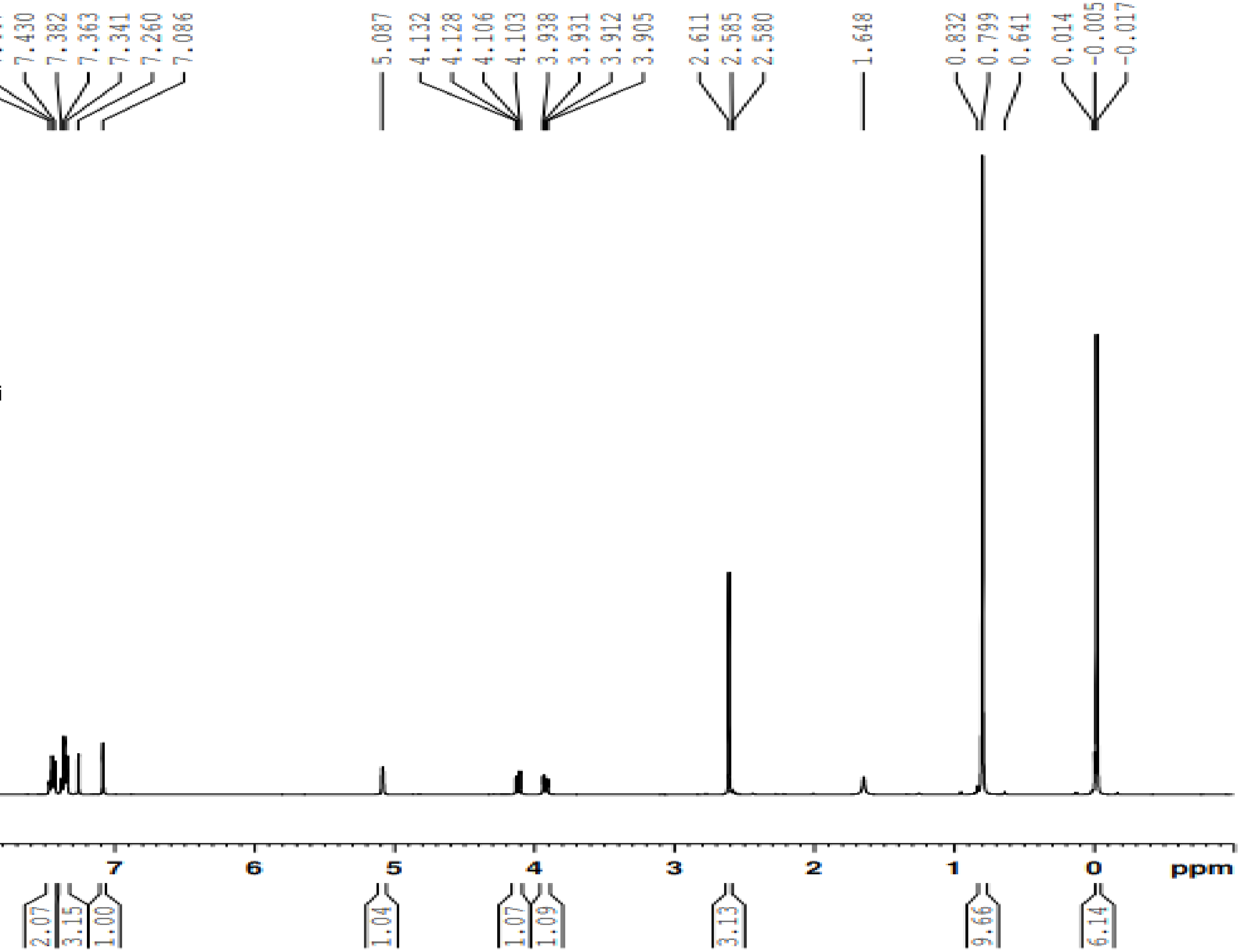

Figure S8. $^{13}C\{^1H\}$ NMR spectrum (100 MHz, $CDCl_3$) of **5c**.

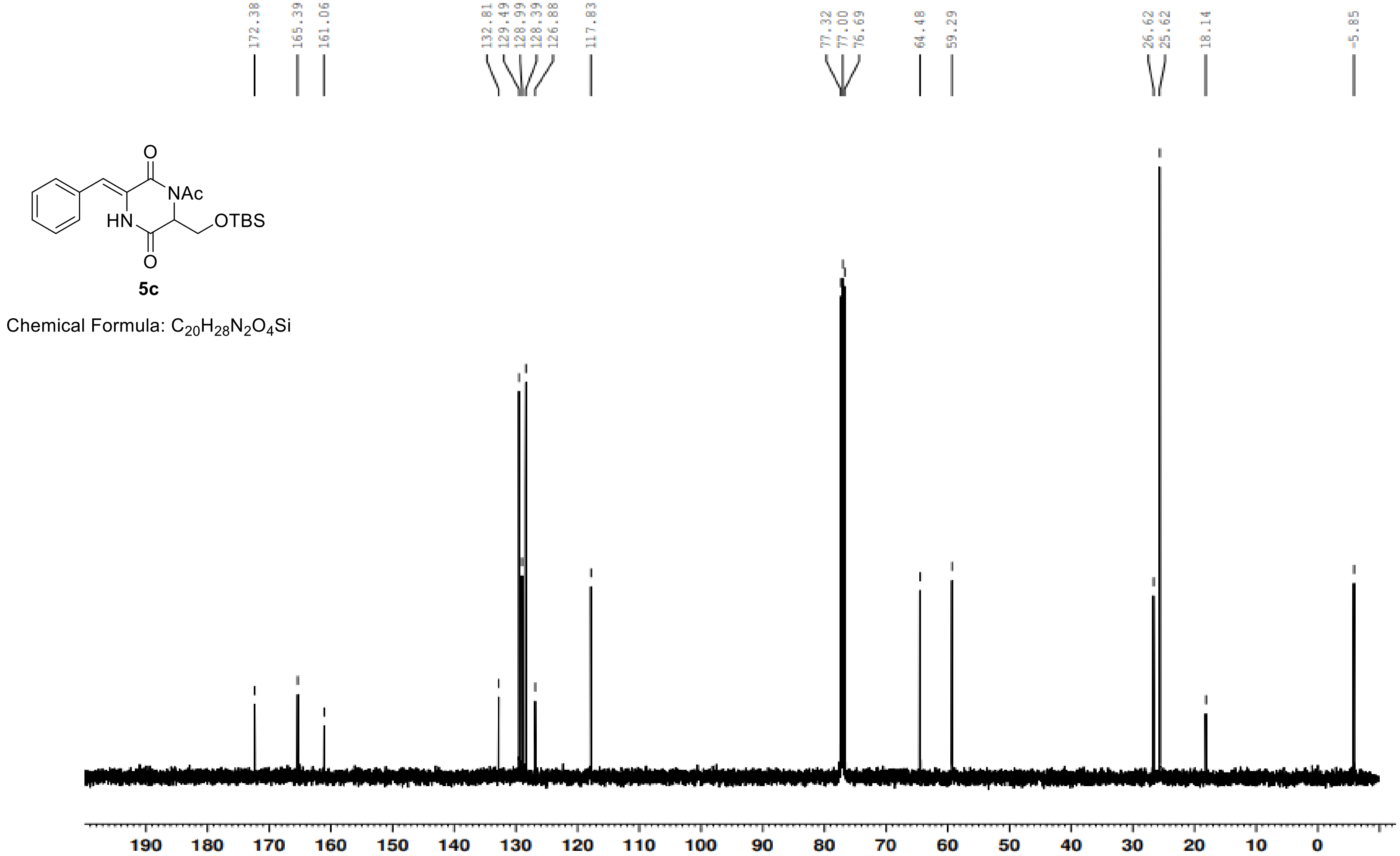

Figure S9. ^{1}H NMR spectrum (400 MHz, $CDCl_3$) of **5d**.

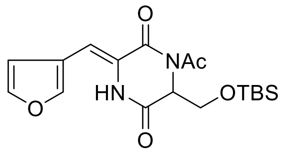

5d

Chemical Formula: $C_{18}H_{26}N_2O_5Si$

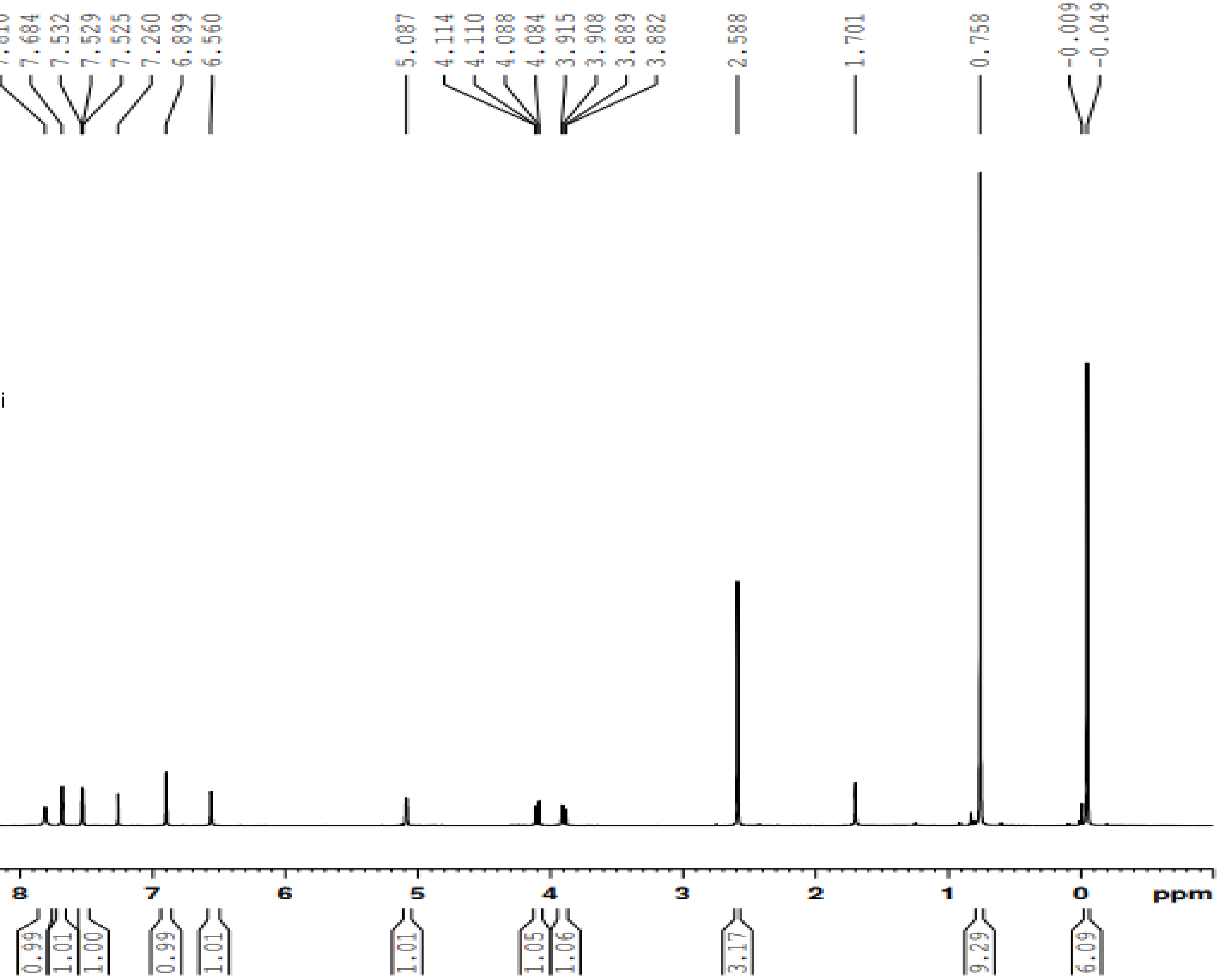

Figure S10. $^{13}C\{^1H\}$ NMR spectrum (100 MHz, $CDCl_3$) of **5d**.

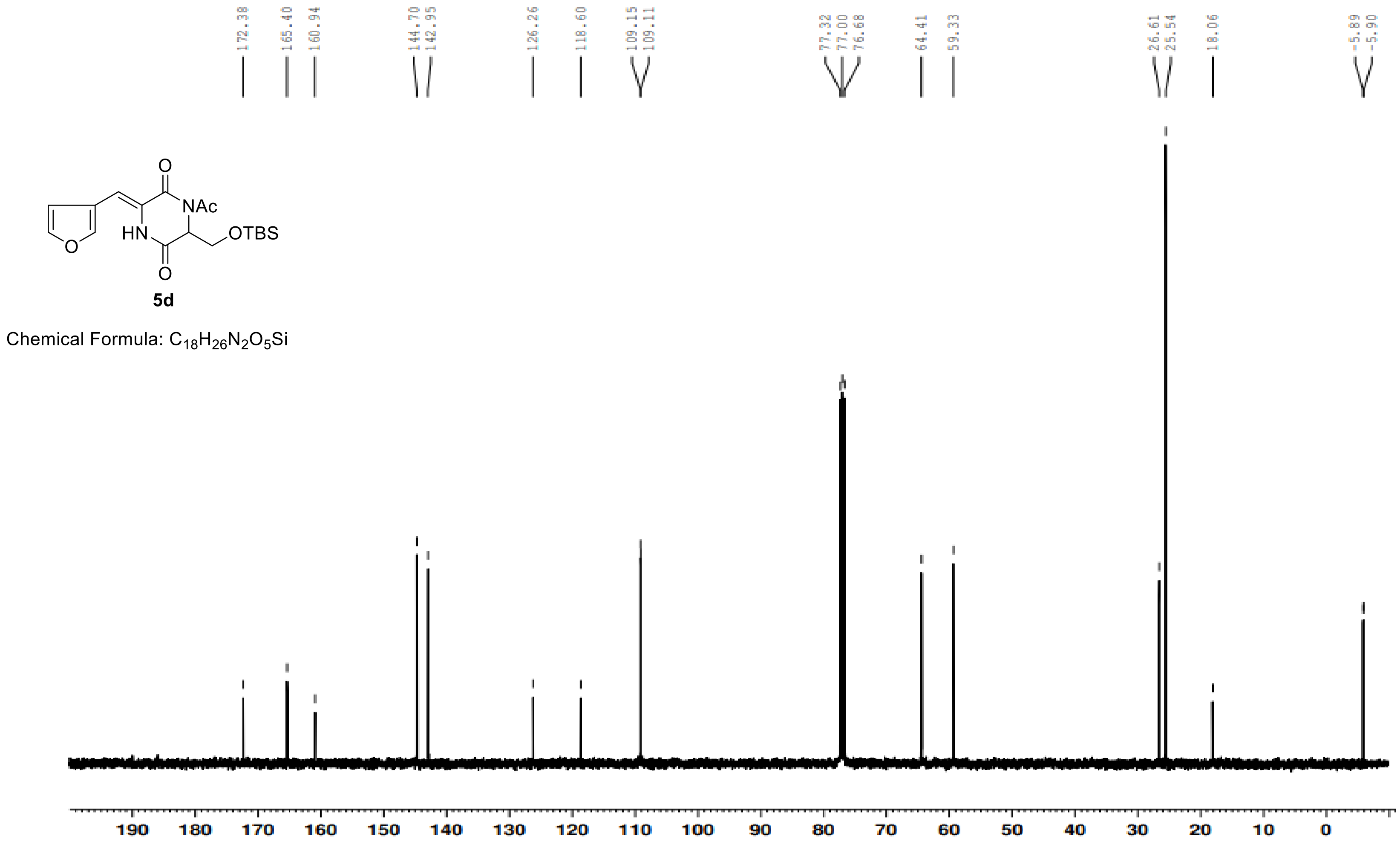

Figure S11. ^{1}H NMR spectrum (400 MHz, $CDCl_3$) of **5e**.

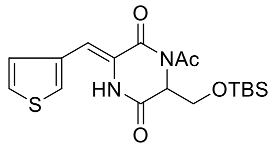

5e

Chemical Formula: $C_{18}H_{26}N_2O_4SSi$

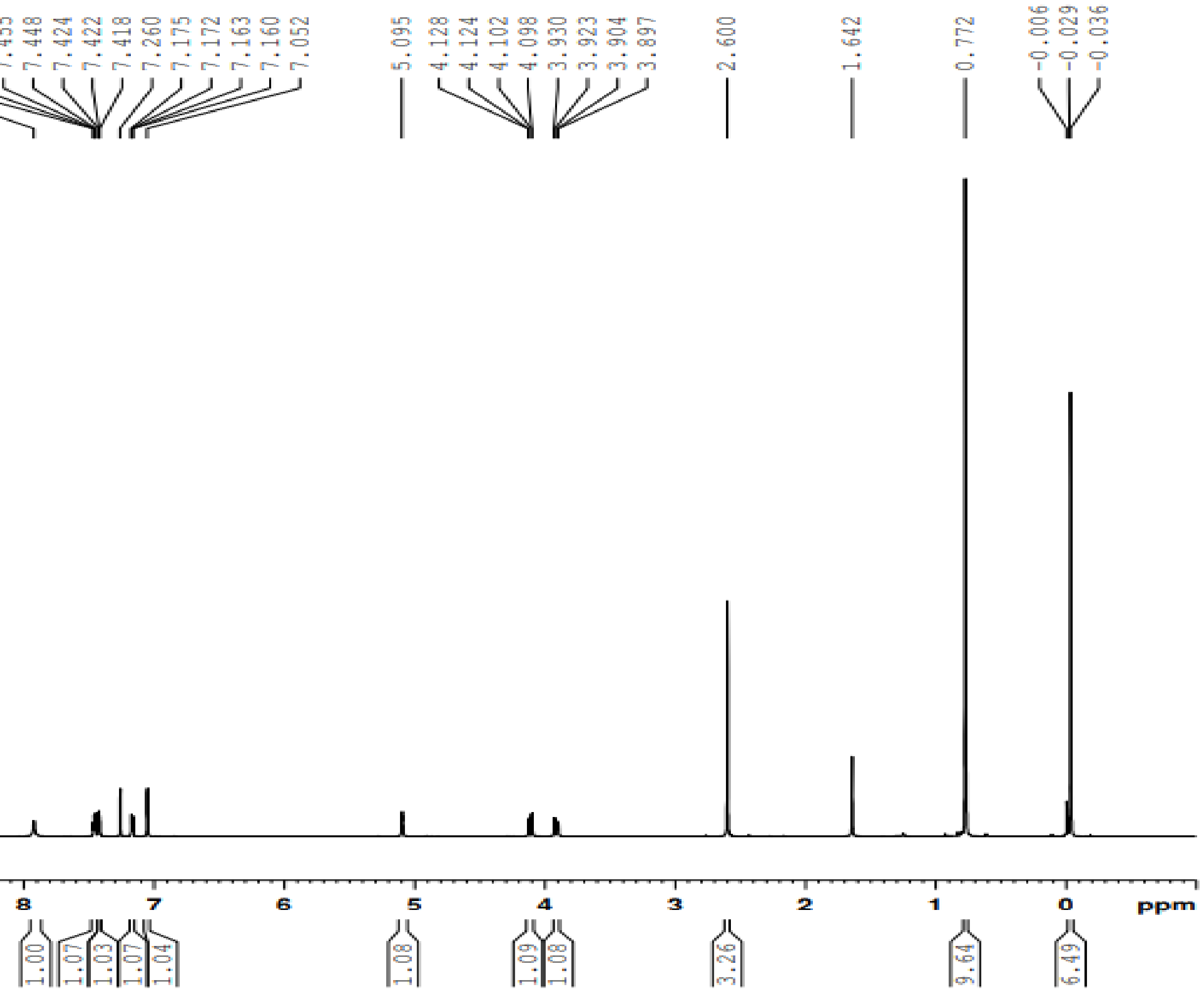

Figure S12. $^{13}C\{^1H\}$ NMR spectrum (100 MHz, $CDCl_3$) of **5e**.

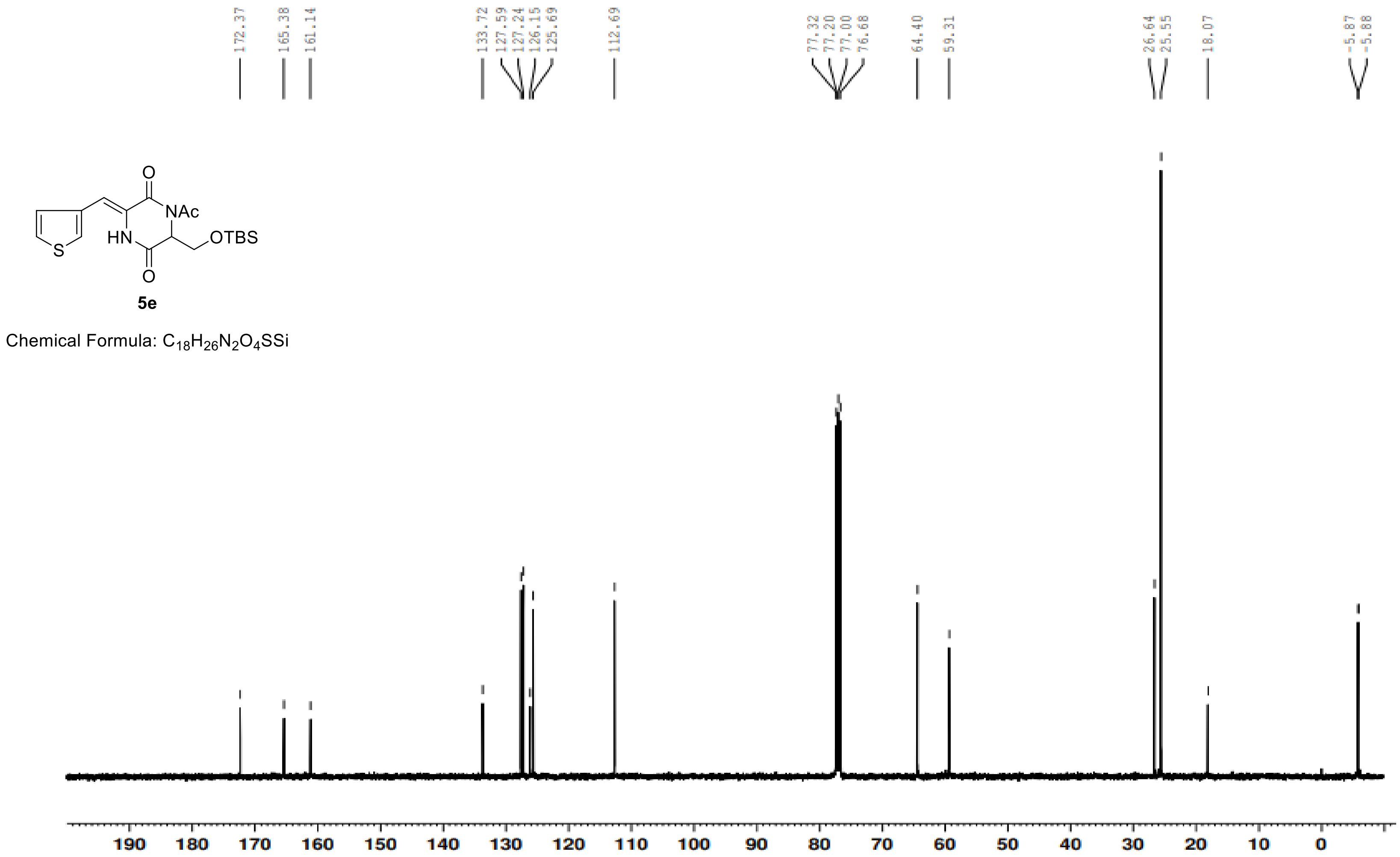

Figure S13. ^{1}H NMR spectrum (400 MHz, $CDCl_3$) of **5f**.

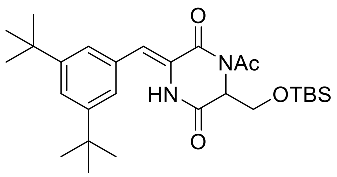

5f

Chemical Formula: $C_{28}H_{44}N_2O_4Si$

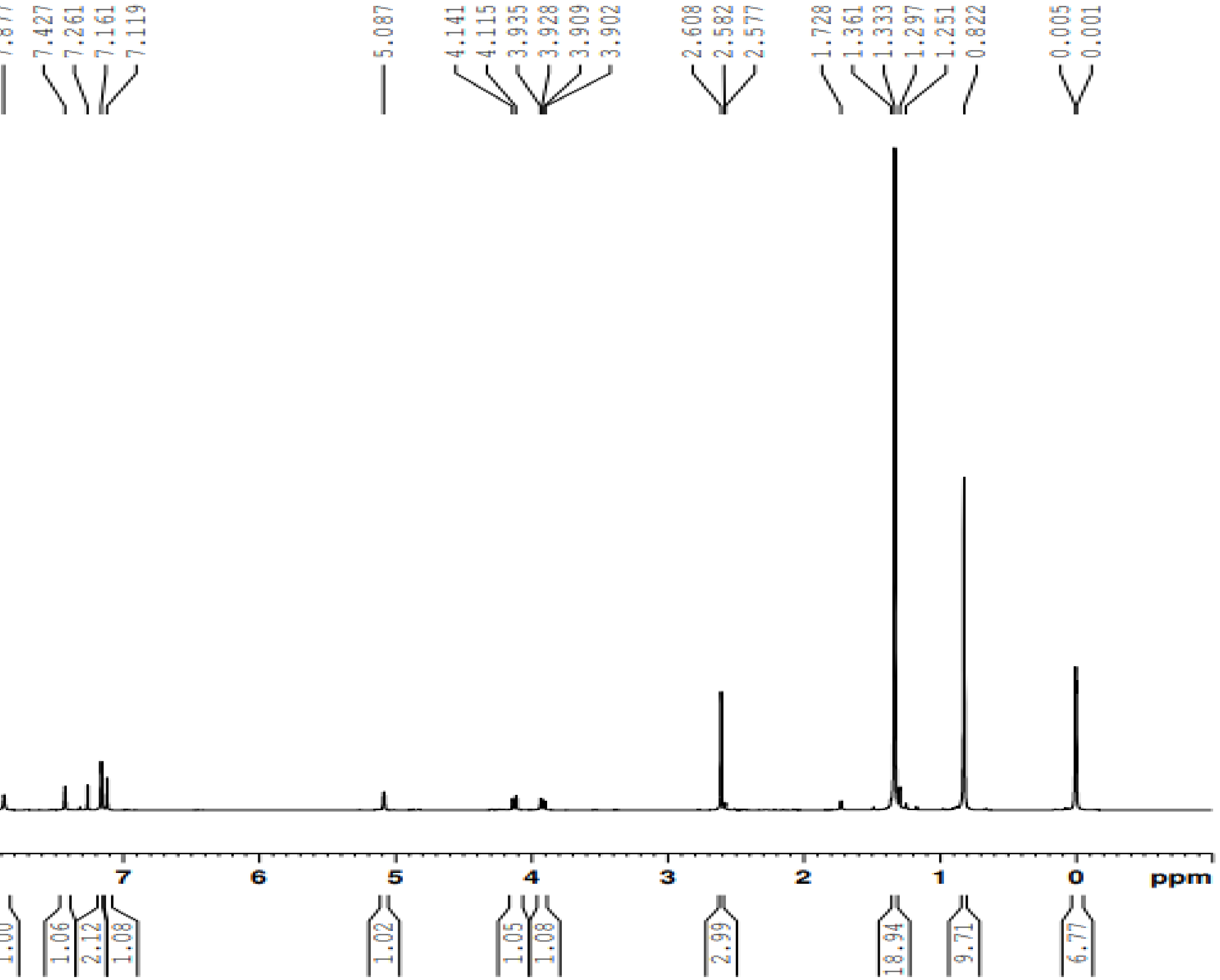

Figure S14. $^{13}C\{^{1}H\}$ NMR spectrum (100 MHz, $CDCl_3$) of **5f**.

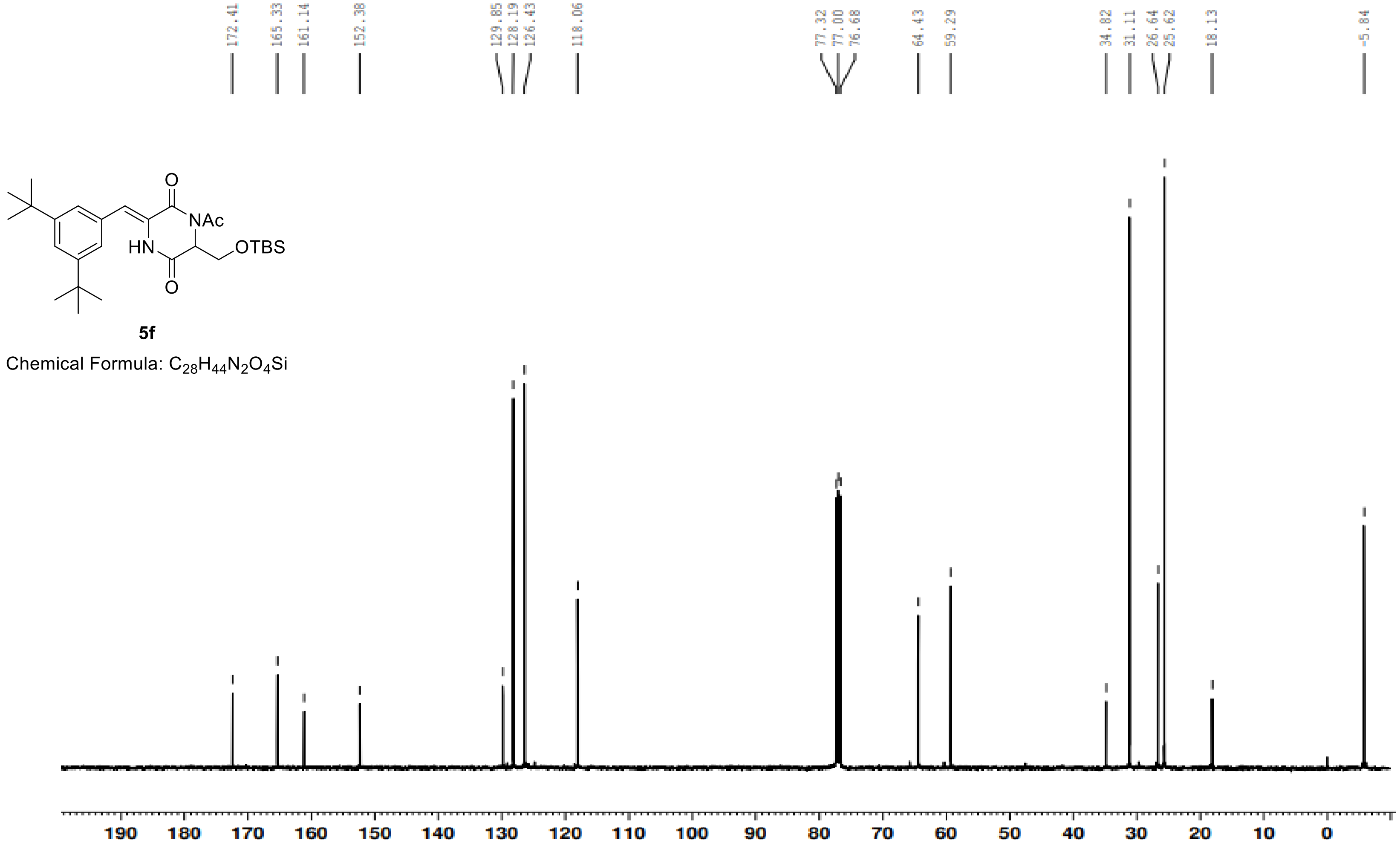

Figure S15. ^{1}H NMR spectrum (400 MHz, $CDCl_3$) of **5g**.

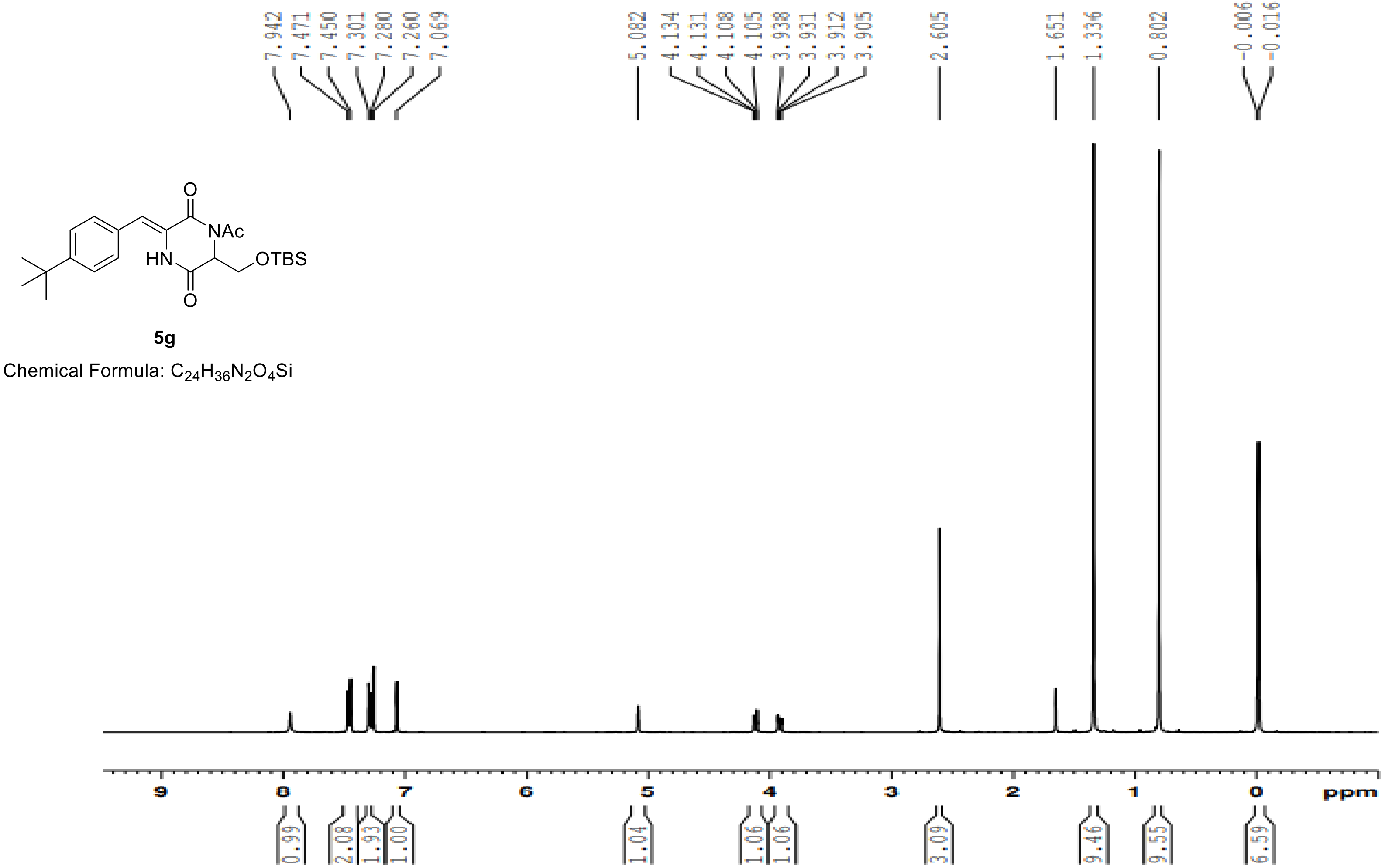

Figure S16. $^{13}C\{^{1}H\}$ NMR spectrum (100 MHz, $CDCl_3$) of **5g**.

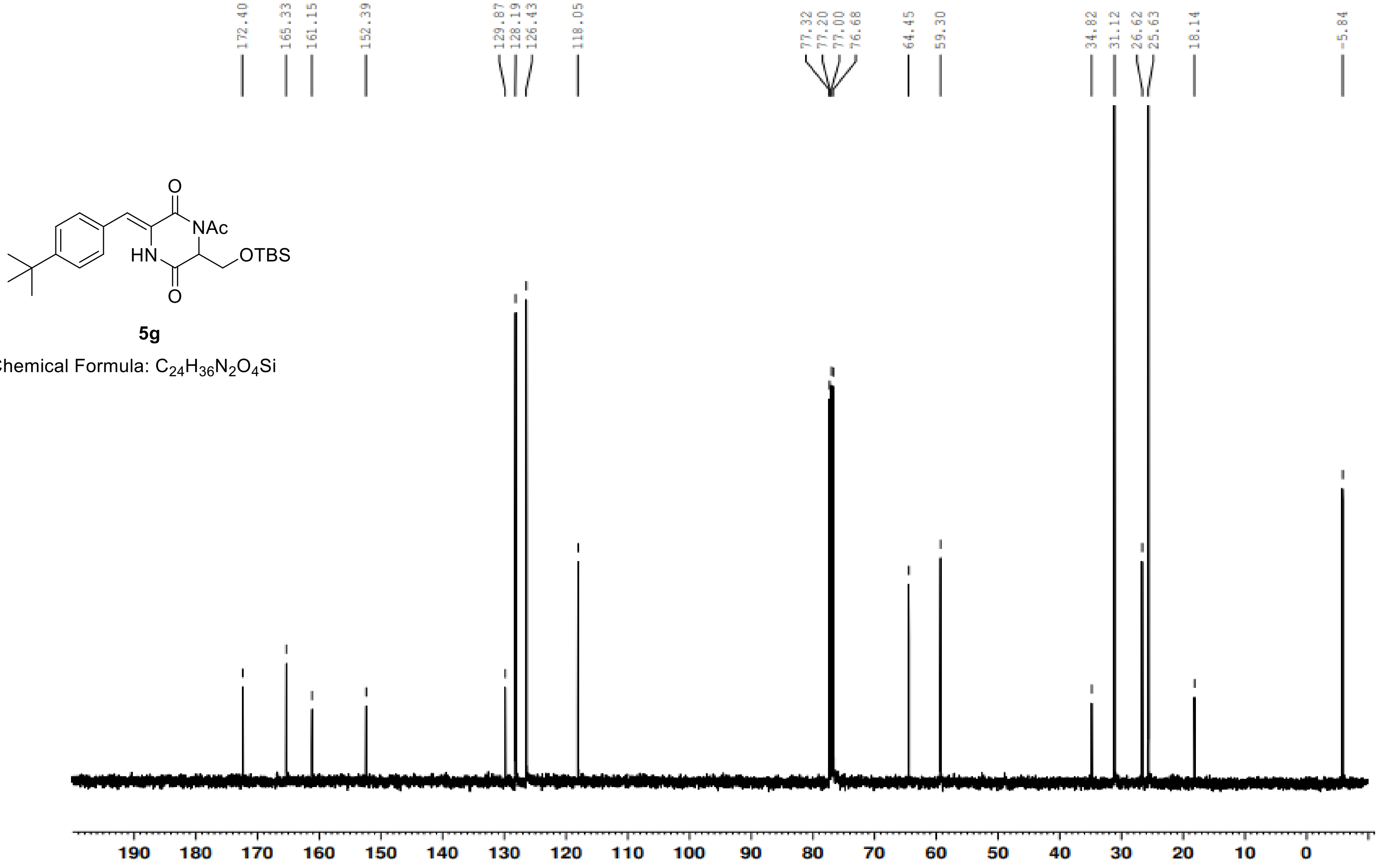

5g

Chemical Formula: $C_{24}H_{36}N_2O_4Si$

Figure S17. ^{1}H NMR spectrum (400 MHz, $CDCl_3$) of **5h**.

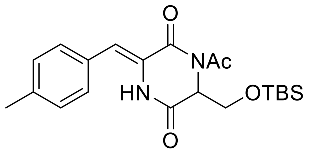

5h

Chemical Formula: $C_{21}H_{30}N_2O_4Si$

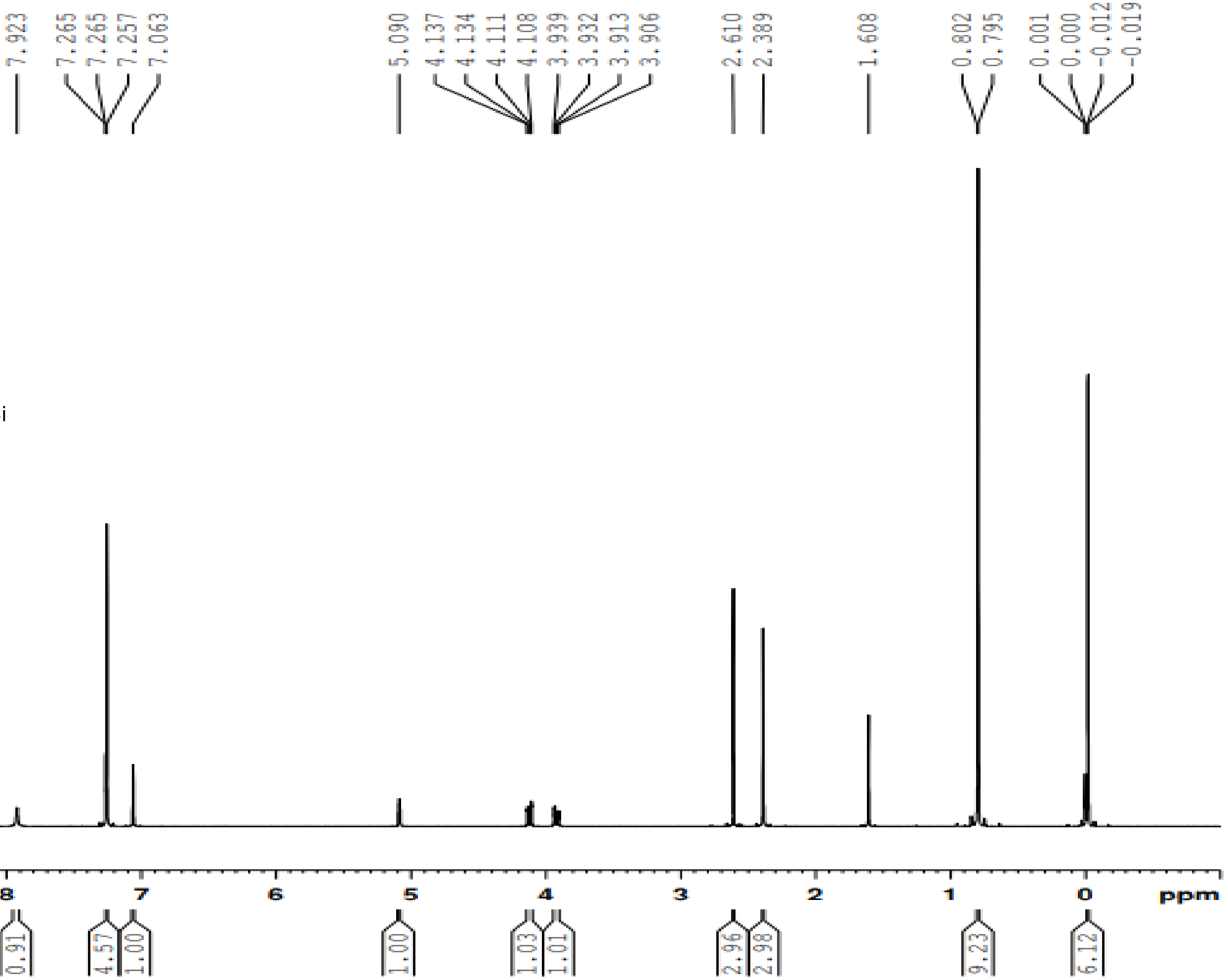

Figure S18. $^{13}C\{^{1}H\}$ NMR spectrum (100 MHz, $CDCl_3$) of **5h**.

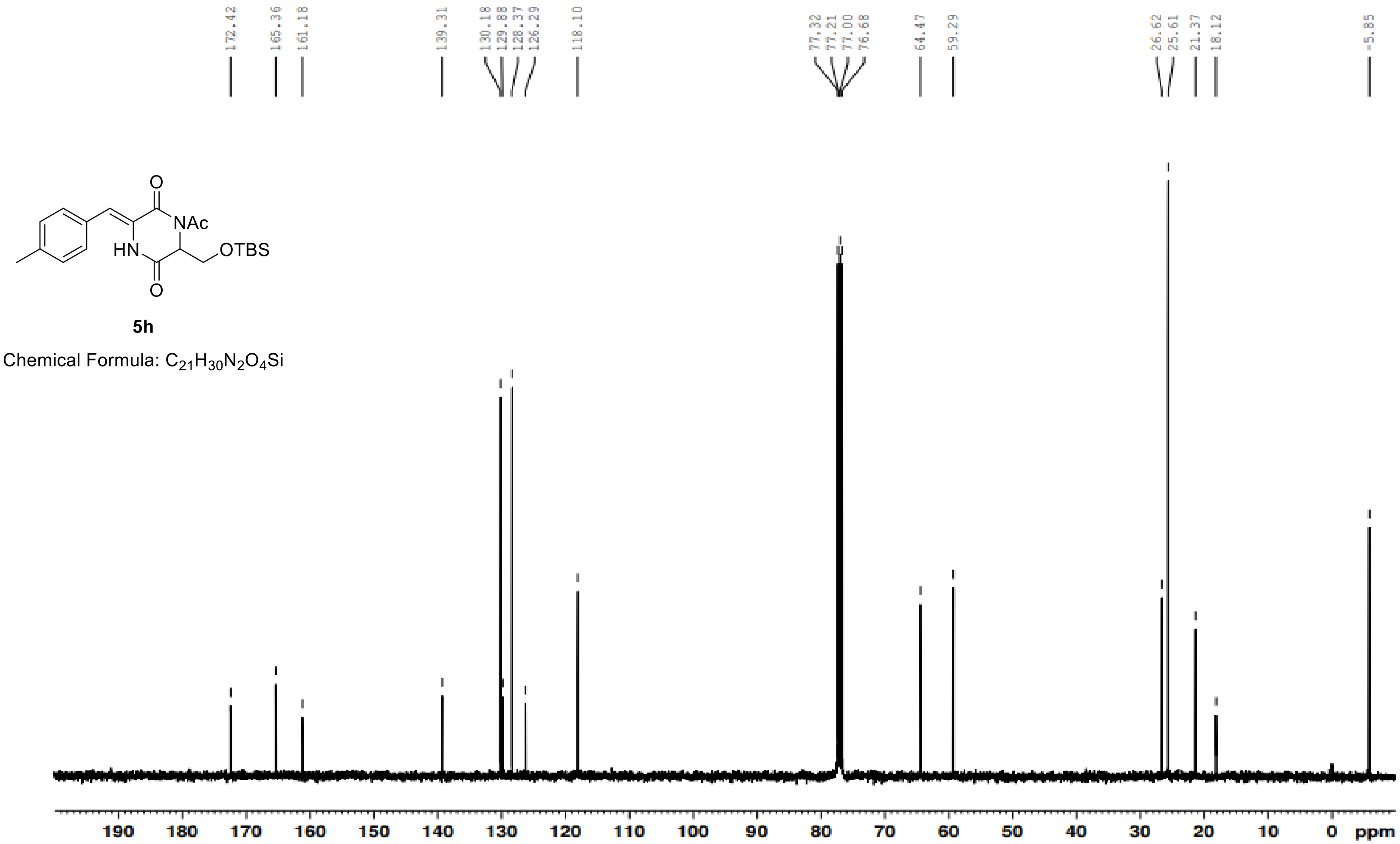

Figure S19. ^{1}H NMR spectrum (400 MHz, $CDCl_3$) of **5i**.

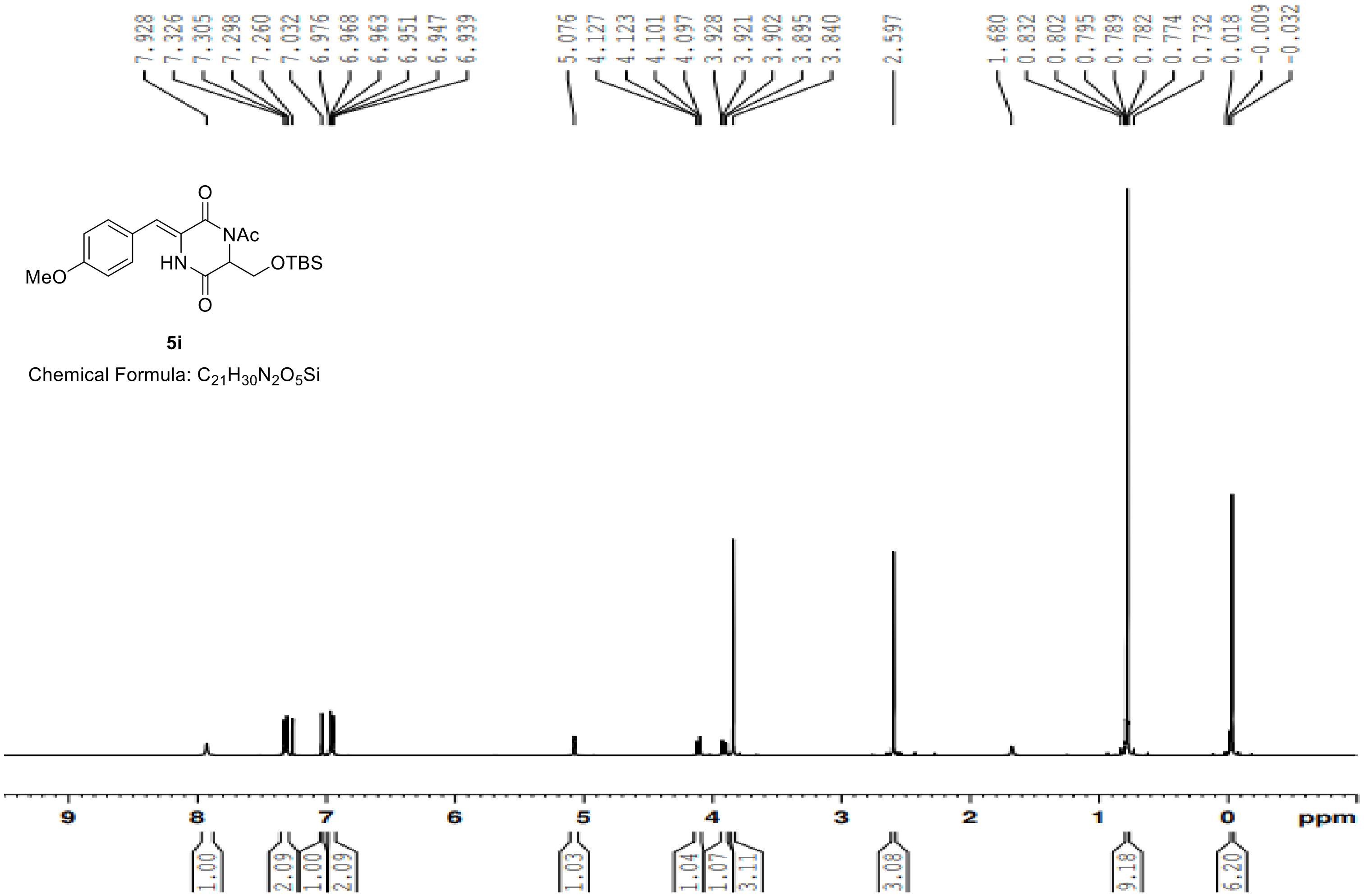

Figure S20. $^{13}C\{^{1}H\}$ NMR spectrum (100 MHz, $CDCl_3$) of **5i**.

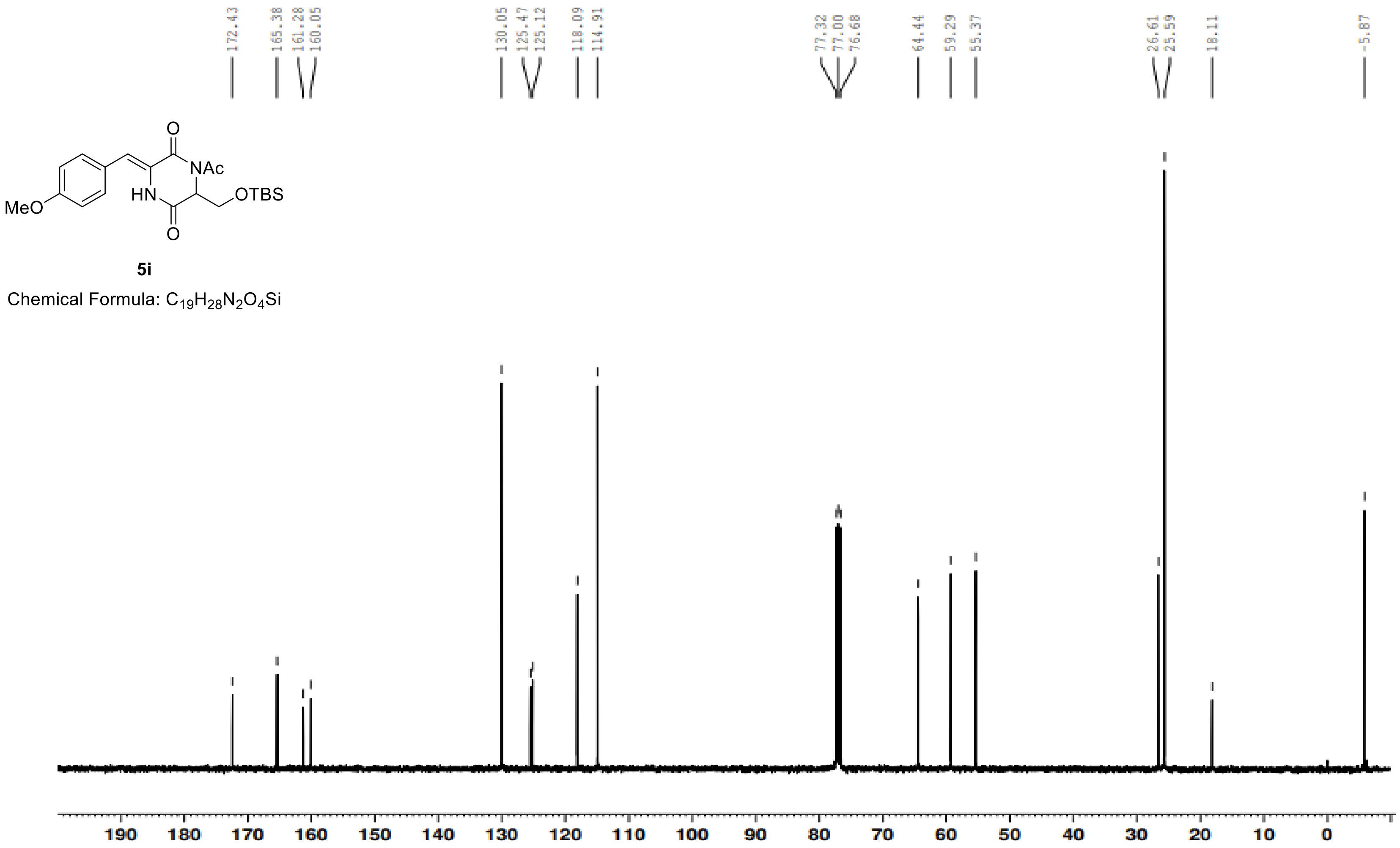

Figure S21. ^{1}H NMR spectrum (400 MHz, $CDCl_3$) of **5j**.

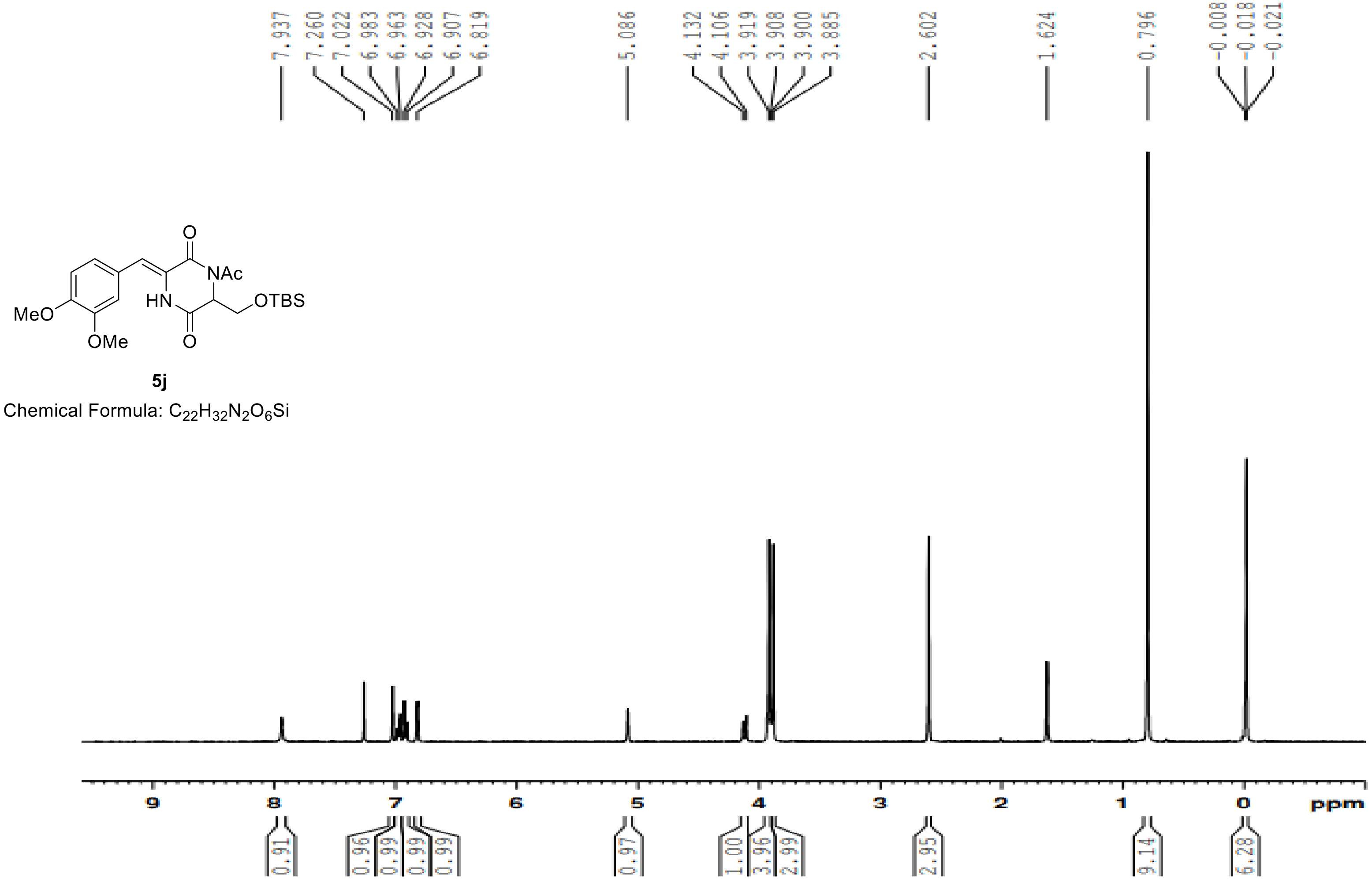

Figure S22. $^{13}C\{^{1}H\}$ NMR spectrum (100 MHz, $CDCl_3$) of **5j**.

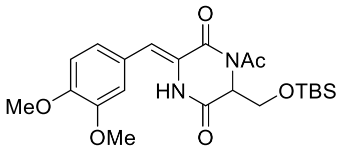

5j

Chemical Formula: $C_{22}H_{32}N_2O_6Si$

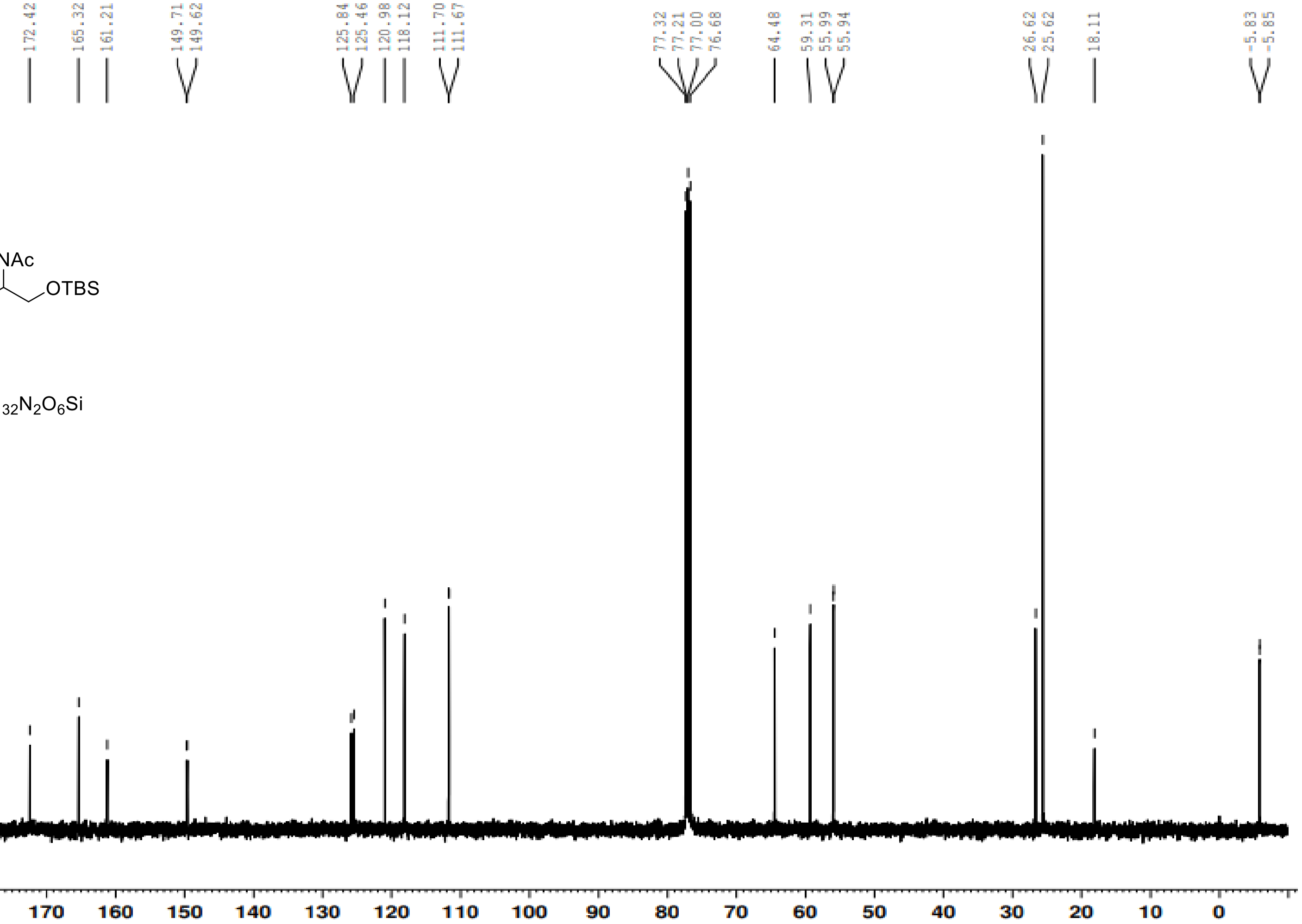

Figure S23. ^{1}H NMR spectrum (400 MHz, $CDCl_3$) of **5k**.

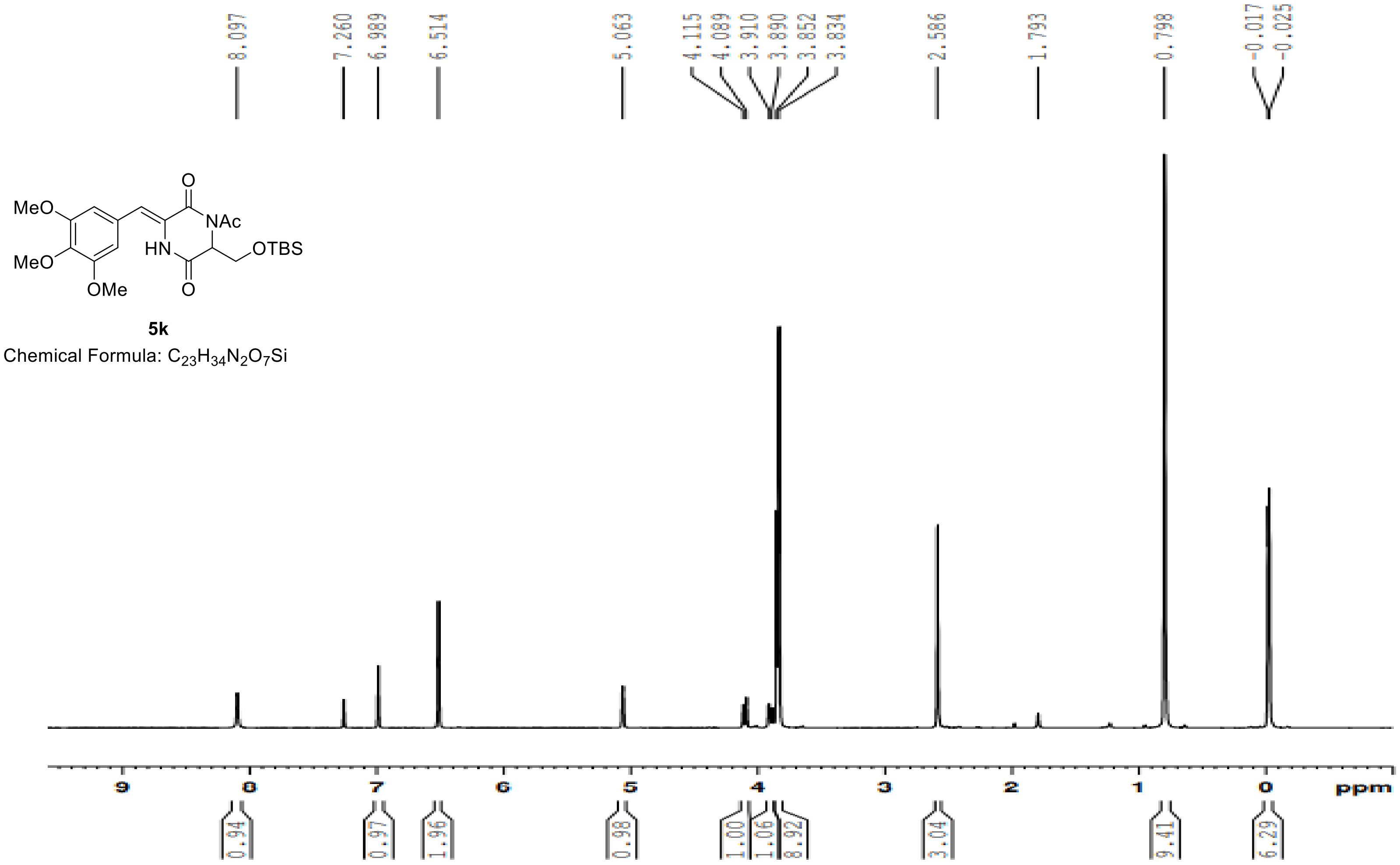

Figure S24. $^{13}C\{^{1}H\}$ NMR spectrum (100 MHz, $CDCl_3$) of **5k**.

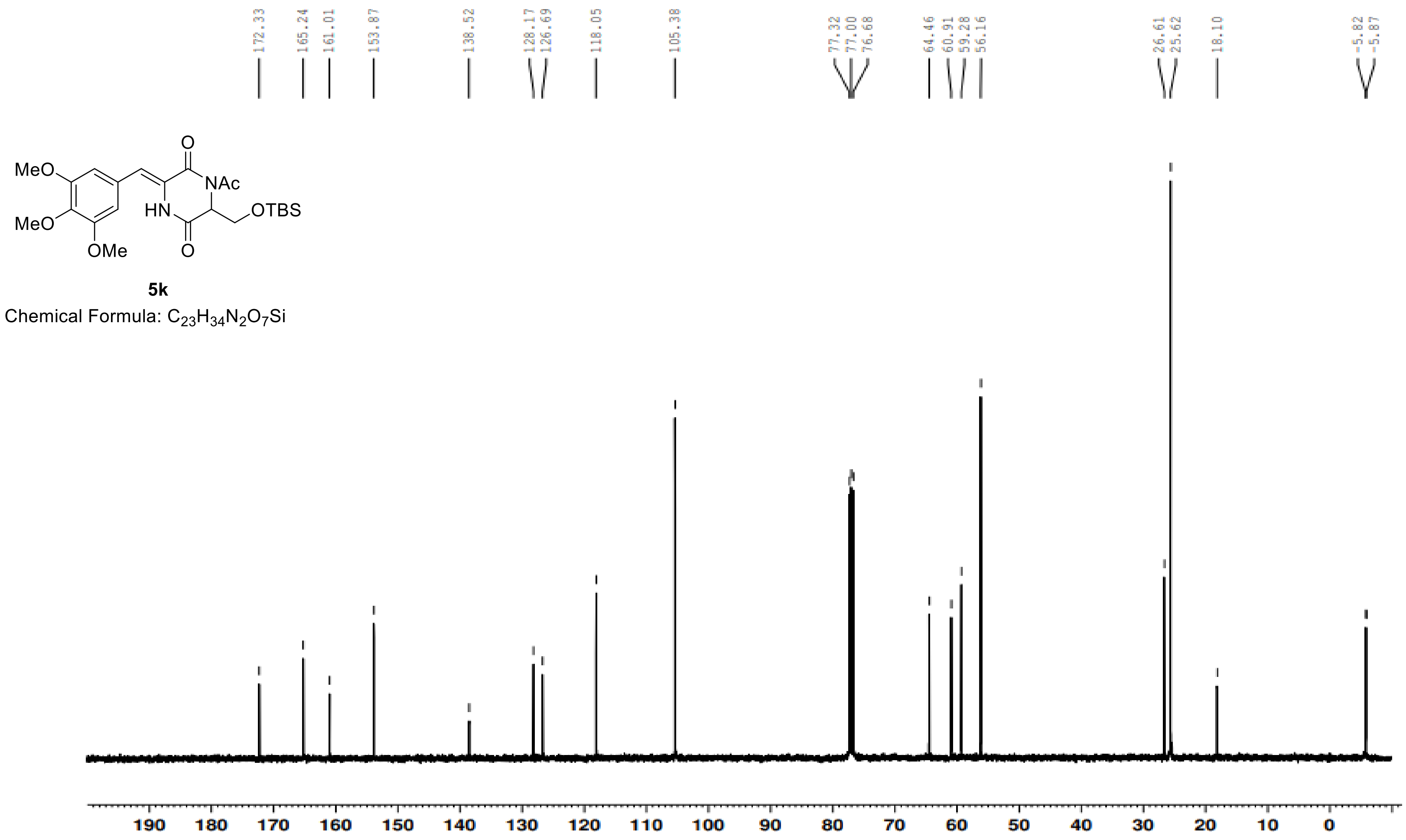

Figure S25. ^{1}H NMR spectrum (400 MHz, $CDCl_3$) of ***5l***.

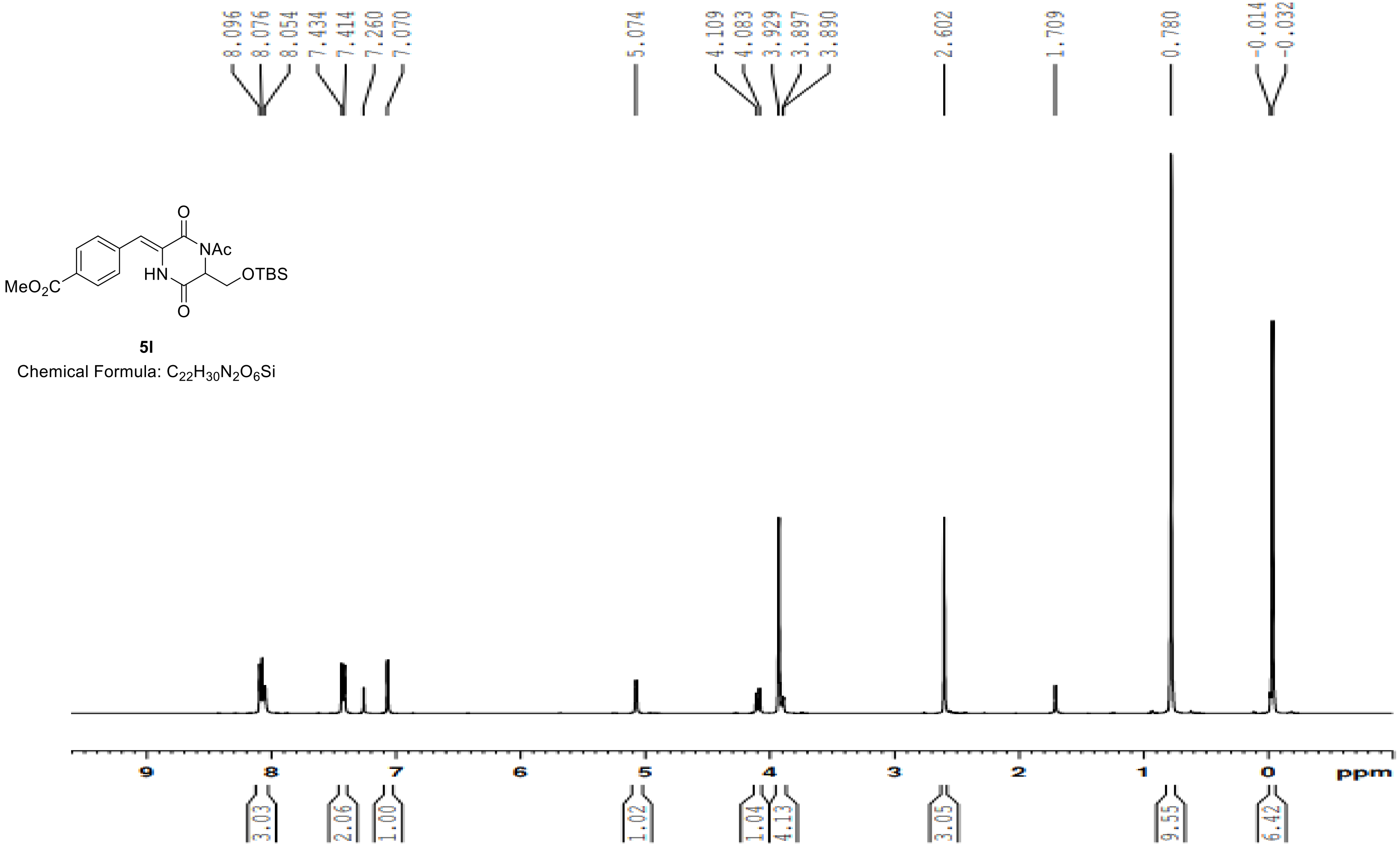

5l

Chemical Formula: $C_{22}H_{30}N_2O_6Si$

Figure S26. $^{13}C\{^{1}H\}$ NMR spectrum (100 MHz, $CDCl_3$) of ***5l***.

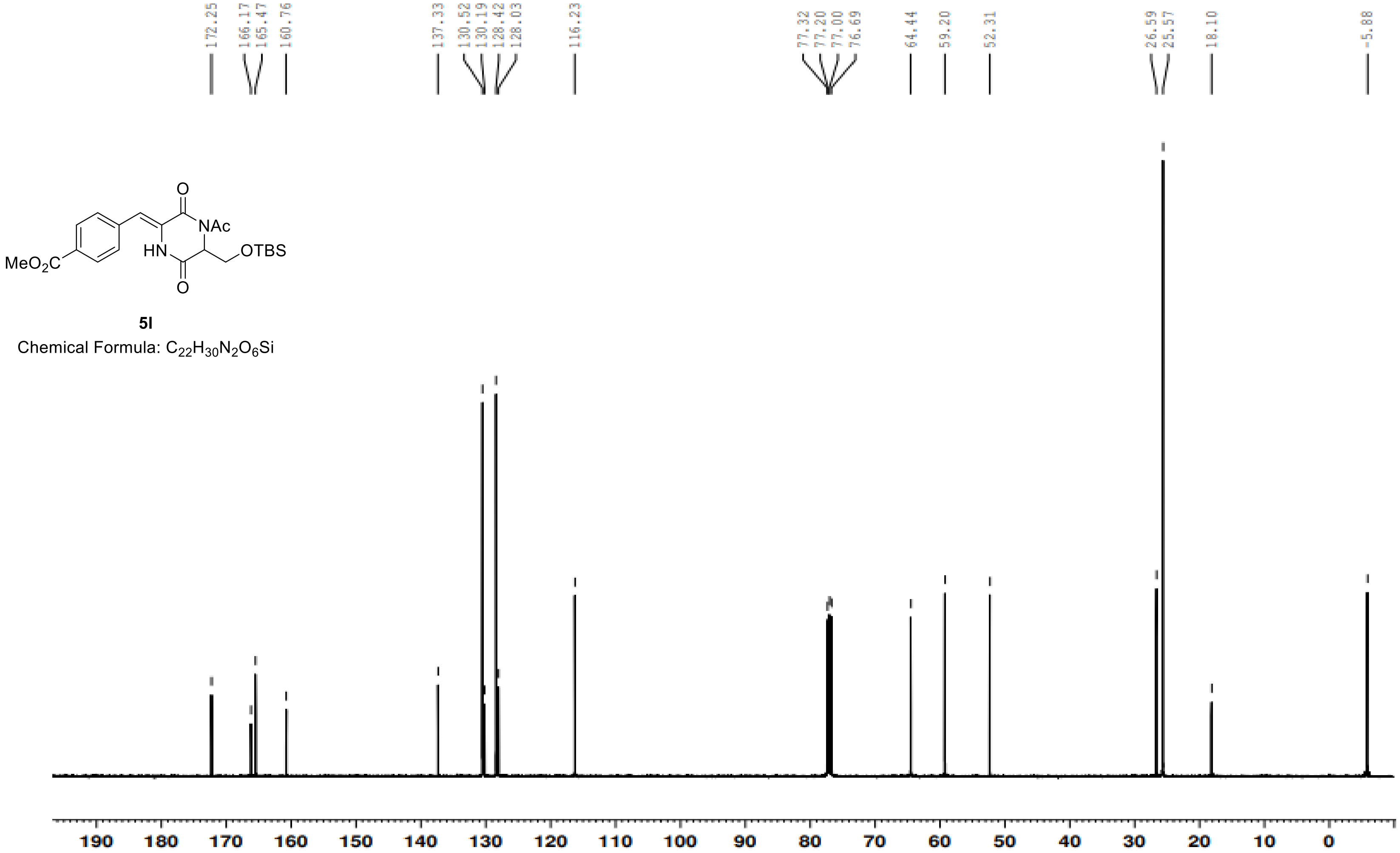

Figure S27. ^{1}H NMR spectrum (400 MHz, $CDCl_3$) of **5m**.

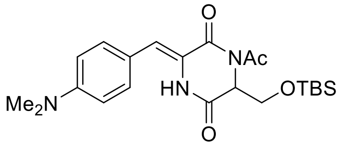

5m

Chemical Formula: $C_{22}H_{33}N_3O_4Si$

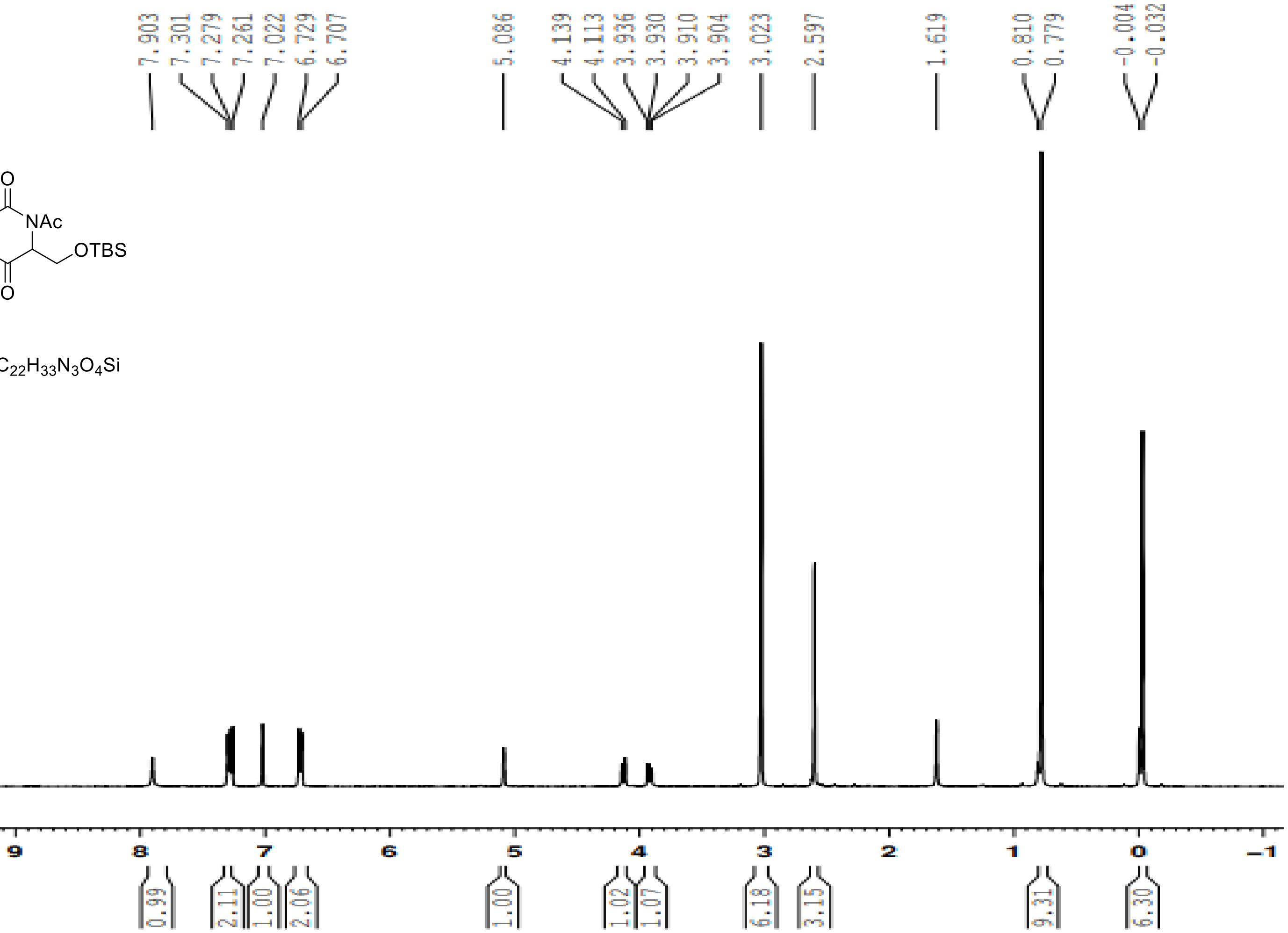

Figure S28. $^{13}C\{^{1}H\}$ NMR spectrum (100 MHz, $CDCl_3$) of **5m**.

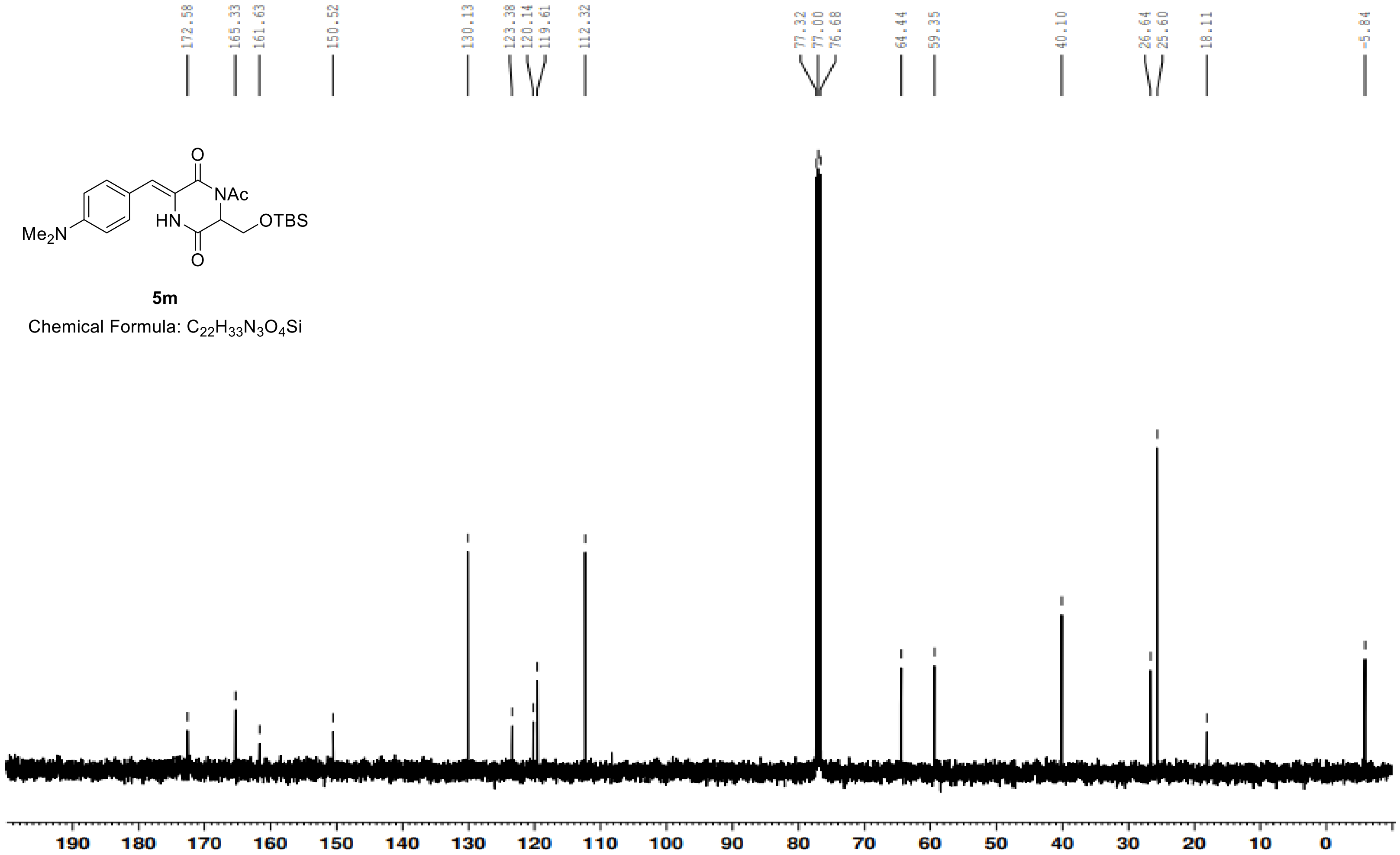

Figure S29. ^{1}H NMR spectrum (400 MHz, $CDCl_3$) of **5n**.

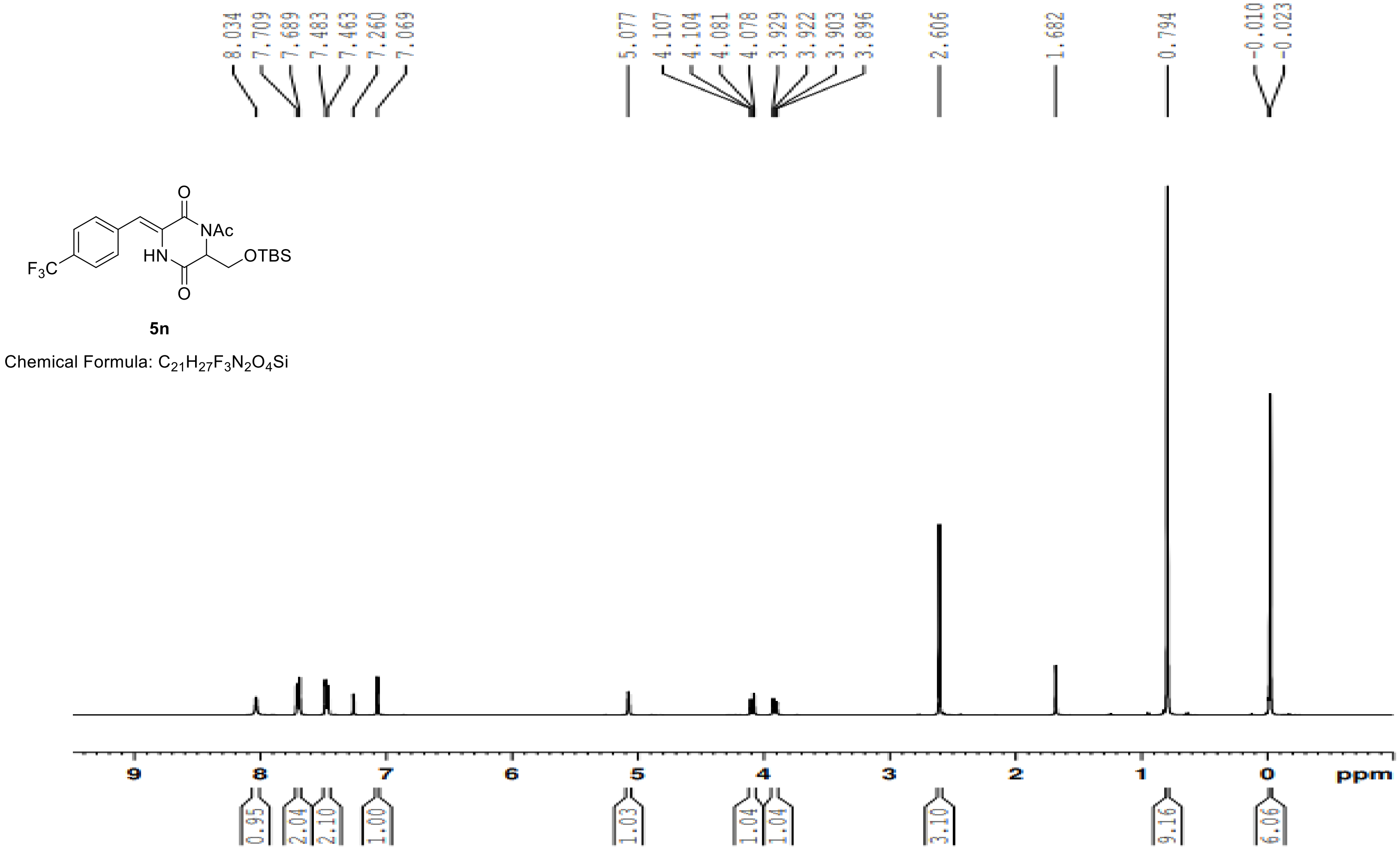

Figure S30. $^{13}C\{^1H\}$ NMR spectrum (100 MHz, $CDCl_3$) of **5n**.

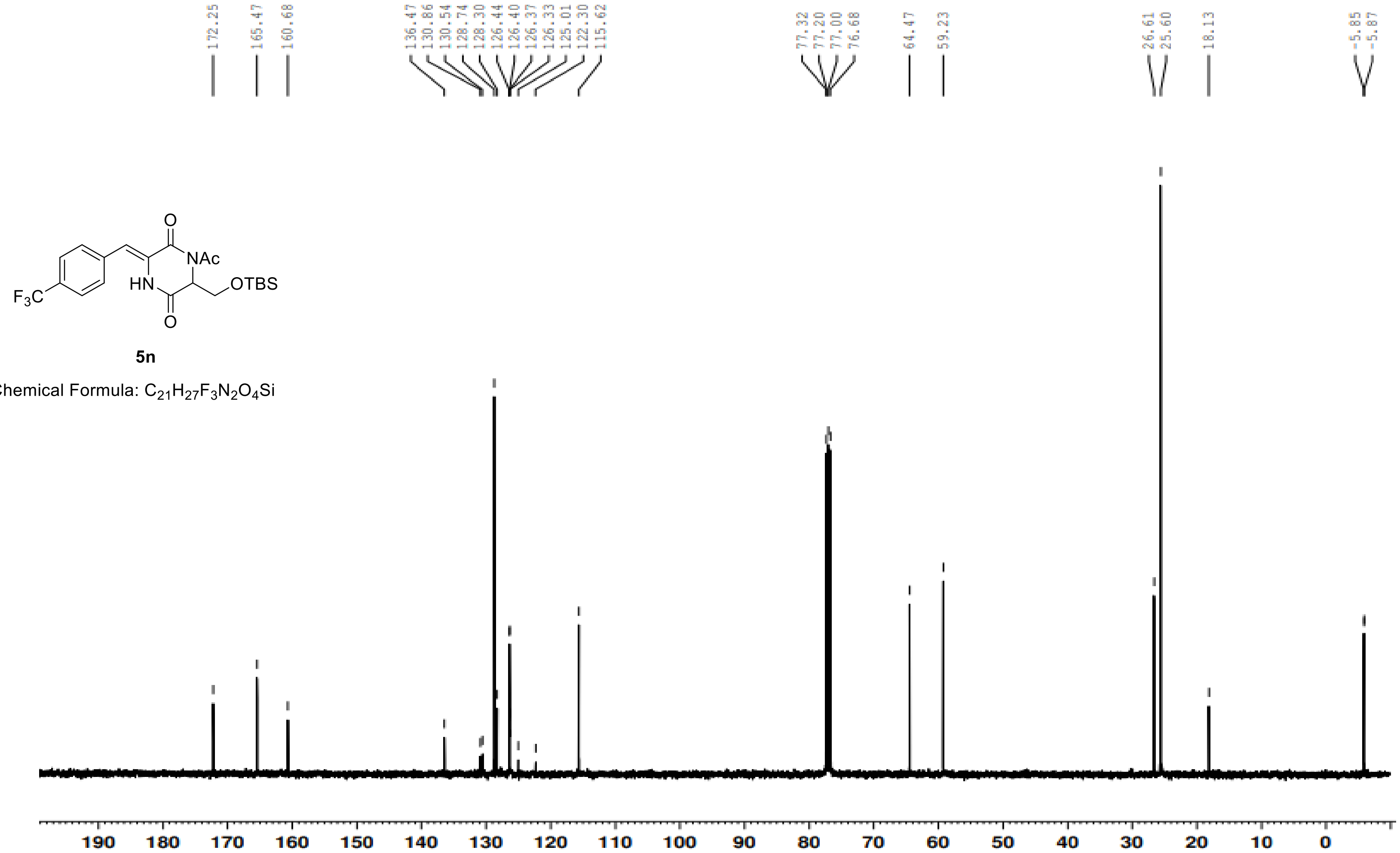

5n

Chemical Formula: $C_{21}H_{27}F_3N_2O_4Si$

Figure S31. ^{1}H NMR spectrum (400 MHz, $CDCl_3$) of **5o**.

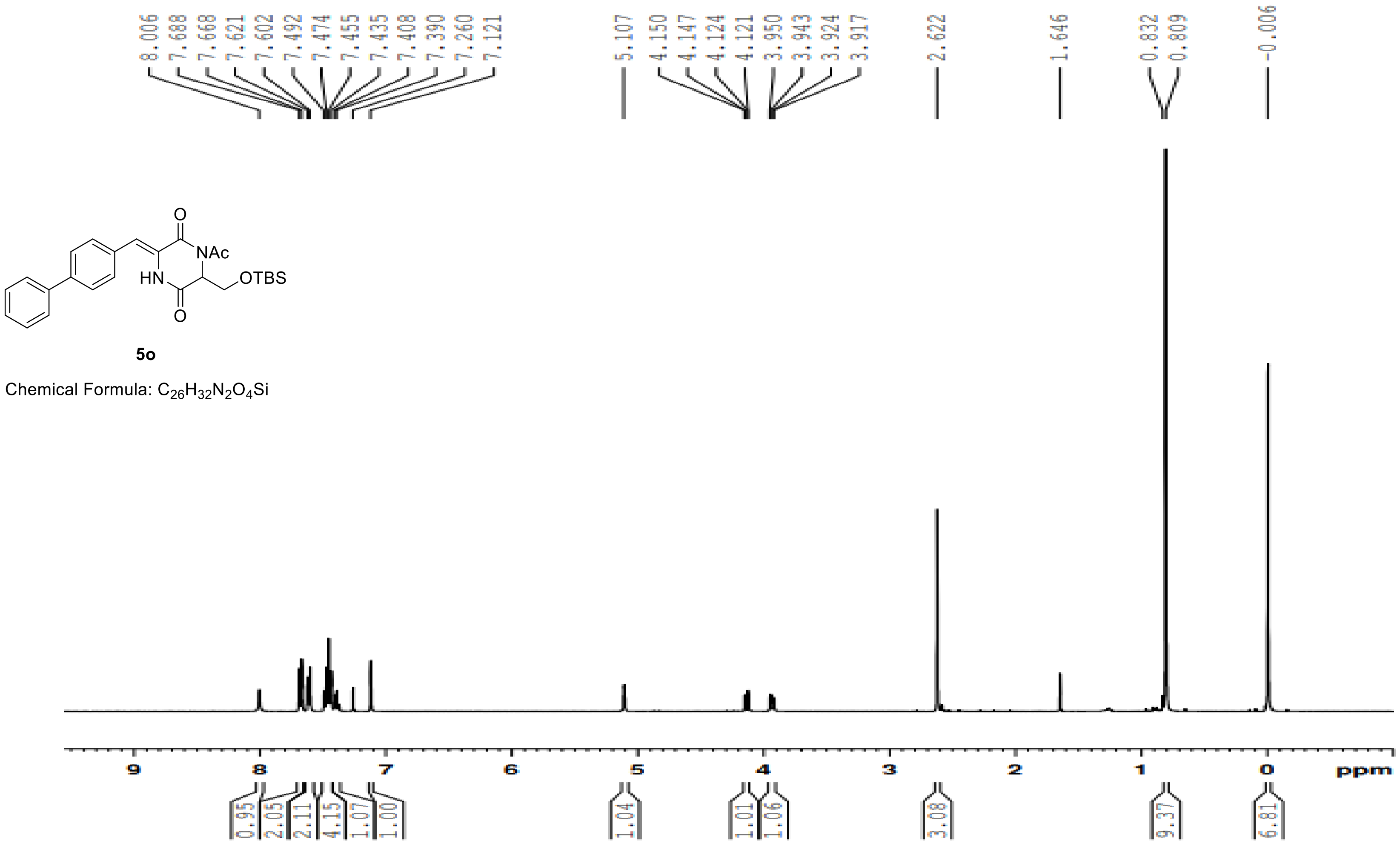

5o

Chemical Formula: $C_{26}H_{32}N_2O_4Si$

Figure S32. $^{13}C\{^1H\}$ NMR spectrum (100 MHz, $CDCl_3$) of **5o**.

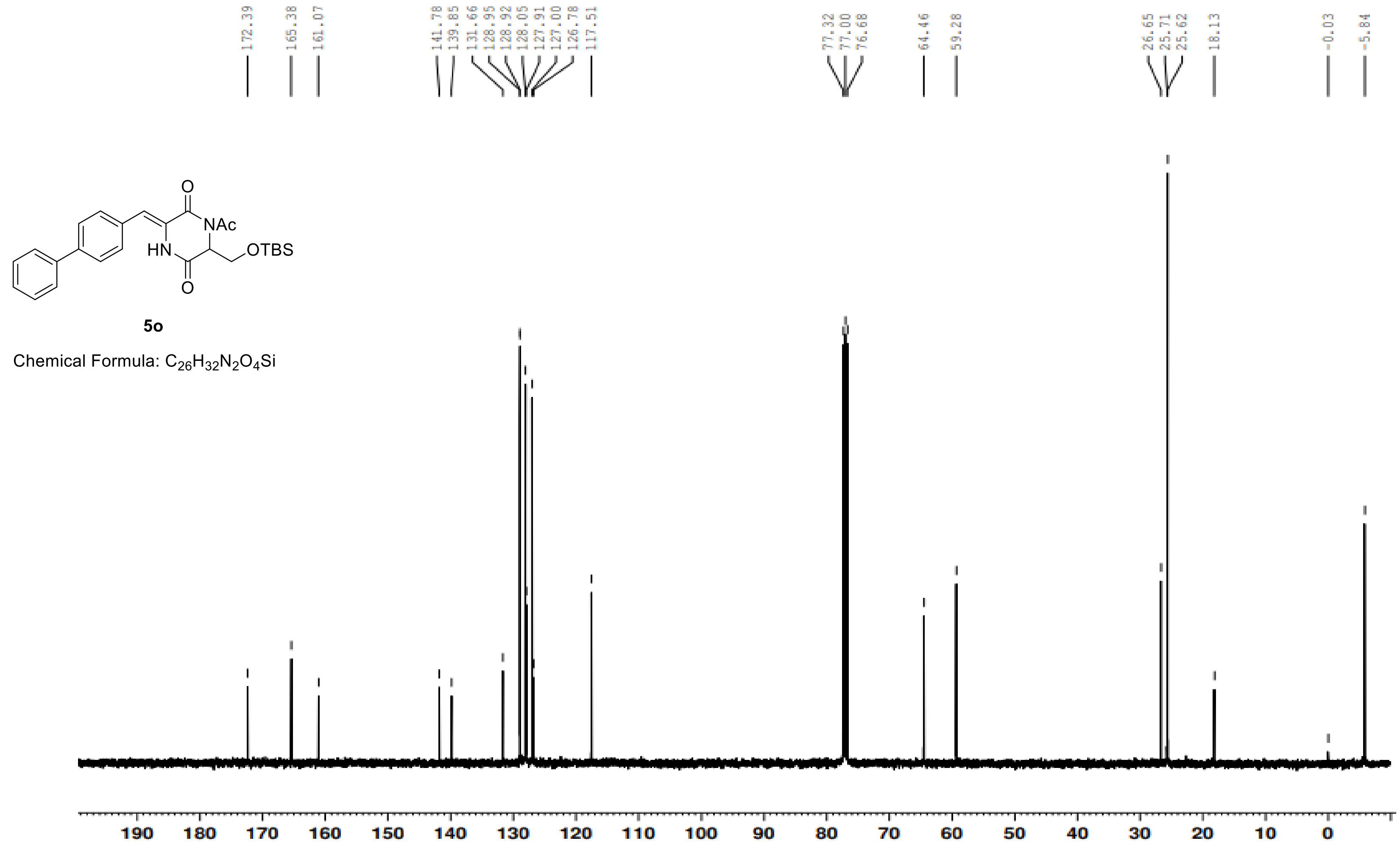

Figure S33. ^{1}H NMR spectrum (400 MHz, $CDCl_3$) of **5p**.

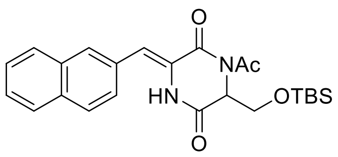

5p

Chemical Formula: $C_{24}H_{30}N_2O_4Si$

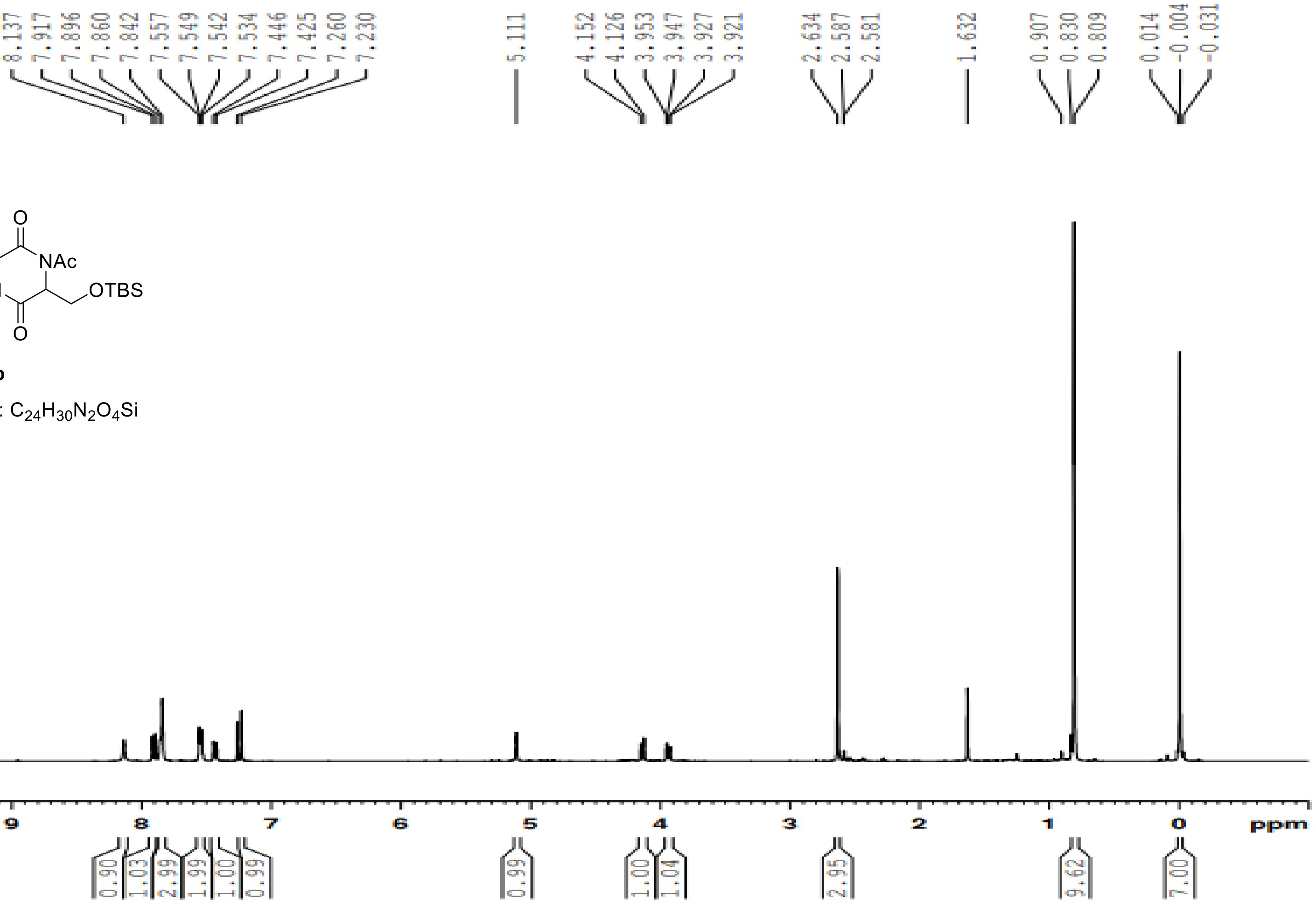

Figure S34. $^{13}C\{^{1}H\}$ NMR spectrum (100 MHz, CDCl$_3$) of **5p**.

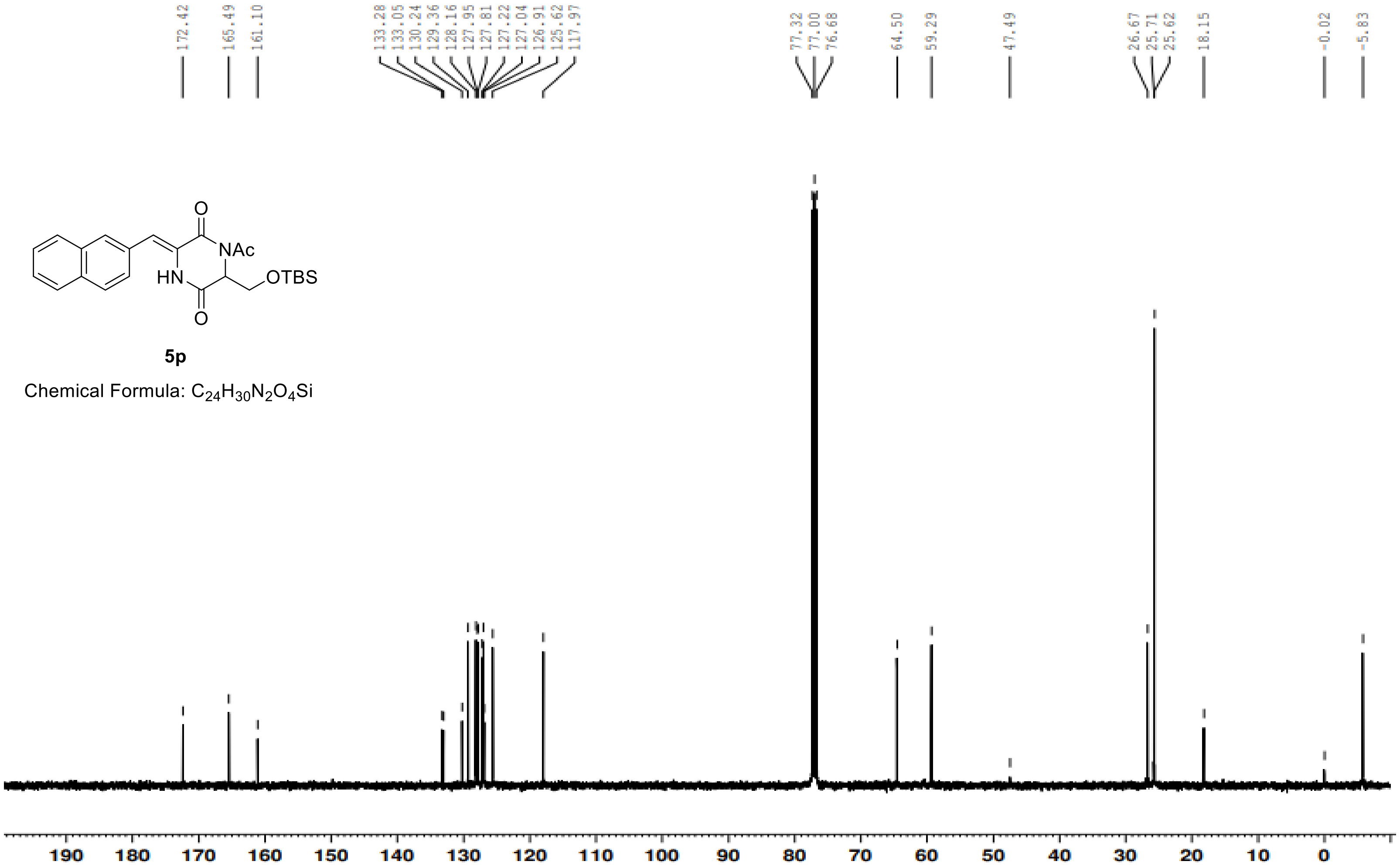

Figure S35. ^{1}H NMR spectrum (400 MHz, $CDCl_3$) of **5q**.

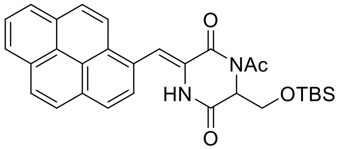

5q

Chemical Formula: $C_{30}H_{32}N_2O_4Si$

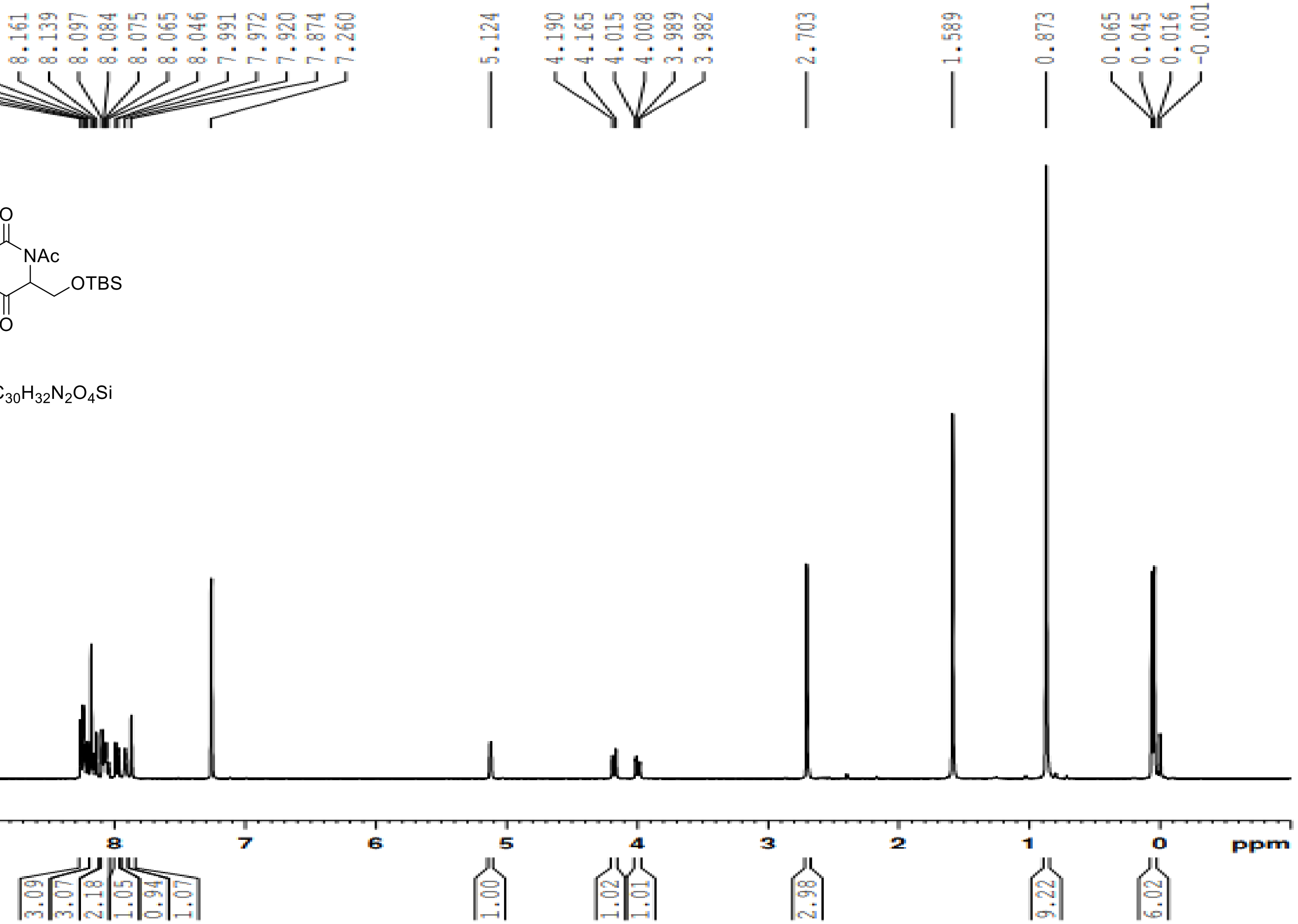

Figure S36. $^{13}C\{^{1}H\}$ NMR spectrum (100 MHz, $CDCl_3$) of **5q**.

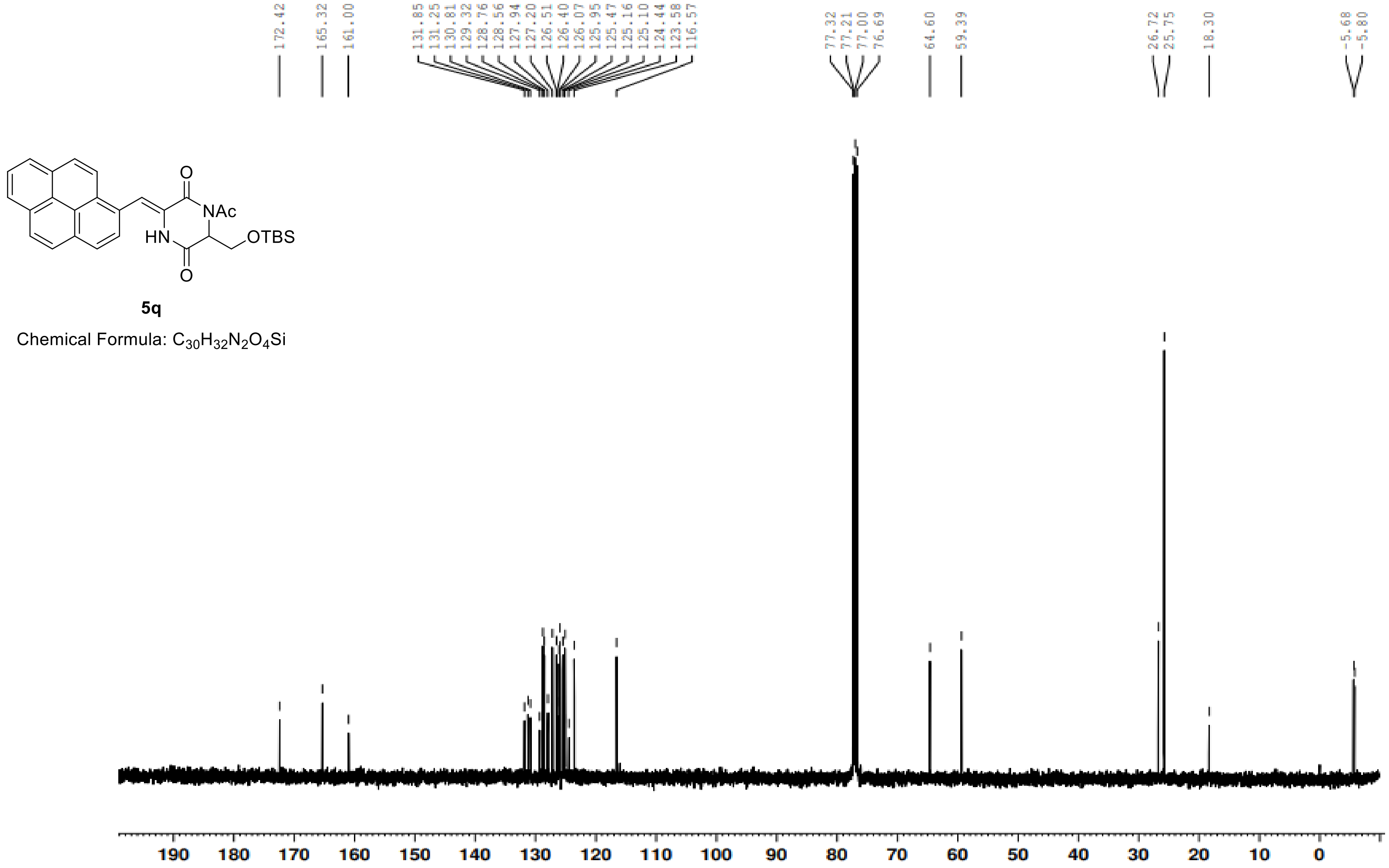

Figure S37. ^{1}H NMR spectrum (400 MHz, $CDCl_3$) of **5s**.

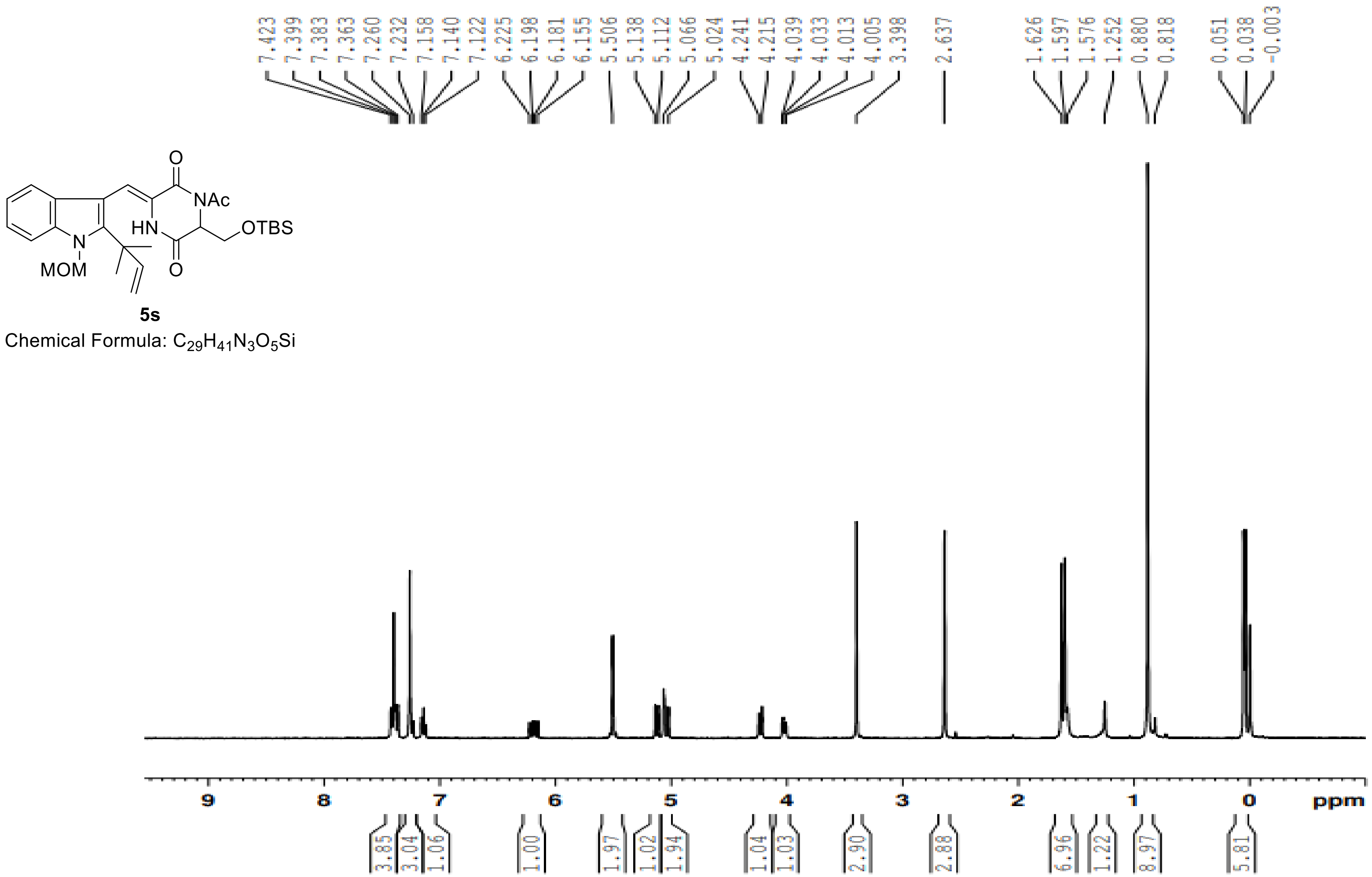

Figure S38. $^{13}C\{^{1}H\}$ NMR spectrum (100 MHz, $CDCl_3$) of **5s**.

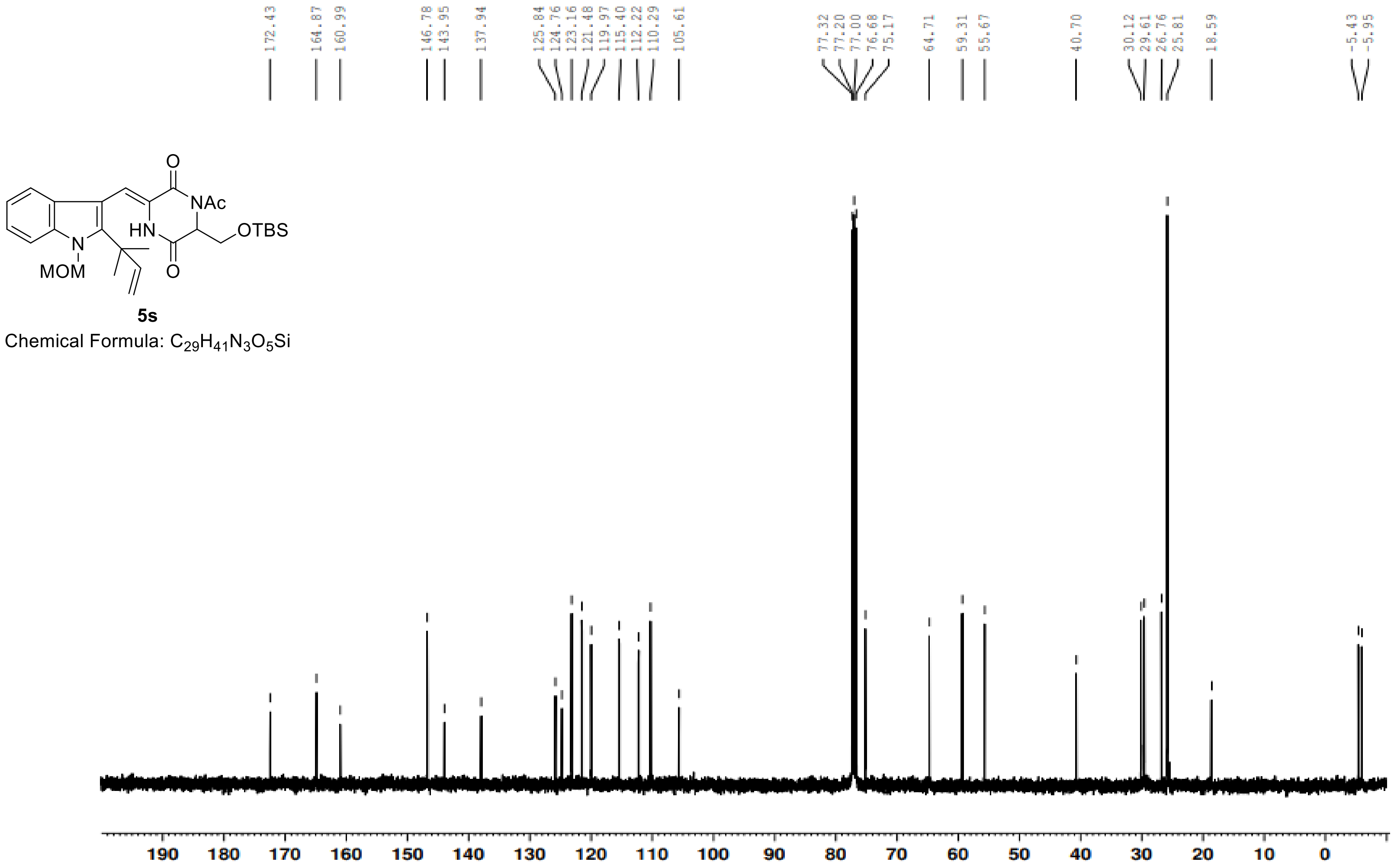

5s

Chemical Formula: $C_{29}H_{41}N_3O_5Si$

Figure S39. ^{1}H NMR spectrum (400 MHz, $CDCl_3$) of **1a**.

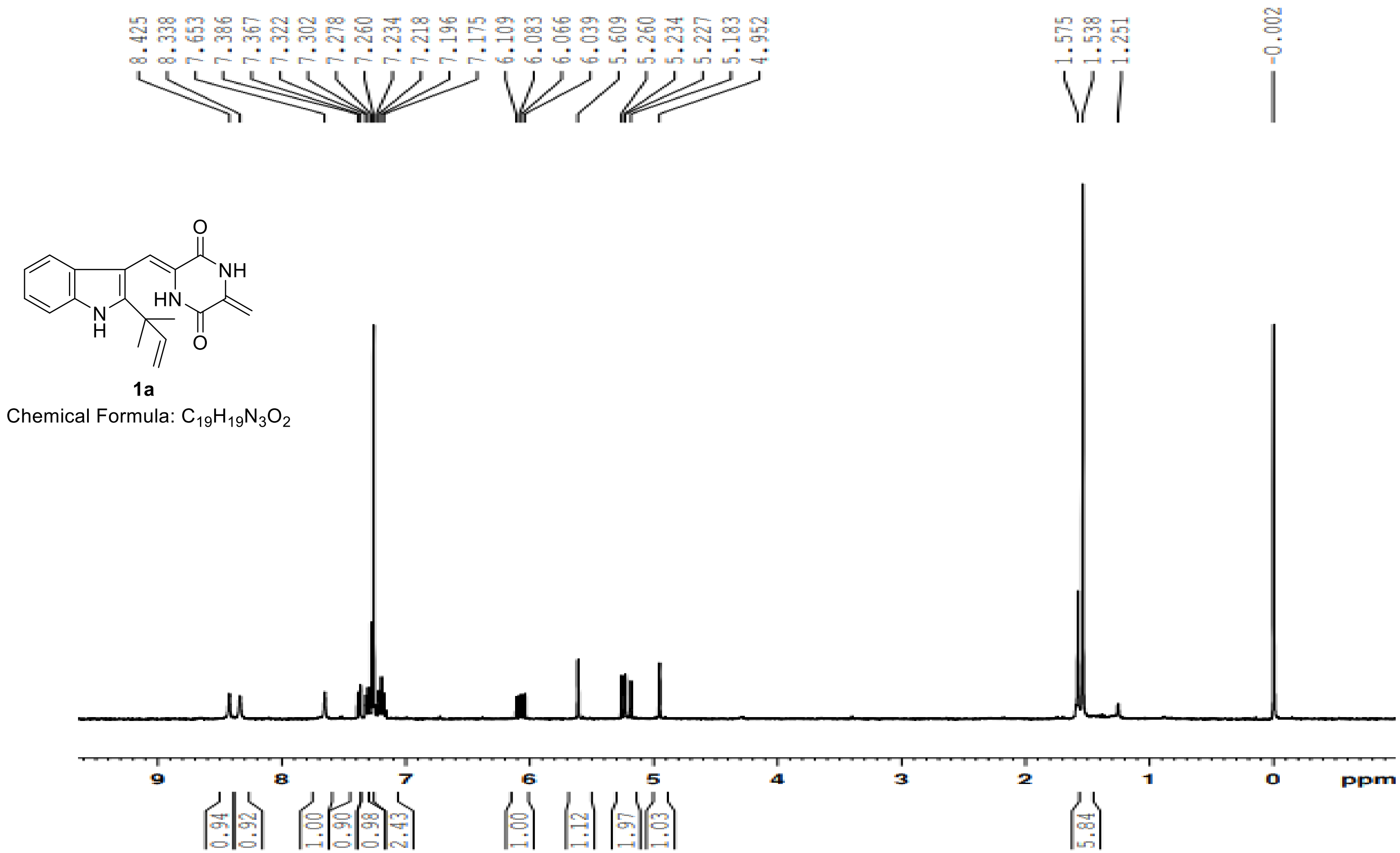

Figure S40. $^{13}C\{^{1}H\}$ NMR spectrum (100 MHz, $CDCl_3$) of **1a**.

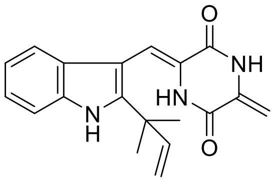

1a

Chemical Formula: $C_{19}H_{19}N_3O_2$

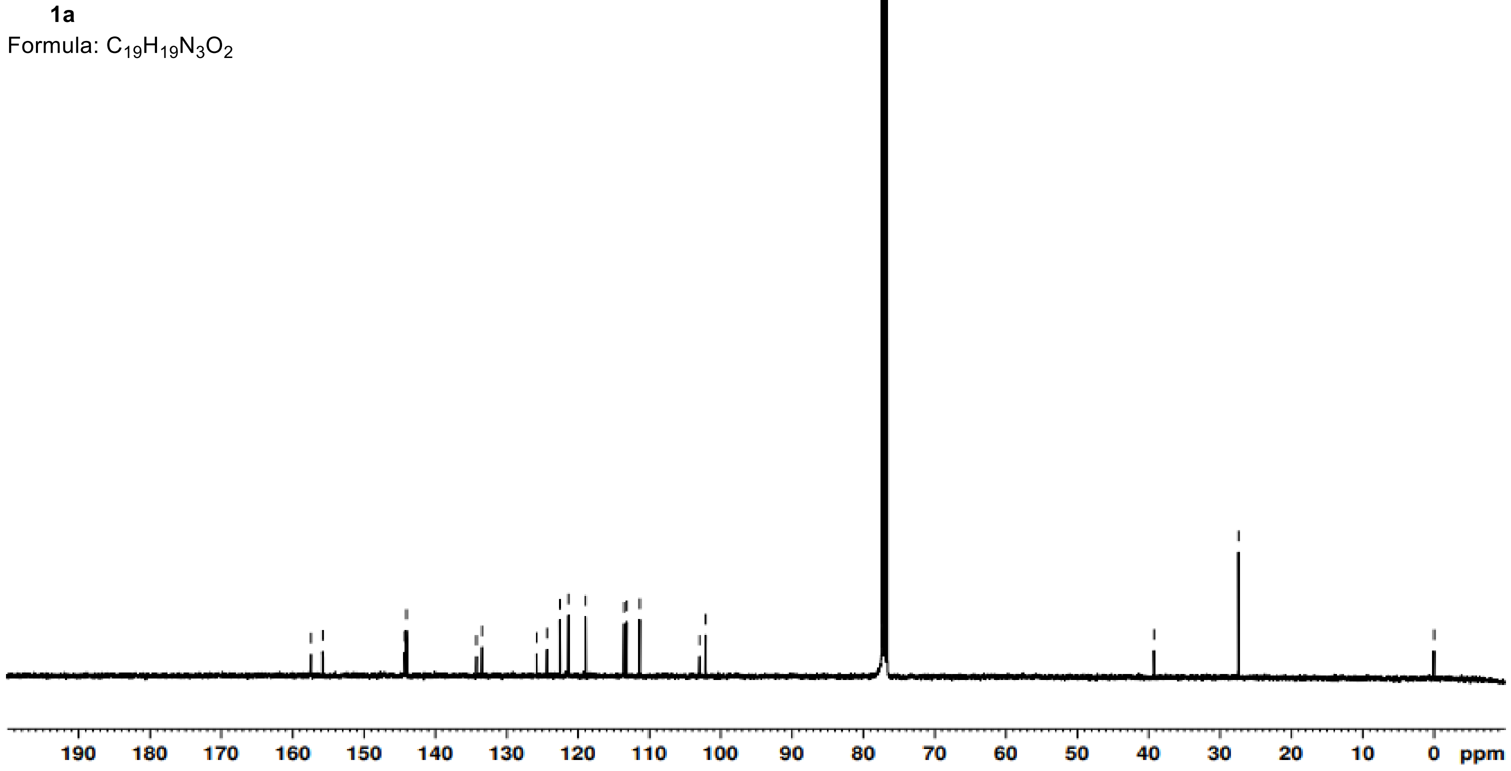

Figure S41. ^{1}H NMR spectrum (400 MHz, DMSO-d_6) of **1b**.

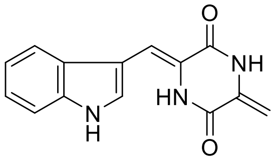

1b

Chemical Formula: $C_{14}H_{11}N_3O_2$

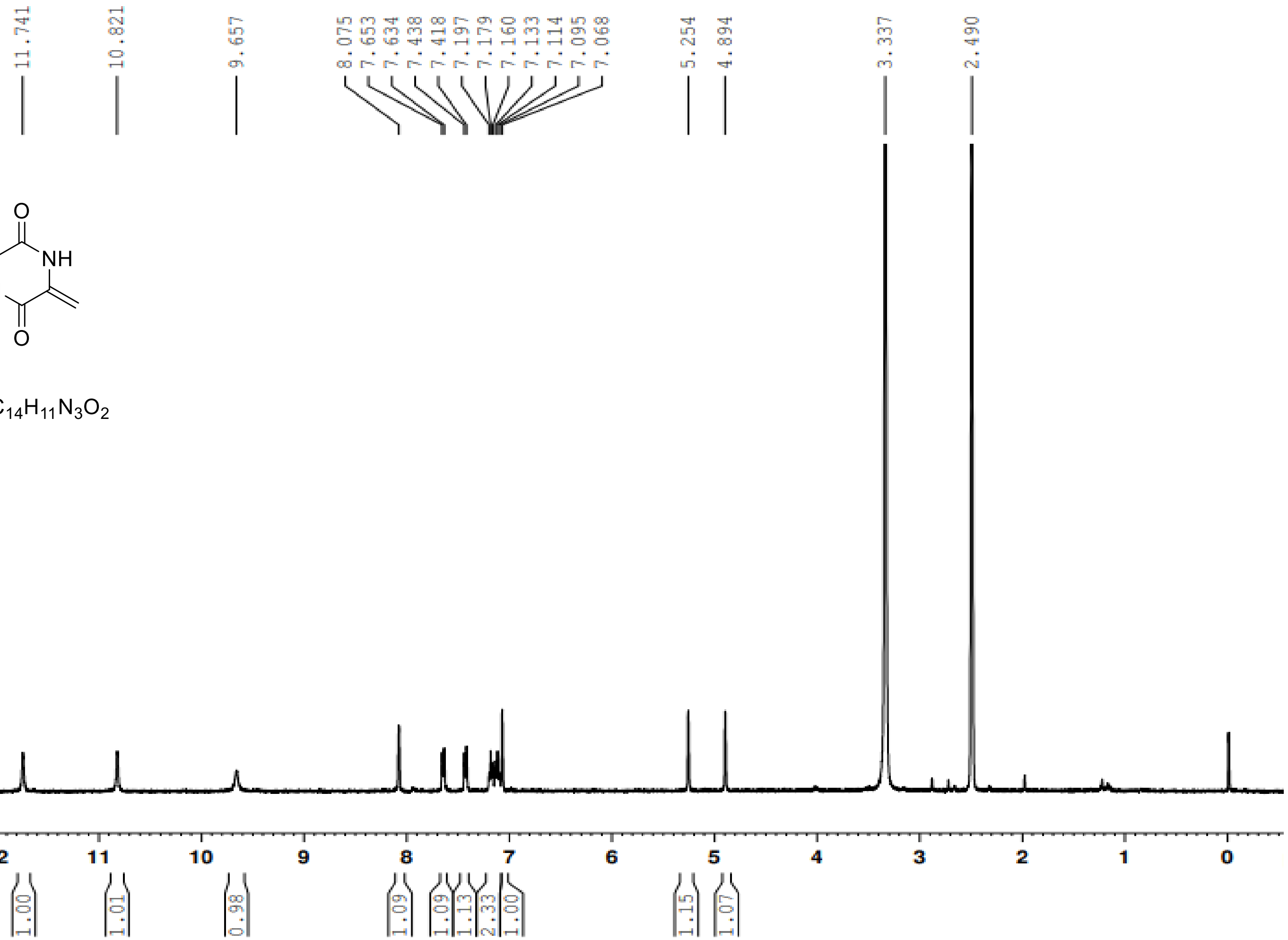

Figure S42. $^{13}C\{^{1}H\}$ NMR spectrum (100 MHz, DMSO-d_6) of **1b**.

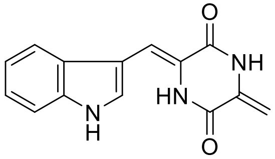

1b

Chemical Formula: $C_{14}H_{11}N_3O_2$

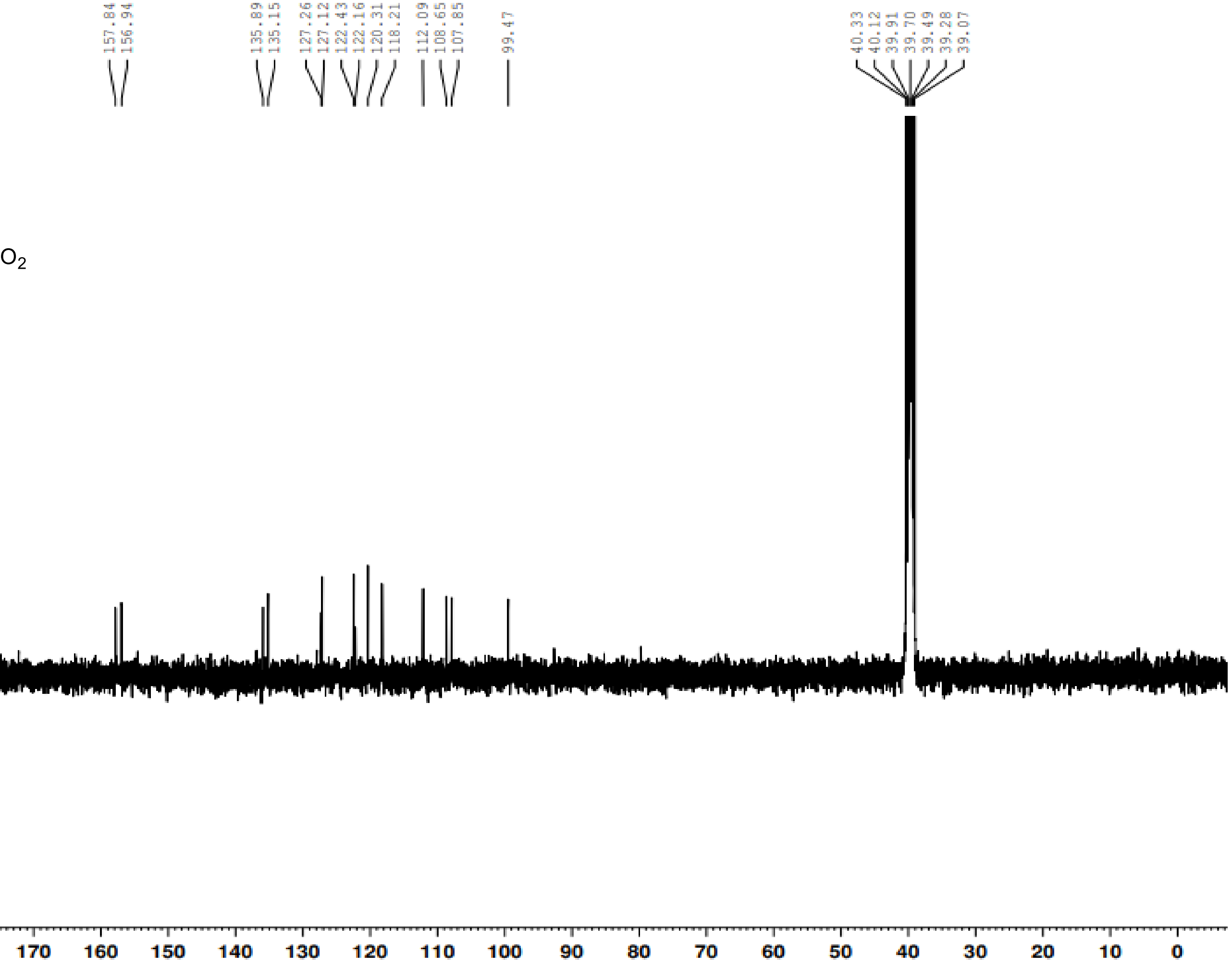

Figure S43. ^{1}H NMR spectrum (400 MHz, DMSO-d_6) of **1b′**.

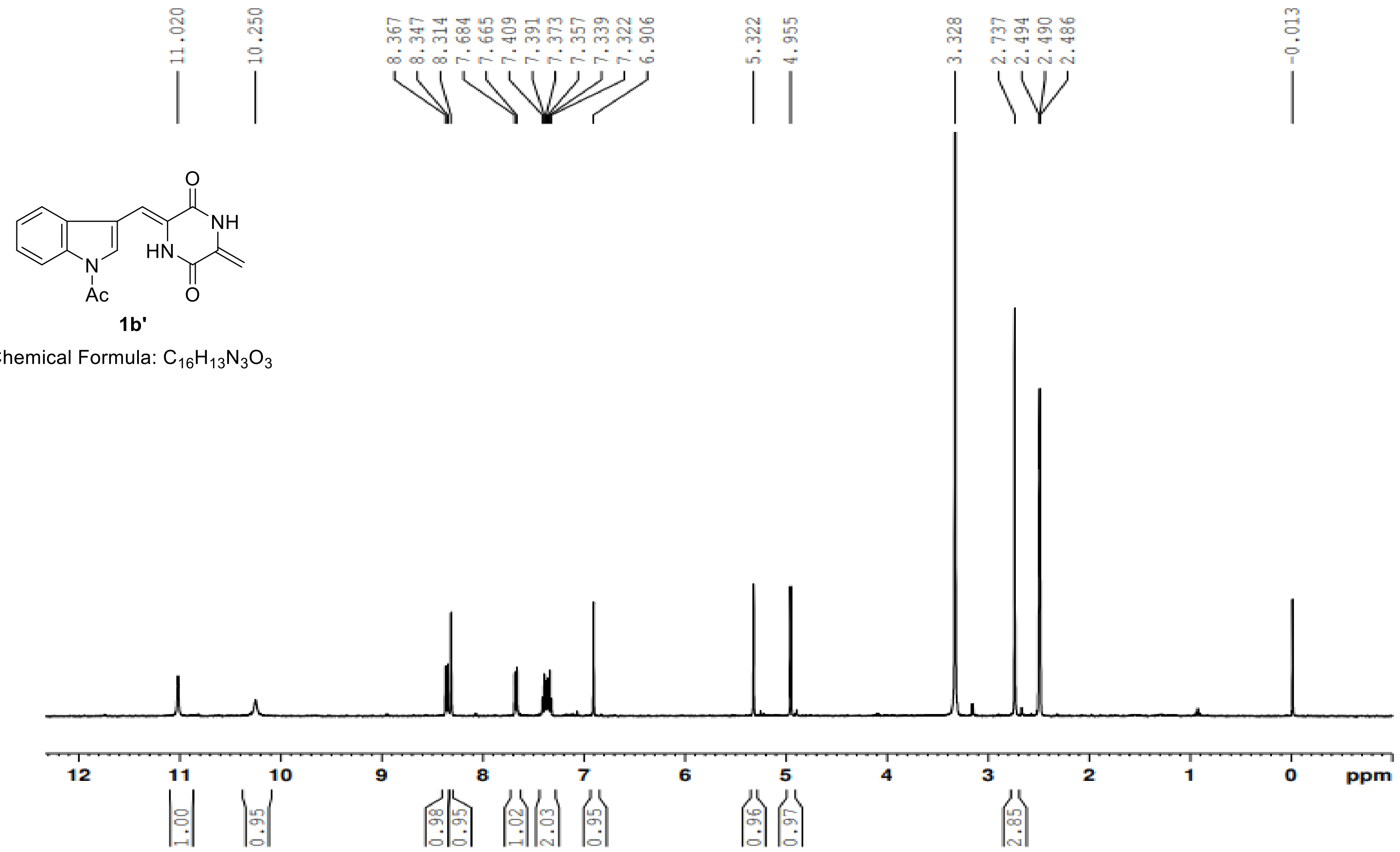

1b'

Chemical Formula: $C_{16}H_{13}N_3O_3$

Figure S44. $^{13}C\{^1H\}$ NMR spectrum (100 MHz, DMSO-d_6) of **1b′**.

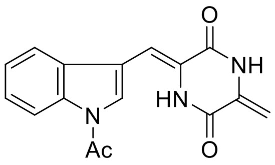

1b'

Chemical Formula: $C_{16}H_{13}N_3O_3$

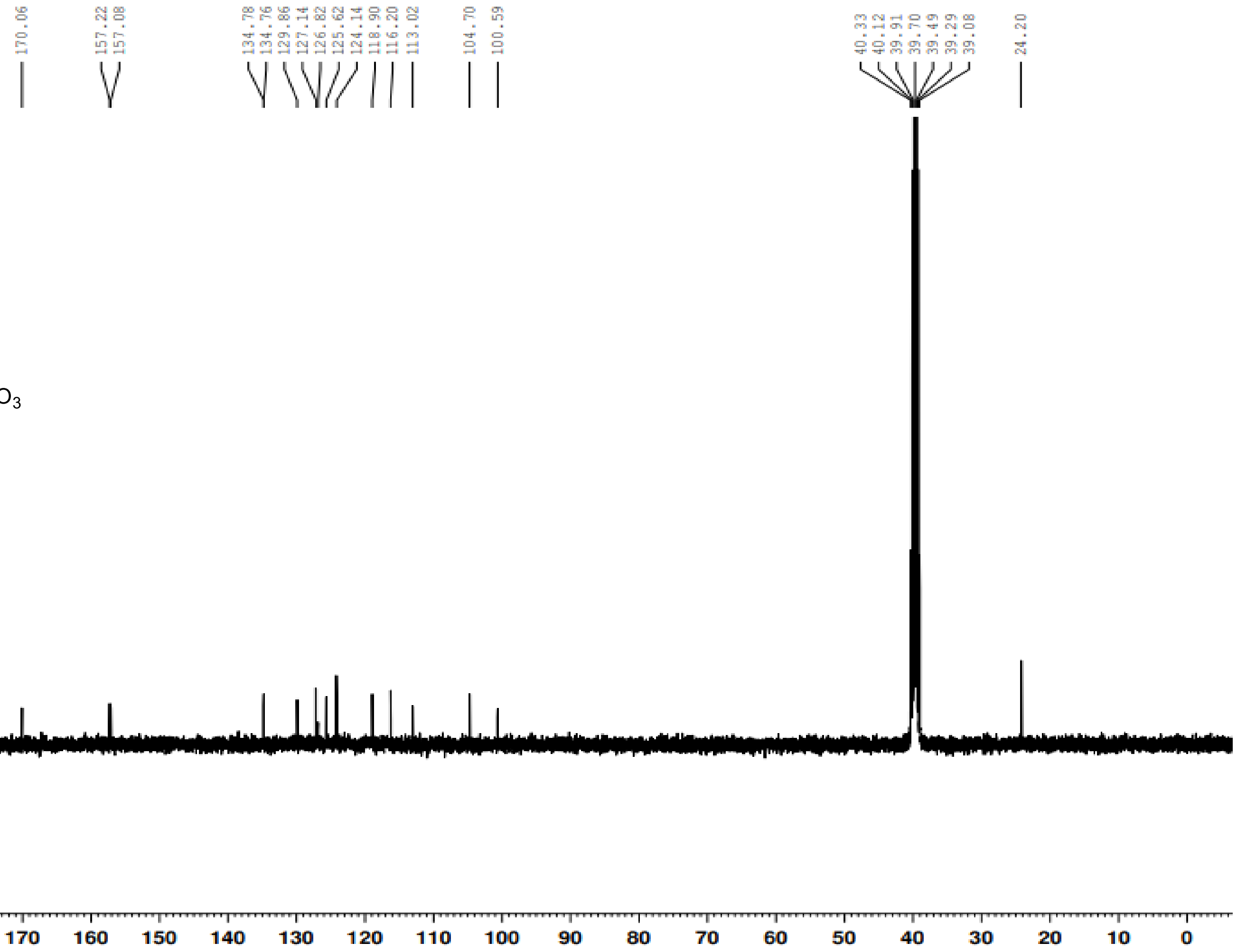

Figure S45. ^{1}H NMR spectrum (400 MHz, DMSO-d_6) of **1c**.

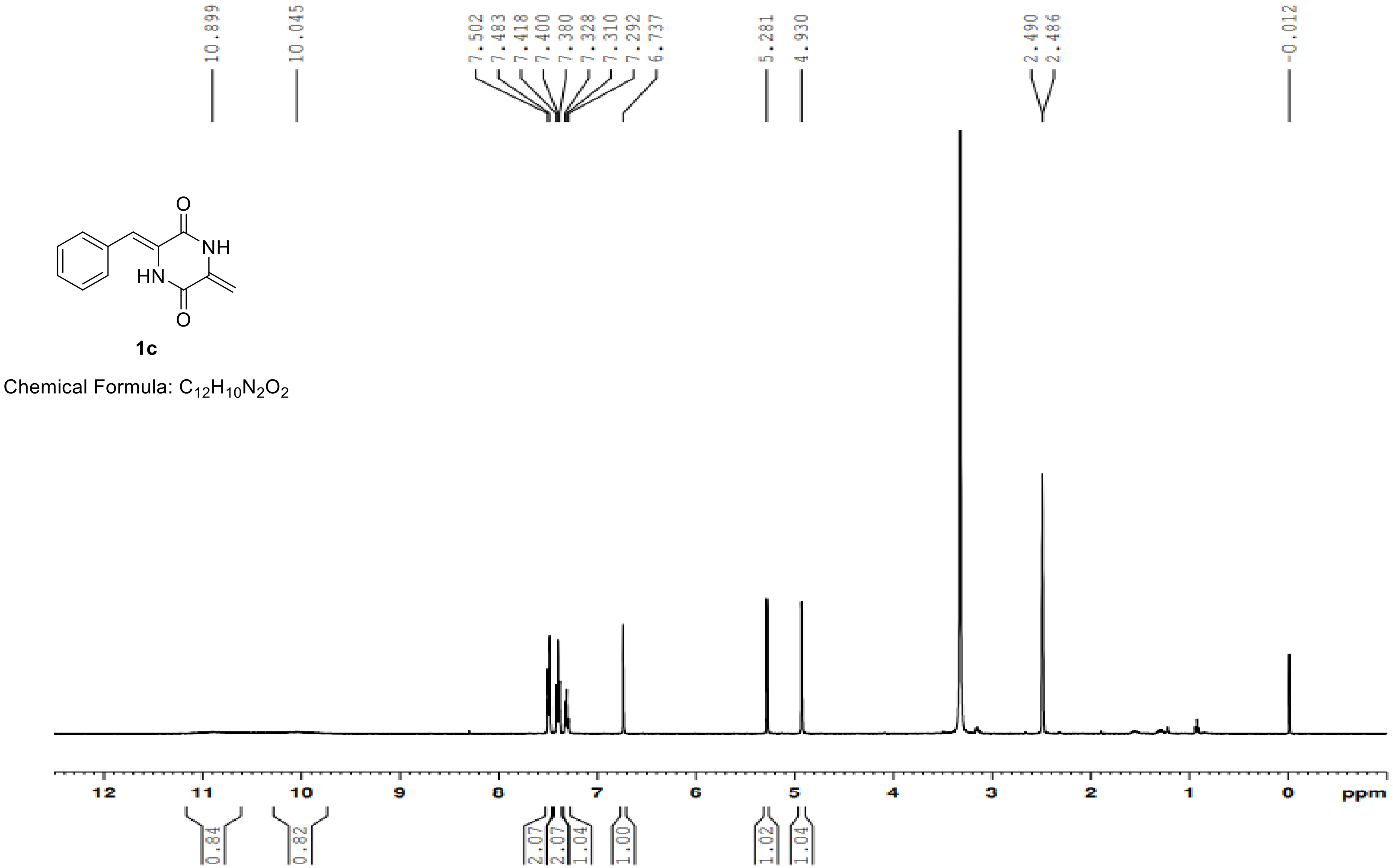

Figure S46. $^{13}C\{^1H\}$ NMR spectrum (100 MHz, DMSO-d_6) of **1c**.

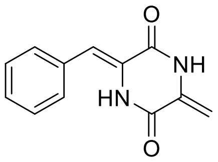

1c

Chemical Formula: $C_{12}H_{10}N_2O_2$

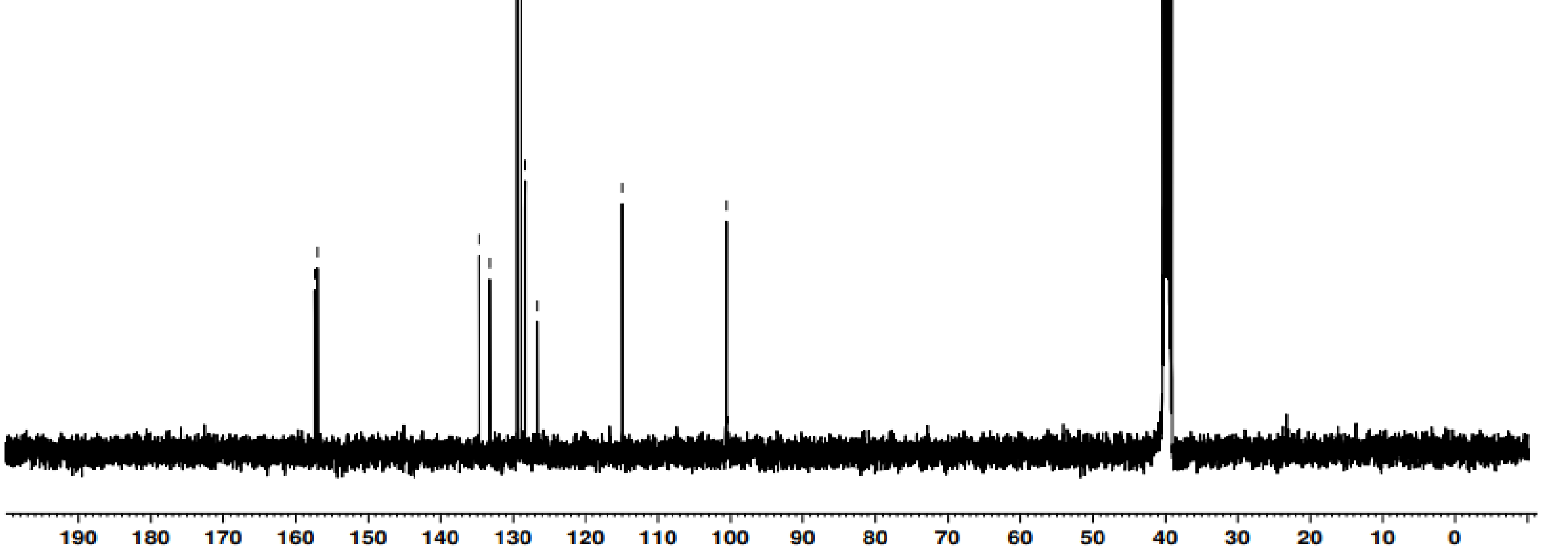

Figure S47. ^{1}H NMR spectrum (400 MHz, DMSO-d_6) of **1d**.

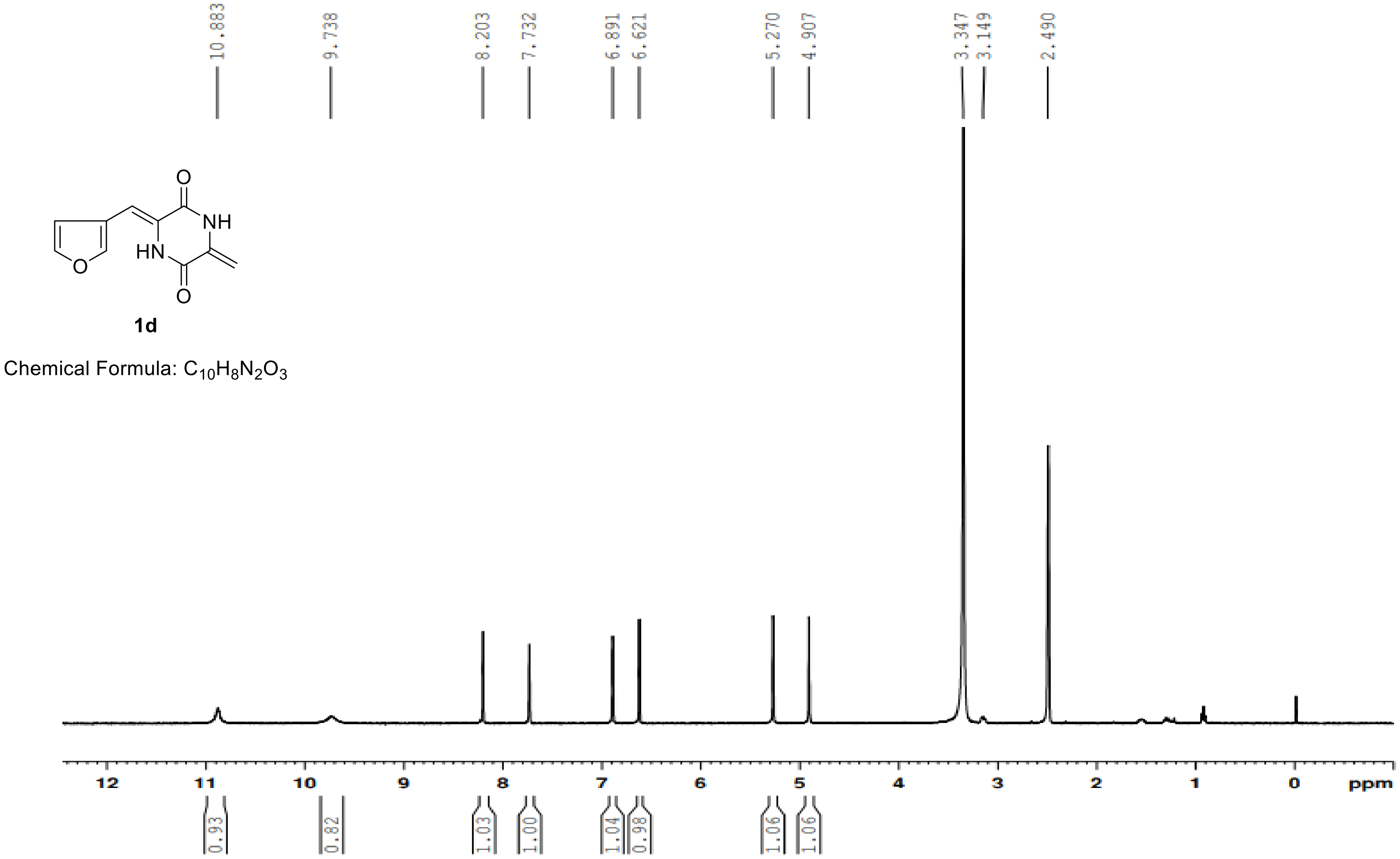

Figure S48. $^{13}C\{^1H\}$ NMR spectrum (100 MHz, DMSO-d_6) of **1d**.

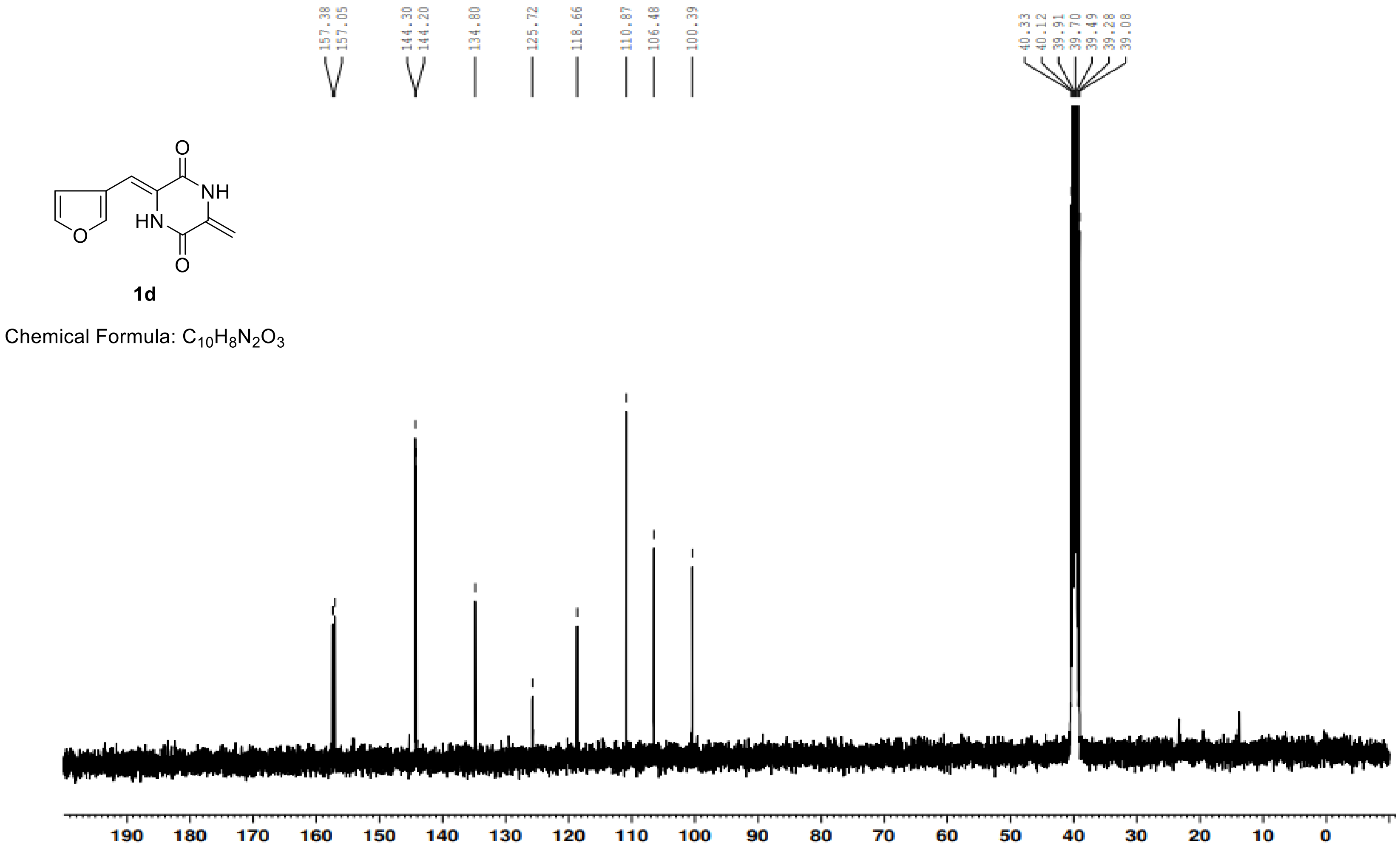

Figure S49. ^{1}H NMR spectrum (400 MHz, DMSO-d_6) of **1e**.

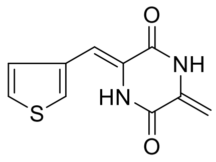

1e

Chemical Formula: $C_{10}H_8N_2O_2S$

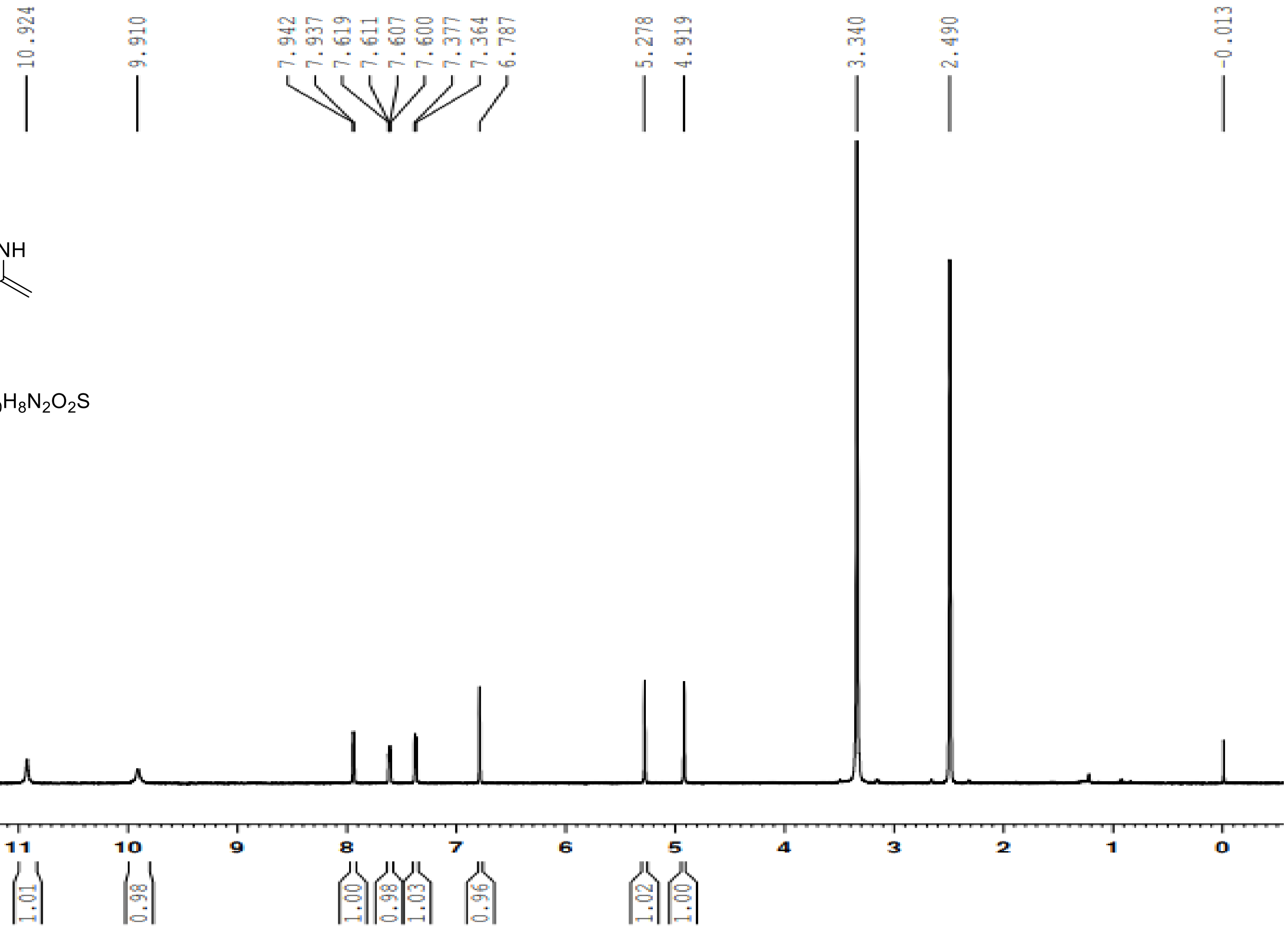

Figure S50. $^{13}C\{^{1}H\}$ NMR spectrum (100 MHz, DMSO-d_6) of **1e**.

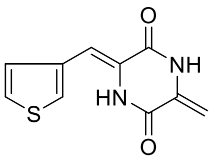

1e

Chemical Formula: $C_{10}H_8N_2O_2S$

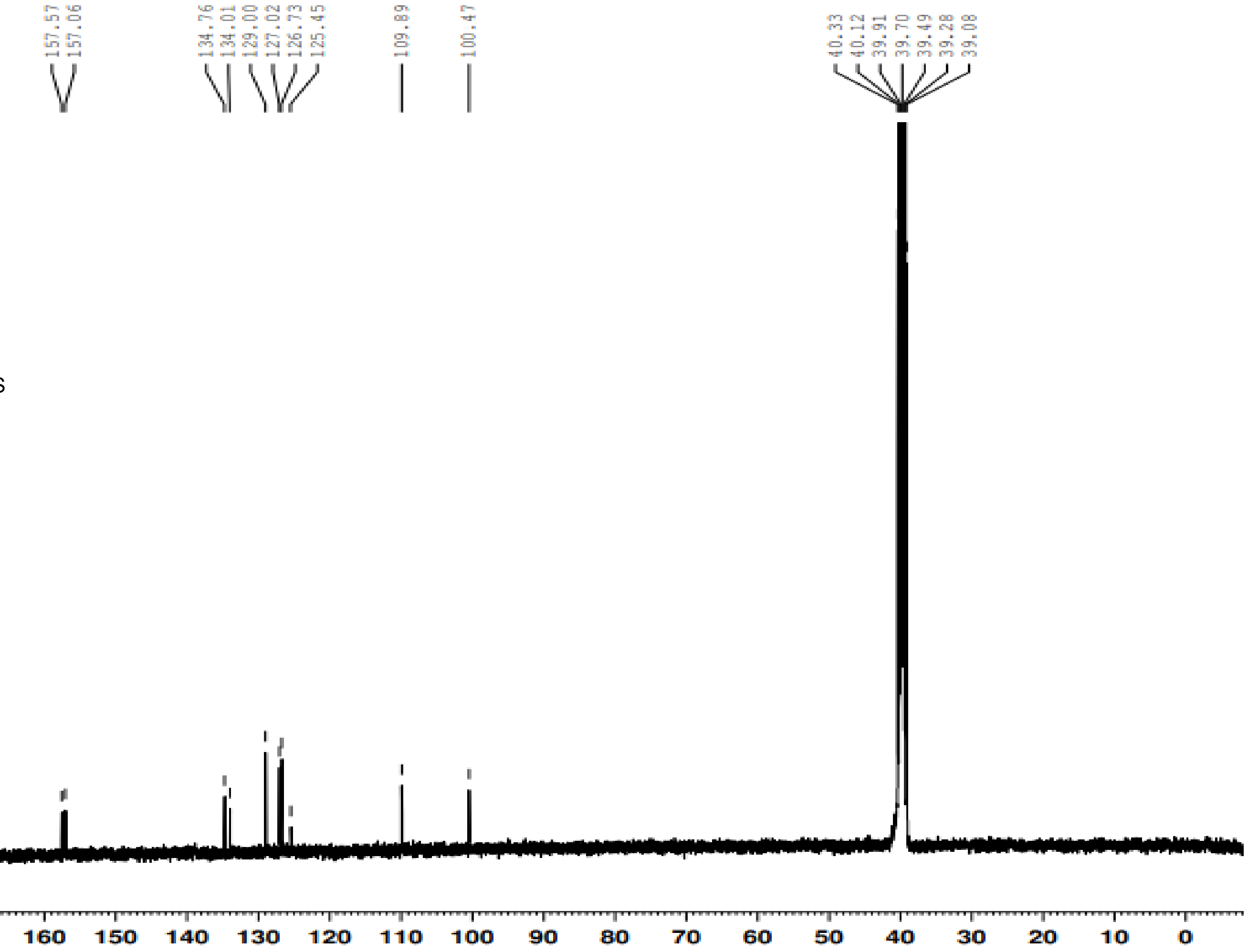

Figure S51. ^{1}H NMR spectrum (400 MHz, DMSO-d_6) of **1f**.

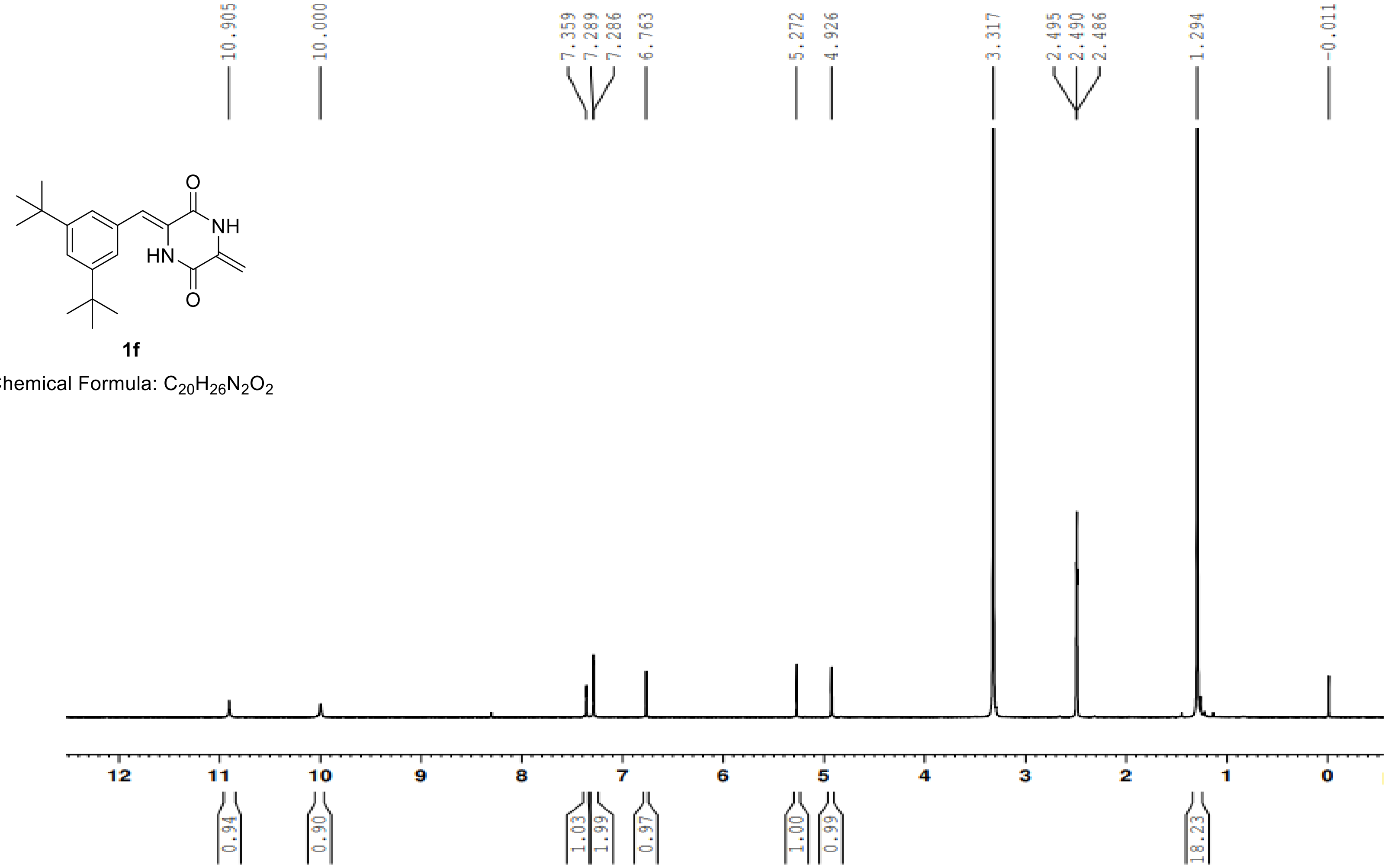

1f

Chemical Formula: $C_{20}H_{26}N_2O_2$

Figure S52. $^{13}C\{^{1}H\}$ NMR spectrum (100 MHz, DMSO-d_6) of **1f**.

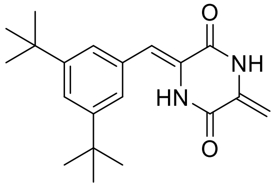

1f

Chemical Formula: $C_{20}H_{26}N_2O_2$

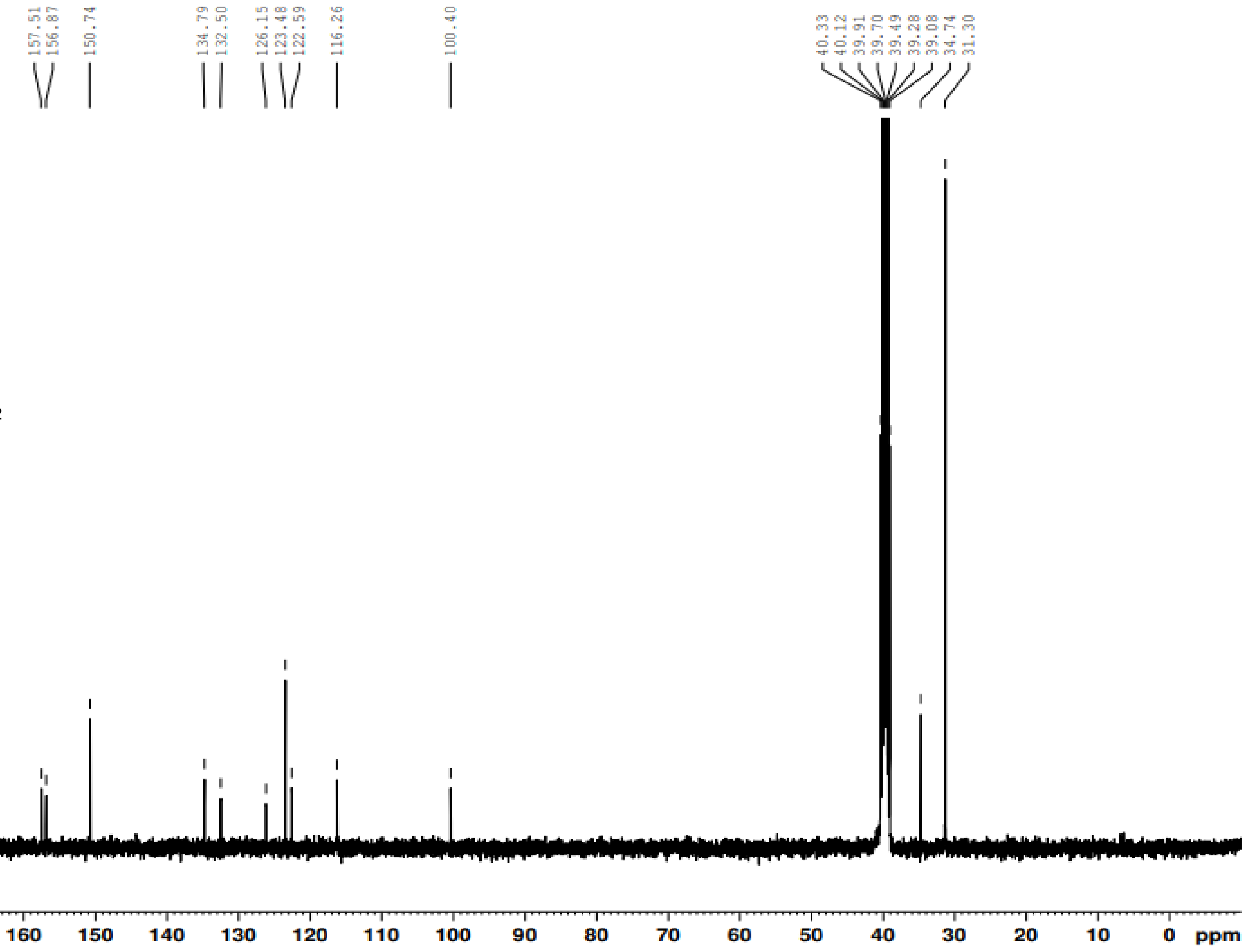

Figure S53. ^{1}H NMR spectrum (400 MHz, DMSO-d_6) of **1g**.

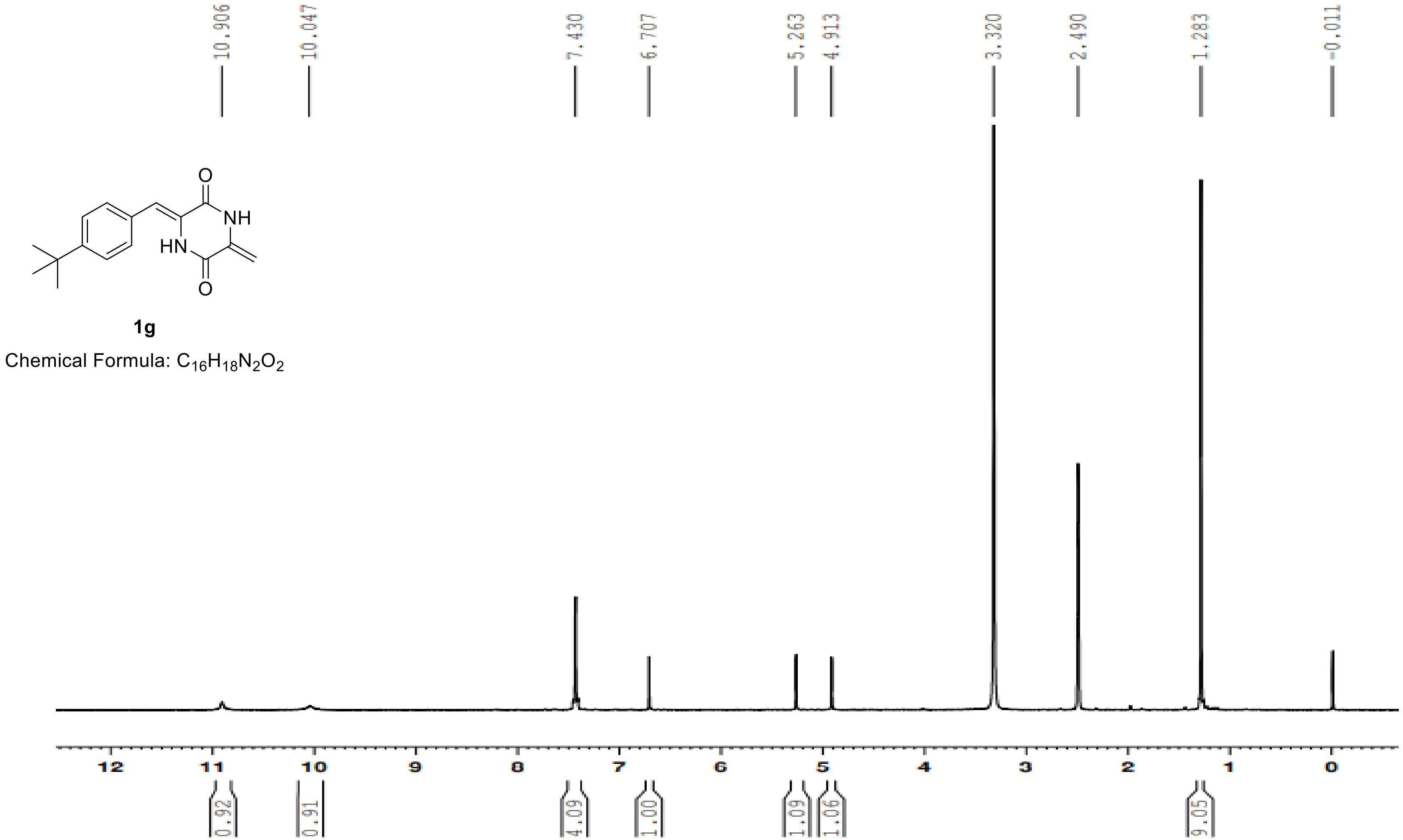

Figure S54. $^{13}C\{^1H\}$ NMR spectrum (100 MHz, DMSO-d_6) of **1g**.

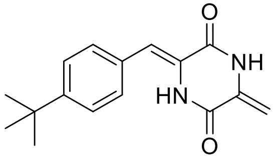

1g

Chemical Formula: $C_{16}H_{18}N_2O_2$

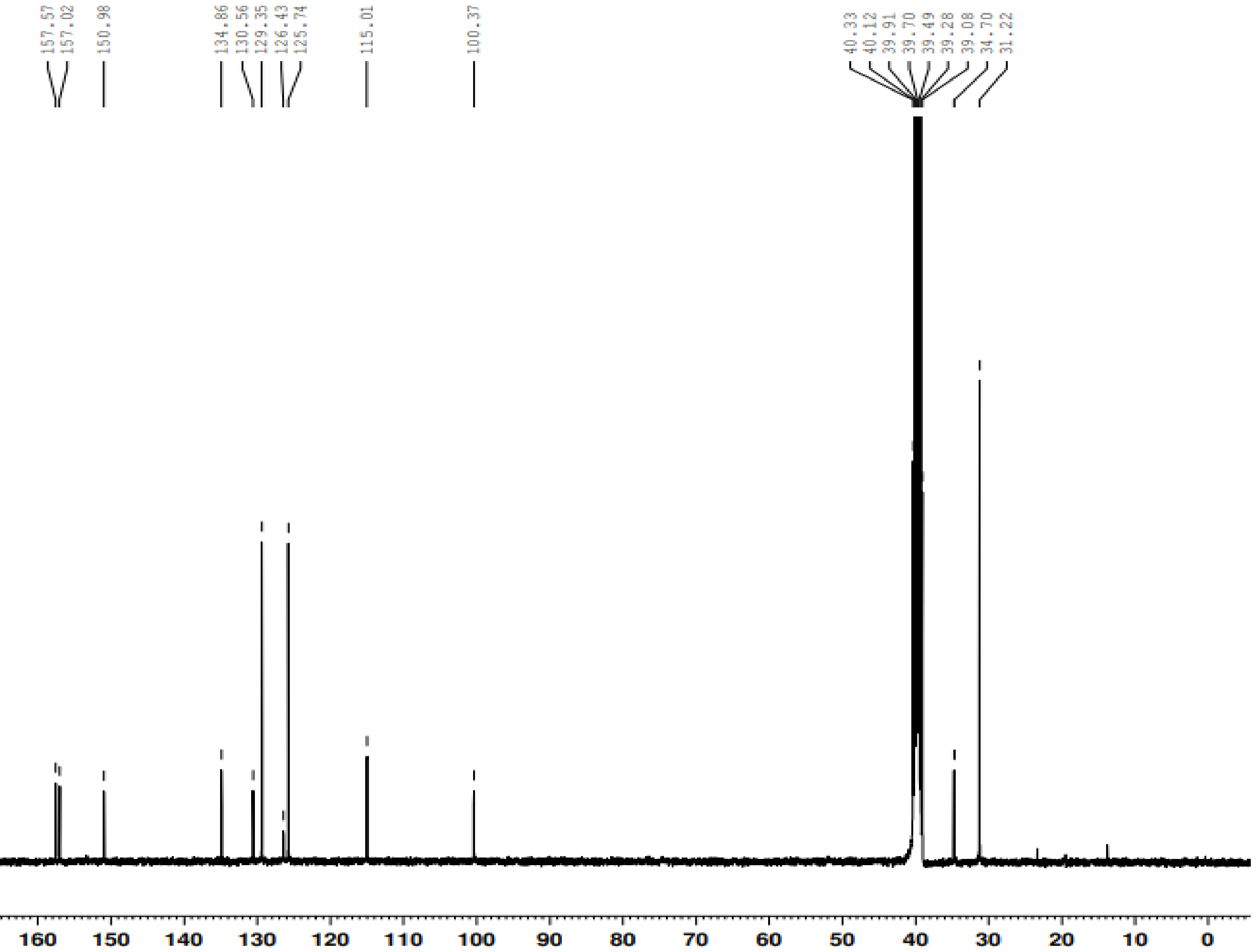

Figure S55. ^{1}H NMR spectrum (400 MHz, DMSO-d_6) of **1h**.

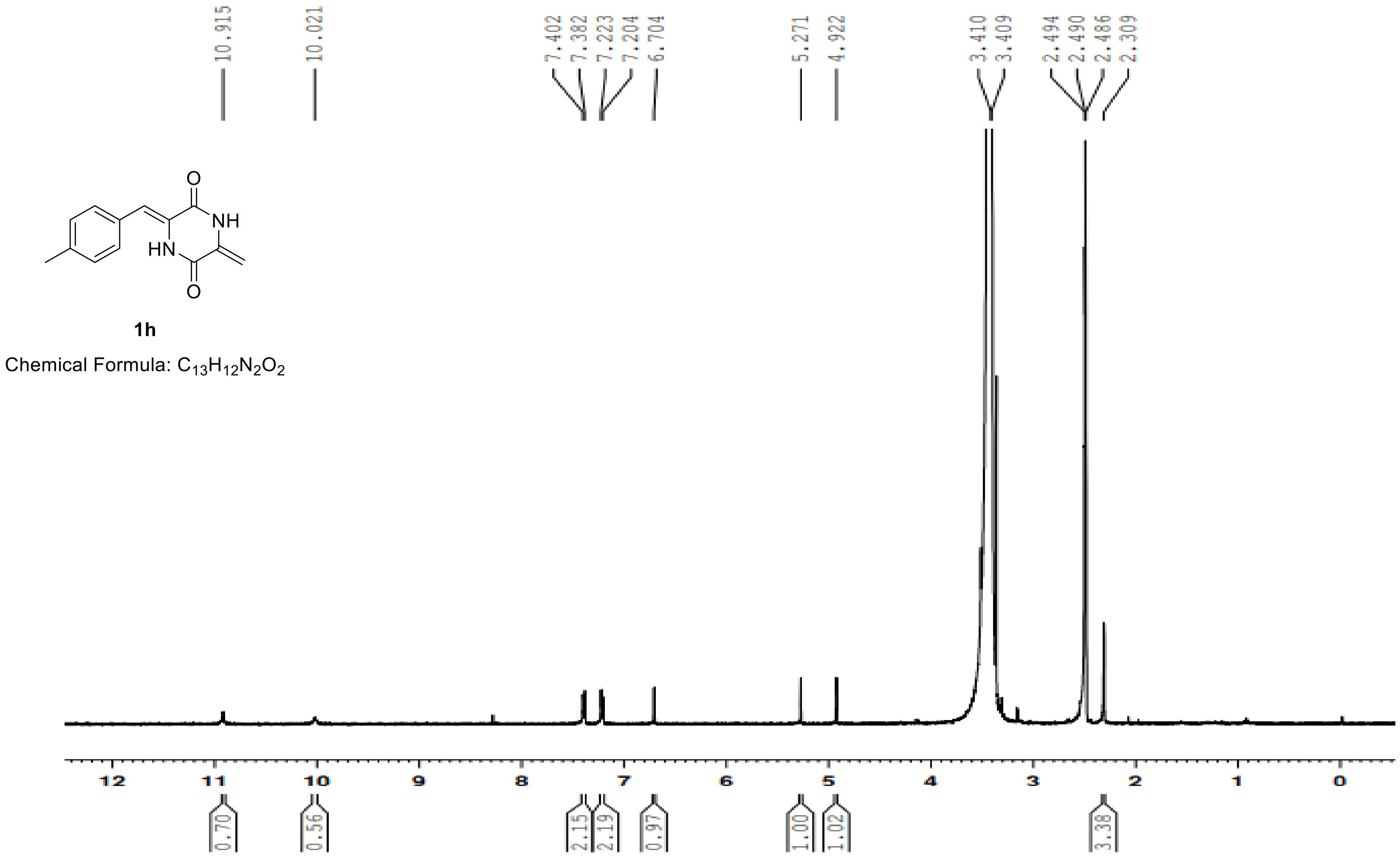

Figure S56. $^{13}C\{^1H\}$ NMR spectrum (100 MHz, DMSO-d_6) of **1h**.

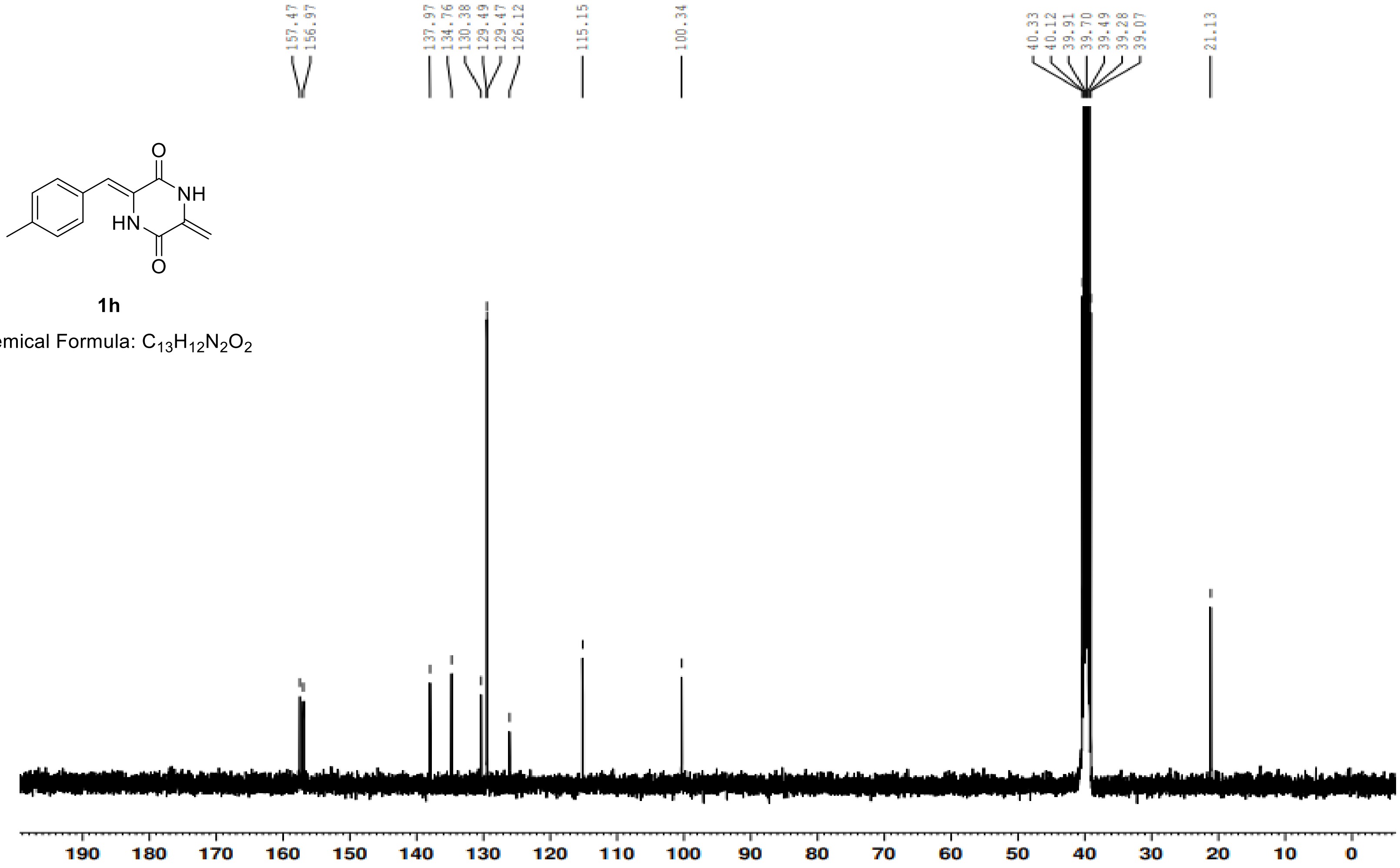

1h

Chemical Formula: $C_{13}H_{12}N_2O_2$

Figure S57. ^{1}H NMR spectrum (400 MHz, DMSO-d_6) of **1i**.

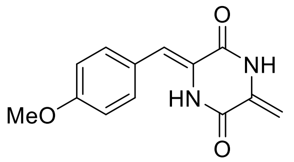

1i

Chemical Formula: $C_{13}H_{12}N_2O_3$

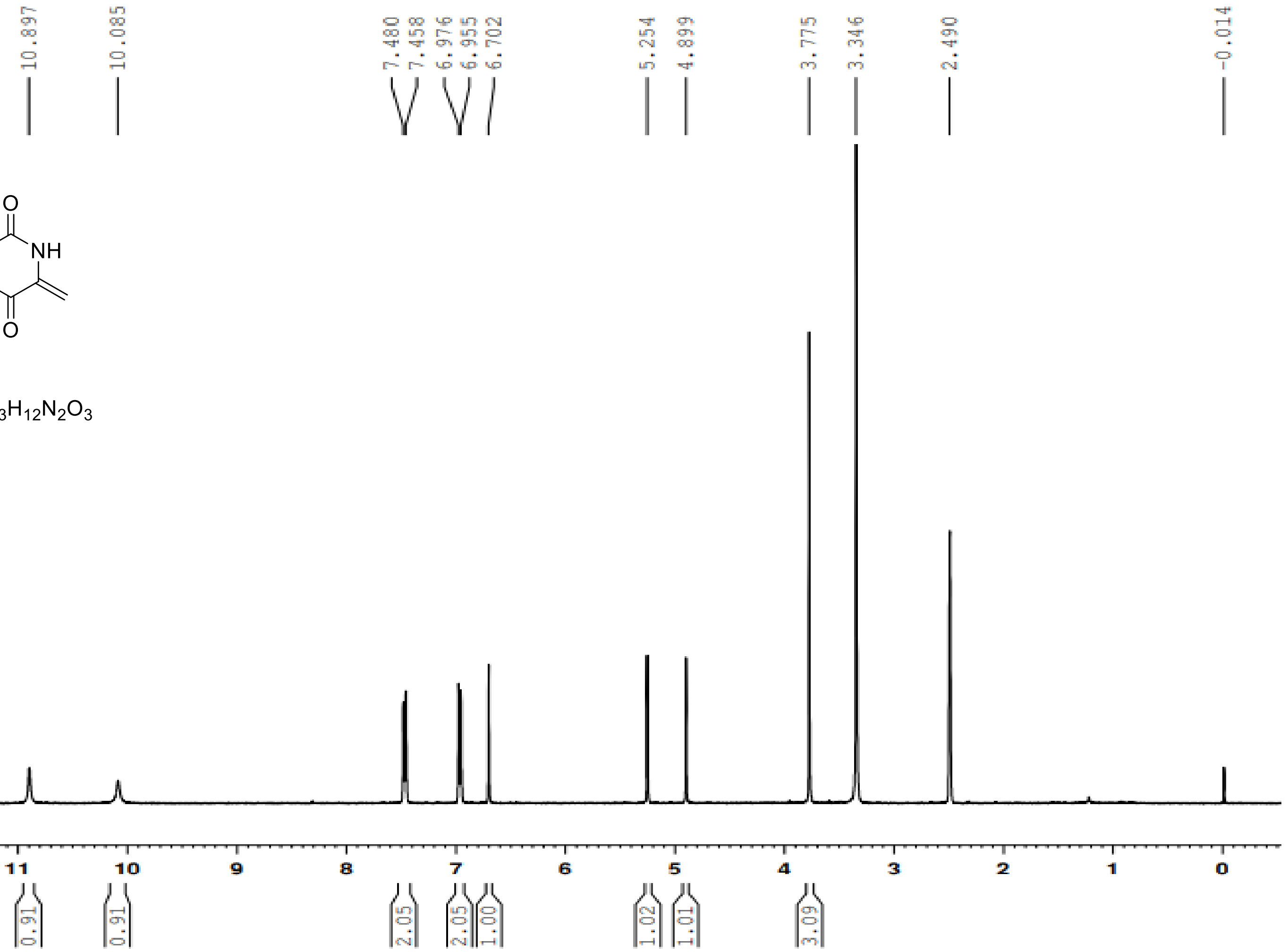

Figure S58. $^{13}C\{^1H\}$ NMR spectrum (100 MHz, DMSO-d_6) of **1i**.

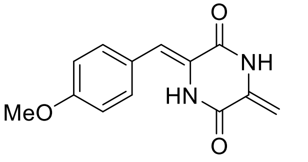

1i

Chemical Formula: $C_{13}H_{12}N_2O_3$

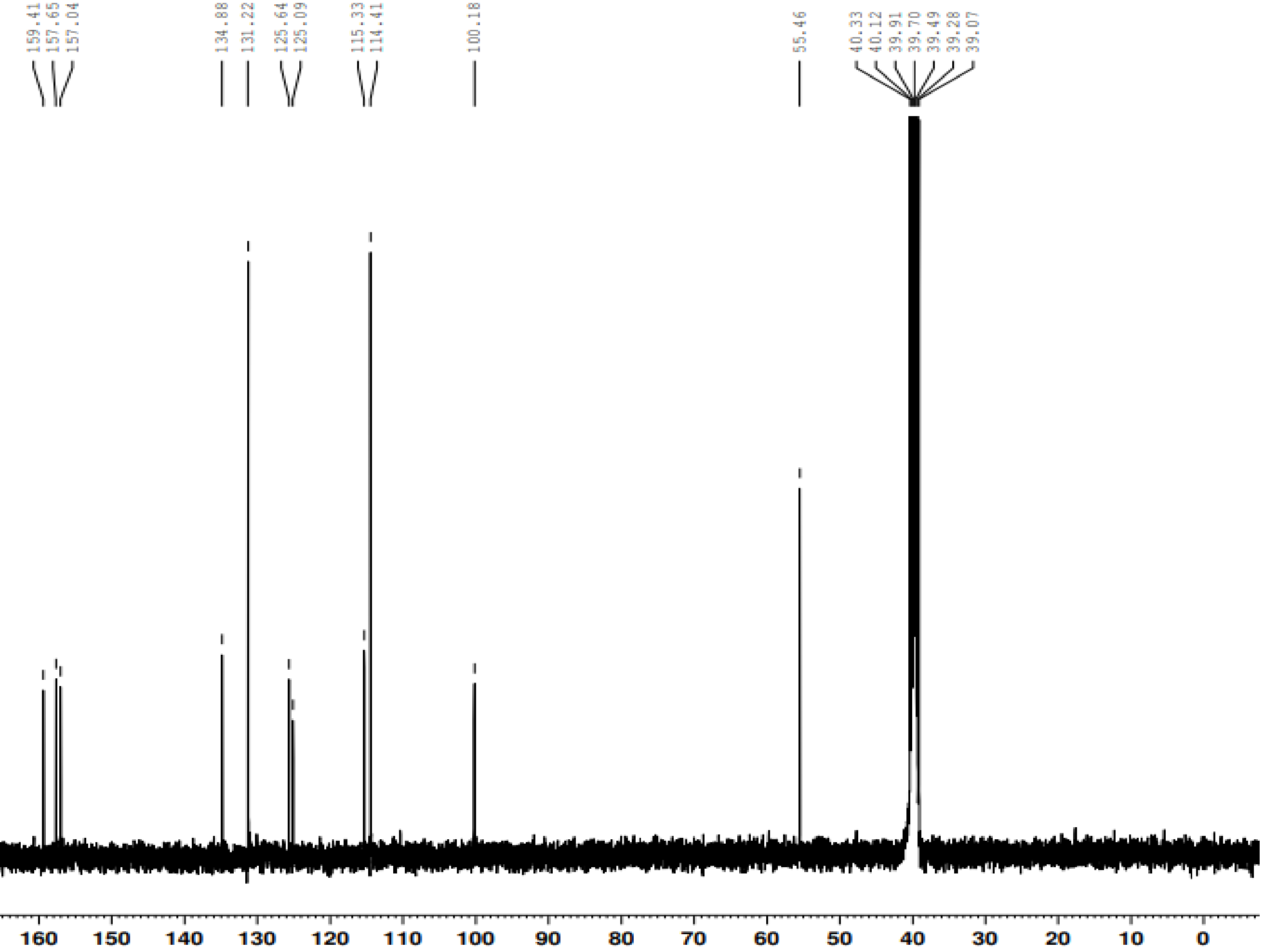

Figure S59. ^{1}H NMR spectrum (400 MHz, DMSO-d_6) of **1j**.

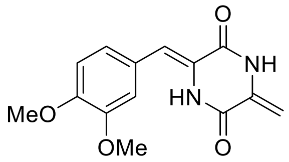

1j

Chemical Formula: $C_{14}H_{14}N_2O_4$

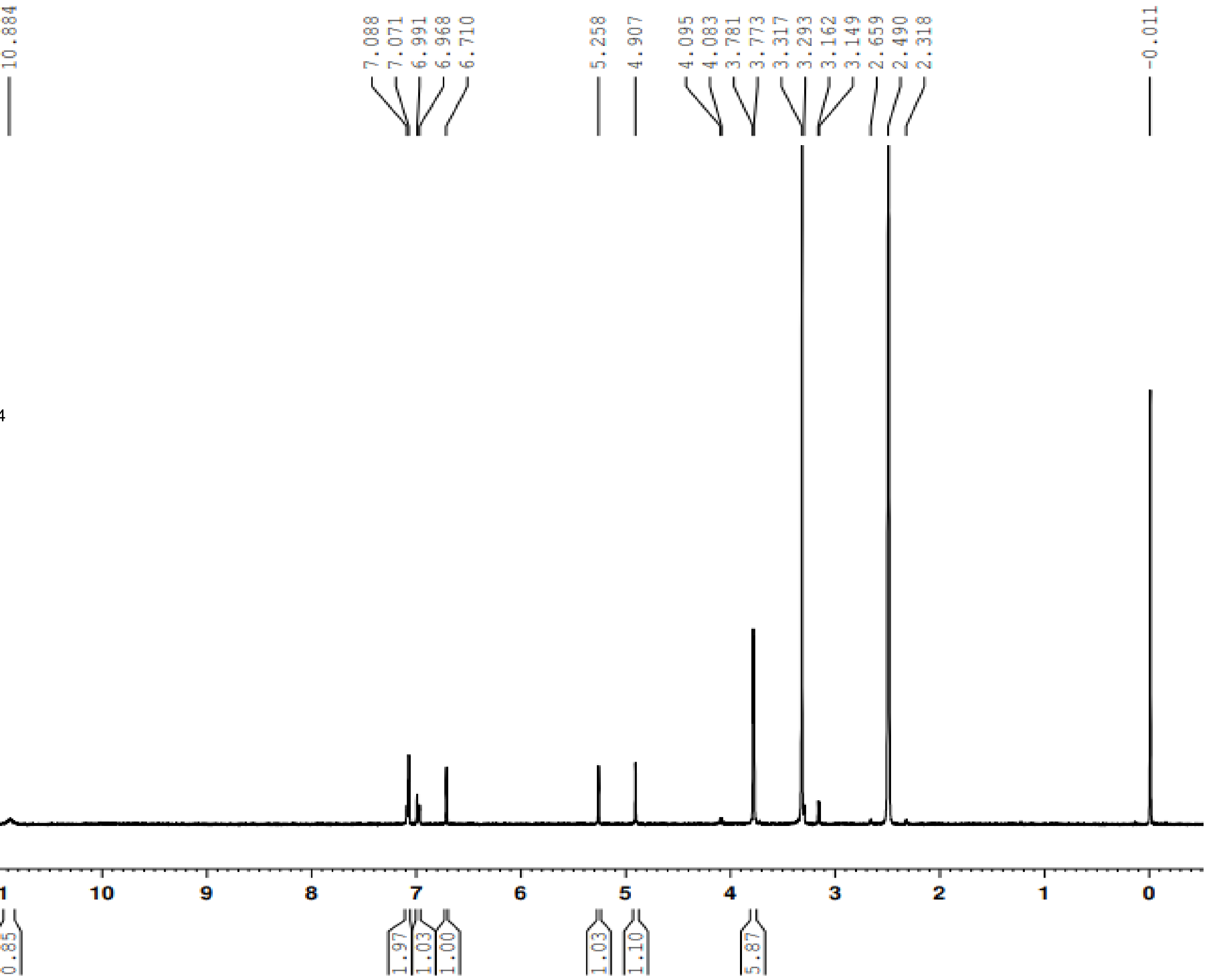

Figure S60. $^{13}C\{^{1}H\}$ NMR spectrum (100 MHz, DMSO-d_6) of **1j**.

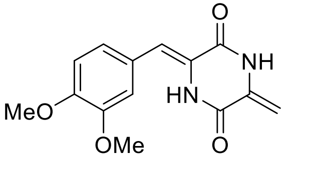

1j

Chemical Formula: $C_{14}H_{14}N_2O_4$

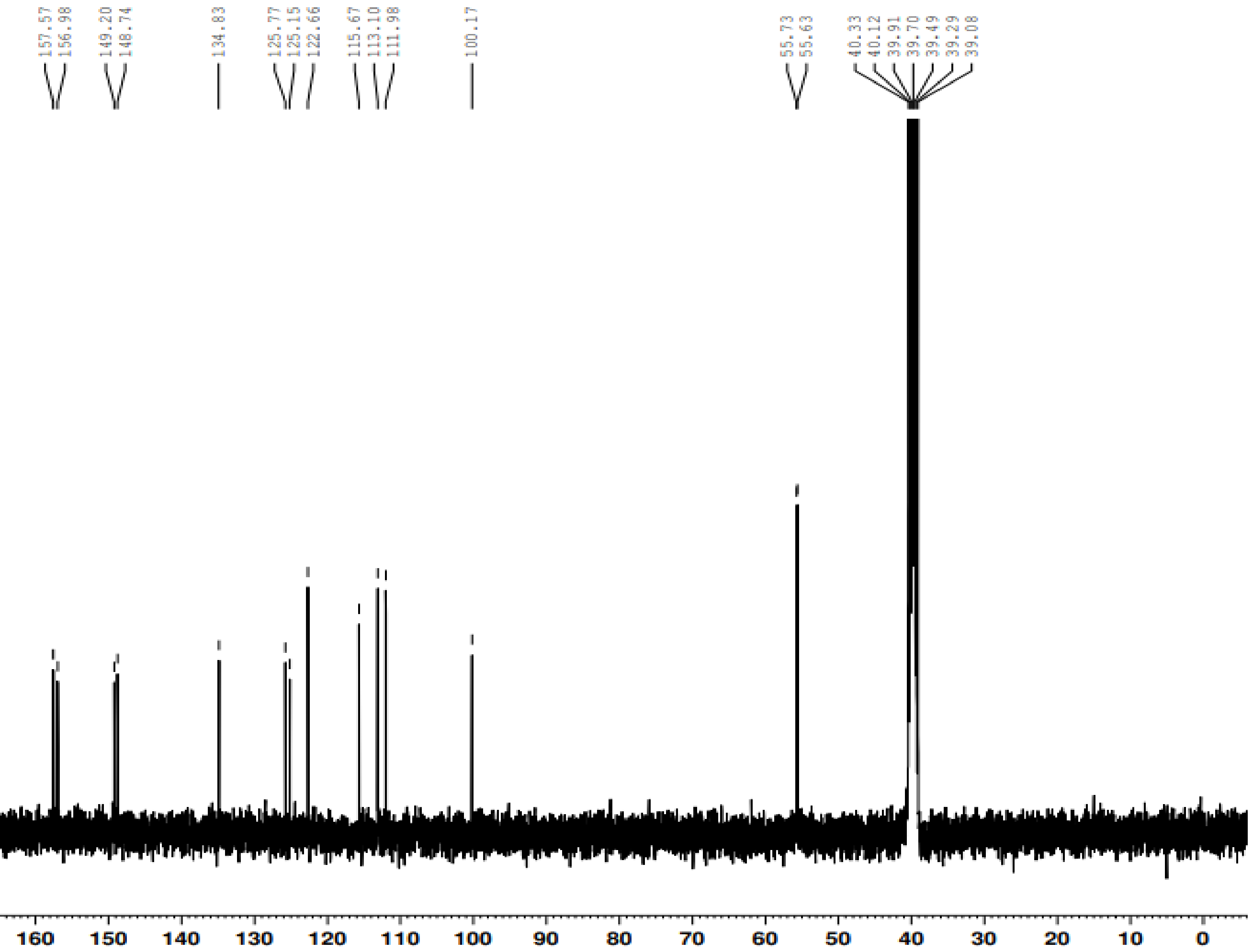

Figure S61. ^1H NMR spectrum (400 MHz, DMSO-d_6) of **1k**.

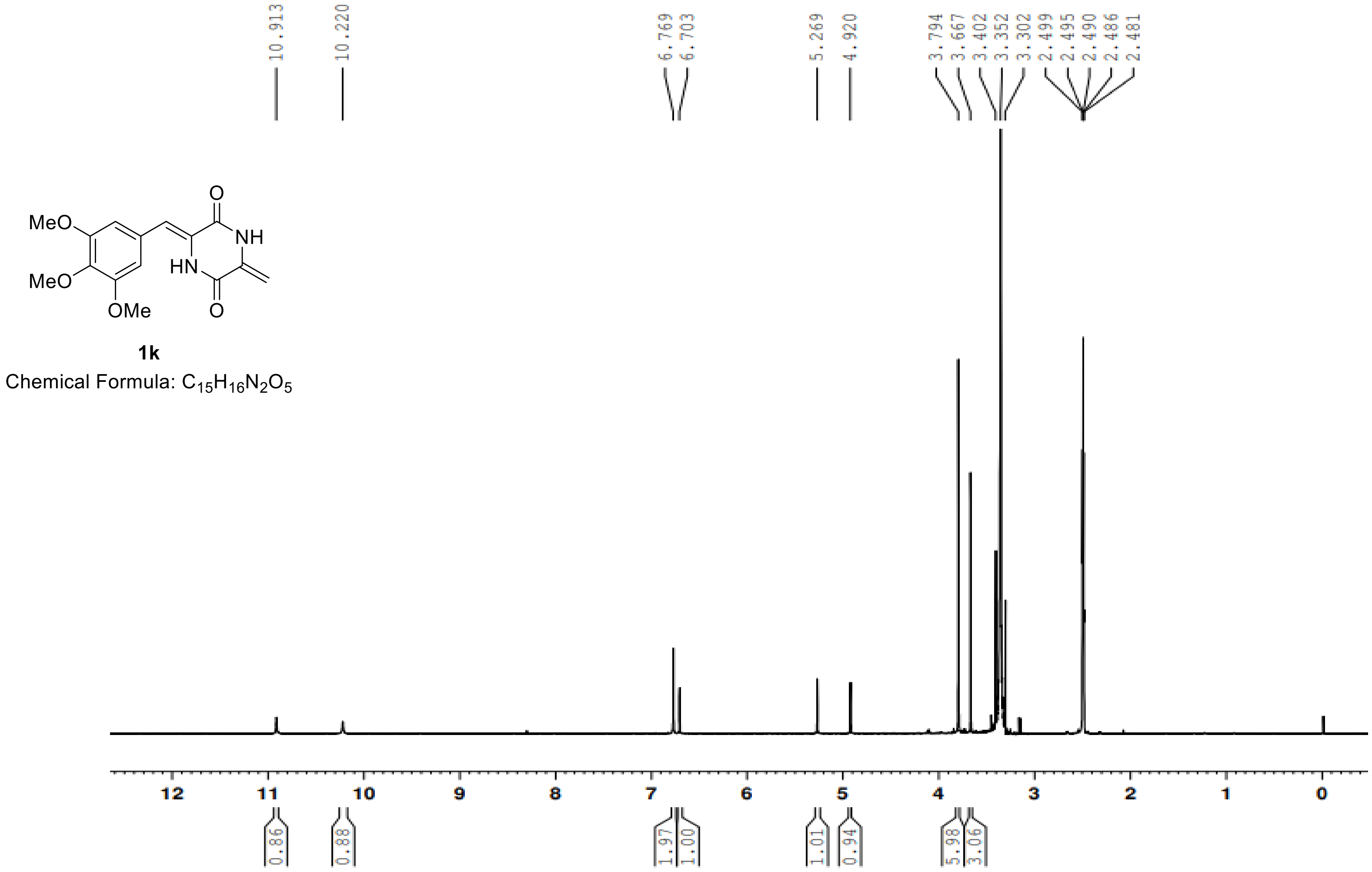

Figure S62. $^{13}C\{^{1}H\}$ NMR spectrum (100 MHz, DMSO-d_6) of **1k**.

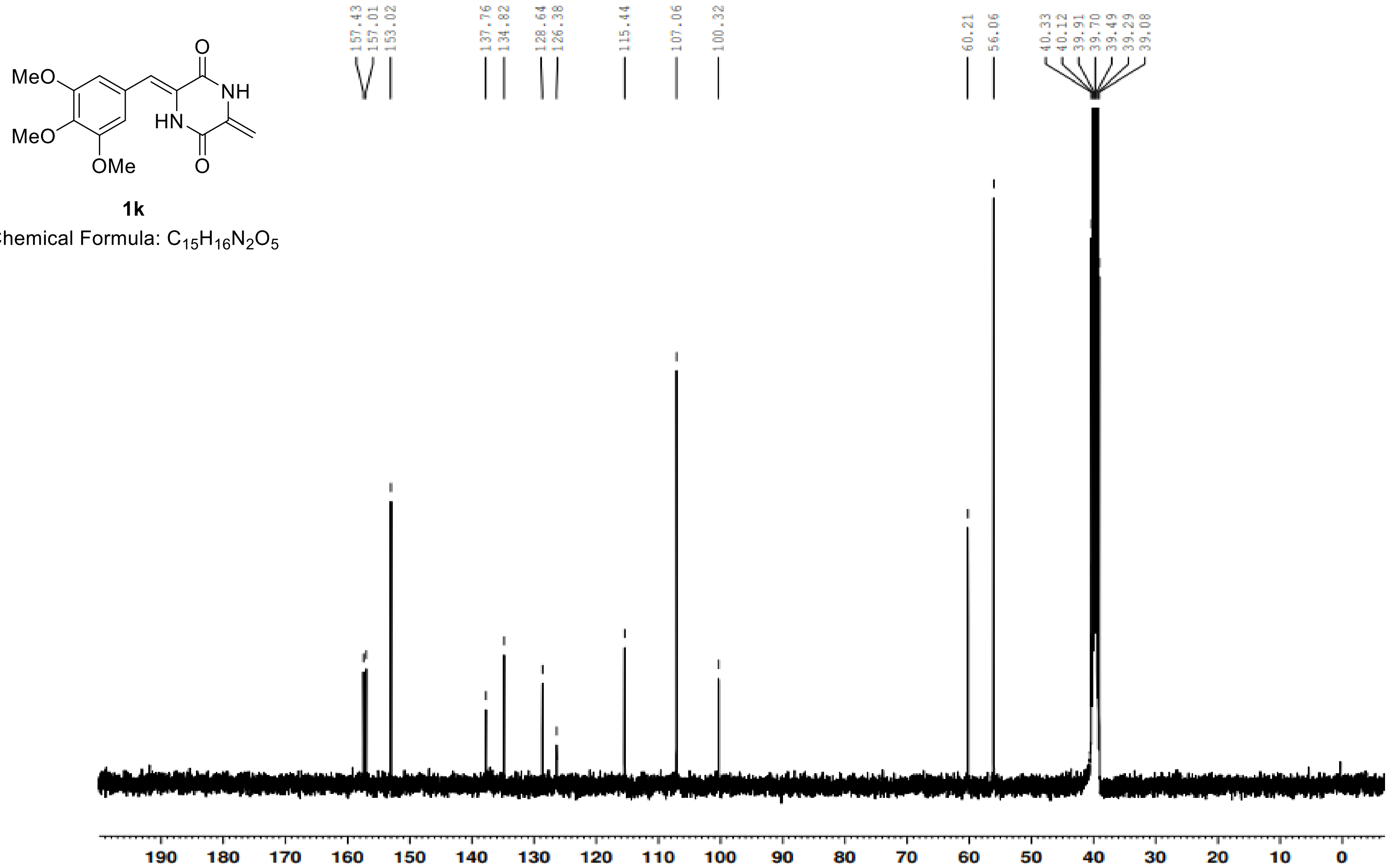

Figure S63. ^{1}H NMR spectrum (400 MHz, DMSO-d_6) of **1*l***.

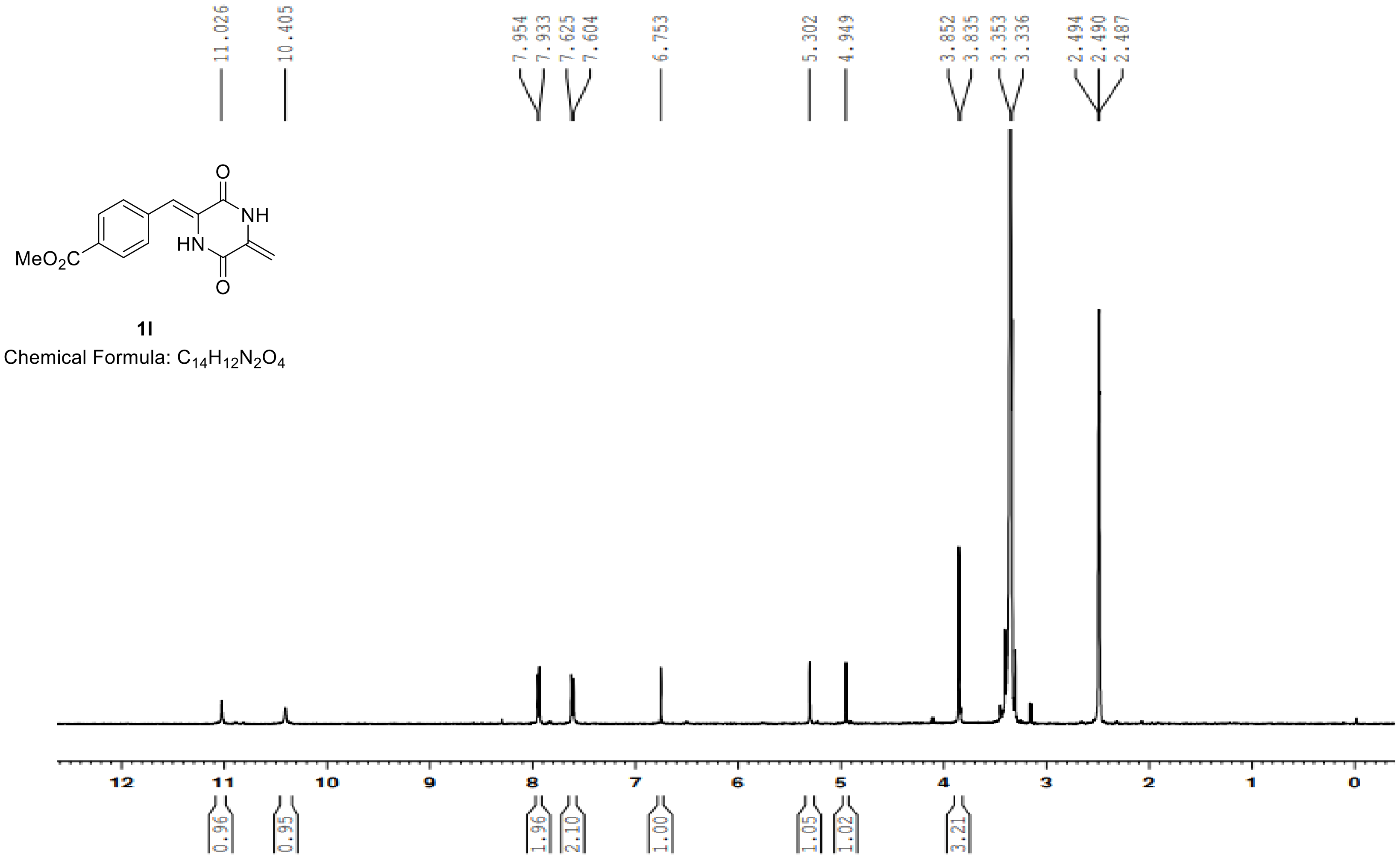

Figure S64. $^{13}C\{^{1}H\}$ NMR spectrum (100 MHz, DMSO-d_6) of **1*l***.

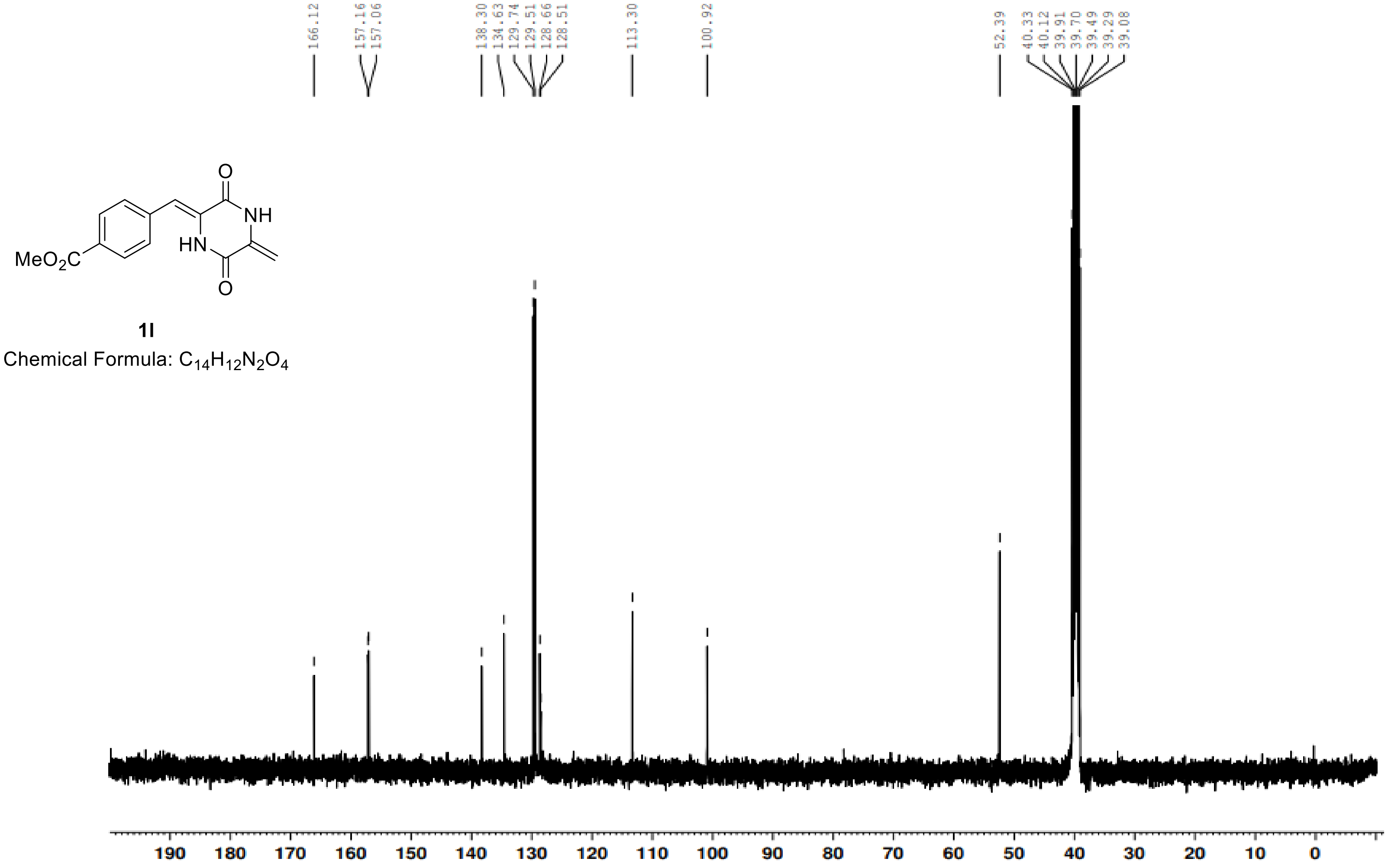

Figure S65. ^{1}H NMR spectrum (400 MHz, DMSO-d_6) of **1m**.

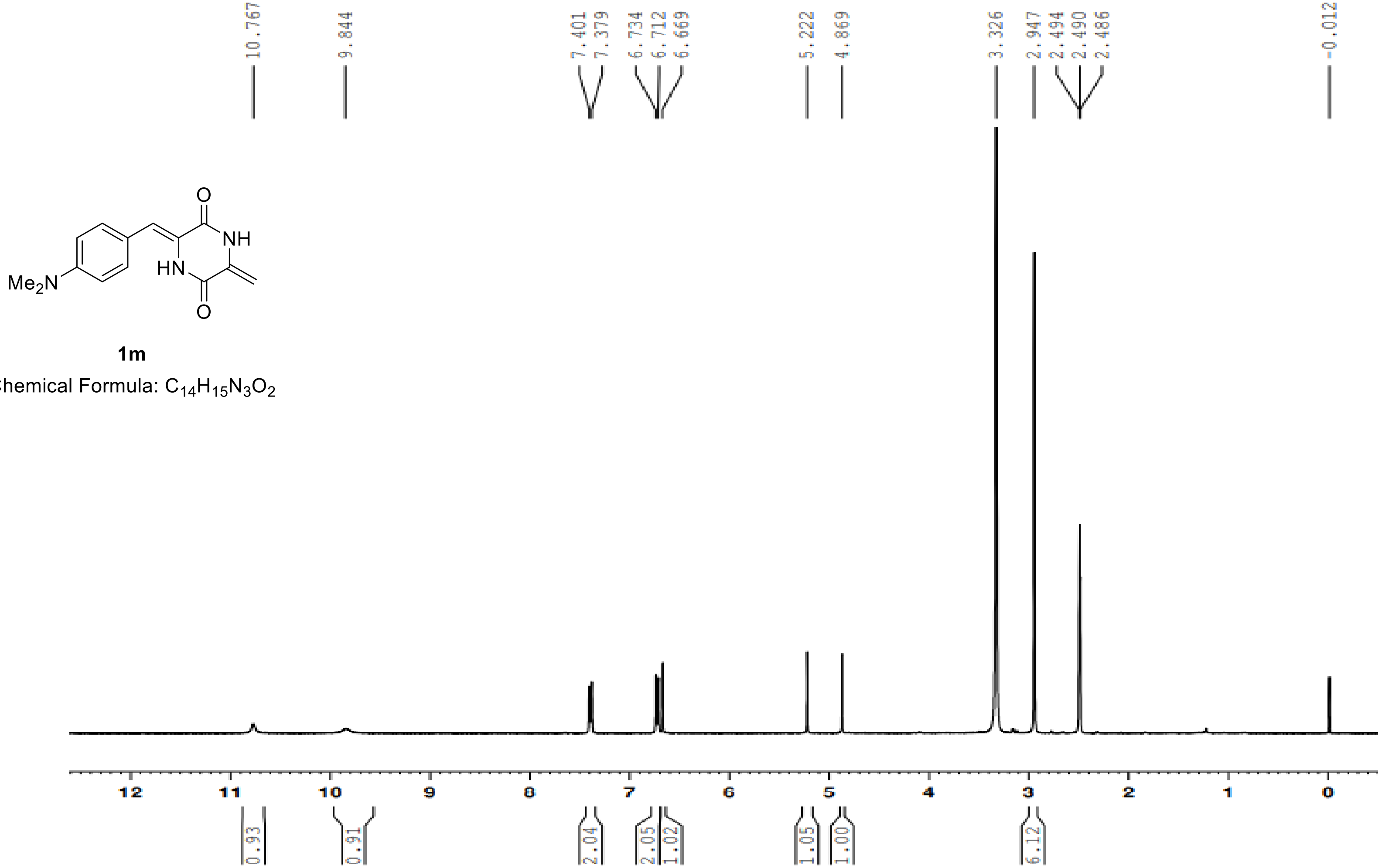

1m

Chemical Formula: $C_{14}H_{15}N_3O_2$

Figure S66. $^{13}C\{^{1}H\}$ NMR spectrum (100 MHz, DMSO-d_6) of **1m**.

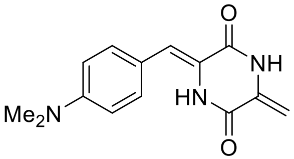

1m

Chemical Formula: $C_{14}H_{15}N_3O_2$

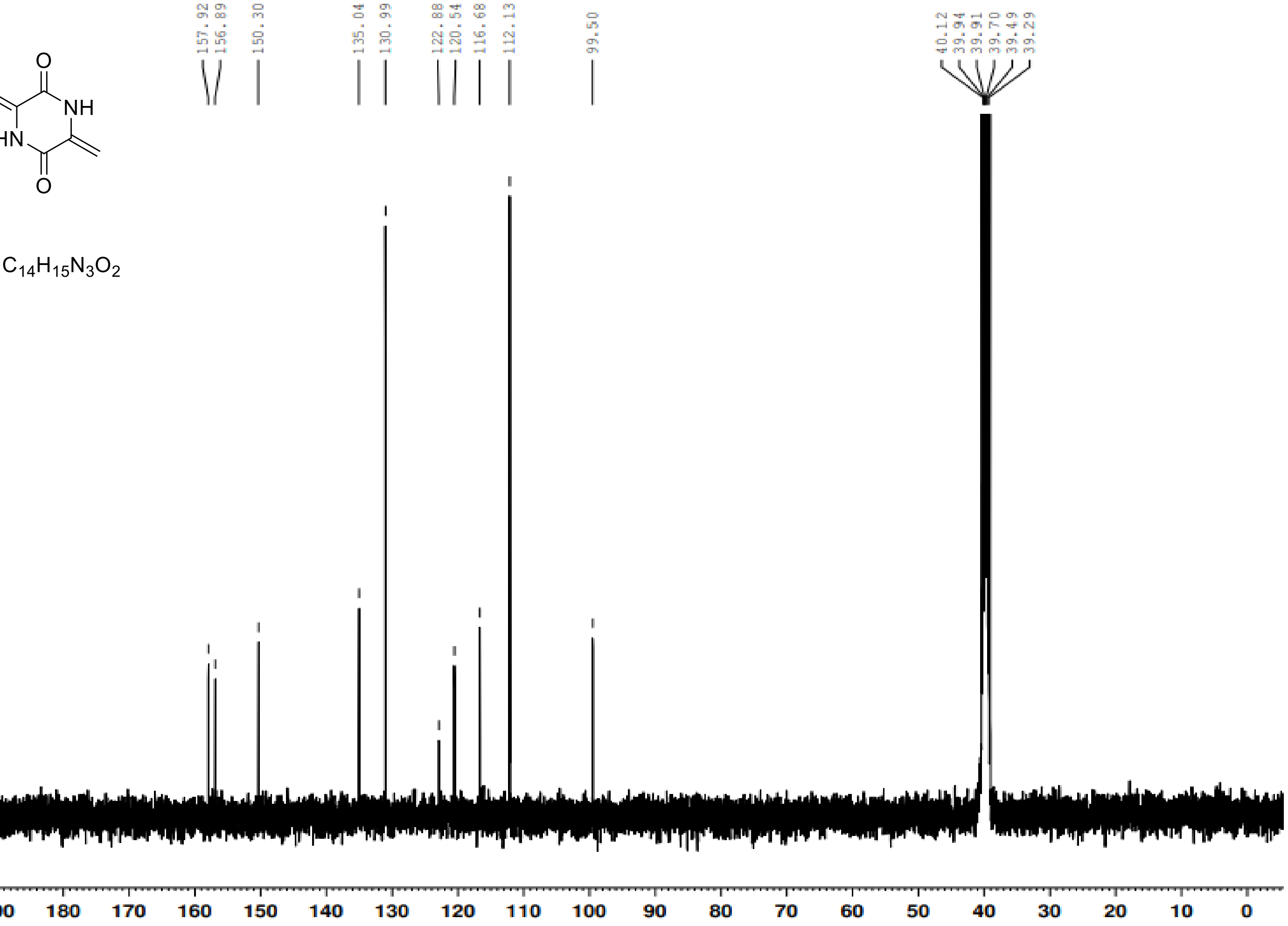

Figure S67. ^{1}H NMR spectrum (400 MHz, DMSO-d_6) of **1n**.

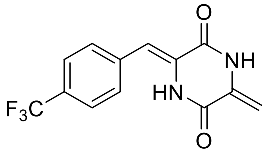

1n

Chemical Formula: $C_{13}H_9F_3N_2O_2$

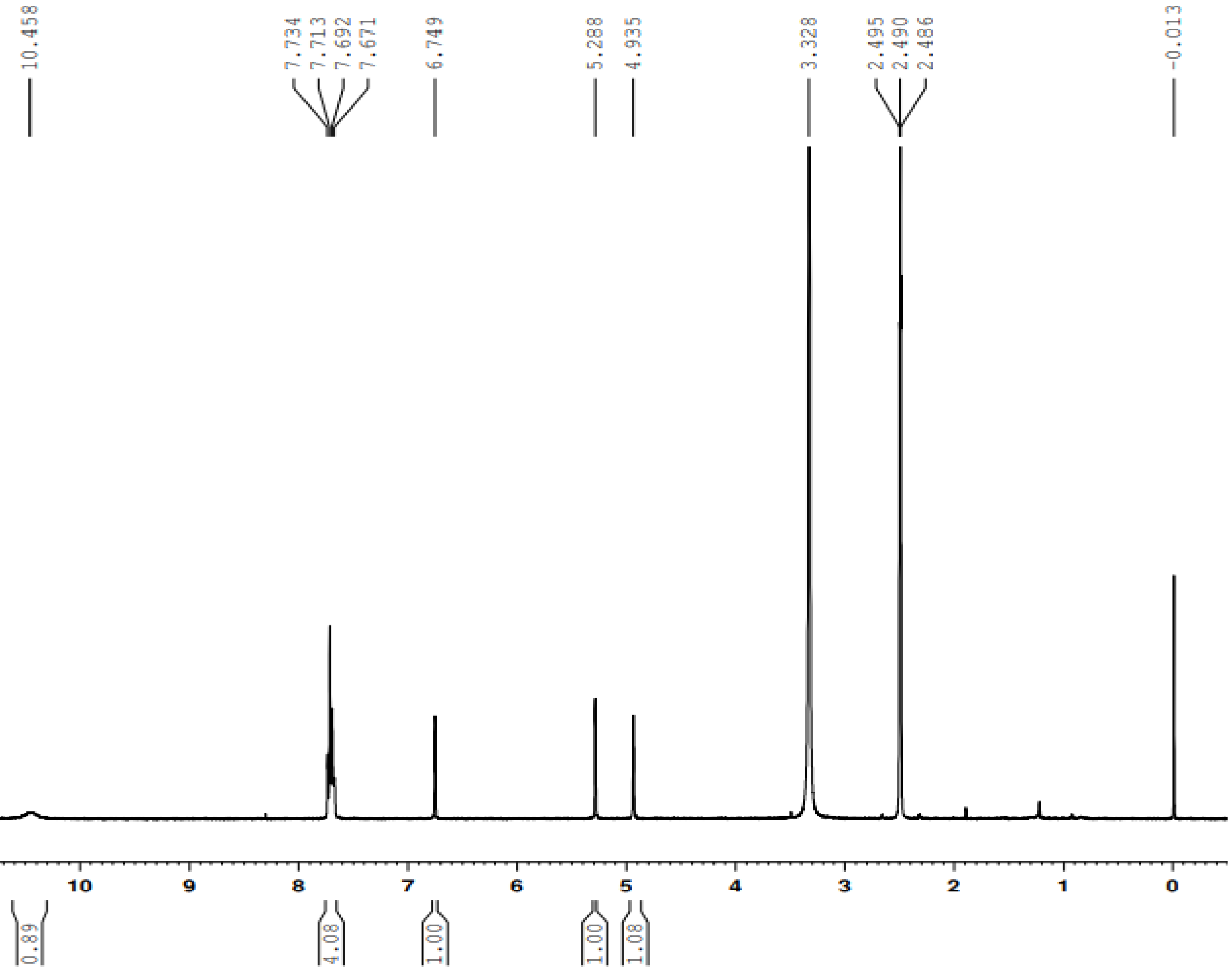

Figure S68. $^{13}C\{^1H\}$ NMR spectrum (100 MHz, DMSO-d_6) of **1n**.

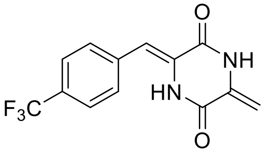

1n

Chemical Formula: $C_{13}H_9F_3N_2O_2$

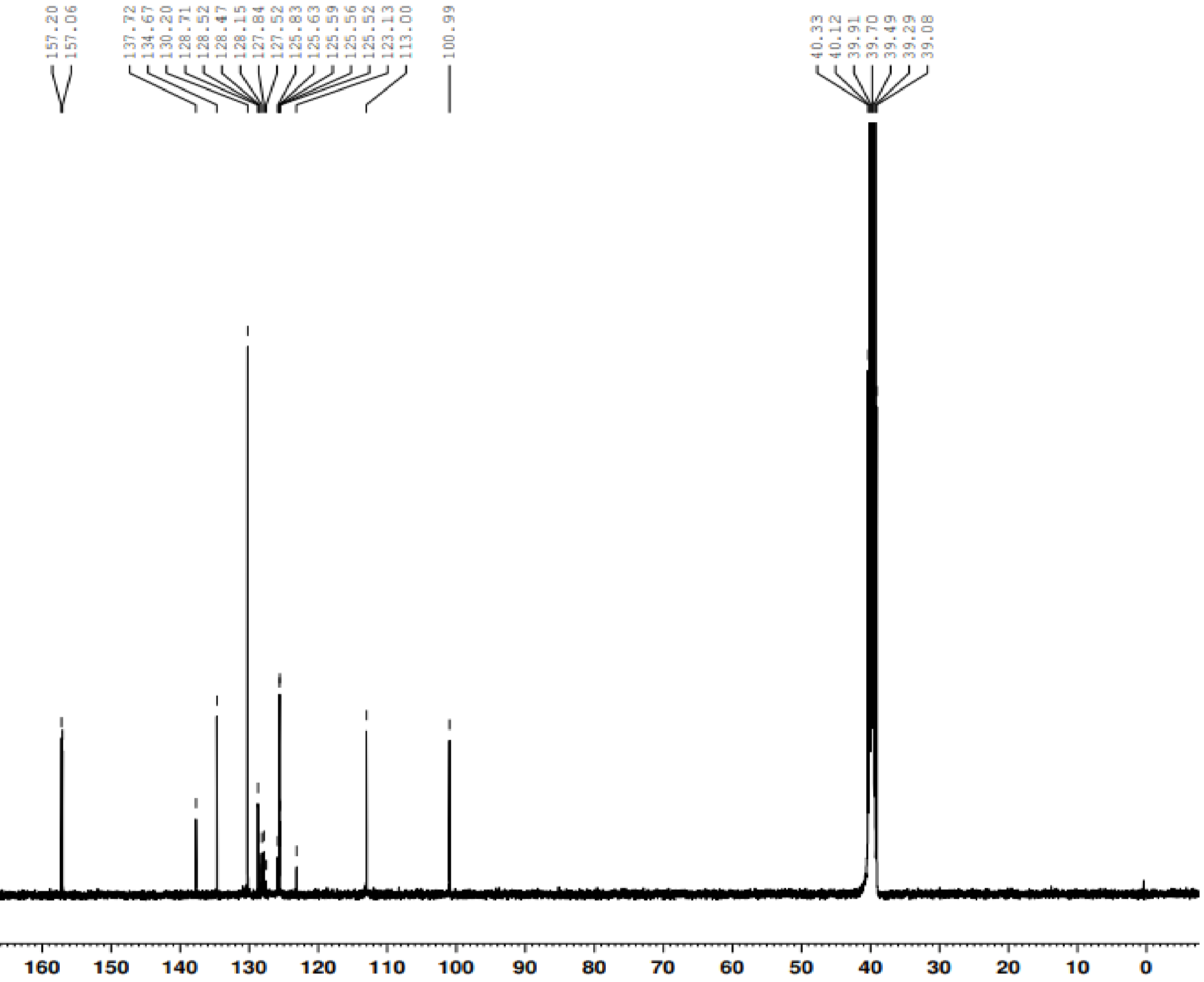

Figure S69. ^{1}H NMR spectrum (400 MHz, DMSO-d_6) of **1o**.

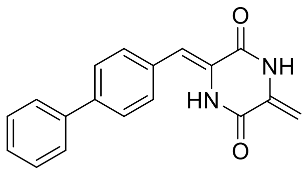

1o

Chemical Formula: $C_{18}H_{14}N_2O_2$

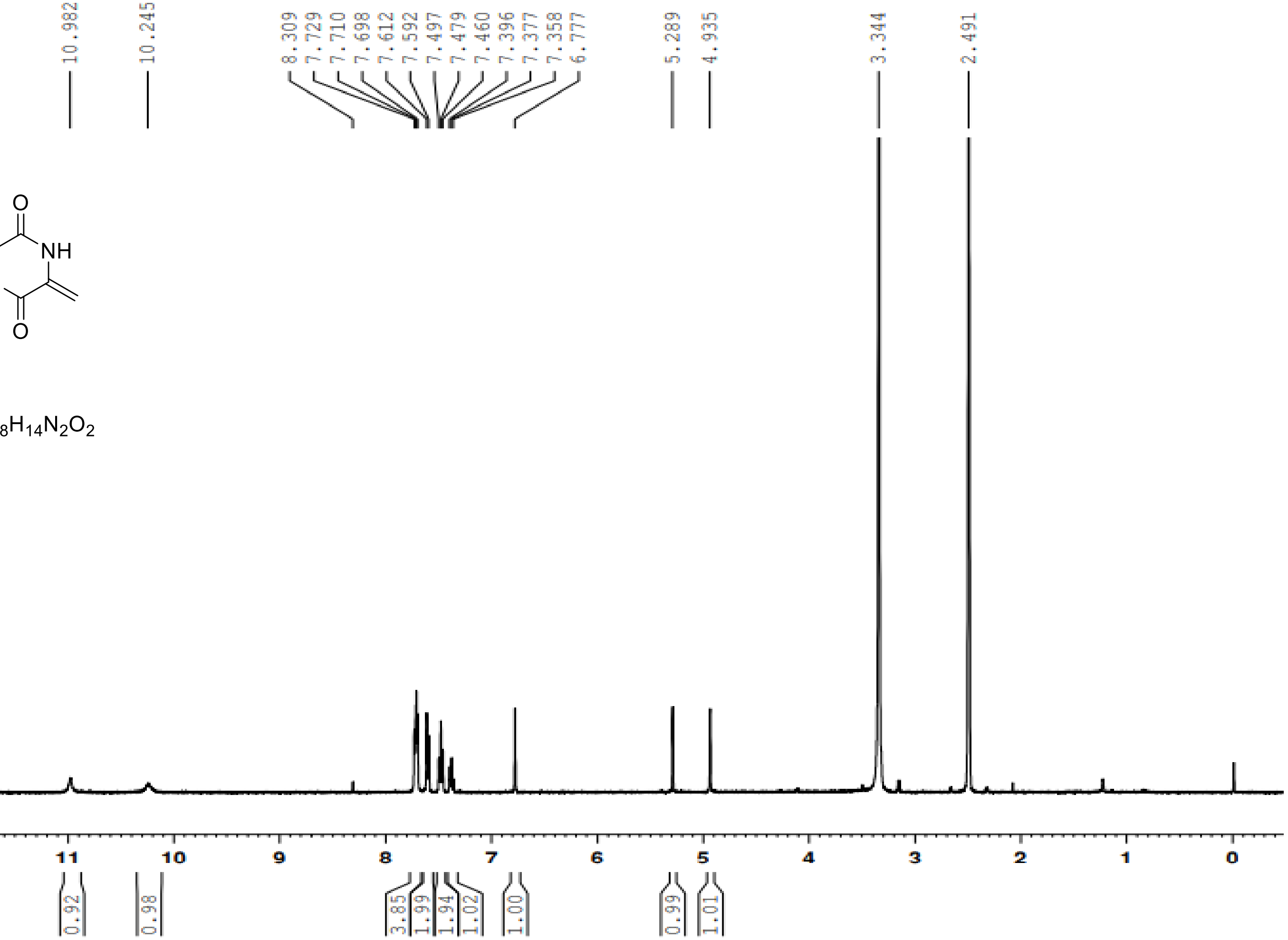

Figure S70. $^{13}C\{^{1}H\}$ NMR spectrum (100 MHz, DMSO-d_6) of **1o**.

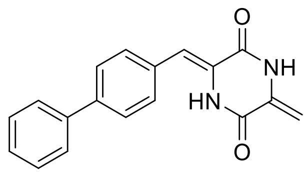

1o

Chemical Formula: $C_{18}H_{14}N_2O_2$

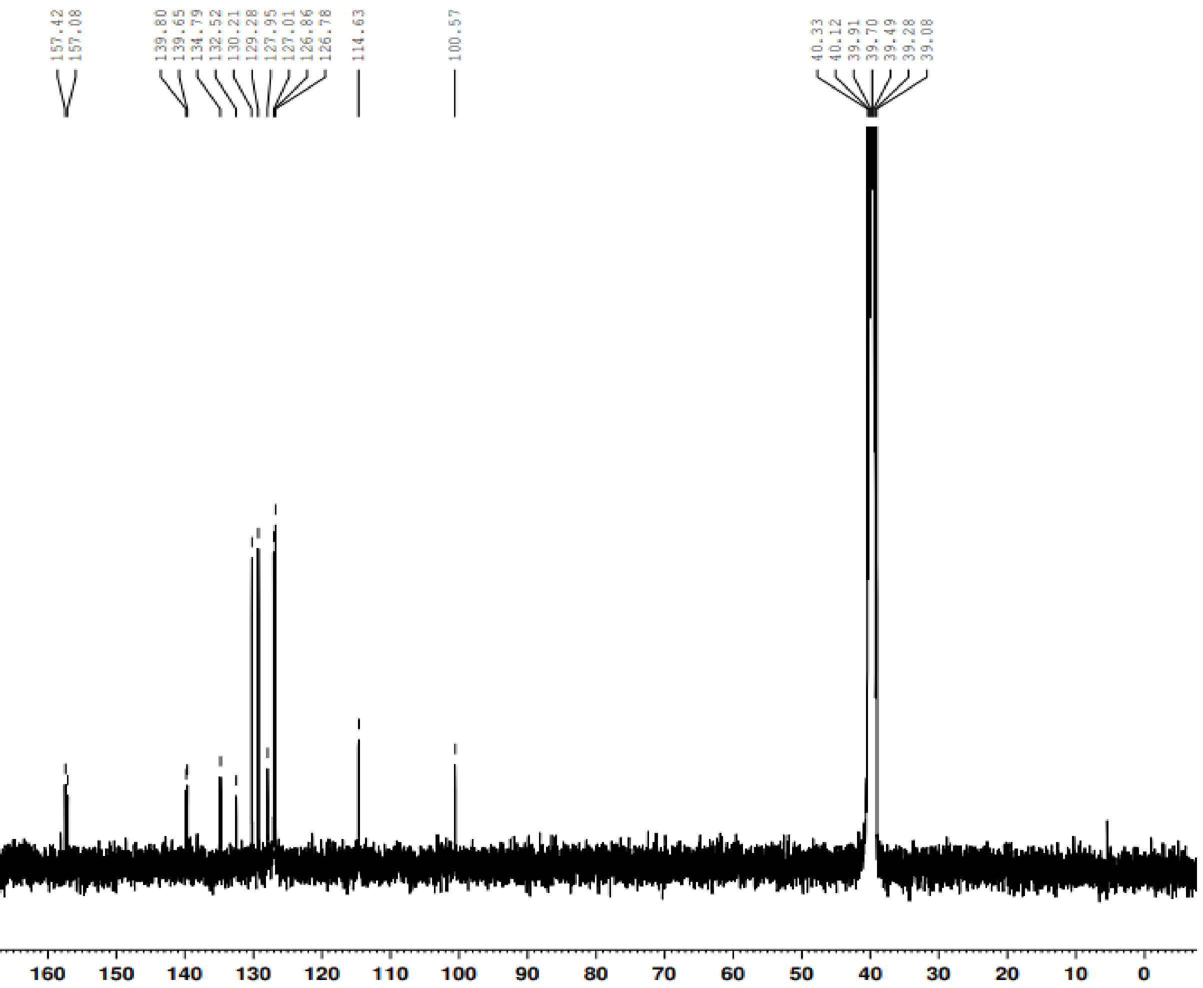

Figure S71. ^{1}H NMR spectrum (400 MHz, DMSO-d_6) of **1p**.

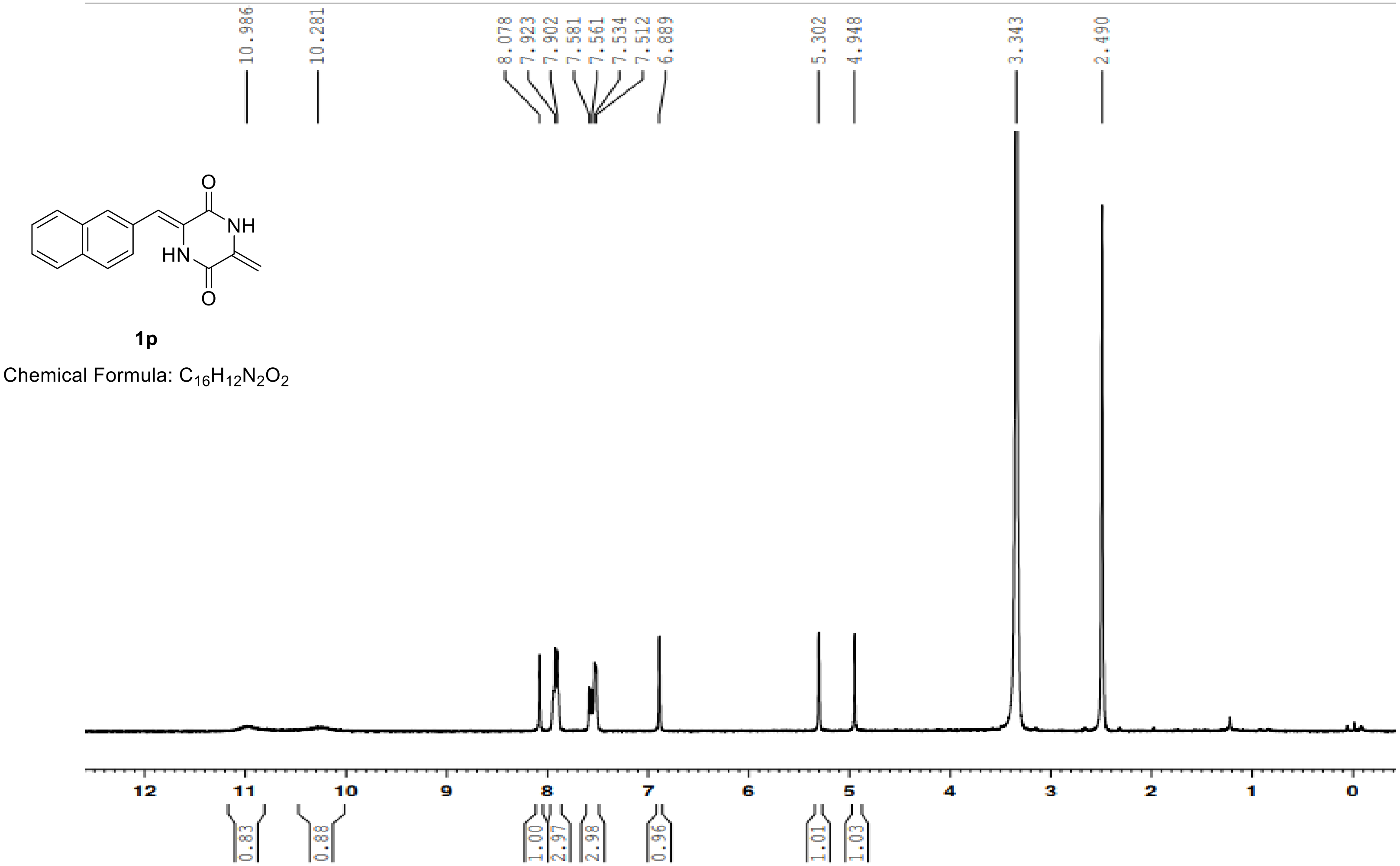

Figure S72. $^{13}C\{^{1}H\}$ NMR spectrum (100 MHz, DMSO-d_6) of **1p**.

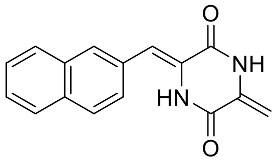

1p

Chemical Formula: $C_{16}H_{12}N_2O_2$

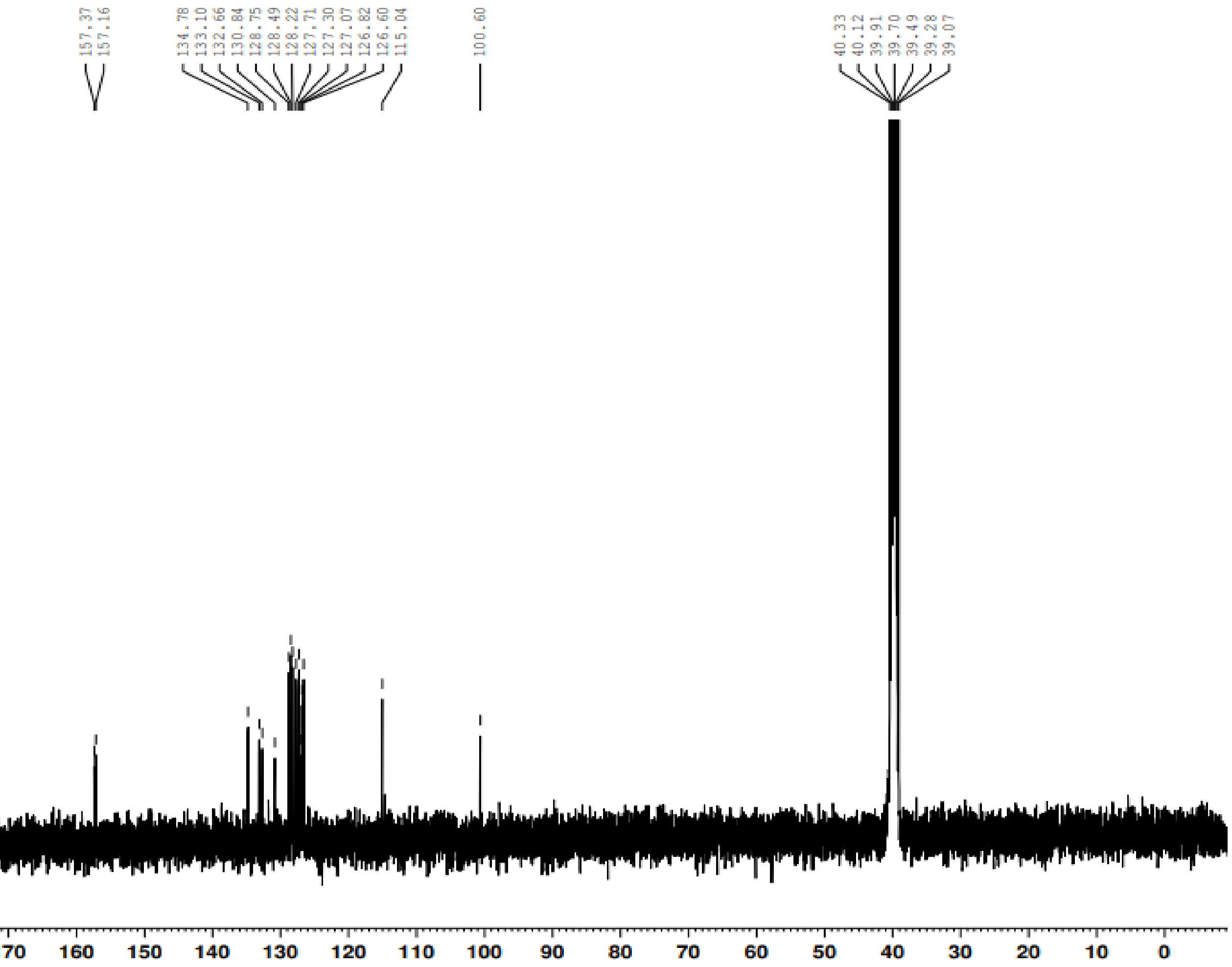

Figure S73. ^{1}H NMR spectrum (400 MHz, DMSO-d_6) of **1q**.

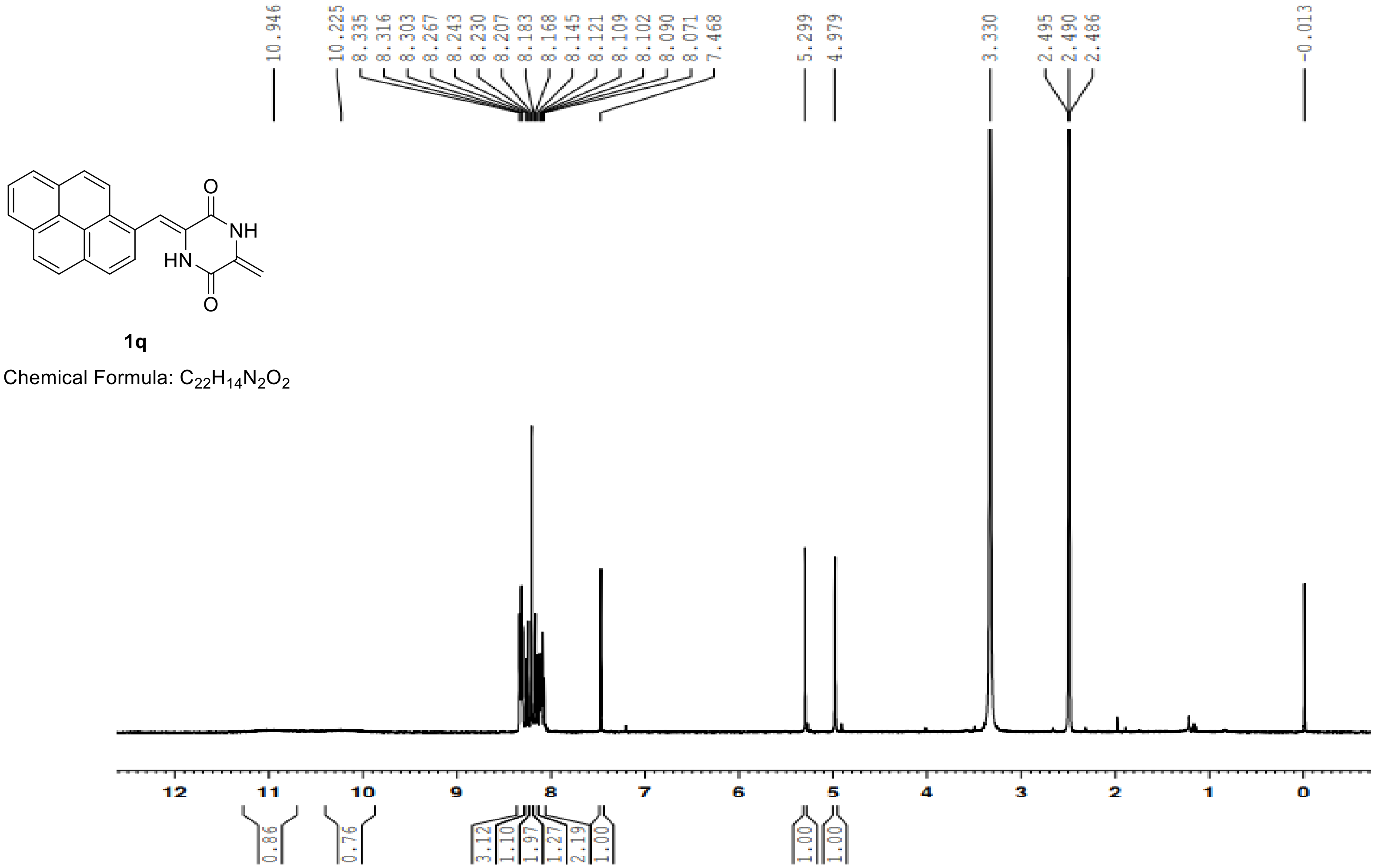

Figure S74. $^{13}C\{^{1}H\}$ NMR spectrum (100 MHz, DMSO-d_6) of **1q**.

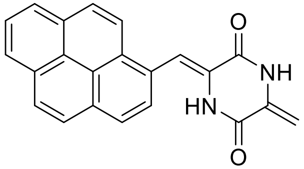

1q

Chemical Formula: $C_{22}H_{14}N_2O_2$

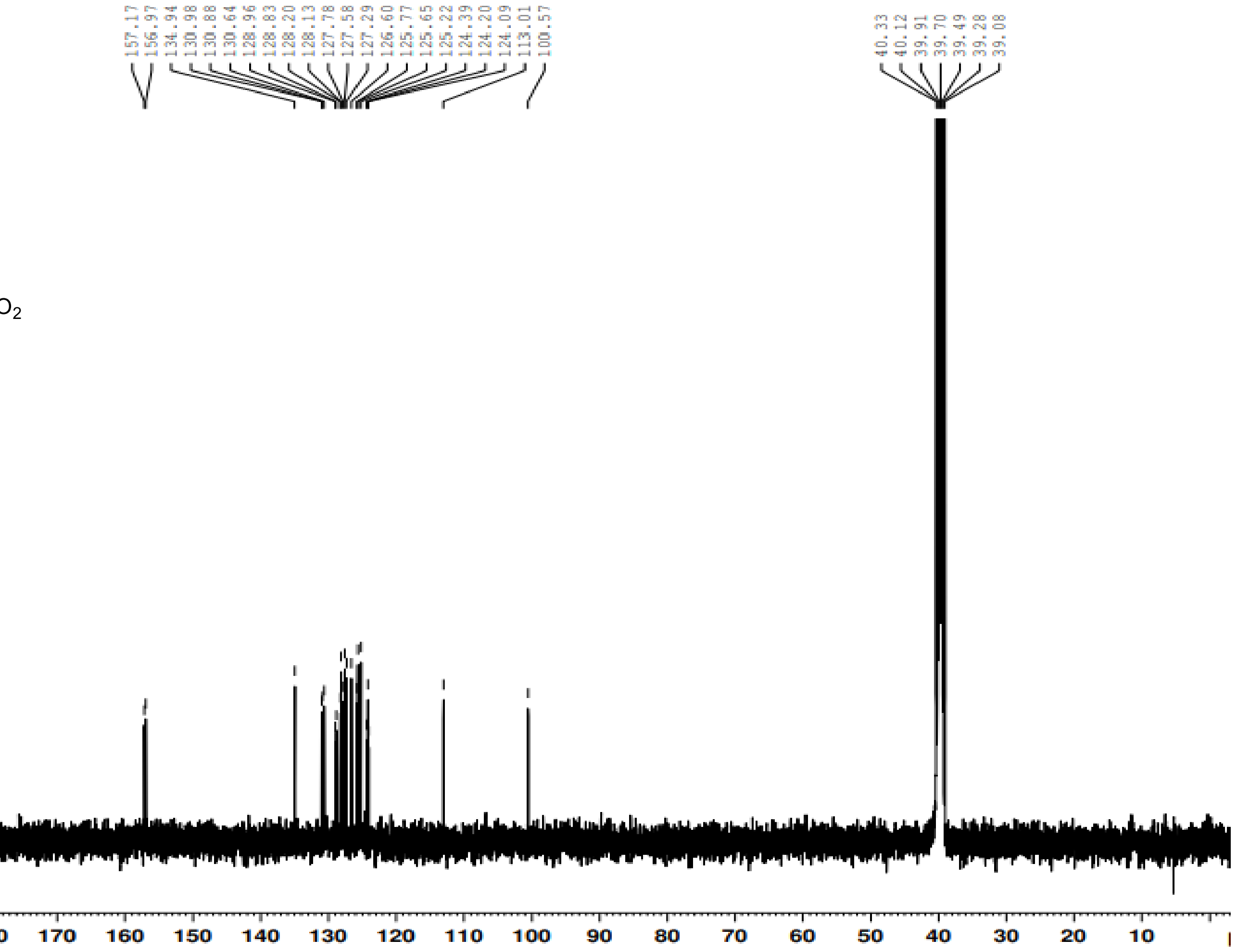

Figure S75. ^{1}H NMR spectrum (400 MHz, $CDCl_3$) of **1s**.

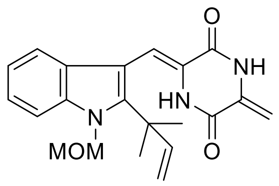

1s

Chemical Formula: $C_{21}H_{23}N_3O_3$

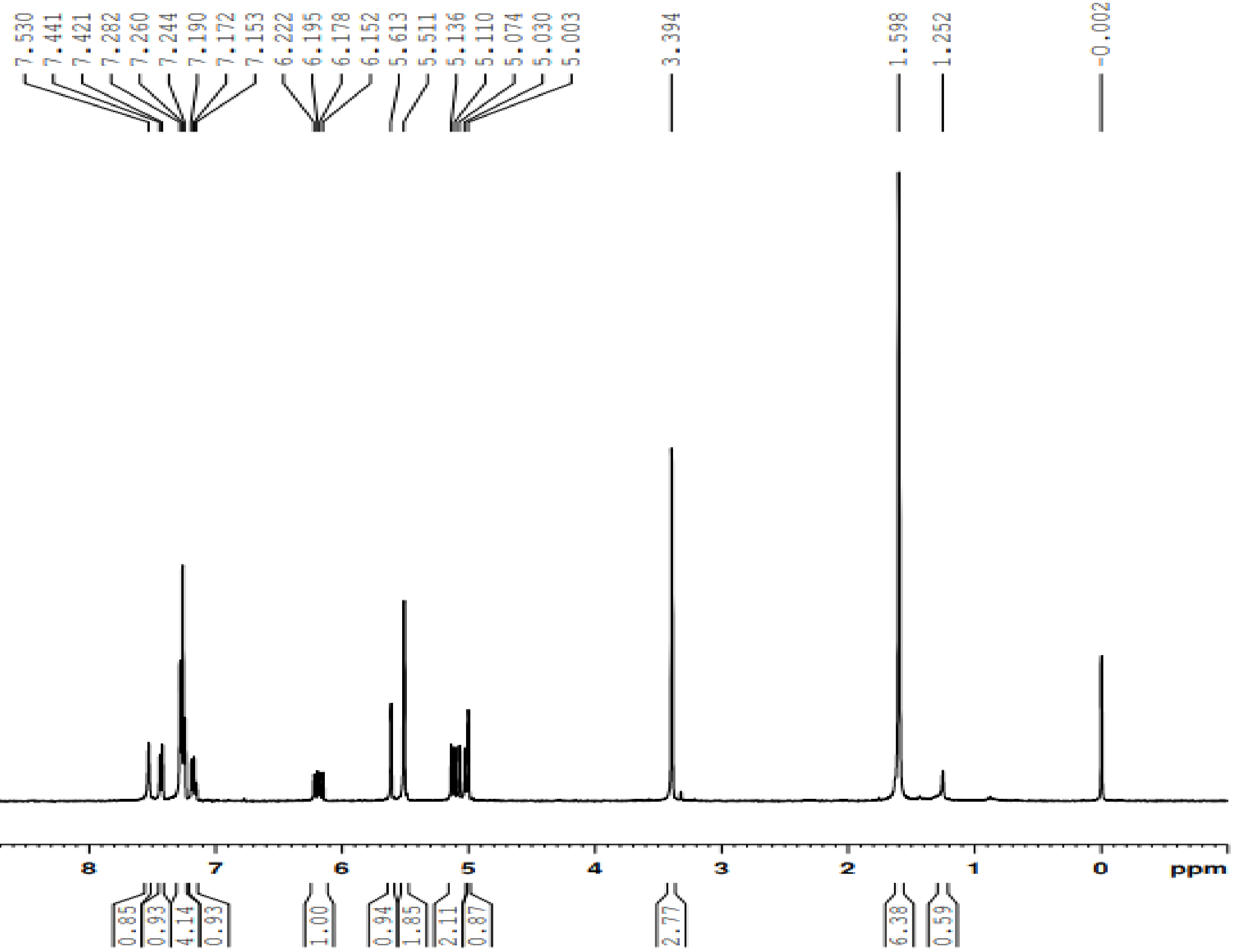

Figure S76. $^{13}C\{^1H\}$ NMR spectrum (100 MHz, $CDCl_3$) of **1s**.

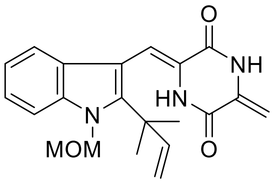

1s

Chemical Formula: $C_{21}H_{23}N_3O_3$

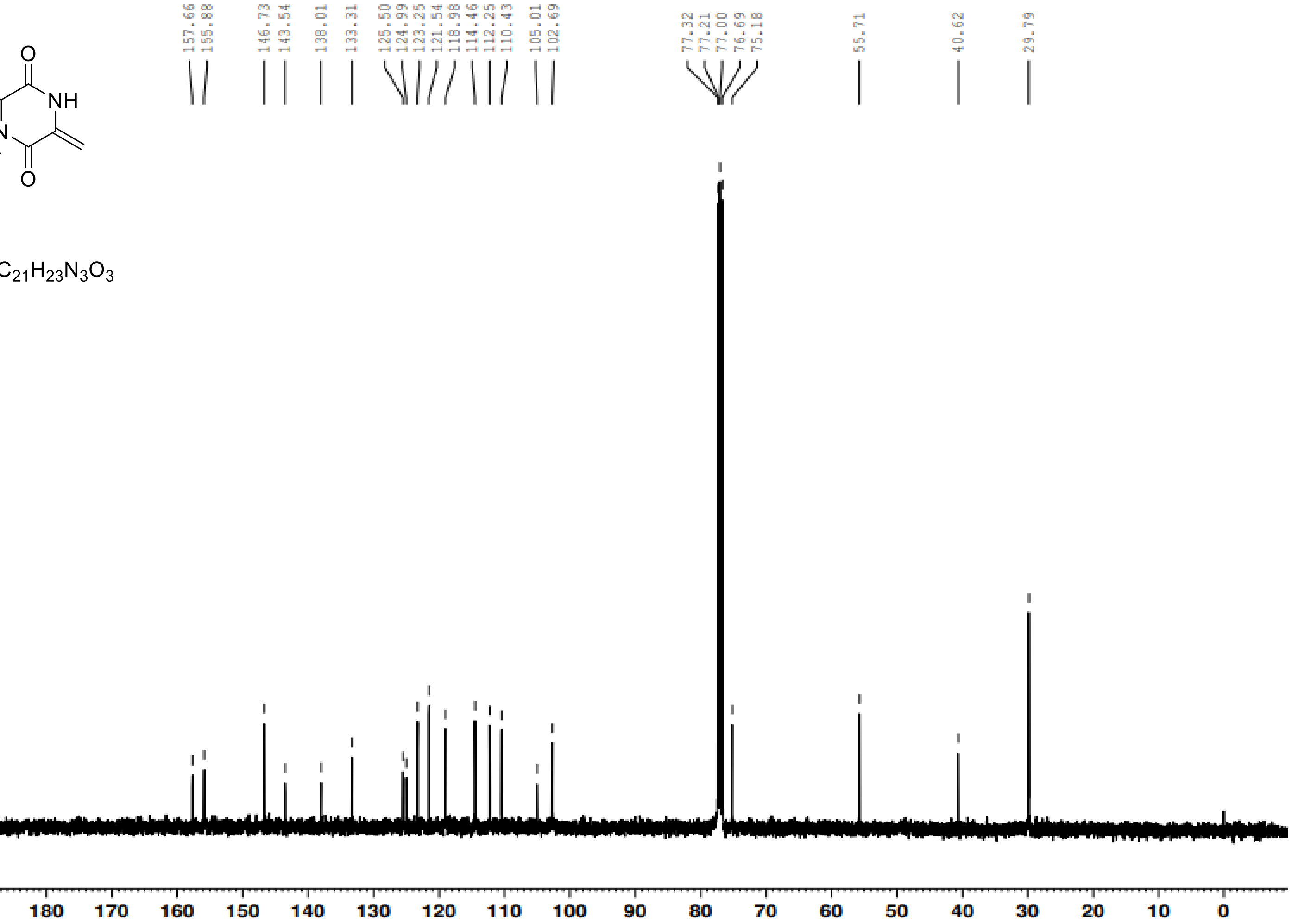

Figure S77. ^{1}H NMR spectrum (400 MHz, $CDCl_3$) of **6**.

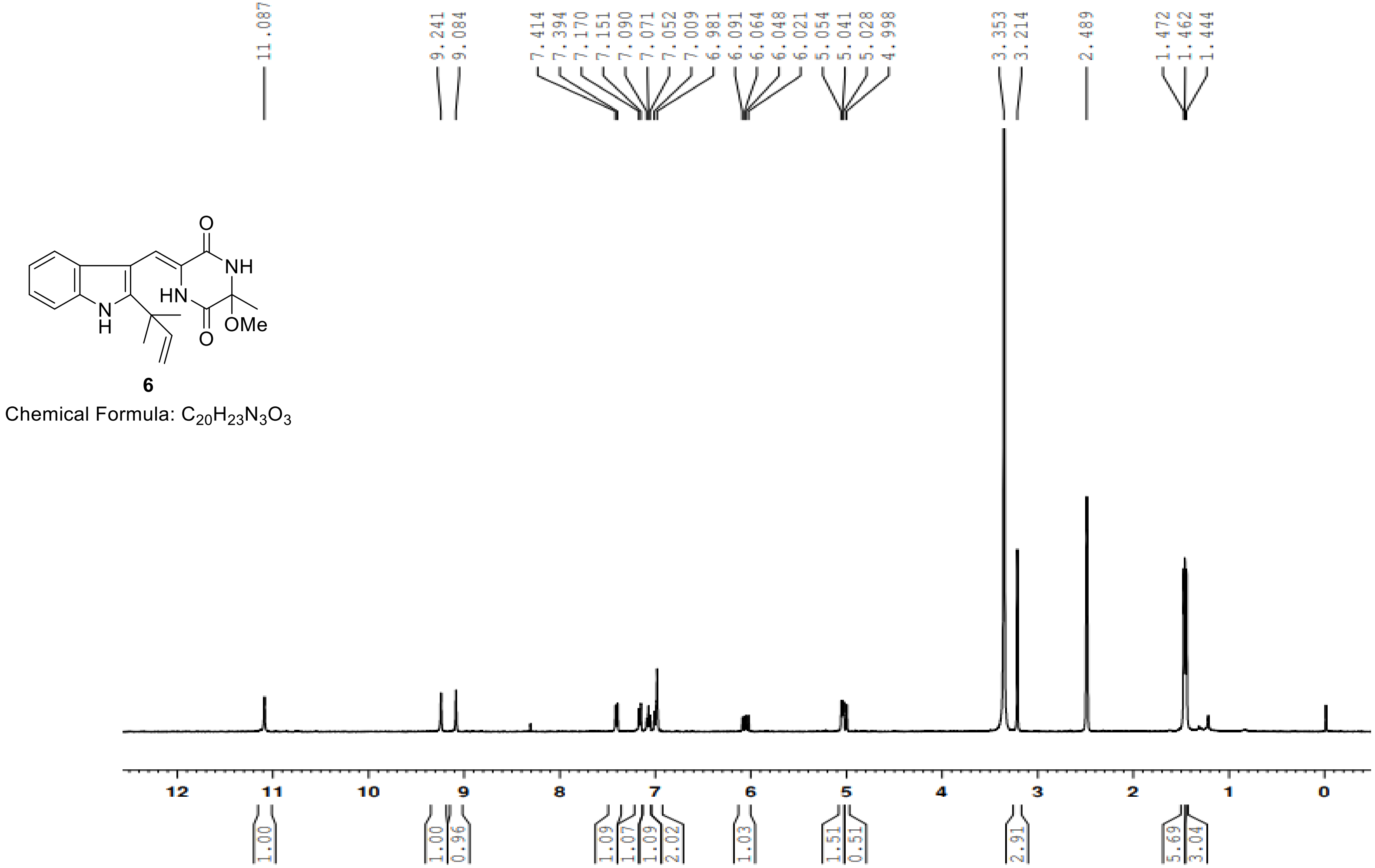

Figure S78. $^{13}C\{^1H\}$ NMR spectrum (100 MHz, $CDCl_3$) of **6**.

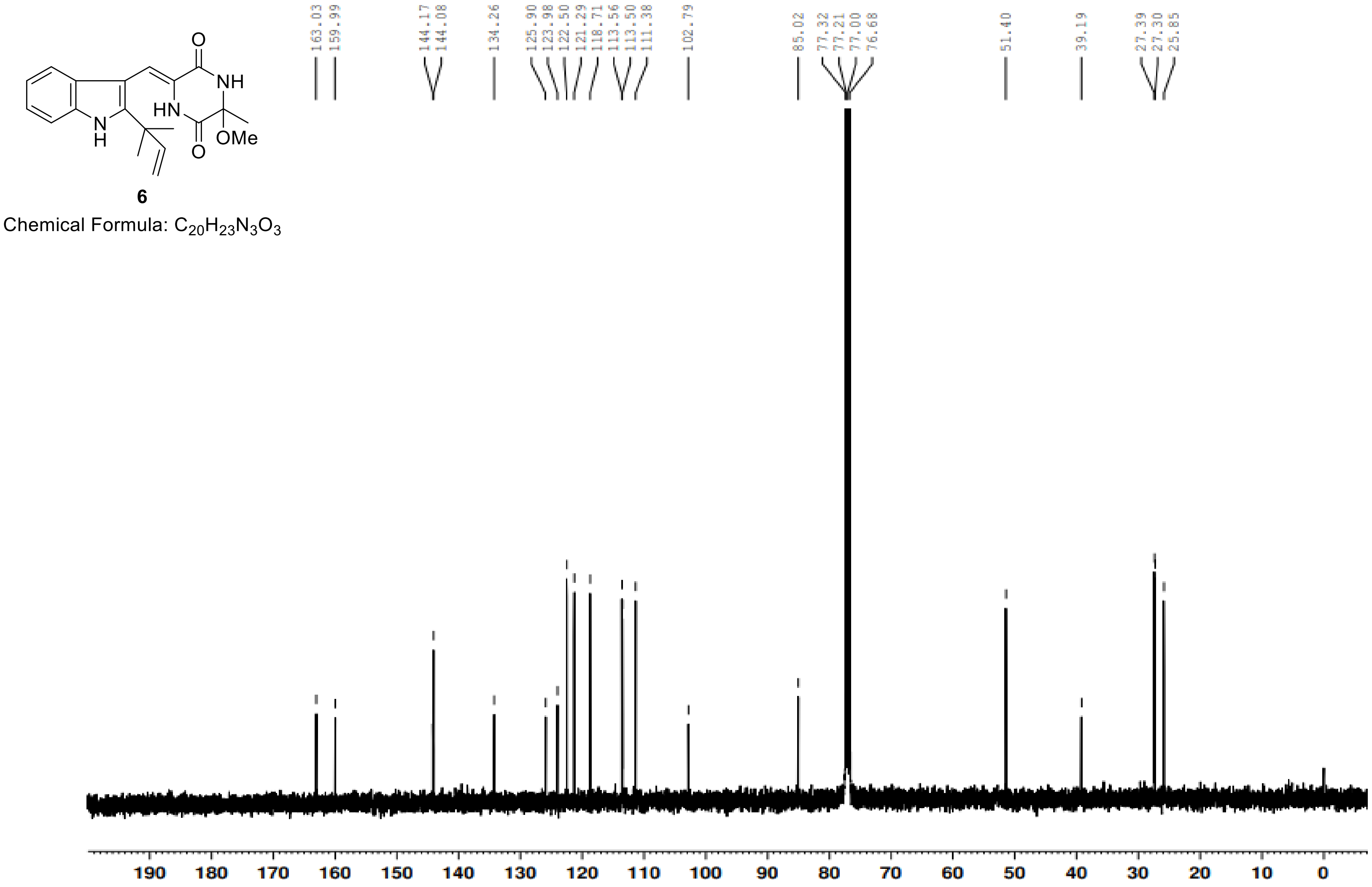

Supplement: Supplementary file 1 — np1c01120_si_001.pdf [file np1c01120_si_001.pdf]
